# Cancer/testis antigen‐Plac1 promotes invasion and metastasis of breast cancer through Furin/NICD/PTEN signaling pathway

**DOI:** 10.1002/1878-0261.12311

**Published:** 2018-06-14

**Authors:** Yongfei Li, Jiahui Chu, Jun Li, Wanting Feng, Fan Yang, Yifan Wang, Yanhong Zhang, Chunxiao Sun, Mengzhu Yang, Shauna N. Vasilatos, Yi Huang, Ziyi Fu, Yongmei Yin

**Affiliations:** ^1^ Department of Oncology The First Affiliated Hospital of Nanjing Medical University China; ^2^ Department of Pharmacology and Chemical Biology UPMC Hillman Cancer Center University of Pittsburgh PA USA; ^3^ Nanjing Maternal and Child Health Medical Institute Obstetrics and Gynecology Hospital Affiliated of Nanjing Medical University China

**Keywords:** breast cancer, cancer metastasis, cancer/testis gene, immunotherapy targets, prognostic factor

## Abstract

Placenta‐specific protein 1 (Plac1) is a cancer/testis antigen that plays a critical role in promoting cancer initiation and progression. However, the clinical significance and mechanism of Plac1 in cancer progression remain elusive. Here, we report that Plac1 is an important oncogenic and prognostic factor, which physically interacts with Furin to drive breast cancer invasion and metastasis. We have shown that Plac1 expression positively correlates with clinical stage, lymph node metastasis, hormone receptor status, and overall patient survival. Overexpression of Plac1 promoted invasion and metastasis of breast cancer cells *in vitro* and *in vivo*. Co‐immunoprecipitation and immunofluorescence cell staining assays revealed that interaction of Plac1 and Furin degraded Notch1 and generated Notch1 intracellular domain (NICD) that could inhibit PTEN activity. These findings are consistent with the results of microarray study in MDA‐MB‐231 cells overexpressing Plac1. A rescue study showed that inhibition of Furin and overexpression of PTEN in Plac1 overexpression cells blocked Plac1‐induced tumor cell progression. Taken together, our findings suggest that functional interaction between Plac1 and Furin enhances breast cancer invasion and metastasis and the Furin/NICD/PTEN axis may act as an important therapeutic target for breast cancer treatment.

**Abbreviations**
ATCC: American type culture collection
CTAs: cancer/testis antigens
DEGs: differentially expressed genes
Furin: phosphatase and tensin homolog
H&E: hematoxylin and eosin
HR: hormone receptor
IHC: immunohistochemistry
LNM: lymph node metastasis
MFS: metastasis‐free survival
NICD: Notch1 intracellular domain
OS: overall survival
Plac1: placenta‐specific protein 1
SI: staining index
STR: short tandem repeat

## 1 Introduction

Over the past decades, due to the improvement of early detection, as well as the development of more effective adjuvant and targeted therapies, the worldwide mortality of patients with breast cancer significantly decreases. Nevertheless, many patients with breast cancer remain incurable due to relapse and metastasis after surgery and adjuvant therapies (Benckert *et al*., 2012; Lin *et al*., 2017). When metastasis occurs, a major step is the functional activation of metastasis‐promoting genes, which accelerates a cascade of crucial steps, including the loss of adhesion of the primary tumor cells, invasion through surrounding extracellular matrix, intravasation into the blood and/or lymphatics with subsequent extravasation from microvasculature of distant organs, colonization, and proliferation at a metastatic site (Valastyan and Weinberg, 2011). Although progress has been made in identifying the key genes and pathways involved in tumor metastasis as therapeutic targets, the highly effective targets for therapy against breast cancer metastasis are still lacking. Thus, discovery of more effective targets for monitoring and treating metastatic tumors remains as a major challenge in the clinical management of breast cancer.

Cancer/testis antigens (CTAs) represent a group of genes whose expression is limited to human germ cells, but is aberrantly upregulated in a variety of malignant tumors, especially in advanced cancers with stem cell‐like characteristics (Kulkarni *et al*., 2012). The progression of cancer cells from primary tumor to metastasis resembles the process of germ cell colonization of the gonad. Trophoblasts invade and burrow into the endometrium to implant the embryo forming the nonmaternal part of the placenta that exhibits similar features as cancer cells (Li *et al*., 2017). Therefore, it is rational to hypothesize that CTAs also play important roles in tumor metastasis and promote the development of breast cancer, and thus likely serve as effective targets for breast cancer therapy. Previous studies have demonstrated that a number of CTAs are associated with metastasis in various cancers, including lung, colon, prostate, cervical, gastric, and breast cancer (Gjerstorff *et al*., 2016; Shang *et al*., 2014; Simpson *et al*., 2005). However, it remains unclear about the association between the expression of CTAs and the cancer prognosis. Thus, it is critical to elucidate the roles of CTAs as biomarkers and therapeutic targets in breast cancer.

Placenta‐specific protein 1 (Plac1), a known CTA, is primarily expressed in cytomembrane of the trophoblastic lineage, and plays an important role in the development of human placenta, including trophoblast invasion and migration (Chang *et al*., 2014). While its expression is mostly restricted to placenta and testis in normal tissues, Plac1 is frequently activated and highly expressed in wide variety of human cancers (Devor and Leslie, 2013; Ghods *et al*., 2014; Liu *et al*., 2015; Silva *et al*., 2007; Wu *et al*., 2017). Moreover, Plac1 possesses cancer‐specific immunogenicity that makes it a potential target for cancer immunotherapy (Liu *et al*., 2012). Emerging evidence has indicated that Plac1 is involved in regulation of tumor metastasis and progression (Koslowski *et al*., 2007; Nagpal *et al*., 2013; Wu *et al*., 2017). For example, a previous study using siRNA inhibition of Plac1 in breast cancer cell lines effectively suppressed tumor migration and invasion (Koslowski *et al*., 2007). Nevertheless, the precise role of Plac1 in regulation of breast cancer metastasis and progression remains unclear. In addition, the effect of Plac1 on tumor progression *in vivo* and the correlation between its expression and clinical prognosis are completely unknown. Therefore, more robust investigation into the function of Plac1 in breast cancer is necessary.

The goals of this study are to explore the function of Plac1 in regulating breast cancer invasion and metastasis using *in vitro* and *in vivo* experiments and clinical specimens. Our findings suggest that Plac1 and its associated factors play important roles in breast cancer invasion and metastasis and may serve as an effective therapeutic target for treatment of this disease.

## 2 Materials and methods

### 2.1 Clinicopathological characterization of clinical breast cancer specimens

A total of 250 paraffin‐embedded breast cancer samples were obtained and diagnosed at The First Affiliated Hospital of Nanjing Medical University and Affiliated Obstetrics and Gynecology Hospital of Nanjing Medical University from 2006 to 2011. The detailed information on clinicopathological characteristics of these specimens is summarized in Table 1. The use of human tissues and written informed consent were provided by the Institutional Research Ethics Committee. The experiments were undertaken with the understanding and written consent of each subject. The study methodologies conformed to the standards set by the Declaration of Helsinki. The study methodologies were approved by the Nanjing Medical University ethics committee.

**Table 1:** Association of PLAC1 expression with clinicopathological features in breast cancer patients

Characteristic variables	All study patients *N* = 250	PLAC1 expression		_χ_^2^	*P*
		Low	High		
Age					
< 50	81	35	46	2.762	0.097
≥ 50	169	92	77		
Tumor size					
≤ 2 cm	52	32	20	3.029	0.082
> 2 cm	198	95	103		
Tumor grade					
Grade I–II	159	80	79	0.084	0.772
Grade III	91	47	44		
T stage					
T I–II	176	97	79	4.427	0.035^a^
T III	74	30	44		
Lymph node status					
Negative	141	85	56	11.637	0.001^a^
Positive	109	42	67		
HER2					
Negative	130	68	62	0.246	0.62
Positive	120	59	61		
HR (ER or PR)					
Negative	80	49	31	5.14	0.023^a^
Positive	170	78	92		

a Significant difference *P *<* *0.05. The chi‐square test shows possible significance of correlation between detection of Plac1 expression and clinicopathologic parameters. Lymph node status: (a), Negative: number of positive nodal metastasis ≤ 3. (b), Positive: number of positive nodal metastasis > 3.

### 2.2 Immunohistochemistry

Immunohistochemistry (IHC) analysis was blindly performed by two independent pathologists on the 250 paraffin‐embedded breast cancer tissues and the nude mouse organ tissue sections. The sections of paraffin‐embedded tissues were incubated with rabbit anti‐Plac1 polyclonal antibody (1 : 100; Abcam, Cambridge, MA, USA), rabbit anti‐Notch1 intracellular domain (NICD) polyclonal antibody (1 : 100; Abcam), rabbit anti‐MMP2 polyclonal antibody (1 : 100; Abcam), rabbit anti‐MMP9 polyclonal antibody (1 : 100; Abcam), or rabbit anti‐PTEN polyclonal antibody (1 : 100; Cell Signaling Technology, Danvers, MA, USA) at 4 °C overnight. The UltraVision Quanto Detection System (Thermo Fisher Scientific, Waltham, MA, USA) was used, and signals were visualized with DAB Quanto Chromogen provided in the same kit. The intensity of immunostaining was determined by combining the proportion of positively stained tumor cells and the intensity of staining (Zhang *et al*., 2017). Scores representing positive tumor cell proportions were graded as 0, < 5%; 1, 5–25%; 2, 26–50%; 3, 51–75%; and 4, > 75%. The intensity was graded as follows: 0, no staining; 1, weak staining; 2, moderate staining; and 3, strong staining. The staining index (SI) was calculated by multiplication of these two primary scores. The SI ≥ 6 was defined as the optimal cutoff, SI ≥ 6 was defined as high expression of Plac1, and the others were considered as Plac1‐low.

### 2.3 Cell cultures

The human breast cancer cell lines, MCF‐7 and MDA‐MB‐231, were obtained from American type culture collection (ATCC, Manassas, VA, USA). MCF‐7 and MDA‐MB‐231 cells were cultured in Dulbecco's modified Eagle's medium (DMEM; Thermo Fisher Scientific) with 10% FBS (HyClone, Logan, UT, USA). Those cell lines have been authenticated by short tandem repeat (STR) profiling. All cells were maintained at 37 °C with 5% CO_2_ environment.

### 2.4 RNAi and plasmid transfection

Lentivirus of Plac1 plasmid, Plac1 shRNA1, and Plac1 shRNA2 was custom‐designed and generated by Genepharma Co. Ltd (Shanghai, China). MCF‐7 and MDA‐MB‐231 cells were infected with the lentivirus of Plac1 plasmid, Plac1 shRNA1, or Plac1 shRNA2 and selected with 1 μg·mL^−1^ puromycin for 10 days. PTEN plasmid and Furin siRNAs were purchased from Genepharma Co. Ltd. The sequences of these siRNAs are listed in Table S1. Cells were seeded at 60‐mm plates and transfected with oligonucleotide or plasmids using Lipofectamine 2000 (Life Technologies, Carlsbad, CA, USA) according to manufacturer's instructions. Transfected cells were incubated at 37 °C with 5% CO_2_ for 48 h.

### 2.5 Cell migration and invasion assays

Migration and invasion assays were performed using migration chambers with an 8‐μm pore size membrane (Corning, Corning, NY, USA). Prior to invasion assay, 60 μL of DMEM with 2% Matrigel (BD Biosciences, San Jose, CA, USA) was added to an upper chamber. Cells (2 × 10^4^ for migration assay or 2 × 10^5^ for invasion assay) were resuspended in serum‐free DMEM and added to the upper chamber. DMEM with 10% FBS was added to lower chamber to allow cells to migrate or invade for 24 or 36 h, respectively. Migrated or invaded cells on the lower surface of the chamber membrane were fixed with 4% paraformaldehyde and then stained with 5% crystal violet (Sigma‐Aldrich, St Louis, MO, USA). Each experiment was performed in triplicate, and mean values of migrated or invaded cells were calculated.

### 2.6 RNA extraction and RT‐PCR

Total RNA was extracted from breast cancer cells using TRIzol (Life Technologies, Shanghai, China) and used in first‐strand cDNA synthesis with a First‐Strand Synthesis System for RT‐PCR (Takara, Dalian, China) according to the manufacturer's instructions. Each experiment was performed independently in triplicate. The PCR primers used to amplify the indicated genes are listed in Table S1. qRT‐PCR was performed with a Roche LightCycler 96 Real‐Time PCR System (Roche, Basel, Switzerland). Total RNA of Plac1 overexpression MDA‐MB‐231 cells and control cells were prepared for gene microarray.

### 2.7 Western blot analysis and co‐immunoprecipitation

Cells were harvested and lysed on ice for 10 min in RIPA buffer (50 mm Tris, 150 mm NaCl, 0.5% EDTA, 0.5% NP‐40) and centrifuged for 15 min at 13 000 ***g***. The concentration of total proteins was quantified by a colorimetric assay. 30 μg of total proteins was loaded and separated on 6%, 8%, or 10% SDS/PAGE gel and then transferred to poly(vinylidene difluoride) membrane. After blocking with 5% nonfat milk for 2 h at room temperature, the membrane was incubated with primary antibodies overnight at 4 °C. The antibodies used were antibodies to Plac1 (1 : 1000; Abcam), Furin (1 : 1000; Abcam), NICD (1 : 1000; Abcam), HES1 (1 : 1000; Cell Signaling Technology), PTEN (1 : 1000; Cell Signaling Technology), p‐AKT (1 : 1000; Cell Signaling Technology), MMP2 (1 : 1000; Abcam), and MMP9 (1 : 1000; Abcam). The membranes were incubated with secondary antibodies for 2 h, and proteins were then detected using the Pierce ECL chemiluminescence system (Thermo Fisher Scientific). The total proteins were extracted from MCF‐7 and MDA‐MB‐231 cells for co‐immunoprecipitation using IP/CO‐IP Kit (Thermo Fisher Scientific) according to the manufacturer's instructions. The bound proteins were analyzed by western blotting and proteome analysis.

### 2.8 Immunofluorescence cell staining

Breast cancer cells were fixed with 4% paraformaldehyde and then incubated in immunostaining blocking buffer (Beyotime P0102) for 1 h to permeabilize the cells and block nonspecific protein–protein interactions. For colocalization, cells were then incubated with anti‐Plac1 antibody (1 : 100; Abcam) and anti‐Furin antibody (1 : 100; Abcam), followed by Alexa Fluor^®^ 488 and Alexa Fluor^®^ 594‐secondary antibody, respectively (Yeasen, Shanghai China). Nuclei were detected using DAPI staining. For NICD nuclear import, cells were then incubated with anti‐NICD antibody (1 : 100; Abcam) followed by Alexa Fluor^®^ 594‐secondary antibody (Yeasen). Proteins were then detected using confocal microscopy.

### 2.9 Animal experiments

All animal studies were performed under the protocol approved by the Animal Research Committee of Nanjing Medical University in accordance with the ‘Guide for the Care and Use of Laboratory Animals’ and the ‘Principles for the Utilization and Care of Vertebrate Animals’. In studies using mouse liver and lung metastasis models, 20 mice were divided into two groups (10 mice per group): One group was injected with mixed populations from Plac1‐overexpressing MDA‐MB‐231 cells and the other group with MDA‐MB‐231 cells transfected with empty vector. Cells were harvested by trypsinization and washed twice with PBS. The cells were then suspended in PBS. Approximately 1 × 10^6^ cells in 100 μL PBS were injected into the tail veins of 4‐week‐old female athymic nude mice. All of the mice were sacrificed 6 weeks after the injection of cells and necropsied for the observation of visible metastatic lesions in the liver and lung. Metastatic nodules in each liver or lung were counted. Liver or lung tissues were embedded in paraffin for hematoxylin and eosin (H&E) staining for IHC analysis.

### 2.10 Statistical analysis

ibm spss statistics 20 (IBM, Armonk, NY, USA) and graphpad prism 7 (GraphPad Software, La Jolla, CA, USA) were used for the statistical analysis. The significance of differences was analyzed using two‐tailed Student's *t*‐test or a chi‐square test, as appropriate. The two‐tailed Pearson chi‐square test was used to examine the relationship between Plac1 expression and the clinicopathologic parameters. The correlations between Plac1 expression and metastasis‐free survival (MFS) curves and overall survival curves (OS) were assessed using Kaplan–Meier plots and compared with the log‐rank test. Multivariate Cox regression analyses were used to evaluate survival data. Differences were considered significant when the *P* values were < 0.05.

## 3 Results

### 3.1 Plac1 overexpression correlates with poor prognosis of breast cancer

**Figure 1:**
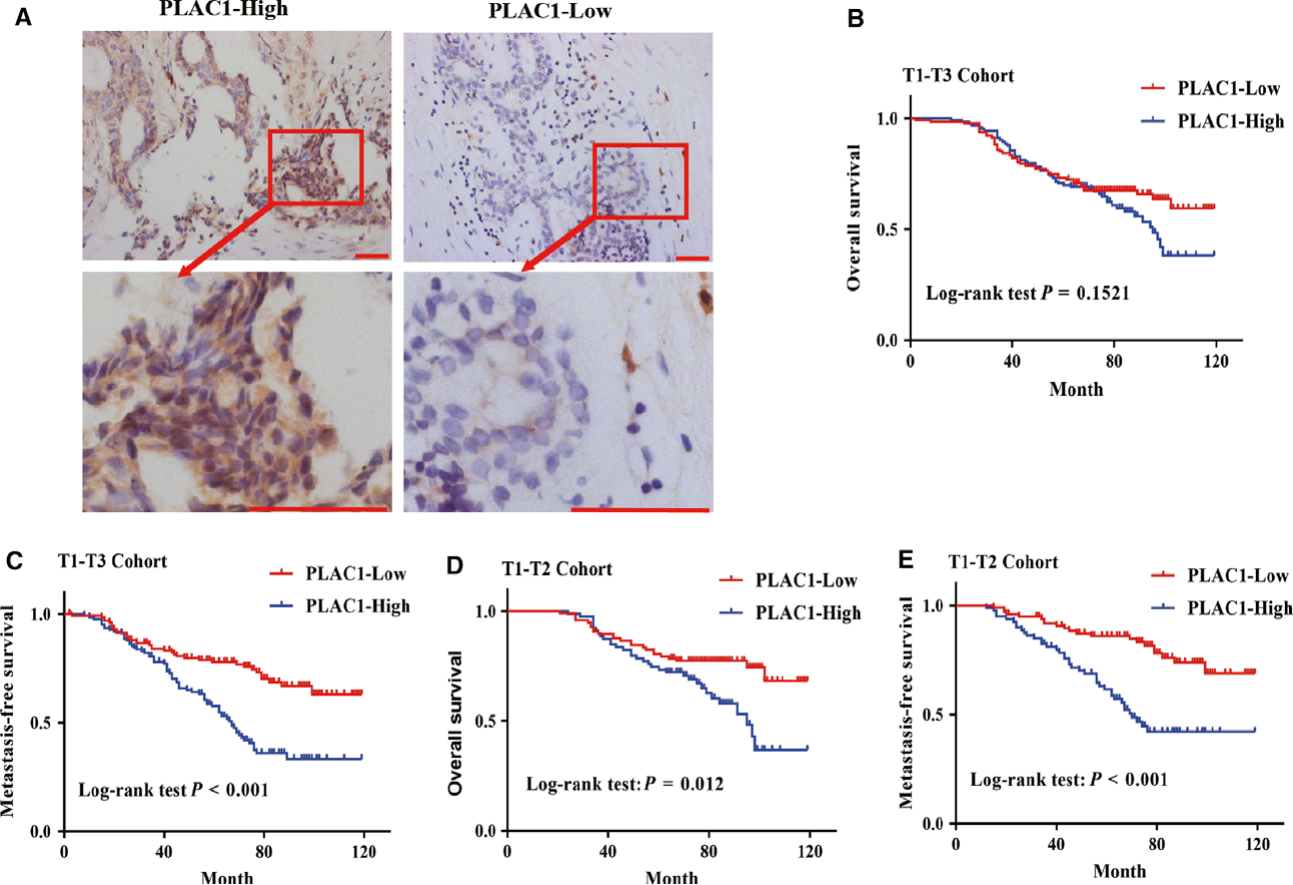
Plac1 overexpression is associated with poor prognosis in breast cancer. (A) Representative IHC staining of Plac1 from 250 paraffin‐embedded breast cancer tissues. IHC Scoring of Plac1 in breast cancer tissues. The intensity of Plac1 staining is scored by SI and grouped into low (SI < 6) and high (SI ≥ 6) expression. Scale bar, 50 μm. (B) Kaplan–Meier curve analysis showing OS of all breast cancer patients stratified by Plac1 expression level. (C) Kaplan–Meier curve analysis showing MFS of all breast cancer patients stratified by Plac1 expression level. (D) Kaplan–Meier survival analysis OS of patients with stages T1 and T2 stratified by Plac1 expression level. (E) Kaplan–Meier curve analysis MFS of patients with stages T1 and T2 stratified by Plac1 expression level.

To determine the pathologic correlation between Plac1 expression and breast cancer progression, 250 breast cancer tissues were evaluated for the correlation between Plac1 expression and established breast cancer prognostic factors (Table 1). The SI of Plac1 was calculated based on both the staining intensity and the proportion of positive cells. SI score of specimen ≥ 6 was defined as Plac1‐high, and the SI scores < 6 were considered as Plac1‐low (Fig. 1A). The expression level of Plac1 significantly correlated with clinical stage (*P *=* *0.035), hormone receptor (HR) status (*P *=* *0.023), and lymph node metastasis (LNM, *P *=* *0.001), which are known to be important features of breast cancer recurrence after resection, and are generally associated with a poor prognosis. Kaplan–Meier survival curves and log‐rank tests indicated that patients with high Plac1 expression had worse MFS (*P *<* *0.001; Fig. 1C). However, Plac1 was not obviously associated with differentiation, gender, age, HER2 status, or tumor size in patients with breast cancer (Table 1) and OS (*P *<* *0.152; Fig. 1B). In addition, multivariate Cox regression analysis revealed that Plac1 expression, LNM, HER2 status, and HR status were each recognized as independent prognostic factors for MFS in breast cancer (Table 2). These results demonstrate that overexpression of Plac1 might contribute to the metastasis of breast cancer.

**Table 2:** Multivariate analysis using Cox regression analysis demonstrates that PLAC1 is independent prognostic factor of breast cancer

Characteristic variables	OS		MFS	
	*P*	HR (95% CI)	*P*	HR (95% CI)
T1–T3 cohort (*N* = 250)				
Age	0.626	0.895 (0.573–1.399)	0.966	0.991 (0.648–1.516)
Tumor size	0.449	0.830 (0.515–1.340)	0.004^a^	0.516 (0.330–0.809)
Tumor grade	0.191	0.749 (0.485–1.155)	0.902	0.975 (0.646–1.471)
T stage	0.002^a^	1.937 (1.267–2.962)	0.001^a^	1.947 (1.229–2.918)
Lymph node status	0.890	1.031 (0.674–1.575)	< 0.001^a^	2.199 (1.445–3.345)
HER2	< 0.001^a^	0.345 (0.217–0.549)	0.003^a^	0.513 (0.332–0.793)
HR (ER or PR)	< 0.001^a^	0.231 (0.149–0.359)	< 0.001^a^	0.301 (0.194–0.468)
PLAC1 expression	0.112	1.409 (0.923–2.149)	< 0.001^a^	2.261 (1.480–3.454)
T1–T2 cohort (*N* = 176)				
Age	0.992	1.003 (0.551–1.827)	0.731	0.906 (0.518–1.587)
Tumor size	0.821	1.082 (0.548–2.133)	0.080	0.573 (0.307–1.069)
Tumor grade	0.727	0.901 (0.501–1.620)	0.915	1.032 (0.576–1.849)
Lymph node status	0.207	0.680 (0.373–1.238)	0.010^a^	2.056 (1.190–3.553)
HER2	0.002^a^	0.388 (0.214–0.703)	0.021^a^	0.513 (0.291–0.903)
HR (ER or PR)	< 0.001^a^	0.270 (0.151–0.481)	< 0.001^a^	0.306 (0.173–0.543)
PLAC1 expression	0.002^a^	2.547 (1.422–4.563)	0.001^a^	2.738 (1.549–4.804)

a Statistically significant *P *<* *0.05.

### 3.2 Plac1 is implicated in prognosis of early stage of breast cancer

To determine whether Plac1 may serve as a biomarker of early‐stage breast cancer, we performed Kaplan–Meier curve analysis on breast cancer patients with T1 and T2 stage disease. Log‐rank tests indicated that Plac1 overexpression correlated with worse OS (*P *=* *0.012; Fig. 1D) and shorter MFS (*P *<* *0.001; Fig. 1E). Multivariate Cox regression survival analysis showed that patients with overexpression of Plac1 were at higher risk of poor OS (*P *=* *0.002, HR: 2.547, 95% CI: 1.422–4.563) and poor MFS (*P *=* *0.001, HR: 2.738, 95% CI: 1.549–4.804; Table 2). This multivariate statistical analysis was adjusted for LNM, Plac1 level, tumor size, gender, age, and HER2 and HR status. These data demonstrate that Plac1 could be used as a potential prognostic factor in early‐stage breast cancer patients.

### 3.3 Plac1 promotes breast cancer cell migration and invasion

**Figure 2:**
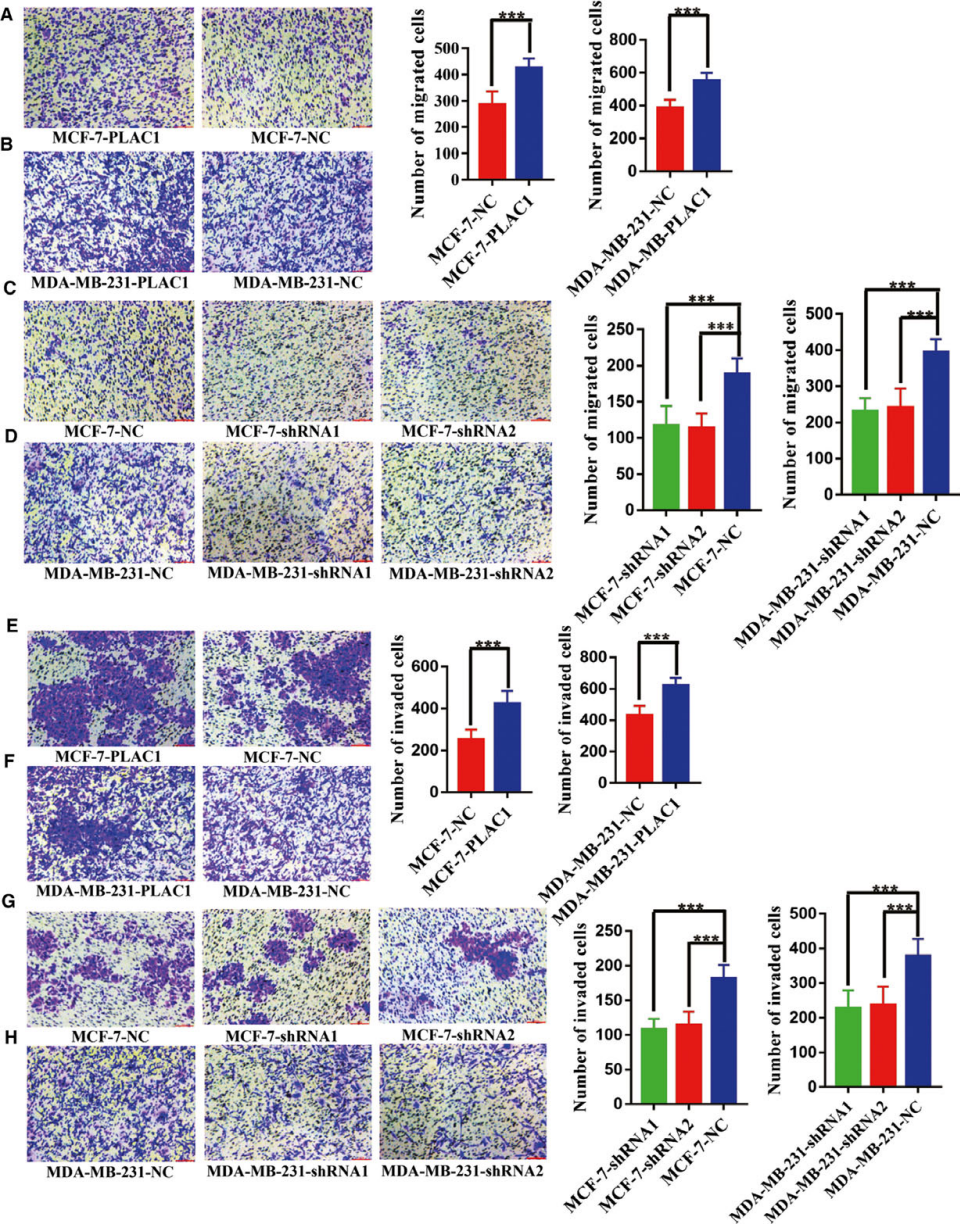
Functional studies uncover oncogenic characteristics of Plac1 *in vitro*. (A,B,E,F) Plac1 overexpression increases motility of MCF‐7 and MDA‐MB‐231 cells. Representative images (left) and quantitative data (right) of transwell assays measuring migration (A,B) or invasion (E,F). (C,D,G,H) Plac1 knockdown decreased motility of MCF‐7 and MDA‐MB‐231 cells. Representative images (left) and quantitative data (right) of transwell assays measuring migration (C,D) or invasion (G,H). All experiments were performed at least three times, and data were analyzed using the two‐tailed *t*‐test. ***P < 0.001 versus control. Error bars indicate SEM. Scale bar, 200 μm.

The results of our study of clinical samples demonstrated that Plac1 expression might be associated with tumor invasion and metastasis. Next, transwell migration and Matrigel invasion assays were used to investigate the effects of Plac1 on cell migration and invasion. qRT‐PCR and western blotting revealed different expression levels of Plac1 in breast cancer cells (MCF‐7, MDA‐MB‐231, BT‐474, MDA‐MB‐453, MDA‐MB‐468, HCC‐1937, SKBR3, and T47D; Fig. S1A,B). MCF‐7 and MDA‐MB‐231 cell lines were chosen for Plac1 overexpression and knockdown studies (Fig. S1C,D). Transwell migration and Matrigel invasion assays using Plac1‐overexpressing MCF‐7 and MDA‐MB‐231 cells showed that Plac1 overexpression facilitated cell migration and invasion (*P *<* *0.05; Fig. 2A,B,E,F), whereas depletion of Plac1 in MCF‐7 and MDA‐MB‐231 cells significantly inhibited cell migration and invasion (*P *<* *0.05; Fig. 2C,D,G,H). These data suggest that Plac1 is a critical factor in controlling breast cancer migration and invasion.

### 3.4 Plac1 physically interacts with Furin and regulates protein level of NICD

**Figure 3:**
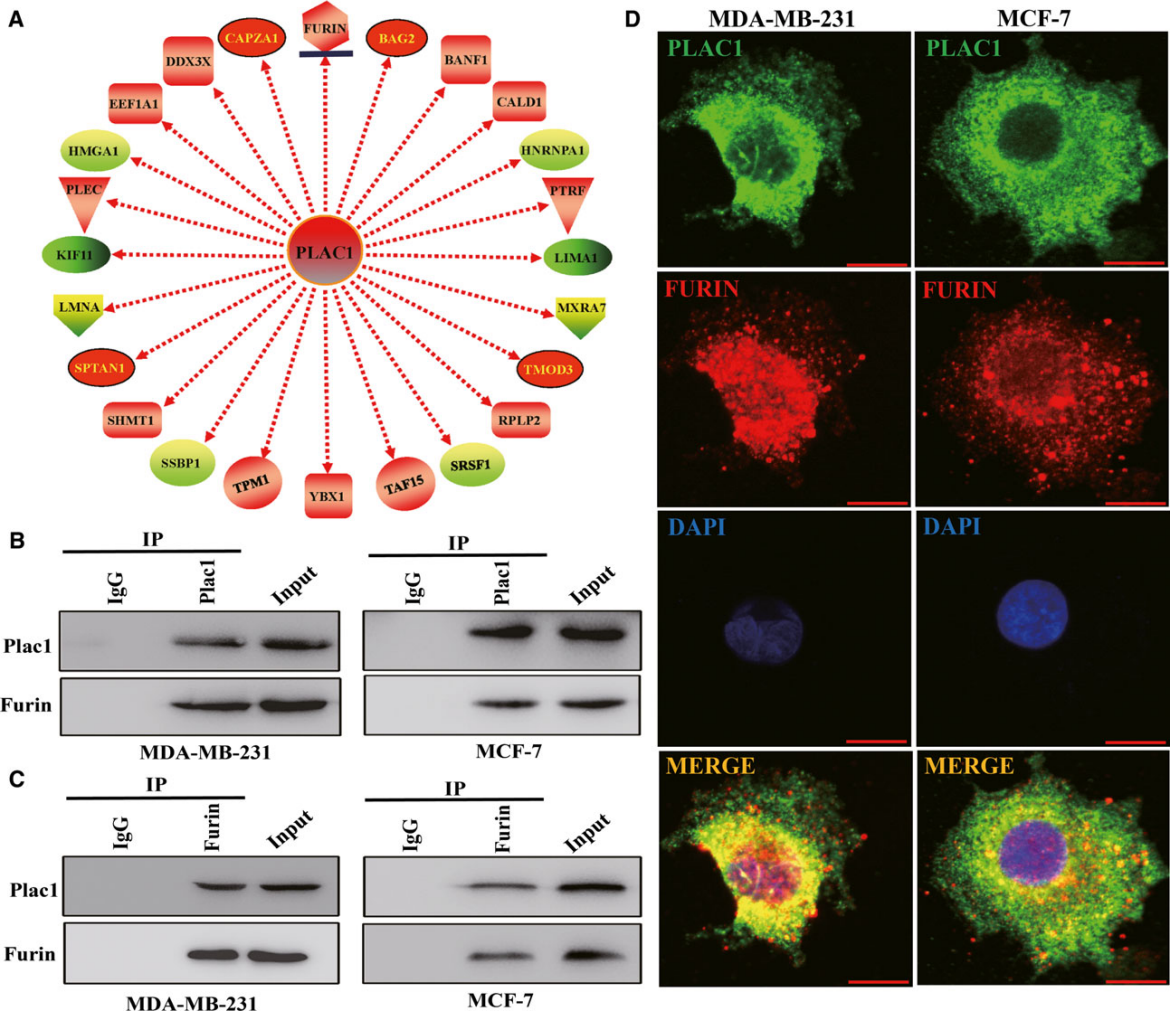
Plac1 physically interacts with Furin in breast cancer cells. (A) MCF‐7 cell lysates were immunoprecipitated with IgG control antibody or anti‐Plac1 antibody and then were analyzed by mass spectrometry analysis. Some of the proteins bound to Plac1 are displayed. B and C, Co‐immunoprecipitation assays of Plac1 and Furin in MCF‐7 and MDA‐MB‐231 cells. (B) Cell lysates were immunoprecipitated with IgG control antibody or anti‐Plac1 antibody and then immunoblotted with anti‐Plac1 or anti‐Furin antibody. (C) Cell lysates were immunoprecipitated with IgG control antibody or anti‐Furin antibody and then immunoblotted with anti‐Furin or anti‐Plac1 antibody. Plac1 and Furin expression in whole cell lysates (input) was shown. (D) Confocal images of Plac1 (green) and Furin (red) expression in MDA‐MB‐231 and MCF‐7 cells show Plac1 and Furin colocalized with each other. Cell nuclei were stained with DAPI (blue). Scale bars, 20 μm.

**Figure 4:**
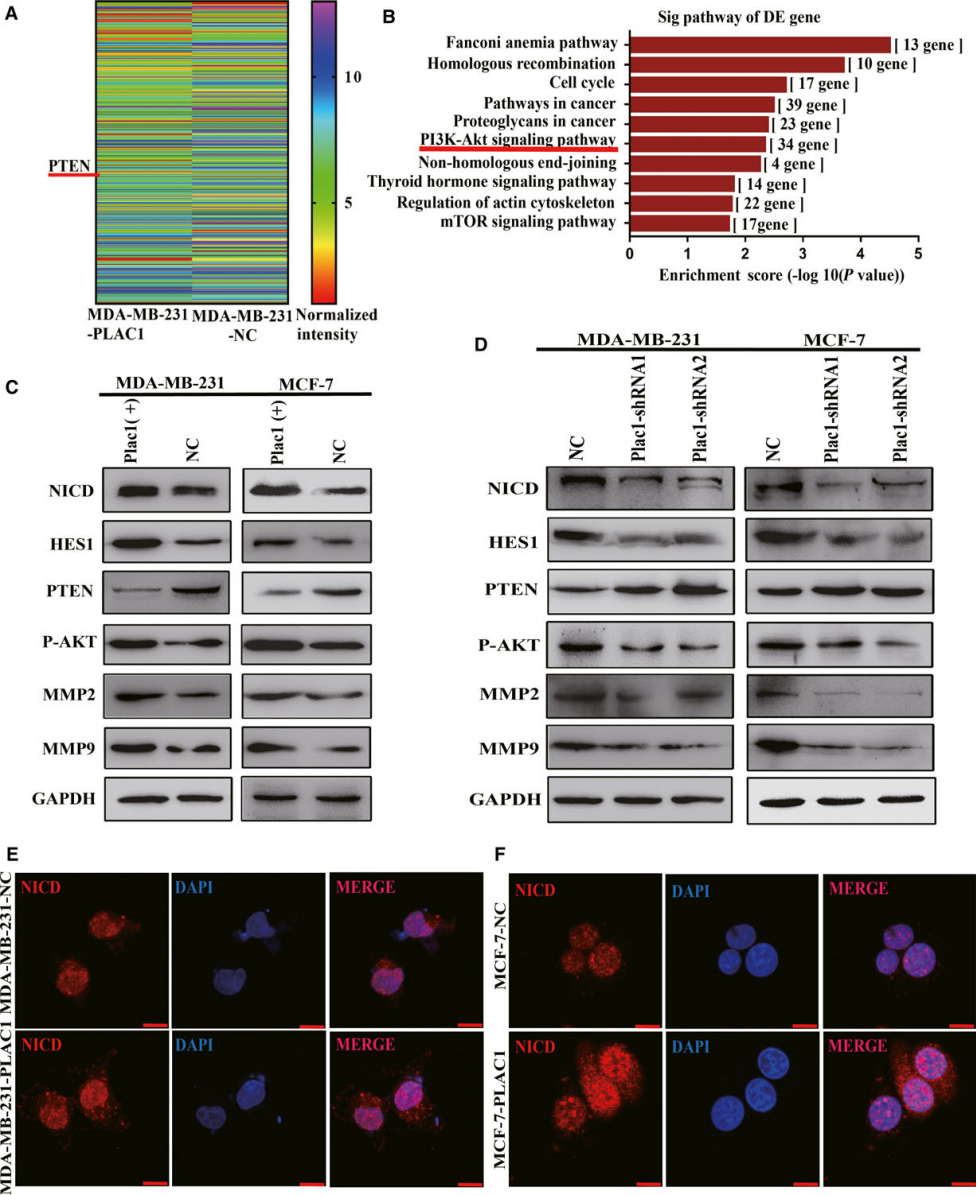
Plac1 regulates nuclear localization of NICD and induces AKT activity through downregulation of PTEN expression. (A) Gene microarray data showed the differences in gene expression between Plac1‐overexpressing MDA‐MB‐231 cells and control cells through heat map visualization. In Plac1‐overexpressing MDA‐MB‐231 cells, PTEN was suppressed versus control. (B) Pathway enrichment analysis showed that the DEGs induced by overexpression of Plac1 were enriched in the AKT signaling pathway. (C,D) Total cell lysates were prepared from each cell line and subjected to western blot analyses with the indicated antibodies. Plac1 overexpression (C) increases the level of NICD which suppresses PTEN to facilitate AKT activation. Plac1 silencing via RNAi (D) suppresses NICD expression and induces PTEN expression to facilitate AKT repression. E and F, Confocal images of NICD (red) expression are representative of nuclear localization. Plac1 increases nuclear localization of NICD in MDA‐MB‐231 (E) and MCF‐7 (F) cells. Cell nuclei were stained with DAPI (blue). Scale bars, 20 μm.

To investigate how Plac1 promotes breast cancer progression, we performed proteomic analysis of the complexes that were pulled down by the Plac1 antibody. Furin, a ubiquitously expressed type I transmembrane protein overexpressed in various cancers and correlative to their invasion and metastasis (Coppola *et al*., 2008; Thomas, 2002), was among the complex and selected for further study (Fig. 3A, Appendix S1). We used CO‐IP and immunofluorescence cell staining assays to assess whether Plac1 could physically interact with Furin. The result showed that Plac1 and Furin form a complex and interact with each other in MCF‐7 and MDA‐MB‐231 cells (Fig. 3B,C,D). The merged yellow signal in MDA‐MB‐231 cells was much more significant than in MCF‐7 cells through confocal microscopy (Fig. 3D). This result demonstrated that the complex of Plac1 and Furin in MDA‐MB‐231 cells was more than MCF‐7 cells (Fig. 3D). We next analyzed whether Plac1 could regulate expression of the Furin target gene, Notch1. Furin can degrade Notch1 and produce NICD fragments, which function as transcription factor. In Plac1‐overexpressing MCF‐7 and MDA‐MB‐231 cells, immunofluorescence cell staining assays revealed that the nuclear expression of NICD was higher than that in control cells (Fig. 4E,F). NICD is a well‐known transcription factor that regulates HES1 transcription; thus, the expression of HES1 was also regulated by Plac1 (Fig. 4C,D).

### 3.5 Plac1 promotes tumor cells’ invasion and metastasis through Furin/NICD/PTEN signaling pathway

**Figure 5:**
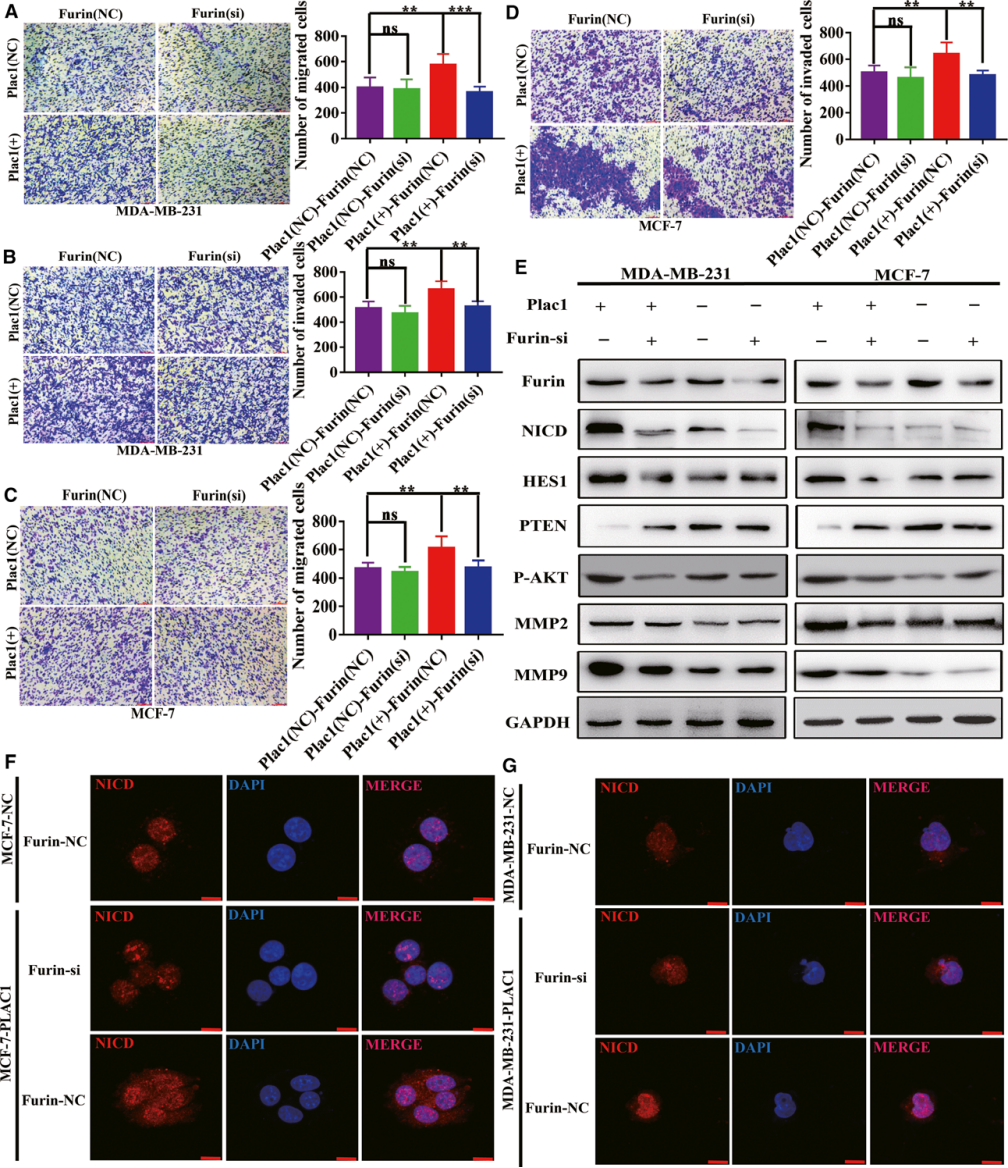
Plac1‐mediated cancer cell invasion and metastasis is reversed by Furin knockdown. (A,B,C,D) Plac1 overexpression increases invasion, and metastasis of MCF‐7 and MDA‐MB‐231 cells is reversed by Furin knockdown. Representative images (left) and quantitative data (right) of transwell assays measuring migration or invasion. All experiments were performed at least three times, and data were analyzed using the two‐tailed *t*‐test. **P < 0.01, ***P < 0.001. Error bars indicate SEM. Scale bar, 200 μm. (E) Total cell lysates were prepared from each cell line and subjected to western blot analyses with the indicated antibodies. Furin knockdown reverses Plac1‐induced NICD activation and PTEN suppression to facilitate AKT activation. (F,G) Confocal images of NICD (red) expression are representative of nuclear localization. Furin knockdown reverses Plac1‐induced nuclear localization of NICD in MDA‐MB‐231 and MCF‐7 cells. Cell nuclei were stained with DAPI (blue). Scale bars, 20 μm.

**Figure 6:**
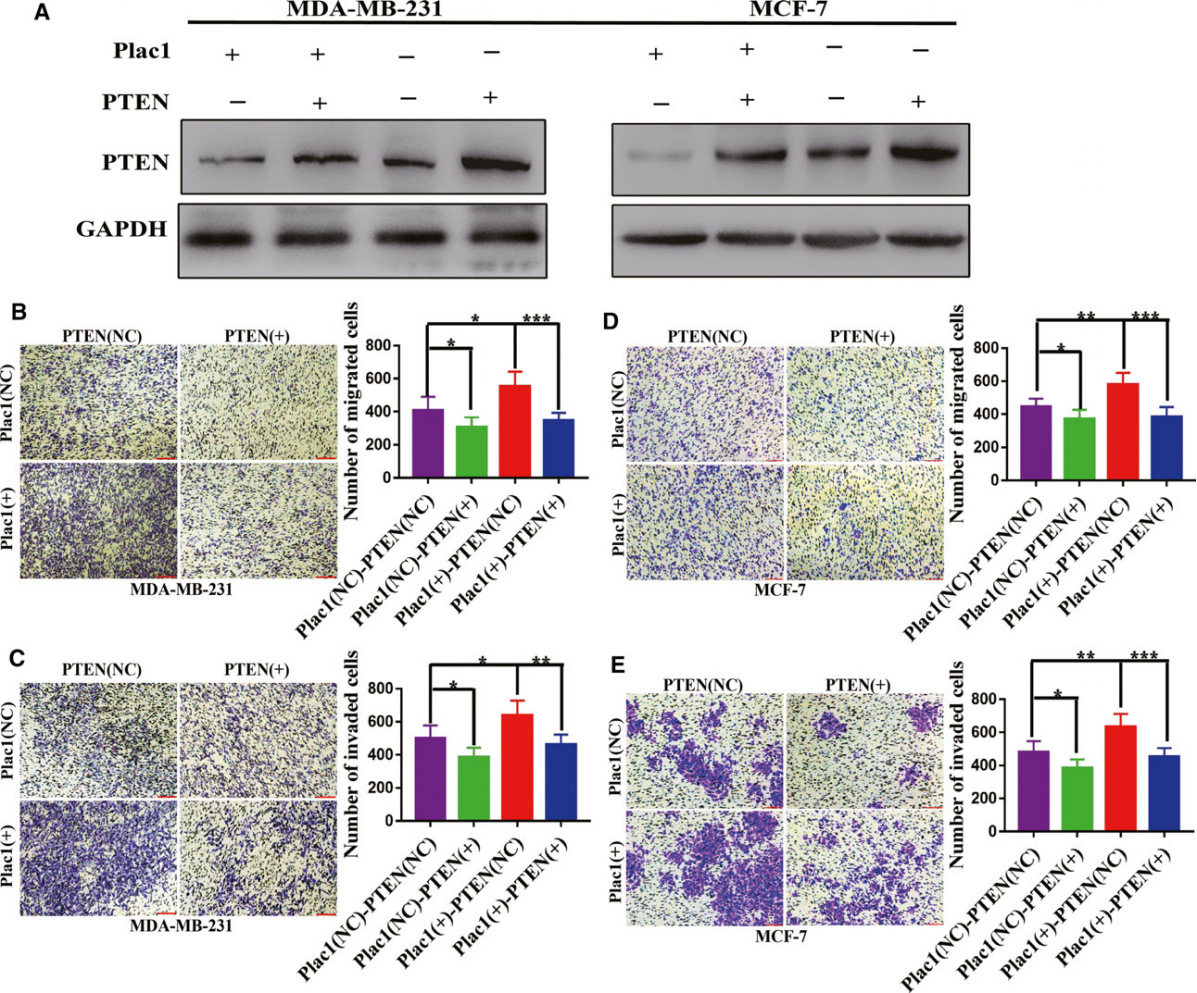
PTEN overexpression reverses Plac1‐mediated cancer cell invasion and metastasis. (A) After cells were transfected with PTEN plasmid, overexpression of PTEN was confirmed by western blot analysis. (B) (C,D,E) Invasion and metastasis of MCF‐7 and MDA‐MB‐231 cells by Plac1 overexpression are reversed by PTEN overexpression. Representative images (left) and quantitative data (right) of transwell assays measuring migration or invasion. All experiments were performed at least three times, and data were analyzed using the two‐tailed *t*‐test. *P < 0.05, **P < 0.01, ***P < 0.001. Error bars indicate SEM. Scale bar, 200 μm.

To investigate the mechanisms that contribute to Plac1‐mediated cell migration and invasion, a gene expression microarray study was carried out to detect gene expression differences between Plac1 overexpression and control MDA‐MB‐231 cells (Appendix S2). The results indicated that with overexpression of Plac1, the differentially expressed genes (DEGs) in the two groups were mainly enriched in AKT signaling pathway, including the tumor suppressor, phosphatase and tensin homolog (PTEN), whose expression was suppressed (Figs 4A,B and S2). Western blot was used to demonstrate that Plac1 regulates the PTEN/AKT signaling pathway, which further regulates MMP2 and MMP9 to promote tumor cell invasion and metastasis (Fig. 4C,D). We knocked down endogenous Furin in cells overexpressing Plac1, followed by transwell migration and Matrigel invasion assays. Results showed that depletion of Furin with siRNA significantly reduced the Plac1‐induced migration and invasion of MCF‐7 and MDA‐MB‐231 cells (Fig. 5A,B,C,D). Knockdown of Furin in Plac1‐overexpressing cells reversed the Plac1‐induced expression of NICD, HES1, PTEN, p‐AKT, MMP2, and MMP9 in MCF‐7 and MDA‐MB‐231 cells (Fig. 5E). Immunofluorescence experiments suggested that Plac1‐induced NICD nuclear localization was reversed by Furin knockdown (Fig. 5F,G). To further confirm that Plac1 promotes tumor cell invasion and metastasis through suppression of PTEN, we overexpressed PTEN in Plac1‐overexpressing cells, followed by transwell migration and Matrigel invasion assays. Results showed that PTEN overexpression also significantly reversed the Plac1‐induced migration and invasion of MCF‐7 and MDA‐MB‐231 cells (Fig. 6B,C,D,E). Those results confirm the previous studies that the transcription factor NICD may be responsible for the regulation of PTEN activity as HES1 acts as an upstream regulator of PTEN (Wong *et al*., 2012). Our data strongly demonstrate that Plac1 promotes tumor cell invasion and metastasis through Furin/NICD/PTEN signaling pathway.

### 3.6 Plac1 promotes invasion and metastasis *in vivo* via Furin/NICD/PTEN axis

**Figure 7:**
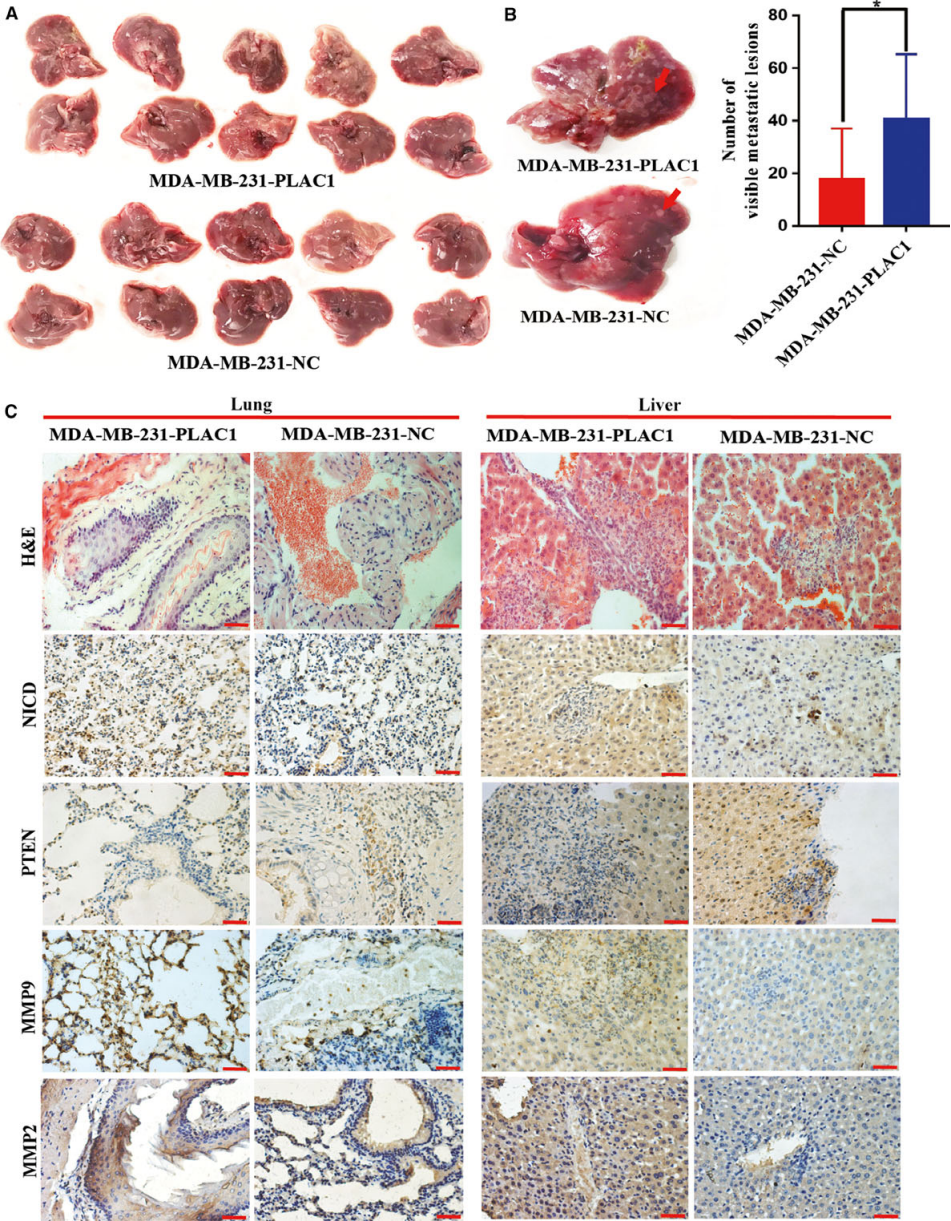
Plac1 promotes *in vivo* tumor metastasis through activation of the NICD/PTEN/MMP2/MMP9 axis. (A) MDA‐MB‐231 cells were injected into the tail veins of female athymic nude mice and followed over 6 weeks. Number of metastatic colonies from livers showing modest growth promotion in nude mice harboring MDA‐MB‐231 Plac1 overexpression versus MDA‐MB‐231 empty vector xenografts (*n* = 10/group). (B) Representative images of livers (left) and quantitative data (right) of mice harboring MDA‐MB‐231 Plac1 overexpression xenografts indicate number of metastatic colonies; *P < 0.05 versus control. (C) Representative images of lung and liver metastases from nude mice harboring MDA‐MB‐231 Plac1 overexpression or MDA‐MB‐231 empty vector xenografts, stained using H&E and immunostained for the indicated antibody. Scale bars, 50 μm.

To test whether overexpression of Plac1 promotes the metastasis of breast cancer cells *in vivo*, we injected Plac1‐overexpressing MDA‐MB‐231 cells or control cells into tail veins of nude mice. Results of experimental metastasis models showed that Plac1 overexpression in MDA‐MB‐231 cells significantly increased liver colonization of tumor cells (Fig. 7A,B). H&E staining and statistical analysis showed that the metastatic rate of Plac1‐overexpressing cells was substantially increased compared to control cells (Fig. 7C). To validate our findings, we conducted IHC staining in successive liver and lung sections from the mouse models. Our research group observed a positive correlation with nuclear NICD, MMP2, and MMP9 expression and correlation with PTEN expression in the Plac1‐overexpressing animal specimen (Fig. 7C). These data further suggest the existence of a Furin/NICD/PTEN axis by which Plac1 promotes tumor cell invasion and metastasis *in vitro* and *in vivo* in breast cancer.

## 4 Discussion

The current report provides clinical and experimental evidence to support the tumor‐promoting role of Plac1 in breast cancer. Our results uncover that patients whose tumors exhibit a high level of Plac1 are associated with high risk of axillary lymph node and distant metastasis, which is an independent prognostic factor in breast cancer. Furthermore, multivariate analysis indicated that Plac1 expression was an independent prognostic factor for OS and MFS. The mechanism of our Plac1 study reveals that Plac1 physically interacts with Furin, which generates NICD fragments to inhibit the expression of PTEN, thereby promoting tumor progression in human breast cancer. Those clinical and mechanistic data strongly demonstrate the important role of Plac1/Furin/NICD/PTEN signaling axis in breast cancer progression, which could serve as a potential target for metastatic breast cancer.

Previous studies have demonstrated that Plac1 could be a promising biomarker for the diagnosis and prognosis of many cancers (Devor and Leslie, 2013; Ghods *et al*., 2014; Tchabo *et al*., 2009). In prostate cancer, Plac1 expression was positively associated with the Gleason score and negatively correlated with prostate‐specific antigen expression (Ghods *et al*., 2014). Also, in hepatocellular carcinoma and gastric adenocarcinoma patients, Plac1 overexpression correlated with poor prognosis (Dong *et al*., 2008; Liu *et al*., 2015). However, the roles of Plac1 expression in promoting breast cancer are not understood. In this study, our results showed that high Plac1 protein expression was markedly associated with HR status, advanced TNM stage, and metastatic axillary lymph nodes, which are known to be important features of breast cancer recurrence after resection, and generally have a poor prognosis. These findings demonstrate that Plac1 could be a potentially favorable therapeutic index when translated clinically. Kaplan–Meier curve and multivariate analysis demonstrated that high Plac1 expression in breast cancer tissues was proven to be a significant and independent prognostic factor for MFS. Moreover, to investigate the prognostic value of Plac1 in patients with early‐stage breast cancer, 176 clinical stage T1 and T2 breast cancer cases were studied and revealed that Plac1 overexpression is correlated with worse OS and MFS. Stratification by clinical stage displayed similar or even higher prognostic significance of Plac1 than the widely employed patients. In tested breast cancer tissues, we found that patients with high Plac1 expression are associated with worse clinical outcomes, which indicates that level of Plac1 expression in breast cancer tissues may serve as a risk predictor for patients. The predictive value of prognosis based on Plac1 expression level indicates the anticipated availability as a novel molecular biomarker, particularly for patients with early‐stage disease. These data also highlight the potential usefulness of Plac1 for developing targeted breast cancer therapy.

Our current functional studies indicate that Plac1 has an oncogenic role to promote tumor migration and invasion, which is consistent with the results from a previous study (Koslowski *et al*., 2007). However, a prerequisite for promoting tumor cell invasion and metastasis is that individual cells break down cell–cell contacts and remodel cell–matrix adhesion sites (Principe *et al*., 2017). Plac1 appears to be responsible for upregulating the expression of MMP2 and MMP9, which degrade the extracellular matrix to promote tumor cell invasion and metastasis. To further elucidate the underlying mechanism by which Plac1 regulates the expression of MMP2 and MMP9, proteomic analysis, Co‐IP, and immunofluorescence assays were performed and revealed that Plac1 physically interacts with Furin, resulting in the downstream activation of the Notch1 signaling pathway with concomitant NICD and HES1 upregulation. The carcinogenicity of NICD has been demonstrated in various cancers such as lung, blood, head and neck, brain, and breast cancer (Mukherjee *et al*., 2016; Yeh *et al*., 2016; Yu *et al*., 2016). The roles of Plac1 in the progression of cancers have been directly ascribed to its processing activity that gives rise to the activation of cancer‐related signal pathway proteins‐Notch1 pathway (Broadus *et al*., 2016; Tao *et al*., 2014). By gain‐ and loss‐of‐function assays, we show that depletion of Furin in Plac1‐overexpressing breast cancer cells attenuated the Plac1‐induced expression of NICD, HES1, MMP2, and MMP9 and tumor cell invasion and metastasis. Thus, Plac1 increases the level of NICD to increase the invasion and metastasis of breast cancer cells through physically interacting with Furin. Those results suggest that tumor cell invasion and metastasis can be reduced by inhibiting the physical interaction of Plac1 and Furin, which provides a therapeutic strategy for suppressing invasion and metastasis of breast cancer.

In a previous study, Koslowski *et al*. (2007) found the level of p‐AKT downregulated by knockdown of Plac1 expression in breast cancer cell lines. Our microarray data demonstrate that AKT alteration is caused by inhibition of PTEN in Plac1‐overexpressing cells. Our rescue experiments further confirmed that PTEN could reverse the tumor cell metastasis and invasion induced by Plac1. PTEN is often downregulated in cancers and functions to control important cellular processes, including survival, proliferation, migration, and invasion, which is vital for tumor cells to disseminate to adjacent or distant tissues, and finally causes distant metastasis‐related recurrence (Davies *et al*., 2015; Jaraiz‐Rodriguez *et al*., 2017). More importantly, PTEN gene transcription was regulated by Notch1 signaling pathway activation in various cancers (Hu *et al*., 2014; Kim *et al*., 2016; Zhou *et al*., 2013). In this study, we also found that increased phosphorylation of AKT in breast cancer cells through Plac1‐induced PTEN downregulation can be reversed by Furin knockdown, suggesting that Plac1 controls conversion of cancer cells into an invasive and metastatic state by regulating the Furin/NICD/PTEN/AKT axis. In other words, interaction of Plac1 and Furin may be sufficient to alter this pathway, further enhancing MMP2 and MMP9 protein levels to promote the invasion and metastasis of breast cancer cells. Based on these data, we can further conclude that Plac1 regulates invasion and metastasis through stimulation of Furin/NICD/PTEN/AKT axis. This pathway may be a new target for breast cancer treatment.

The function of Plac1 in promoting breast cancer aggressiveness provides a new mechanism for explaining the poorly understood metastasis and poor survival of breast cancer patients with aberrant Plac1 overexpression. We demonstrated that Plac1 regulates invasion and metastasis via Furin/NICD/PTEN signaling axis, suggesting that targeting Plac1 or blocking the interaction of Plac1 and Furin could be a novel strategy to inhibit the Furin/NICD/PTEN signaling pathway. However, Plac1 and Furin binding sites are not clear and need to be further elucidated to provide intervention sites. Indeed, in highly invasive/metastatic MDA‐MB‐231 cells, Plac1 is expressed at lower levels than in MCF‐7 cells, but the physical interaction of Plac1 with Furin in MDA‐MB‐231 cells is more abundant than in MCF‐7 cells (Fig. 3D). This further demonstrates that Plac1 promotes invasion and metastasis of breast cancer cells through interaction with Furin. Metastasis of breast cancer is fatal, and currently, it is difficult to benefit from clinical treatment. Because of the restrictive expression and immunogenicity of Plac1, using it as an immunotherapy target could improve the prognosis of patients with breast cancer. Our team is studying the monoclonal antibody against Plac1 to determine its potential to inhibit tumor metastasis. More importantly, we are trying to use CRISPR/Cas9 technology to modify the expression fragments of Plac1, which express the T‐cell‐specific recognition fragments to enhance the sensitivity of immune recognition in our future research. Those studies will further elucidate the clinical value of Plac1.

## 5 Conclusion

In conclusion, this study describes the expression and clinical significance of Plac1 and explores the mechanism of action of Plac1 in breast cancer and demonstrates that the expression of Plac1 is a promising prognostic marker and potential therapeutic target and could be useful to reduce breast cancer metastases in future clinical applications by interfering with Plac1 or its downstream targets.

## Author contributions

YL, JC, and YY conceptualized and designed the experiments. YY provided funding support. YL, JC, JL, WF, FY, YW, YZ, CS, and MY performed all experimental procedures related to the study. ZF and YH provided technical support. YL prepared the manuscript. SNV, YY, and YH edited it. All authors contributed to the data analysis during discussions at joint meetings.

## Accession numbers

The gene microarray data in this paper are deposited to GEO (http://www.ncbi.nlm.nih.gov/geo/) with accession number: http://www.ncbi.nlm.nih.gov/geo/query/acc.cgi?acc=GSE104070.

## Supporting information


**Fig. S1.** Detection of Plac1 expression in various breast cancer cells and overexpression or knockdown of Plac1 in MDA‐MB‐231 and MCF‐7 cells.

**Fig. S2.** Effects of DEGs on cell biological behavior in Plac1‐overexpressing MDA‐MB‐231 cells vs. control cells.

**Table S1.** RNAi of Plac1 and Furin, primers and Plac1 plasmid, sequences used in the article.

**Appendix S1**. The proteins bound to Plac1 analysed by TripleTOF^®^ 5600+ LC/MS/MS system.

**Appendix S2**. The DEGs detected by Agilent DNA Microarray Scanner from MDA‐MB‐231‐Plac1 cells and MDA‐MB‐231‐NC cells.
