# Supplementary material for: Cancer/testis antigen‐Plac1 promotes invasion and metastasis of breast cancer through Furin/NICD/PTEN signaling pathway
Source: Mol Oncol. 2018 Jun 14;12(8):1233–48. doi: 10.1002/1878-0261.12311 (PMC6068355; doi:10.1002/1878-0261.12311)
Supplement: Supplementary file 1 — Fig. S1. Detection of Plac1 expression in various breast cancer cells and overexpression or knockdown of Plac1 in MDA‐MB‐231 and MCF‐7 cells. [file MOL2-12-1233-s001.pdf]

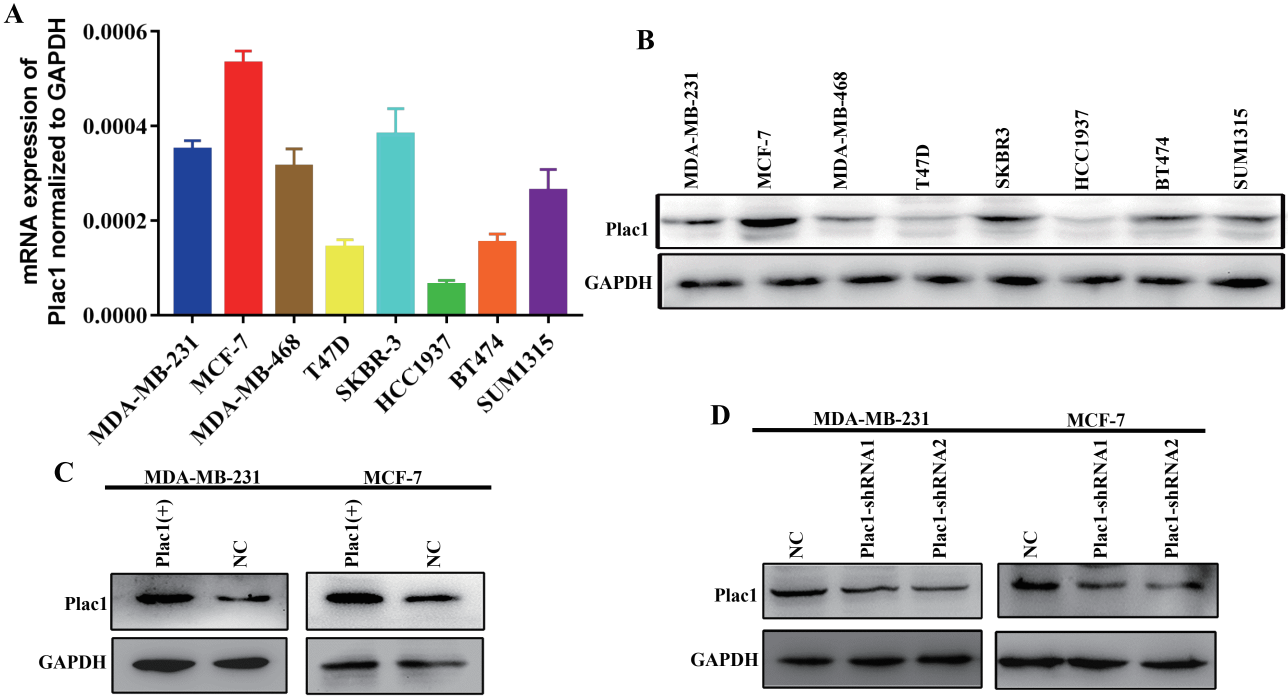

Supplemental Figure S1. Detection of Plac1 expression in various breast cancer cells and overexpression or knockdown of Plac1 in MDA-MB-231 and MCF-7 cells. **A** and **B**, qRT-PCR and Western blot analysis of Plac1 expression in various cell lines as indicated. **C** and **D**, Overexpression and knockdown of Plac1 were confirmed by western blot analysis in MDA-MB-231 and MCF-7 cells infected with the lentivirus of Plac1 plasmid, Plac1 shRNA1 or Plac1 shRNA2.
