# Supplementary material for: Cancer/testis antigen‐Plac1 promotes invasion and metastasis of breast cancer through Furin/NICD/PTEN signaling pathway
Source: Mol Oncol. 2018 Jun 14;12(8):1233–48. doi: 10.1002/1878-0261.12311 (PMC6068355; doi:10.1002/1878-0261.12311)
Supplement: Supplementary file 2 — Fig. S2. Effects of DEGs on cell biological behavior in Plac1‐overexpressing MDA‐MB‐231 cells vs. control cells. [file MOL2-12-1233-s002.pdf]

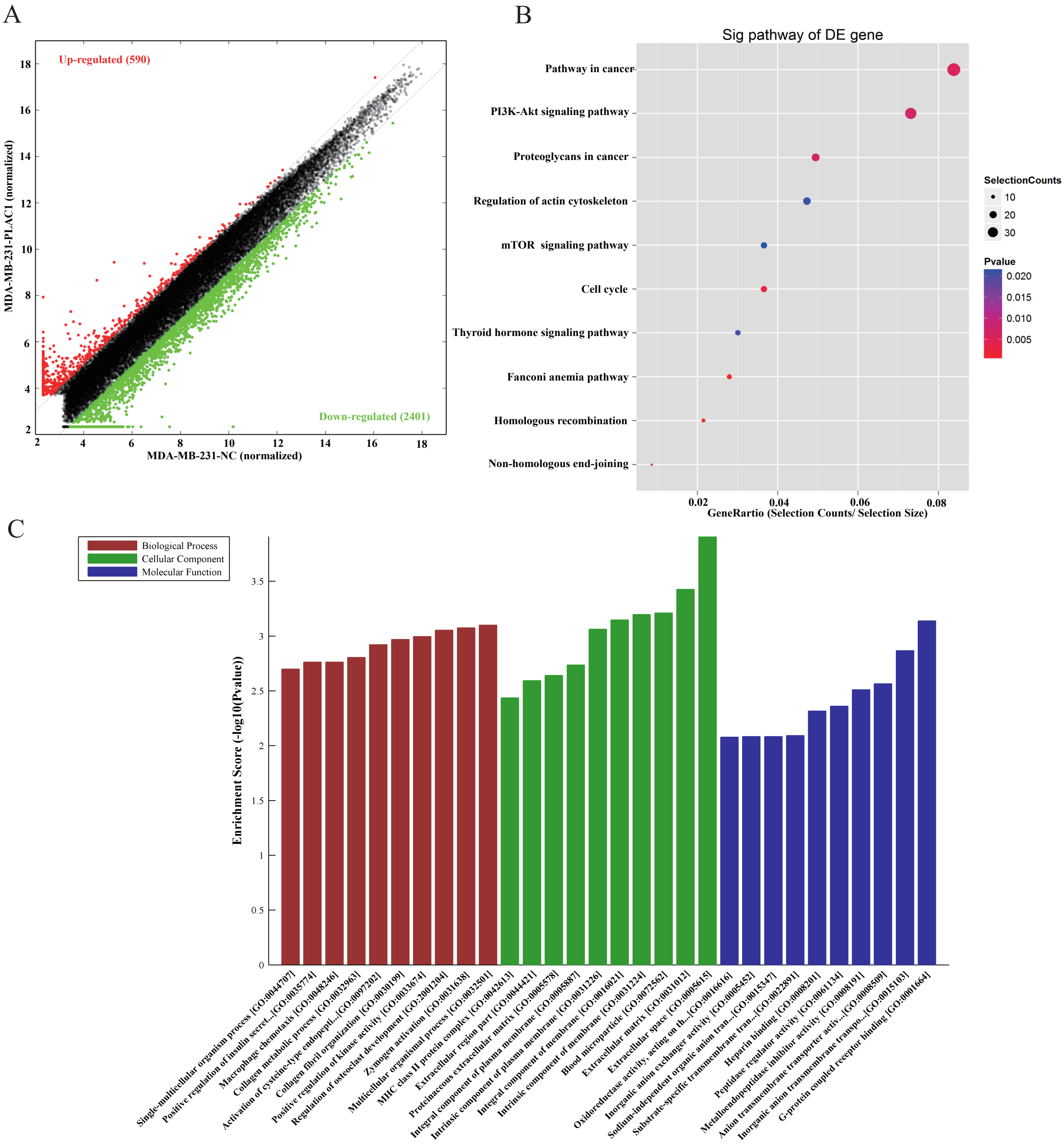

Supplemental Figure S2. Effects of differentially expressed genes on cell biological behavior in Plac1 overexpressing MDA-MB-231 cells vs. control cells. **A**, Volcano plot of differentially expressed genes in Plac1 overexpressing MDA-MB-231 cells vs. control cells. **B** and **C**, GO analysis showed that differentially expressed genes are enriched in tumor-associated signaling pathways.
