# Supplementary material for: Cancer/testis antigen‐Plac1 promotes invasion and metastasis of breast cancer through Furin/NICD/PTEN signaling pathway
Source: Mol Oncol. 2018 Jun 14;12(8):1233–48. doi: 10.1002/1878-0261.12311 (PMC6068355; doi:10.1002/1878-0261.12311)
Supplement: Supplementary file 3 — Table S1. RNAi of Plac1 and Furin, primers and Plac1 plasmid, sequences used in the article. [file MOL2-12-1233-s003.pdf]

| Seqname | Sense（5'-3'） | Anti-sense（5'-3'） |
|---|---|---|
| **Furin-siRNA** | GGACUUGGCAGGCAAUUAUTT | AUAAUUGCCUGCCAAGUCCTT |
| **Plac1-primers** | Forward: AGTTCACCTACCGTGTTACTGA | Reverse: AGTTCACCTACCGTGTTACTGA |
| **Plac1-shRNA1** | GGTTCAGGACAAAGTCCAATG | |
| **Plac1-shRNA2** | GCTACGAGGTGTTCAGCTTGT | |
| **Plac1-plasmid** | LV13(EF-1aF/Luciferase05&Puro) | ATGA AAGTTTTTAA GTTCATAGGA CTGATGATCC TCCTCACCTC TGCGTTTTCA GCCGGTTCAG GACAAAGTCC AATGACTGTG CTGTGCTCCA TAGACTGGTT CATGGTCACA GTGCACCCCT TCATGCTAAA CAACGATGTG TGTGTACACT TTCATGAACT ACACTTGGGC CTGGGTTGCC CCCCAAACCA TGTTCAGCCA CACGCCTACC AGTTCACCTA CCGTGTTACT GAATGTGGCA TCAGGGCCAA AGCTGTCTCT CAGGACATGG TTATCTACAG CACTGAGATA CACTACTCTT CTAAGGGCAC GCCATCTAAG TTTGTGATCC CAGTGTCATG TGCTGCCCCC CAAAAGTCCC CATGGCTCAC CAAGCCCTGC TCCATGAGAG TAGCCAGCAA GAGCAGGGCC ACAGCCCAGA AGGATGAGAA ATGCTACGAG GTGTTCAGCT TGTCACAGTC CAGTCAAAGG CCCAACTGCG ATTGTCCACC TTGTGTCTTC AGTGAAGAAG AGCATACCCA GGTCCCTTGT CACCAAGCAG GGGCTCAGGA GGCTCAACCT CTGCAGCCAT CTCACTTTCT TGATATTTCT GAGGATTGGT CTCTTCACAC AGATGATATG ATTGGGTCCA TGTGA |

**Supplemental Table S1.** RNAi of Plac1 and Furin, primers and Plac1 plasmid, sequences used in the article.
