# Supplementary material for: Cancer/testis antigen‐Plac1 promotes invasion and metastasis of breast cancer through Furin/NICD/PTEN signaling pathway
Source: Mol Oncol. 2018 Jun 14;12(8):1233–48. doi: 10.1002/1878-0261.12311 (PMC6068355; doi:10.1002/1878-0261.12311)
Supplement: Supplementary file 4 — Appendix S1. The proteins bound to Plac1 analysed by TripleTOF® 5600+ LC/MS/MS system. [file MOL2-12-1233-s004.pdf]

Supplemental Material S1. The proteins bound to Plac1 analysed by TripleTOF® 5600+ LC/MS/MS system. Total protein from MCF-7 cells were immunoprecipitated with anti-Plac1 antibody or IgG control antibody.

Gene nam	Description	First protein	Peptides
BAG2	BAG family molecular chaperone regulator 2	O95816	2
BANF1	Barrier-to-autointegration factor	O75531	2
C4B	Complement C4-B	F5GXS0	2
CALD1	Caldesmon	E9PGZ1	3
CALM2	Calmodulin (Fragment)	H0Y7A7	6
CAPZA1	F-actin-capping protein subunit alpha-1	P52907	3
CAPZB	Isoform 2 of F-actin-capping protein subunit beta	P47756-2	3
CLTC	Clathrin heavy chain	A0A087WVQ6	3
DBN1	Drebrin (Fragment)	D6RFI1	2
DDX3X	ATP-dependent RNA helicase DDX3X	A0A0D9SFB3	2
DES	Desmin	P17661	2
DKFZp686	Epididymis luminal protein 189	Q5HYB6	9
EEF1A1	Elongation factor 1-alpha 1	A0A087WVQ9	2
FURIN	**Furin**	P09958	6
FUS	RNA-binding protein FUS	H3BPE7	4
hCG_2039	Histone H2A	A0A0U1RR32	2
HIST1H1C	Histone H1.2	P16403	3
HMGA1	High mobility group protein HMG-I/HMG-Y	P17096	5
HMGA1	Isoform HMG-Y of High mobility group protein HMG-I/HM	P17096-2	4
HNRNPA1	Heterogeneous nuclear ribonucleoprotein A1	F8W6I7	8
HSPA8	Heat shock cognate 71 kDa protein	E9PKE3	7
KIF11	Kinesin-like protein KIF11	P52732	2
LIMA1	Isoform 4 of LIM domain and actin-binding protein 1	Q9UHB6-4	13
LIMCH1	LIM and calponin homology domains-containing protein	E7EPK0	2
LMNA	Prelamin-A/C	Q3BDU5	2
LMO7	LIM domain only protein 7 (Fragment)	E9PMP7	5
LRRFIP2	Isoform 2 of Leucine-rich repeat flightless-interacti	Q9Y608-2	3
MXRA7	HBV PreS1-transactivated protein 1	Q6ZR64	2
MYH10	Myosin-10	P35580	27
NCL	Nucleolin (Fragment)	H7BY16	2
NONO	Non-POU domain-containing octamer-binding protein (Fr	C9JYS8	2
PABPC1	Polyadenylate-binding protein	A0A087WTT1	5
PARP1	Poly [ADP-ribose] polymerase 1	P09874	8
PLAC1	Placenta-specific protein 1	Q9HBJ0	2
PLEC	Isoform 7 of Plectin	Q15149-7	3
PTRF	Polymerase I and transcript release factor	Q6NZI2	2
RPLP2	60S acidic ribosomal protein P2	P05387	4
RPS29	40S ribosomal protein S29	P62273	2
SHMT1	Isoform 4 of Serine hydroxymethyltransferase, cytosol	P34896-4	2
SPTAN1	Spectrin alpha chain, non-erythrocytic 1	A0A0D9SF54	4
SRSF1	Serine/arginine-rich-splicing factor 1	J3KTL2	5
SSBP1	Single-stranded DNA-binding protein, mitochondrial	Q04837	3
SUB1	Activated RNA polymerase II transcriptional coactivat	P53999	5
TAF15	TATA-binding protein-associated factor 2N	A0A075B7D9	3
TMOD3	Tropomodulin-3	Q9NYL9	7
TPM1	Tropomyosin alpha-1 chain (Fragment)	H0YL52	6
TPM2	Tropomyosin beta chain	Q5TCU3	11
TPM2	Tropomyosin beta chain	P07951	10
TPM4	Tropomyosin alpha-4 chain	P67936	11
VIM	Vimentin	B0YJC4	4
YBX1	Nuclease-sensitive element-binding protein 1 (Fragmen	H0Y449	4

Unique peptides	Sequence	Unique s	Mol. weig	Q-value	iBAQ ip	iBAQ igg
2	13.3	13.3	23.772	0	521360	0
2	40.4	40.4	10.058	0	7539800	0
2	1.4	1.4	187.67	0	29487	0
3	7.5	7.5	61.705	0	59595	0
6	42.2	42.2	20.762	0	31494000	0
3	15	15	32.922	0	716040	0
3	12.9	12.9	30.628	0	213740	0
3	2.2	2.2	192.06	0	9223.4	0
2	21.8	21.8	13.437	0	322290	0
2	3.4	3.4	70.839	0	14477	0
1	4.5	2.8	53.535	0.018957	189530	0
6	38.4	29.7	27.175	0	1259900	0
2	4.3	4.3	47.883	0	119100	0
6	6.4	6.4	86.677	0	375210	0
2	9.3	7.6	53.496	0	1381500	0
2	16.6	16.6	18.481	0	278910	0
3	15.5	15.5	21.364	0	1290800	0
2	41.1	23.4	11.676	0	25772000	0
1	34.4	14.6	10.679	0	5387000	0
8	37.1	37.1	33.155	0	1671000	0
7	14.2	14.2	68.805	0	178000	0
2	1.9	1.9	119.16	0	0	8808.3
13	24.7	24.7	85.223	0	785610	0
2	2.3	2.3	101.8	0	49489	0
2	4.1	4.1	55.637	0	22631	0
5	6	6	119.42	0	157710	0
3	10.2	10.2	45.413	0	338310	0
2	10.3	10.3	23.412	0	49990	2048.2
14	16.9	10.9	229	0	197040	0
2	11.5	11.5	32.389	0	68943	0
2	8.5	8.5	29.508	0	44435	0
5	10.7	10.7	58.535	0	211350	0
8	10.4	10.4	113.08	0	335460	0
2	16.5	16.5	23.616	0	700630	0
3	1.5	1.5	512.6	0	3960.2	0
2	7.4	7.4	43.476	0	175420	0
4	53	53	11.665	0	1635200	217060
2	32.1	32.1	6.6767	0	4340200	0
2	7.2	7.2	37.672	0	66314	0
4	1.8	1.8	282.83	0	8278	0
5	21.3	21.3	28.329	0	529580	0
3	32.4	32.4	17.259	0	565120	0
5	42.5	42.5	14.395	0	5766200	0
1	12.7	7.6	48.839	0	41604	0
7	32.1	32.1	39.594	0	869100	0
2	17	7.5	30.38	0	178600	0
2	37.7	11.6	32.814	0	418330	0
1	31.7	5.6	32.85	0	19070	0
4	40.7	14.9	28.521	0	739140	0
3	9	7.2	49.653	0	257140	0
4	23.8	23.8	41.905	0	659670	0
