# Supplementary material for: Cancer/testis antigen‐Plac1 promotes invasion and metastasis of breast cancer through Furin/NICD/PTEN signaling pathway
Source: Mol Oncol. 2018 Jun 14;12(8):1233–48. doi: 10.1002/1878-0261.12311 (PMC6068355; doi:10.1002/1878-0261.12311)
Supplement: Supplementary file 5 — Appendix S2. The DEGs detected by Agilent DNA Microarray Scanner from MDA‐MB‐231‐Plac1 cells and MDA‐MB‐231‐NC cells. [file MOL2-12-1233-s005.pdf]

Supplemental Material S2. The differentially expressed genes detected by Agilent DNA Microarray Scanner from MDA-MB-231-Plac1cells and MDA-MB-231-NC cells.

Fold Change cut-off: **2.0**
Condition pairs: **231plac1_plus_vs_231plac1_NC**

Column A: ProbeName it represents probe name.
Column B: Fold change, Absolute Fold change between two samples.
Column C: Regulation, it depicts which sample has greater or lower intensity values wrt other sample.
Column D ~ K: Annotations to each probe, including GenbankAccession, GenomicCoordinates, GeneSymbol, Description, Go,
Column L ~ M: Raw Intensity of each sample.
Column N ~ O: Normalized Intensity of each sample (log2 transformed).

2

	Fold change and Regulation				
**ProbeName**	**Fold Change**	**Regulation**	**GenbankAccession**	**GenomicCoordinate**	**GeneSymbol**
A_23_P2683	3.0667743	down	NM_024604		RPAP3
A_33_P3367647	2.0342655	down		chr6:139134033-139134092	
A_32_P85539	2.0636507	down	NM_013320	chr12:104500200-10	HCFC2
A_24_P28657	4.058082	down	NM_015446	chr1:247003155-247	AHCTF1
A_23_P112846	2.4644821	down	NM_001144978	chr4:075168172-075	MTHFD2L
A_23_P163113	2.2660575	down	NM_017922	chr14:045584252-04	PRPF39
A_23_P9280	2.213369	down	NR_029410	chr9:086504177-086	LOC389765
A_23_P104146	2.0147377	down	NM_005095	chr1:035887010-035	ZMYM4
A_23_P120048	4.8778967	down	NM_013450	chr2:160175985-160	BAZ2B
A_23_P253524	4.3118112	down	NM_001813	chr4:104027398-104	CENPE
A_33_P3231878	3.9463899	down	NM_002645	chr11:017108358-01	PIK3C2A
A_23_P355623	2.25699	down	NM_019004	chr7:092029770-092	ANKIB1
A_23_P120442	2.0392779	down	NM_181659	chr20:046283630-04	NCOA3
A_23_P115645	2.1882476	down	NM_001025076	chr10:011367869-01	CUGBP2
A_24_P191664	2.0957425	down	NM_014498	chr3:167727993-167	GOLIM4
A_23_P105862	2.0971179	down	NM_023037	chr13:032870558-03	FRY
A_23_P115167	2.1012377	down	NM_015350	chr1:090058368-090	LRRC8B
A_23_P388812	2.5729689	down	NM_152515	chr2:113495817-113	CKAP2L
A_23_P133284	2.0594827	down	NM_018343	chr5:096498802-096	RIOK2
A_23_P122068	2.2947577	down	NM_181435	chr5:034020663-034	C1QTNF3
A_33_P3215239	3.249763	down	NM_016195	chr10:091522529-09	KIF20B
A_23_P107724	2.4551038	down	NM_013380	chr19:044830983-04	ZFP112
A_23_P139509	2.7125355	down	NM_016570		ERGIC2
A_33_P3305655	2.8185327	down	NR_027992	chr15:020874916-02	BCL8
A_33_P3259092	2.4854685	down	NM_001079802	chr9:108399016-108	FKTN
A_23_P122624	2.3106744	down	NM_032860	chr6:144184627-144	LTV1
A_23_P83976	4.0453962	down	NM_145036	chr17:063685309-06	CCDC46
A_23_P214139	4.4894198	down	NM_002912	chr6:111620573-111	REV3L
A_23_P110569	2.1840025	down	NM_018700	chr5:114460760-114	TRIM36
A_33_P3415321	2.2940693	down	NR_002329	chr7:116596709-116	ST7OT4
A_24_P940921	4.0567826	down	NM_015046	chr9:135137022-135	SETX
A_23_P252748	3.0832674	down	NM_007152	chr11:003380673-00	ZNF195
A_23_P120710	5.1895988	down	NM_003316	chr21:038574855-03	TTC3
A_23_P12363	2.1132406	down	NM_005012	chr1:064644540-064	ROR1
A_32_P74955	2.8958543	down	NM_152641	chr12:046301413-04	ARID2
A_23_P117225	2.150157	down	NM_000123	chr13:103527950-10	ERCC5
A_23_P209288	2.1801362	down	NM_003590	chr2:225335155-225	CUL3
A_33_P3782469	2.3228339	down	AL833463	chr15:55831224-558	PYGO1
A_23_P121545	2.7102206	down	NM_201591	chr4:176554554-176	GPM6A
A_23_P111929	2.5167835	down	NM_016023	chr8:092090690-092	OTUD6B
A_23_P105276	2.4560859	down	NM_003428	chr12:133635754-13	ZNF84
A_24_P177353	4.0834048	down	NM_001081550	chrX:122734545-12	THOC2
A_32_P10396	2.224375	down	NM_014991	chr4:085591282-085	WDFY3
A_32_P108826	2.6106186	down	NM_194314	chr1:197123395-197	ZBTB41
A_23_P59988	2.2370474	down	NM_054028	chr8:011189505-011	AMAC1L2
A_23_P148969	2.2737931	down	NM_017768	chr1:070610619-070	LRRC40
A_23_P100883	2.2417727	down	NM_015355	chr17:030327536-03	SUZ12

RefSeqAccession, UniGeneID and EntrezGeneID.

:31plac1_plus_vs_231plac1_NC 2.0 fold down regulated genes					
Annotations					Raw In
Description	Go	RefSeqAccession	UniGeneID	EntrezGeneID	231plac1_plus](raw
Homo sapiens RNA ן	GO:0005488	NM_024604	Hs.437855	79657	395.03073
Putative guanine nucleotide exchange factor LFDH (Lung-specific F-box and DH domain-containing protei					7.246642
Homo sapiens host c	GO:0005737|GO:00(	NM_013320	Hs.506558	29915	132.96454
Homo sapiens AT ho	GO:0000910|GO:00(	NM_015446	Hs.300887	25909	519.8579
Homo sapiens methy	GO:0000287|GO:00(	NM_001144978	Hs.479954	441024	17.108208
Homo sapiens PRP3	GO:0008380|GO:00(	NM_017922	Hs.274337	55015	135.1116
Homo sapiens kinesin family member 27 p		NR_029410	Hs.601256	389765	208.79262
Homo sapiens zinc fi	GO:0008270|GO:00(	NM_005095	Hs.269211	9202	364.78543
Homo sapiens bromo	GO:0005515|GO:00(	NM_013450	Hs.470369	29994	21.633797
Homo sapiens centro	GO:0005515|GO:00(	NM_001813	Hs.75573	1062	172.11954
Homo sapiens phospl	GO:0005515|GO:00(	NM_002645	Hs.175343	5286	548.2953
Homo sapiens ankyri	GO:0005515|GO:00(	NM_019004	Hs.83293	54467	145.761
Homo sapiens nuclea	GO:0005794|GO:00:	NM_181659	Hs.592142	8202	27.739933
Homo sapiens CUG t	GO:0006397|GO:00(	NM_001025076	Hs.309288	10659	62.894444
Homo sapiens golgi i	GO:0005801|GO:00(	NM_014498	Hs.143600	27333	356.7051
Homo sapiens furry h	GO:0016020|GO:001	NM_023037	Hs.507669	10129	19.675072
Homo sapiens leucin	GO:0005515|GO:001	NM_015350	Hs.482017	23507	43.82738
Homo sapiens cytoskeleton associated prot		NM_152515	Hs.434250	150468	211.29964
Homo sapiens RIO k	GO:0000166|GO:001	NM_018343	Hs.27021	55781	781.97675
Homo sapiens C1q a	GO:0005576	NM_181435	Hs.171929	114899	5.54072
Homo sapiens kinesi	GO:0007088|GO:00(	NM_016195	Hs.240	9585	178.01486
Homo sapiens zinc fi	GO:0005622|GO:00(	NM_013380	Hs.48589	7771	61.68931
Homo sapiens ERGI	GO:0005737|GO:00(	NM_016570	Hs.339453	51290	347.80936
Homo sapiens B-cell CLL/lymphoma 8 (B		NR_027992	Hs.657985	606	4.9999995
Homo sapiens fukuti	GO:0007517|GO:00(	NM_001079802	Hs.55777	2218	9.423765
Homo sapiens LTV1 homolog (S. cerevisia		NM_032860	Hs.185675	84946	1513.488
Homo sapiens coiled-coil domain containi		NM_145036	Hs.408676	201134	32.92834
Homo sapiens REV3	GO:0006261|GO:00(	NM_002912	Hs.232021	5980	222.15688
Homo sapiens tripart	GO:0005622|GO:00(	NM_018700	Hs.519514	55521	158.86568
Homo sapiens ST7 overlapping transcript 4		NR_002329	Hs.662742	338069	28.228085
Homo sapiens senata	GO:0006396|GO:00(	NM_015046	Hs.460317	23064	319.0031
Homo sapiens zinc fi	GO:0005622|GO:00(	NM_007152	Hs.386294	7748	641.5804
Homo sapiens tetratri	GO:0005773|GO:00(	NM_003316	Hs.368214	7267	59.49819
Homo sapiens recept	GO:0005515|GO:00(	NM_005012	Hs.654491	4919	914.14545
Homo sapiens AT ric	GO:0005622|GO:00(	NM_152641	Hs.317304	196528	107.862915
Homo sapiens excisi	GO:0043066|GO:00(	NM_000123	Hs.258429	2073	161.6237
Homo sapiens cullin	GO:0031461|GO:00(	NM_003590	Hs.372286	8452	25.15792
Pygopus homolog 1	GO:0005515|GO:0001822|GO:0008270|G		Hs.87194	26108	58.12644
Homo sapiens glycop	GO:0005262|GO:001	NM_201591	Hs.75819	2823	5.430054
Homo sapiens OTU domain containing 6B		NM_016023	Hs.30532	51633	43.09047
Homo sapiens zinc fi	GO:0005622|GO:00(	NM_003428	Hs.445019	7637	232.94397
Homo sapiens THO c	GO:0008380|GO:00(	NM_001081550	Hs.149991	57187	161.38893
Homo sapiens WD re	GO:0005737|GO:001	NM_014991	Hs.480116	23001	167.2448
Homo sapiens zinc fi	GO:0005622|GO:00(	NM_194314	Hs.529439	360023	60.95053
Homo sapiens acyl-n	GO:0016020|GO:001	NM_054028	Hs.458397	83650	8.524523
Homo sapiens leucin	GO:0005515	NM_017768	Hs.147836	55631	682.3642
Homo sapiens suppre	GO:0008284|GO:001	NM_015355	Hs.462732	23512	1849.813

tensity	Normalized Intensity	
[231plac1_NC](raw)	plac1_plus](normali	plac1_NC](normalized)
1454.5942	8.77956	10.396282
18.167297	3.0298676	4.0543756
336.36365	7.1941032	8.239302
2484.9568	9.165159	11.185957
50.556656	4.232816	5.5341005
375.12424	7.213683	8.3938675
571.4134	7.863462	9.009706
902.2854	8.663973	9.674565
126.5906	4.5700693	6.8563285
900.62823	7.56417	9.672464
2548.542	9.2393265	11.21986
401.1802	7.318552	8.492952
68.003555	4.9367146	5.964773
164.47302	6.097311	7.227087
920.6427	8.6334505	9.700912
49.39381	4.4331627	5.5015707
110.16281	5.577241	6.6484804
668.27466	7.880367	9.243801
1907.827	9.753592	10.795874
15.997393	2.662663	3.8610048
700.61145	7.6161685	9.316503
180.9426	6.0710754	7.3668594
1144.1171	8.597405	10.037047
15.69335	2.3362613	3.8312056
28.830864	3.3969696	4.7104874
3935.3987	10.673107	11.881421
160.91562	5.175148	7.191429
1209.4258	7.953472	10.120001
424.40326	7.4449425	8.571917
78.05035	4.959185	6.157094
1559.6969	8.473551	10.493887
2328.685	9.468142	11.092602
375.0382	6.017661	8.393284
2244.7966	9.959303	11.03876
381.20892	6.881819	8.415808
425.2741	7.470545	8.574987
65.57488	4.7888374	5.9132557
161.67905	5.984275	7.200161
18.484173	2.6370707	4.075481
129.44644	5.5550194	6.8866005
700.3341	8.019423	9.315784
797.13666	7.4674444	9.497217
454.9166	7.522992	8.676392
190.262	6.0527306	7.4371223
23.401384	3.2522044	4.4138002
1835.9656	9.554437	10.739538
4621.3296	10.952082	12.116722

A_24_P671164	3.9965793	down	NM_000949	chr5:035056262-035	PRLR
A_23_P11237	2.3787743	down	NM_004606	chrX:070680545-07(	TAF1
A_23_P306890	2.2815053	down	NM_007195	chr18:051820933-05	POLI
A_23_P113462	3.1644439	down	NM_017641	chr12:039688210-03	KIF21A
A_23_P104054	2.9901441	down	NM_016227	chr1:172580756-172	C1orf9
A_23_P212002	5.4875436	down	NM_005385	chr3:042689920-042	NKTR
A_32_P106732	3.8231232	down	NM_020937	chr14:045669210-04	FANCM
A_24_P351466	2.4018206	down	NM_020890	chr3:108269504-108	KIAA1524
A_23_P136817	2.731573	down	NM_014708		KNTC1
A_24_P50818	3.955649	down		chr2:197645020-197644961	
A_24_P288779	2.5150359	down	NM_130775	chrX:052842182-052	XAGE5
A_24_P29277	2.9415325	down	NM_001130105	chr5:074667084-074	COL4A3BP
A_23_P213255	3.5613371	down	NM_020159	chr4:095211718-095	SMARCAD1
A_23_P204550	2.8651487	down	NM_017988	chr12:100733740-10	SCYL2
A_33_P3802146	3.4796423	down	AK131313	chr10:42755760-42755701	
A_23_P210131	2.0918712	down	XM_001717577	chr2:96657375-9665	LOC400987
A_33_P3242099	3.4710833	down	NM_203459	chr1:200822485-200	CAMSAP1L1
A_23_P50907	2.4921139	down	NM_002210	chr2:187545295-187	ITGAV
A_24_P53282	2.2253666	down	NM_001304	chr17:028795047-02	CPD
A_33_P3406245	2.1195471	down	NM_005681	chr1:222742915-222	TAF1A
A_23_P122615	3.9890439	down	NM_032870	chr6:099848620-099	SFRS18
A_33_P3315504	2.1609445	down	NM_153332	chr8:008890645-008	ERI1
A_33_P3362453	2.2701182	down		chrX:051665138-051665079	
A_33_P3348924	3.3920012	down	AK128128	chr16:33346963-333	LOC100128348
A_23_P206532	2.4213926	down	NM_001031835	chr16:047733400-04	PHKB
A_33_P3217238	2.7161026	down	NM_014109	chr8:124332837-124	ATAD2
A_23_P97584	3.6375675	down	NM_014597		DNTTIP2
A_23_P117157	2.3447254	down	NM_003850	chr13:048517447-04	SUCLA2
A_24_P175783	2.0918954	down	NM_015313	chr11:120359849-12	ARHGEF12
A_33_P3831566	3.1157403	down	AF072164	chr9:91769268-91769209	
A_23_P45087	4.0748106	down	NM_016220	chr7:064171177-064	ZNF107
A_23_P130429	4.010073	down	NM_005406	chr18:018535191-01	ROCK1
A_23_P372638	3.6525188	down	NM_025134		CHD9
A_23_P80902	3.3592193	down	NM_020242	chr3:044894386-044	KIF15
A_33_P3298661	2.8986294	down	NM_021964	chr3:124952272-124	ZNF148
A_23_P106241	4.1924527	down	NM_004239	chr14:092435951-09	TRIP11
A_33_P3258607	3.3648043	down	AK131322	chr9:26118336-26118395	
A_24_P940776	4.691304	down	NM_018429	chr5:070862873-070	BDP1
A_24_P941148	3.5903289	down	NM_017645	chr9:019053295-019	HAUS6
A_33_P3224426	2.2868211	down	AK125040	chr11:134605634-134605693	
A_24_P204214	2.1608217	down	NM_006311	chr17:016068436-01	NCOR1
A_33_P3244872	6.3250571	down	NM_014810	chr1:180053181-180	CEP350
A_33_P3246997	3.3885719	down	BC056409	chr17:48814662-48814721	
A_33_P3285047	2.1319506	down	NM_153028	chr16:003368494-00	ZNF75A
A_23_P115597	4.2448365	down	NM_014915	chr10:027302014-02	ANKRD26
A_33_P3365845	2.0528346	down		chr13:025912864-025912923	
A_24_P926760	2.3048039	down	NM_005385		NKTR
A_33_P3242713	3.3415827	down	AK022058	chr18:8015115-8015174	
A_23_P162874	2.9835046	down	NM_005348	chr14:102549512-10	HSP90AA1
A_32_P141612	2.0269853	down	NM_002552	chr2:148691827-148	ORC4L
A_24_P541919	2.5896911	down	NM_144973	chr12:031535725-03	DENND5B
A_23_P53267	2.1522117	down	NM_198261	chr12:122989804-12	RSRC2
A_33_P3405531	3.7689188	down	NM_007114	chr3:069069159-069	TMF1
A_23_P82941	3.4449723	down	NM_006421	chr8:068110253-068	ARFGEF1
A_33_P3308706	2.5044995	down	CR749502	chr11:11901729-11901788	
A_32_P167122	2.1236595	down	NM_018254	chr1:211489128-211	RCOR3
A_23_P17393	2.0333358	down	NM_001316	chr20:047712950-04	CSE1L
A_24_P109554	3.1944421	down	NM_007018		CEP110
A_23_P112950	2.176414	down	NM_018179	chr12:014650947-01	ATF7IP
A_23_P162734	3.4724382	down	NM_005977	chr13:026787364-02	RNF6

Homo sapiens prolac	GO:0042978|GO:00(	NM_000949	Hs.368587	5618	4.9999995
Homo sapiens TAF1	GO:0006368|GO:00:	NM_004606	Hs.158560	6872	14.314585
Homo sapiens polym	GO:0006260|GO:00(	NM_007195	Hs.438533	11201	186.90282
Homo sapiens kinesi	GO:0000166|GO:00(	NM_017641	Hs.374201	55605	25.385096
Homo sapiens chrom	GO:0016020|GO:001	NM_016227	Hs.204559	51430	1358.8224
Homo sapiens natura	GO:0016018|GO:001	NM_005385	Hs.529509	4820	604.9428
Homo sapiens Fanco	GO:0008026|GO:00(	NM_020937	Hs.509229	57697	197.87723
Homo sapiens KIAA	GO:0005515|GO:00(	NM_020890	Hs.591308	57650	25.342558
Homo sapiens kineto	GO:0005828|GO:00(	NM_014708	Hs.300559	9735	371.02185
General transcription factor 3C polypeptide 3 (Transcription factor IIIC subunit gamma)(TF3C-gamma)(TF					23.106659
Homo sapiens X antigen family, member 5		NM_130775	Hs.356874	170627	4.9999995
Homo sapiens collag	GO:0005515|GO:00:	NM_001130105	Hs.270437	10087	150.51701
Homo sapiens SWI/S	GO:0005515|GO:00(	NM_020159	Hs.410406	56916	377.48862
Homo sapiens SCY1	GO:0048471|GO:00(	NM_017988	Hs.506481	55681	1132.2222
Homo sapiens cDNA FLJ16301 fis, clone PLACE7000333. [AK			Hs.255729		1923.6248
UPF0634 protein C (Protein immuno-react		XM_001717577	Hs.646318	400987	193.34308
Homo sapiens calmo	GO:0005874	NM_203459	Hs.23585	23271	332.8585
Homo sapiens integri	GO:0005515|GO:00(	NM_002210	Hs.436873	3685	2820.4675
Homo sapiens carbo	GO:0005802|GO:00(	NM_001304	Hs.446079	1362	272.1866
Homo sapiens TATA	GO:0005515|GO:00(	NM_005681	Hs.153088	9015	430.90656
Homo sapiens splicir	GO:0016607|GO:00(	NM_032870	Hs.520287	25957	63.83395
Homo sapiens exorib	GO:0005622|GO:00(	NM_153332	Hs.20000	90459	1077.2236
					4.9999995
Homo sapiens cDNA FLJ46249 fis, clone TESTI4021377. [AK			Hs.637572	100128348	4.9999995
Homo sapiens phospl	GO:0005516|GO:00(	NM_001031835	Hs.78060	5257	381.17316
Homo sapiens ATPa	GO:0017111|GO:00(	NM_014109	Hs.370834	29028	2971.4788
Homo sapiens deoxy	GO:0005634|GO:004	NM_014597	Hs.85769	30836	1997.2543
Homo sapiens succin	GO:0005739|GO:00(	NM_003850	Hs.546323	8803	257.7219
Homo sapiens Rho gi	GO:0005515|GO:00(	NM_015313	Hs.24598	23365	32.988625
Homo sapiens HSFE-1 mRNA, partial cds. [AF072164]			Hs.667154		38.28145
Homo sapiens zinc fi	GO:0005622|GO:00(	NM_016220	Hs.50216	51427	117.60334
Homo sapiens Rho-a	GO:0007242|GO:00:	NM_005406	Hs.306307	6093	108.70749
Homo sapiens chrom	GO:0005515|GO:00(	NM_025134	Hs.59159	80205	37.1258
Homo sapiens kinesi	GO:0007067|GO:00(	NM_020242	Hs.658939	56992	330.90048
Homo sapiens zinc fi	GO:0016564|GO:00(	NM_021964	Hs.592591	7707	62.896107
Homo sapiens thyroi	GO:0005515|GO:00(	NM_004239	Hs.632339	9321	135.88736
Putative deoxyuridine 5'-triphosphate nucleotidohydrolase-like p			Hs.533221		5.579885
Homo sapiens B doul	GO:0005634|GO:00(	NM_018429	Hs.258272	55814	124.38368
Homo sapiens HAUS	GO:0051297|GO:00(	NM_017645	Hs.533468	54801	504.76343
Putative uncharacterized protein ENSP00000363872 [Source:U			Hs.148365		37.965054
Homo sapiens nuclea	GO:0005515|GO:00(	NM_006311	Hs.462323	9611	472.6887
Homo sapiens centro	GO:0005813|GO:00(	NM_014810	Hs.413045	9857	176.14014
Cisplatin resistance-associated overexpressed protein (cAMP re			Hs.130293		22.877306
Homo sapiens zinc fi	GO:0005622|GO:00(	NM_153028	Hs.513292	7627	679.1826
Homo sapiens ankyrin repeat domain 26 (/		NM_014915	Hs.361041	22852	26.606432
					7.321807
Homo sapiens natura	GO:0016018|GO:001	NM_005385	Hs.529509	4820	391.5072
Homo sapiens cDNA FLJ11996 fis, clone HEMBB1001449. [AK022058]					17.535131
Homo sapiens heat sl	GO:0006986|GO:004	NM_005348	Hs.525600	3320	15321.569
Homo sapiens origin	GO:0006260|GO:00(	NM_002552	Hs.558364	5000	58.2745
Homo sapiens DENN	GO:0016020|GO:001	NM_144973	Hs.118166	160518	203.02654
Homo sapiens arginine/serine-rich coiled-c		NM_198261	Hs.432996	65117	1330.343
Homo sapiens TATA	GO:0005515|GO:00(	NM_007114	Hs.267632	7110	103.48257
Homo sapiens ADP-1	GO:0005622|GO:00(	NM_006421	Hs.656902	10565	129.49287
Ubiquitin carboxyl-terminal hydrolase 47 (EC 3.1.2.15)(Ubiquit			Hs.577256		24.253267
Homo sapiens REST	GO:0005515|GO:00(	NM_018254	Hs.356399	55758	113.47589
Homo sapiens CSE1	GO:0005515|GO:00(	NM_001316	Hs.90073	1434	10934.433
Homo sapiens centro	GO:0005515|GO:00(	NM_007018	Hs.653263	11064	43.96334
Homo sapiens activa	GO:0006306|GO:00(	NM_018179	Hs.714407	55729	155.74216
Homo sapiens ring fi	GO:0005515|GO:00(	NM_005977	Hs.136885	6049	809.29

22.104586	2.3362613	4.335027
40.918396	3.9707813	5.2209997
521.77313	7.691003	8.880989
97.10603	4.801321	6.463273
4590.6846	10.520697	12.100912
3814.3225	9.3805065	11.836667
926.1693	7.7752113	9.709963
72.85165	4.7989836	6.063112
1226.2646	8.688317	10.138049
110.383575	4.667592	6.6515064
14.031347	2.3362613	3.6668403
538.8436	7.366293	8.922861
1618.182	8.714933	10.547352
3671.4773	10.262608	11.781218
7327.3223	11.0068	12.805739
496.9812	7.742322	8.807116
1395.0833	8.536624	10.33201
7608.2134	11.542147	12.859517
742.082	8.241995	9.396038
1102.0801	8.896761	9.980517
310.00873	6.122815	8.118858
2686.5085	10.190075	11.301737
12.767329	2.3362613	3.5190287
18.804058	2.3362613	4.098398
1118.3102	8.729683	10.00552
8668.031	11.616148	13.057686
7930.361	11.05546	12.918434
741.5318	8.165993	9.395412
82.81209	5.1770287	6.2418394
142.8965	5.3901463	7.0297213
580.5741	7.005234	9.031967
528.474	6.8957205	8.899349
163.2703	5.3469543	7.215846
1344.9592	8.528551	10.276677
218.90671	6.0976915	7.6330624
687.58307	7.221494	9.2892885
23.543894	2.6726227	4.4231453
703.7934	7.093567	9.323556
2146.542	9.124516	10.968632
104.66541	5.3786325	6.571976
1231.6426	9.033817	10.145397
1328.3192	7.5975275	10.258606
93.53206	4.651205	6.4118824
1723.7185	9.547728	10.639902
136.11661	4.873019	6.958728
18.58763	3.0447352	4.0823526
1092.902	8.765666	9.97031
70.1419	4.26836	6.0088916
46023.855	13.909517	15.486525
140.45341	5.987548	7.0068836
645.0287	7.817288	9.190068
3275.0525	10.490448	11.596268
475.0339	6.8225203	8.736671
541.60156	7.1464696	8.930962
72.90047	4.739505	6.0640273
292.94455	6.9543695	8.040922
22022.055	13.42482	14.4486685
167.87415	5.582011	7.257575
414.30313	7.415263	8.537216
3280.8599	9.803053	11.599002

A_33_P3303146	2.0122939	down	NM_032852	chr1:063329990-063	ATG4C
A_23_P325501	2.063395	down	NM_015358	chr21:037748528-03	MORC3
A_33_P3334590	2.1058627	down	NM_017762	chr15:031260183-03	MTMR10
A_33_P3349334	2.8244628	down	NM_199421	chr14:055516046-05	SOCS4
A_24_P316305	3.3729735	down	EF553519	chr15:35147853-351	AQR
A_32_P233938	2.0408934	down	NM_001040057	chr5:060671789-060	FAM133B
A_24_P248606	3.6806588	down	NM_004457	chr2:223807495-223	ACSL3
A_23_P70213	2.9040178	down	NM_000038	chr5:112180822-112	APC
A_23_P117387	2.3832553	down	NM_054024	chr14:039716981-03	MIA2
A_33_P3403831	2.2472553	down		chr9:136184460-136184519	
A_33_P3349414	2.028191	down	NM_014007	chr9:129597442-129	ZBTB43
A_24_P265856	4.1501869	down	NM_020654	chr3:101043356-101	SENP7
A_23_P320897	3.0308511	down	AK024832	chr1:197479387-197479328	
A_33_P3349546	2.1008734	down	NM_025000	chr2:172341501-172	DCAF17
A_32_P397824	2.2589861	down	NM_018086	chr2:164464661-164	FIGN
A_23_P500861	2.440899	down	NM_182961	chr6:152443111-152	SYNE1
A_23_P435657	3.1694761	down	NR_003683	chr2:073899939-073	ALMS1P
A_24_P133253	3.0644758	down	NM_000899	chr12:088886712-08	KITLG
A_33_P3405168	2.1144765	down	NM_172250	chr4:146579784-146	MMAA
A_33_P3349496	3.9020014	down	XM_001725589	chr12:094671645-09	LOC100130413
A_33_P3211054	2.1341839	down	NM_152373	chr1:041007295-041	ZNF684
A_23_P100127	5.4903343	down	NM_170589	chr15:040917525-04	CASC5
A_33_P3336632	2.5074032	down	NR_003287	chrUn_gl000220:00(	LOC100008589
A_23_P357860	2.384645	down	NM_003262	chr3:169715114-169	SEC62
A_23_P209962	4.3409747	down	NM_024624		SMC6
A_33_P3407195	6.2549355	down	NM_148894	chr4:013612618-013	BOD1L
A_24_P400997	2.5984711	down	NM_015295		SMCHD1
A_33_P3223467	2.1751351	down	NM_025054	chr8:067542631-067	VCPIP1
A_23_P40059	3.0270049	down	NM_000534		PMS1
A_23_P367628	2.21734	down	NM_014802	chr12:022601833-02	KIAA0528
A_24_P395317	6.8256011	down	NM_014953	chr13:073330377-07	DIS3
A_33_P3231602	2.8700298	down	NM_152484	chr19:037902622-03	ZNF569
A_23_P301336	3.4226574	down	NM_014472	chr10:100004055-10	C10orf28
A_33_P3344204	2.1879922	down	NM_024786	chr5:000795933-000	ZDHHC11
A_33_P3259960	2.4231278	down	AK125579	chr7:55505936-5550	LOC100128019
A_23_P153855	3.5020629	down	NM_020855	chr19:022849435-02	ZNF492
A_33_P3302300	2.4584312	down	AK127681	chr2:70237008-7023	LOC100128333
A_24_P56388	2.393789	down	NM_181054	chr14:062214845-06	HIF1A
A_23_P312174	3.943244	down	NM_015120	chr2:073836752-073	ALMS1
A_32_P76035	2.3205124	down	NM_033109	chr2:055862315-055	PNPT1
A_23_P200801	2.1936904	down	NM_001002811	chr1:144890739-144	PDE4DIP
A_23_P94762	2.3570641	down	NM_058230	chr5:178311237-178	ZNF354B
A_23_P64888	2.7233054	down	NM_023921	chr12:010978070-01	TAS2R10
A_24_P166126	2.1460683	down	NM_016599	chr4:120108443-120	MYOZ2
A_33_P3423425	2.6747592	down	NM_014106	chr15:035270627-03	ZNF770
A_23_P129466	2.0131128	down	NM_024997	chr16:010577266-01	ATF7IP2
A_23_P146417	2.315815	down	NM_032012	chr9:111777792-111	C9orf5
A_32_P153725	3.2494375	down	NM_015275	chr12:105562796-10	KIAA1033
A_23_P303810	3.8084818	down	NM_153605	chr3:097663710-097	CRYBG3
A_32_P157945	2.6243352	down	NM_004415	chr6:007586582-007	DSP
A_23_P124438	2.5719463	down	NM_001039127	chr4:000156150-000	ZNF718
A_33_P3218694	4.4409551	down	NM_002687	chr14:039652268-03	PNN
A_33_P3277407	2.1141778	down	NM_182931	chr7:104751253-104	MLL5
A_33_P3212640	3.423046	down	NM_203458	chr1:145285853-145	NOTCH2NL
A_33_P3249731	2.0209734	down	XM_002344316	chr7:051454308-051	LOC100294070
A_33_P3577671	3.2635361	down	NM_130434	chr15:065739034-06	DPP8
A_23_P11664	2.2946307	down	NM_004768	chr1:070716494-070	SFRS11
A_23_P396353	4.1286411	down	NM_020921	chr14:051187234-05	NIN
A_33_P3288384	2.4447603	down	NM_001143937	chr11:014535571-01	PSMA1
A_33_P3377888	2.3111612	down	AK123255	chr11:113690256-113690197	

Homo sapiens ATG4	GO:0008017|GO:000	NM_032852	Hs.7353	84938	84.67795
Homo sapiens MOR(	GO:0018105|GO:005	NM_015358	Hs.421150	23515	288.86023
Homo sapiens myotu	GO:0016791|GO:001	NM_017762	Hs.30141	54893	12.1692095
Homo sapiens suppre	GO:0005515|GO:000	NM_199421	Hs.532610	122809	933.3429
Intron-binding protei	GO:0008380|GO:0006397|GO:0003723|G		Hs.510958	9716	328.58063
Homo sapiens family with sequence simila		NM_001040057	Hs.489105	257415	107.332985
Homo sapiens acyl-C	GO:0005515|GO:000	NM_004457	Hs.655772	2181	410.20535
Homo sapiens adeno	GO:0008017|GO:000	NM_000038	Hs.158932	324	20.630226
Homo sapiens melan	GO:0005576	NM_054024	Hs.287694	117153	15.978937
					4.9999995
Homo sapiens zinc fi	GO:0005622|GO:000	NM_014007	Hs.355581	23099	2130.4492
Homo sapiens SUM(	GO:0005622|GO:001	NM_020654	Hs.529551	57337	164.5806
DENN domain-containing protein 1B (Protein FAM31B) [Sourc			Hs.657779		73.73657
Homo sapiens DDB1	GO:0016020|GO:000	NM_025000	Hs.659439	80067	225.46849
Homo sapiens fidgeti	GO:0017111|GO:000	NM_018086	Hs.593650	55137	6.6620092
Homo sapiens spectri	GO:0008219|GO:000	NM_182961	Hs.12967	23345	38.488308
Homo sapiens Alstrom syndrome 1 pseudo		NR_003683	Hs.278983	200420	23.248571
Homo sapiens KIT li	GO:0030097|GO:000	NM_000899	Hs.1048	4254	191.81598
Homo sapiens methy	GO:0005739|GO:001	NM_172250	Hs.452864	166785	12.104419
PREDICTED: Homo sapiens hypothetical		XM_001725589		100130413	11.282091
Homo sapiens zinc fi	GO:0005622|GO:000	NM_152373	Hs.524767	127396	22.26731
Homo sapiens cancer	GO:0005515|GO:000	NM_170589	Hs.181855	57082	168.83412
Homo sapiens 28S ribosomal RNA (LOC1		NR_003287	Hs.552217	100008589	386.01627
Homo sapiens SEC6	GO:0005791|GO:001	NM_003262	Hs.592561	7095	23.235388
Homo sapiens structu	GO:0005622|GO:000	NM_024624	Hs.526728	79677	177.16066
Homo sapiens biorie	GO:0003677	NM_148894	Hs.444517	259282	218.42133
Homo sapiens structu	GO:0005515|GO:005	NM_015295	Hs.8118	23347	295.5912
Homo sapiens valosi	GO:0007067|GO:000	NM_025054	Hs.632066	80124	169.24399
Homo sapiens PMS1	GO:0030983|GO:000	NM_000534	Hs.111749	5378	295.18134
Homo sapiens KIAA	GO:0005515	NM_014802	Hs.271014	9847	703.3781
Homo sapiens DIS3	GO:0005515|GO:000	NM_014953	Hs.643464	22894	8.316523
Homo sapiens zinc fi	GO:0005622|GO:000	NM_152484	Hs.511848	148266	21.35961
Homo sapiens chromosome 10 open readir		NM_014472	Hs.419800	27291	23.268696
Homo sapiens zinc fi	GO:0016020|GO:000	NM_024786	Hs.654874	79844	49.911495
Homo sapiens cDNA FLJ43591 fis, clone SMINT2002743. [AK			Hs.650702	100128019	7.1204824
Homo sapiens zinc fi	GO:0005622|GO:000	NM_020855	Hs.232108	57615	42.91149
Homo sapiens cDNA FLJ45779 fis, clone NETRP2005282. [AK			Hs.683646	100128333	4.9999995
Homo sapiens hypox	GO:0030949|GO:000	NM_181054	Hs.597216	3091	1205.9658
Homo sapiens Alstro	GO:0042632|GO:000	NM_015120	Hs.184720	7840	183.94388
Homo sapiens polyri	GO:0005739|GO:000	NM_033109	Hs.388733	87178	100.448616
Homo sapiens phosp	GO:0005813|GO:000	NM_001002811	Hs.568247	9659	138.6503
Homo sapiens zinc fi	GO:0005622|GO:000	NM_058230	Hs.591734	117608	66.056366
Homo sapiens taste r	GO:0007165|GO:005	NM_023921	Hs.533756	50839	9.00505
Homo sapiens myoze	GO:0005515|GO:003	NM_016599	Hs.381047	51778	4.9999995
Homo sapiens zinc fi	GO:0005622|GO:000	NM_014106	Hs.718498	54989	915.2501
Homo sapiens activa	GO:0005634|GO:004	NM_024997	Hs.513343	80063	21.204206
Homo sapiens chrom	GO:0008150|GO:000	NM_032012	Hs.308074	23731	5758.727
Homo sapiens KIAA1033 (KIAA1033), m		NM_015275	Hs.12144	23325	654.3631
Homo sapiens beta-g	GO:0005529	NM_153605	Hs.714457	131544	262.4185
Homo sapiens desmo	GO:0030057|GO:000	NM_004415	Hs.519873	1832	1464.2764
Homo sapiens zinc fi	GO:0005622|GO:000	NM_001039127	Hs.720296	255403	65.245445
Homo sapiens pinin,	GO:0030057|GO:000	NM_002687	Hs.409965	5411	56.918484
Homo sapiens myelo	GO:0002446|GO:001	NM_182931	Hs.592262	55904	11.095029
Homo sapiens Notch	GO:0005737|GO:000	NM_203458	Hs.709526	388677	20.896404
PREDICTED: Homo sapiens hypothetical		XM_002344316	Hs.720272	100294070	19.749187
Homo sapiens dipept	GO:0006955|GO:000	NM_130434	Hs.591106	54878	1545.1005
Homo sapiens splicir	GO:0008380|GO:000	NM_004768	Hs.479693	9295	1646.3536
Homo sapiens ninein	GO:0005515|GO:005	NM_020921	Hs.310429	51199	323.62442
Homo sapiens protea	GO:0005515|GO:000	NM_001143937	Hs.102798	5682	32.93046
Homo sapiens cDNA FLJ41261 fis, clone BRAMY2034920. [A			Hs.711262		8.444091

205.94289	6.5402327	7.5490737
731.6002	8.330054	9.375074
31.024715	3.742722	4.8171334
3029.7466	9.990011	11.4879875
1339.4796	8.515834	10.269855
266.75473	6.8738422	7.903043
1798.4795	8.829791	10.709755
71.51828	4.4991317	6.037182
45.788017	4.132988	5.3859215
12.637219	2.3362613	3.5044253
4795.3735	11.1550255	12.175219
826.9073	7.4990797	9.552256
272.44547	6.336499	7.936222
585.3479	7.9743648	9.045354
18.650326	2.9114256	4.087101
113.04808	5.3989687	6.6863813
89.083275	4.6789656	6.34321
715.34735	7.7295647	9.345205
31.03962	3.7375045	4.817805
53.02585	3.640894	5.6051083
56.779217	4.610985	5.7046695
1111.5938	7.537945	9.994839
1171.5233	8.74655	10.072744
66.47509	4.6777115	5.931486
935.53326	7.608594	9.726613
1642.7347	7.924984	10.569979
945.5843	8.363453	9.741116
450.56213	7.541629	8.662734
1084.4125	8.361805	9.959696
1854.5476	9.604802	10.753632
69.25843	3.2199259	5.990882
73.28594	4.550786	6.0718517
96.544266	4.6798763	6.4549932
129.90883	5.761037	6.8906446
21.42257	3.0081098	4.2849803
179.60025	5.549378	7.357583
13.743909	2.3362613	3.6339993
3299.645	10.349441	11.608737
884.8391	7.667595	9.646978
284.59195	6.7814136	7.995857
372.3036	7.2479496	8.3813095
186.37563	6.171815	7.408806
30.217175	3.3347962	4.780155
12.085782	2.3362613	3.4379573
2837.6987	9.960626	11.380035
51.170937	4.54115	5.550578
13767.469	12.530206	13.741726
2502.3108	9.495092	11.195282
1208.4357	8.189264	10.11848
4326.8735	10.623793	12.015745
201.91199	6.1569605	7.519821
306.75446	5.953655	8.104525
28.668346	3.6204448	4.7005415
85.884445	4.518008	6.2932887
47.990955	4.4387217	5.453772
5583.868	10.697695	12.404131
4232.168	10.786258	11.98452
1607.731	8.493687	10.539354
97.30838	5.175227	6.46492
23.954731	3.2389941	4.447612

A_23_P144165	2.977208	down	NM_014648	chr3:108412711-108	DZIP3
A_24_P169092	3.2043572	down	NM_032427	chr11:095712434-09	MAML2
A_23_P250571	3.8671542	down	NM_005509	chr5:118584473-118	DMXL1
A_23_P10081	2.174309	down	NM_182896	chr3:093773223-093	ARL13B
A_24_P551028	2.1380165	down	NM_001001664	chr2:139330359-139	SPOPL
A_33_P3879789	2.4105964	down	BC043286	chr19:32084396-320	LOC339316
A_33_P3334404	2.3239263	down	BC034350	chr1:9011597-9011656	
A_33_P3388855	2.021298	down	NM_133259	chr2:044190787-044	LRPPRC
A_33_P3284029	2.1034259	down	NM_001316	chr20:047711430-04	CSE1L
A_33_P3315554	2.2115035	down	NM_016552	chr2:241418904-241	ANKMY1
A_32_P226009	2.3101	down	NM_023073		C5orf42
A_23_P16722	2.541714	down	NM_014689	chr2:225630183-225	DOCK10
A_33_P3256957	2.2482187	down	AK124574	chr12:8490266-8490	LOC100128402
A_33_P3412538	2.9248649	down	NM_032217	chr4:073940618-073	ANKRD17
A_23_P108028	2.7978488	down	NM_007145	chr19:036728855-03	ZNF146
A_23_P56380	2.1749858	down	NM_018471	chr2:187373716-187	ZC3H15
A_23_P43726	2.2686452	down	NM_015231	chr11:047800345-04	NUP160
A_33_P3311473	4.0354381	down	NM_020453	chr4:047595261-047	ATP10D
A_23_P66260	3.5332504	down	NM_003414	chr16:031927638-03	ZNF267
A_23_P123727	3.5088737	down	NM_024617	chr9:088903200-088	ZCCHC6
A_23_P377141	5.0491131	down	NM_015186		VPS13A
A_23_P396981	2.4703465	down	NM_001012506	chr3:056653364-056	CCDC66
A_24_P21056	3.0721944	down	NM_001007157	chr7:011091294-011	PHF14
A_33_P3215277	2.2095797	down	NM_173500	chr15:043036609-04	TTBK2
A_33_P3267160	3.0287166	down	XR_038224	chr6:084675872-084	LOC100132652
A_33_P3406636	2.0222159	down	NM_152283	chr5:180275886-180	ZFP62
A_23_P12816	2.4419353	down	NM_018063	chr10:096354481-09	HELLS
A_23_P84070	3.0135166	down	NM_016648	chr4:113574268-113	LARP7
A_23_P203013	4.2195349	down	NM_002519	chr11:108028826-10	NPAT
A_23_P384056	2.7098707	down	NM_022757	chr3:123632725-123	CCDC14
A_24_P332230	2.8753807	down	NM_016648	chr4:113567946-113	LARP7
A_23_P354074	3.6514531	down	NM_000081	chr1:235824436-235	LYST
A_33_P3214314	2.4673716	down	NM_018999	chr10:086133480-08	FAM190B
A_23_P148807	2.0440132	down	NM_003503	chr1:091991047-091	CDC7
A_23_P35684	2.375307	down	NM_014937	chr10:121588076-12	INPP5F
A_23_P121095	3.1443668	down	NM_001068	chr3:025639773-025	TOP2B
A_23_P115824	2.972146	down	NM_012207	chr10:070102702-07	HNRNPH3
A_23_P35916	3.0966085	down	NM_000051	chr11:108236085-10	ATM
A_23_P217737	3.8091331	down	NM_000052	chrX:077305601-077	ATP7A
A_23_P49988	2.6234853	down	NM_033062	chr17:039333880-03	KRTAP4-2
A_32_P230547	2.7920562	down	NM_033407	chr1:062939716-062	DOCK7
A_23_P80940	2.0059922	down	NM_002703	chr4:057261568-057	PPAT
A_23_P380951	2.675155	down	NM_144689	chr19:037619928-03	ZNF420
A_23_P121222	2.1066301	down	NM_020165	chr3:008921693-008	RAD18
A_33_P3257367	3.7968158	down	NM_015906	chr1:114935511-114	TRIM33
A_23_P208325	2.1954637	down	NM_004234	chr19:044791103-04	ZNF235
A_33_P3271470	2.2053562	down	NM_001142966	chr18:019034450-01	GREB1L
A_32_P50066	3.6551611	down	NM_001039580	chr4:156263949-156	MAP9
A_33_P3365097	2.6650668	down	NM_020729	chr1:086814480-086	ODF2L
A_23_P218608	4.5172414	down	NM_015904	chr2:100015969-100	EIF5B
A_23_P214977	3.0657138	down	NM_007214	chr6:108192374-108	SEC63
A_33_P3594854	2.2565603	down	BI053448	chr15:43952603-439	LOC649956
A_23_P150325	2.0517671	down	NM_032021	chr11:100864382-10	TMEM133
A_23_P30956	2.1138939	down	NM_015323	chr6:097002960-097	KIAA0776
A_33_P3325231	2.1498786	down	AK126007	chr10:047654619-04	LOC728684
A_23_P44466	2.0610967	down	NM_024781	chr18:066722073-06	CCDC102B
A_33_P3340385	2.1179022	down	NM_003423	chr19:021987813-02	ZNF43
A_23_P82950	5.0364633	down	NM_006197		PCM1
A_33_P3279181	2.0600162	down	XM_001726946	chr1:148340545-148	NBPF8
A_23_P110433	2.3344618	down	NM_015342		PPWD1

Homo sapiens DAZ i	GO:0005515|GO:000	NM_014648	Hs.409210	9666	298.52032
Homo sapiens master	GO:0016607|GO:000	NM_032427	Hs.428214	84441	9.193747
Homo sapiens Dmx-l	GO:0005515	NM_005509	Hs.181042	1657	73.67095
Homo sapiens ADP-r	GO:0000166|GO:000	NM_182896	Hs.533086	200894	253.17885
Homo sapiens speckl	GO:0005515|GO:001	NM_001001664	Hs.333297	339745	632.65076
Homo sapiens cDNA clone IMAGE:5297759 [BC043286]			Hs.432564	339316	4.9999995
Carbonic anhydrase 6 Precursor (EC 4.2.1.1)(Carbonic anhydras			Hs.100322		17.6137
Homo sapiens leucine	GO:0042645|GO:000	NM_133259	Hs.368084	10128	315.53992
Homo sapiens CSE1	GO:0005515|GO:000	NM_001316	Hs.90073	1434	8942.893
Homo sapiens ankyri	GO:0008270|GO:004	NM_016552	Hs.656615	51281	4360.136
Homo sapiens chrom	GO:0016020|GO:001	NM_023073	Hs.586199	65250	28.439308
Homo sapiens dedica	GO:0005085|GO:005	NM_014689	Hs.46578	55619	104.870476
Homo sapiens cDNA FLJ42583 fis, clone BRACE3009090. [AK			Hs.689574	100128402	4.9999995
Homo sapiens ankyri	GO:0030983|GO:000	NM_032217	Hs.719092	26057	566.04785
Homo sapiens zinc fi	GO:0005622|GO:000	NM_007145	Hs.643436	7705	604.1947
Homo sapiens zinc fi	GO:0005737|GO:001	NM_018471	Hs.719934	55854	4534.3135
Homo sapiens nucleo	GO:0005515|GO:000	NM_015231	Hs.643526	23279	703.63293
Homo sapiens ATPas	GO:0006754|GO:000	NM_020453	Hs.437241	57205	4.9999995
Homo sapiens zinc fi	GO:0005622|GO:000	NM_003414	Hs.460645	10308	191.10608
Homo sapiens zinc fi	GO:0016779|GO:003	NM_024617	Hs.655162	79670	43.493015
Homo sapiens vacuol	GO:0005622|GO:000	NM_015186	Hs.459790	23230	46.195786
Homo sapiens coiled-coil domain containi		NM_001012506	Hs.476399	285331	79.218994
Homo sapiens PHD f	GO:0005515|GO:000	NM_001007157	Hs.655688	9678	164.52289
Homo sapiens tau tub	GO:0008219|GO:000	NM_173500	Hs.659846	146057	393.03528
PREDICTED: Homo sapiens similar to hC		XR_038224	Hs.650662	100132652	77.09471
Homo sapiens zinc fi	GO:0005622|GO:000	NM_152283	Hs.509227	643836	10.69559
Homo sapiens helicas	GO:0031508|GO:000	NM_018063	Hs.655830	3070	649.2045
Homo sapiens La ribo	GO:0006396|GO:000	NM_016648	Hs.713663	51574	337.93063
Homo sapiens nuclea	GO:0007049|GO:004	NM_002519	Hs.171061	4863	48.769306
Homo sapiens coiled-coil domain containi		NM_022757	Hs.645028	64770	1134.6677
Homo sapiens La ribo	GO:0006396|GO:000	NM_016648	Hs.713663	51574	552.94366
Homo sapiens lysoso	GO:0005515|GO:003	NM_000081	Hs.532411	1130	44.578724
Homo sapiens family with sequence simila		NM_018999	Hs.461988	54462	472.9406
Homo sapiens cell di	GO:0005515|GO:000	NM_003503	Hs.533573	8317	1078.6409
Homo sapiens inosito	GO:0016787	NM_014937	Hs.369755	22876	154.36636
Homo sapiens topois	GO:0000792|GO:000	NM_001068	Hs.475733	7155	1713.3546
Homo sapiens hetero	GO:0008380|GO:000	NM_012207	Hs.643472	3189	1515.5945
Homo sapiens ataxia	GO:0007094|GO:000	NM_000051	Hs.367437	472	155.80595
Homo sapiens ATPas	GO:0005802|GO:005	NM_000052	Hs.496414	538	4.9999995
Homo sapiens keratin	GO:0045095	NM_033062	Hs.380165	85291	10.309929
Homo sapiens dedica	GO:0030424|GO:004	NM_033407	Hs.406156	85440	517.8015
Homo sapiens phospl	GO:0051539|GO:000	NM_002703	Hs.331420	5471	1017.3757
Homo sapiens zinc fi	GO:0005622|GO:000	NM_144689	Hs.444992	147923	39.37728
Homo sapiens RAD1	GO:0005515|GO:000	NM_020165	Hs.375684	56852	480.49786
Homo sapiens tripart	GO:0005515|GO:001	NM_015906	Hs.26837	51592	363.1033
Homo sapiens zinc fi	GO:0005622|GO:000	NM_004234	Hs.298089	9310	21.64032
Homo sapiens growth	GO:0016020|GO:001	NM_001142966	Hs.149020	80000	7.4824753
Homo sapiens microt	GO:0007067|GO:000	NM_001039580	Hs.61271	79884	91.667755
Homo sapiens outer dense fiber of sperm ta		NM_020729	Hs.149360	57489	111.88937
Homo sapiens eukary	GO:0005515|GO:000	NM_015904	Hs.158688	9669	381.73822
Homo sapiens SEC63	GO:0016020|GO:000	NM_007214	Hs.26904	11231	987.43317
RC0-GN0275-250101-021-c04 GN0275 Homo sapiens cDNA, r			Hs.679911	649956	6.7375584
Homo sapiens transm	GO:0016020|GO:001	NM_032021	Hs.44004	83935	193.52043
Homo sapiens KIAA	GO:0005515	NM_015323	Hs.149367	23376	239.8042
Homo sapiens cDNA FLJ44019 fis, clone TESTI4026192 [AK1			Hs.640184	728684	5.7198095
Homo sapiens coiled-coil domain containi		NM_024781	Hs.280781	79839	5.047553
Homo sapiens zinc fi	GO:0005622|GO:000	NM_003423	Hs.534365	7594	83.975395
Homo sapiens perice	GO:0051297|GO:000	NM_006197	Hs.491148	5108	548.27246
PREDICTED: Homo	GO:0005737	XM_001726946	Hs.534675	728841	26.137648
Homo sapiens peptid	GO:0008380|GO:000	NM_015342	Hs.121432	23398	1287.2335

1081.8392	8.380539	9.954499
36.29189	3.362208	5.042243
348.3655	6.3351517	8.286424
674.7029	8.141267	9.261824
1613.6818	9.446484	10.542757
13.501592	2.3362613	3.6056514
49.067337	4.2745247	5.491089
783.73315	8.456307	9.471589
19007.336	13.14369	14.216431
10144.315	12.1430645	13.288092
79.09925	4.9709377	6.178893
324.38327	6.8416853	8.187487
12.641	2.3362613	3.5050437
1952.0763	9.28264	10.83101
1999.5437	9.378771	10.863089
10319.817	12.198274	13.31928
1896.0667	9.605465	10.787296
22.31196	2.3362613	4.3489866
822.8401	7.723548	9.544544
182.31389	5.5655246	7.3765326
283.86014	5.655266	7.991296
238.18895	6.446247	7.7509604
614.7548	7.4987345	9.118004
1058.594	8.772526	9.916298
285.7199	6.4041324	8.002839
26.332958	3.569705	4.585642
1877.9163	9.4849	10.772925
1233.2483	8.556761	10.148209
248.3905	5.727957	7.805041
3494.098	10.265774	11.703998
1878.1844	9.249905	10.773658
194.55194	5.6013203	7.469791
1400.234	9.034553	10.337528
2551.2288	10.1911335	11.222538
446.32135	7.402374	8.650488
5920.6895	10.844873	12.4976425
5038.746	10.675463	12.246968
585.78876	7.416017	9.046706
21.152597	2.3362613	4.265724
33.24153	3.520864	4.9123487
1727.155	9.160891	10.642219
2370.9622	10.111735	11.116051
126.21109	5.4315615	6.851184
1217.9211	9.054049	10.128986
1654.8512	8.657613	10.582403
56.792427	4.5703955	5.7049212
20.491568	3.0790763	4.220088
410.49966	6.653514	8.523449
363.13257	6.9340553	8.348227
2059.1423	8.732008	10.90745
3454.543	10.067088	11.683311
18.866062	2.9285917	4.102717
489.44608	7.744726	8.781593
623.1513	8.060165	9.140068
15.49954	2.7077112	3.8119664
13.2130995	2.5303798	3.573792
215.33894	6.527913	7.610549
3222.684	9.239235	11.571646
64.481384	4.8439293	5.886585
3432.9805	10.445623	11.668713

A_24_P110558	3.6695842	down	NM_001007189	chr5:139508041-139	C5orf53
A_24_P67806	2.8069129	down	NM_000254	chr1:237066633-237	MTR
A_23_P318300	2.8126519	down	NM_133646	chr2:174091756-174	ZAK
A_23_P108932	2.8163839	down	NR_002229	chr2:054756368-054	RPL23AP32
A_23_P102471	2.3758175	down	NM_000251	chr2:047710161-047	MSH2
A_23_P35576	3.2615777	down	NM_014803	chr10:097920015-09	ZNF518A
A_33_P3413741	2.5306018	down	NM_000916	chr3:008792187-008	OXTR
A_23_P132057	2.6470646	down	NR_024027	chr21:026758626-02	NCRNA00158
A_33_P3407540	3.2985311	down	NM_001128835	chrX:013733706-01:	TRAPPC2
A_33_P3378334	3.9890743	down	NM_001042550	chr9:106896796-106	SMC2
A_23_P255714	2.8866646	down	NM_025103	chr9:027056396-027	IFT74
A_23_P213661	3.0743747	down	NM_015216	chr5:102538799-102	HISPPD1
A_23_P121926	2.0477983	down	NM_005410	chr5:042800539-042	SEPP1
A_33_P3354539	2.7014553	down	NM_145165	chr14:065401707-06	CHURC1
A_33_P3316147	2.5164151	down	NM_001005504	chr6:000105982-000	OR4F21
A_23_P118834	4.0250452	down	NM_001067	chr17:038545768-03	TOP2A
A_32_P51084	2.3827259	down	NM_015135	chr7:135333302-135	NUP205
A_24_P153576	2.6618932	down	NM_173082	chr6:146266660-146	SHPRH
A_23_P339240	3.5484498	down	NM_014996	chr3:155197964-155	PLCH1
A_24_P161293	2.7880917	down	XM_002346226	chr1:145318194-145318253	
A_33_P3304107	2.1556783	down	NM_017658	chr14:045398109-04	KLHL28
A_23_P11598	2.3808488	down	NM_152373	chr1:041012893-041	ZNF684
A_33_P3409077	2.150153	down	NM_152730	chr6:121434268-121	C6orf170
A_23_P65262	2.3232376	down	NM_033111	chr13:033016630-03	N4BP2L2
A_23_P67702	2.6994622	down	NM_003429	chr19:021132382-02	ZNF85
A_24_P106357	2.0836602	down	NM_139281	chr5:110465700-110	WDR36
A_33_P3380897	2.0825007	down	NM_001695	chr8:104085226-104	ATP6V1C1
A_24_P83437	2.0421158	down	NM_182975	chr1:090470867-090	ZNF326
A_24_P808100	2.8039332	down	NM_014060	chrX:119754663-11!	MCTS1
A_23_P2705	2.5173259	down	NM_005767	chr13:048985730-04	LPAR6
A_33_P3704280	2.63321	down	AK095428	chr5:153813457-153	FLJ38109
A_33_P3361771	4.6548962	down	NM_003292	chr1:186280923-186	TPR
A_33_P3352253	4.3796425	down	NM_001001925	chr8:017611347-017	MTUS1
A_23_P202496	2.4052619	down	NM_022451	chr10:096093097-09	NOC3L
A_24_P164505	3.7518829	down	NR_026810	chr17:018428630-01	LOC100129396
A_33_P3438072	2.6114942	down	AK055863	chr8:9192362-91924	LOC157273
A_33_P3543133	4.7582996	down	BC035145	chr14:21668348-216	LOC283624
A_23_P15876	2.2489927	down	NM_052947	chr18:056148827-05	ALPK2
A_24_P77947	2.6039006	down	NM_024553	chr7:092887515-092	CCDC132
A_33_P3389298	2.3209863	down	NM_001099438	chr19:035435506-03	ZNF30
A_23_P359245	2.2130568	down	NM_000245	chr7:116438290-116	MET
A_33_P3272568	2.0347834	down		chrX:23693494-236!	LOC100292021
A_33_P3243832	2.1381529	down	NM_014795	chr2:145182422-145	ZEB2
A_23_P112341	2.3697855	down	NM_024945	chr9:086618846-086	RMI1
A_24_P119259	2.1481374	down	NM_003666	chr1:169346119-169	BLZF1
A_33_P3310475	2.983304	down	NM_018243	chr4:077959708-077	SEPT11
A_33_P3884179	3.6364617	down	AK057596	chr2:96479923-9647	LOC150759
A_32_P159400	3.2842765	down	NM_015902	chr8:103271354-103	UBR5
A_33_P3371752	2.9245243	down		chr1:51923267-51923208	
A_23_P435002	2.3987362	down	NM_152546	chr5:121363253-121	SRFBP1
A_24_P114438	2.1046427	down	NM_013341	chr2:174937535-174	OLA1
A_33_P3456341	2.2429827	down	NM_199188	chr12:050855064-05	LARP4
A_33_P3356525	2.2076822	down	AK127393	chr7_gl000195_rand	FLJ45482
A_23_P119964	3.2781389	down	NM_005760	chr2:037430001-037	CEBPZ
A_33_P3666797	2.1354803	down	AL832853	chr13:95285260-952	LOC144874
A_33_P3211513	2.0493921	down	NM_001162407	chr2:201726021-201	CLK1
A_24_P172768	2.0156302	down	NM_004124	chr14:054941669-05	GMFB
A_23_P144843	2.9079007	down	NM_007036	chr5:054274617-054	ESM1
A_33_P3376131	5.6838438	down	XM_001715677	chr2:113500330-113	NT5DC4
A_23_P117515	4.0521447	down	NM_002892	chr14:058838770-05	ARID4A

Homo sapiens chrom	GO:0005576	NM_001007189	Hs.696360	492311	60.8062
Homo sapiens 5-metl	GO:0005515|GO:003	NM_000254	Hs.498187	4548	66.69595
Homo sapiens sterile	GO:0009314|GO:000	NM_133646	Hs.444451	51776	2271.3088
Homo sapiens ribosomal protein L23a pseu		NR_002229	Hs.657366	56969	42.241005
Homo sapiens mutS l	GO:0019724|GO:001	NM_000251	Hs.597656	4436	3623.9446
Homo sapiens zinc fi	GO:0005622|GO:000	NM_014803	Hs.600823	9849	25.659172
Homo sapiens oxytoc	GO:0005515|GO:001	NM_000916	Hs.2820	5021	46.191433
Homo sapiens non-protein coding RNA 15		NR_024027	Hs.234016	54072	4.9999995
Homo sapiens traffic	GO:0006888|GO:000	NM_001128835	Hs.592238	6399	17.577562
Homo sapiens structu	GO:0005515|GO:000	NM_001042550	Hs.119023	10592	289.78952
Homo sapiens intrafl	GO:0016023	NM_025103	Hs.145402	80173	235.39348
Homo sapiens histidi	GO:0033857|GO:000	NM_015216	Hs.212046	23262	971.0654
Homo sapiens seleno	GO:0008430|GO:000	NM_005410	Hs.275775	6414	5.658311
Homo sapiens church	GO:0045941|GO:001	NM_145165	Hs.325531	91612	37.027985
Homo sapiens olfact	GO:0007608|GO:000	NM_001005504	Hs.690459	441308	5.530104
Homo sapiens topois	GO:0043130|GO:004	NM_001067	Hs.156346	7153	2239.0168
Homo sapiens nucleo	GO:0005515|GO:000	NM_015135	Hs.632299	23165	3820.626
Homo sapiens SNF2	GO:0005515|GO:001	NM_173082	Hs.124537	257218	124.23721
Homo sapiens phosp	GO:0016042|GO:000	NM_014996	Hs.567423	23007	4.9999995
Novel protein [Source:UniProtKB/TrEMB		XM_002346226	Hs.445080		6305.2393
Homo sapiens kelch-	GO:0005515	NM_017658	Hs.550906	54813	47.60087
Homo sapiens zinc fi	GO:0005622|GO:000	NM_152373	Hs.524767	127396	41.226128
Homo sapiens chrom	GO:0005622|GO:003	NM_152730	Hs.121396	221322	8.9135895
Homo sapiens NEDD	GO:0008150|GO:000	NM_033111	Hs.507680	10443	61.461895
Homo sapiens zinc fi	GO:0005622|GO:000	NM_003429	Hs.37138	7639	1289.3131
Homo sapiens WD re	GO:0003674|GO:000	NM_139281	Hs.533237	134430	410.3073
Homo sapiens ATPas	GO:0005515|GO:000	NM_001695	Hs.86905	528	996.42413
Homo sapiens zinc fi	GO:0005622|GO:000	NM_182975	Hs.306221	284695	5.7009244
Homo sapiens malign	GO:0008284|GO:000	NM_014060	Hs.102696	28985	22.45971
Homo sapiens lysoph	GO:0007165|GO:000	NM_005767	Hs.123464	10161	4.9999995
Homo sapiens cDNA FLJ38109 fis, clone D3OST2001788 [AK			Hs.652083	386627	12.91618
Homo sapiens translo	GO:0005515|GO:000	NM_003292	Hs.279640	7175	29.504255
Homo sapiens microt	GO:0005739|GO:000	NM_001001925	Hs.7946	57509	4.9999995
Homo sapiens nucleo	GO:0016607|GO:000	NM_022451	Hs.74899	64318	2085.279
Homo sapiens family with sequence simila		NR_026810	Hs.712114	100129396	6.3780217
Homo sapiens cDNA FLJ31301 fis, clone LIVER1000073 [AK			Hs.650222	157273	4.9999995
Homo sapiens cDNA clone IMAGE:5264647 [BC035145]			Hs.525210	283624	13.09923
Homo sapiens alpha-	GO:0016301|GO:000	NM_052947	Hs.628152	115701	29.528645
Homo sapiens coiled-coil domain containi		NM_024553	Hs.222282	55610	15.087918
Homo sapiens zinc fi	GO:0005622|GO:000	NM_001099438	Hs.657402	90075	55.425194
Homo sapiens met pr	GO:0005008|GO:000	NM_000245	Hs.132966	4233	2525.8132
Peroxiredoxin-4 (EC 1.11.1.15)(Prx-IV)(Thioredoxin peroxidase AO372)(Thioredoxi				100292021	25.069826
Homo sapiens zinc fi	GO:0021846|GO:000	NM_014795	Hs.34871	9839	10.975861
Homo sapiens RMI1,	GO:0006260|GO:000	NM_024945	Hs.284137	80010	2238.6143
Homo sapiens basic l	GO:0005737|GO:000	NM_003666	Hs.130746	8548	78.88964
Homo sapiens septin	GO:0031105|GO:000	NM_018243	Hs.128199	55752	2072.5962
Homo sapiens cDNA FLJ33034 fis, clone THYMU2000236 [A			Hs.651352	150759	91.289665
Homo sapiens ubiqui	GO:0005515|GO:005	NM_015902	Hs.591856	51366	173.90526
Epidermal growth factor receptor substrate 15 (Protein Eps15)(Protein AF-1p) [Source:UniProtKB/Swiss-P					565.3205
Homo sapiens serum	GO:0005515|GO:004	NM_152546	Hs.107622	153443	250.00046
Homo sapiens Obg-li	GO:0005622|GO:000	NM_013341	Hs.157351	29789	85.77302
Homo sapiens La rib	GO:0003723	NM_199188	Hs.26613	113251	144.96101
Homo sapiens cDNA FLJ45482 fis, clone BRTHA2001953. [A			Hs.644332	645566	70.88095
Homo sapiens CCAA	GO:0006366|GO:000	NM_005760	Hs.135406	10153	1136.8239
Homo sapiens mRNA; cDNA DKFZp667K0825 (from clone DI			Hs.439363	144874	28.49531
Homo sapiens CDC-l	GO:0046777|GO:001	NM_001162407	Hs.433732	1195	229.28008
Homo sapiens glia m	GO:0005622|GO:000	NM_004124	Hs.151413	2764	1384.986
Homo sapiens endot	GO:0005515|GO:000	NM_007036	Hs.129944	11082	4.9999995
5'-nucleotidase doma	GO:0000287|GO:001	XM_001715677	Hs.589714	284958	5.2548194
Homo sapiens AT ric	GO:0016564|GO:000	NM_002892	Hs.161000	5926	44.530777

270.58875	6.050117	7.9257336
226.2162	6.18869	7.6776743
6978.1787	11.2388	12.730731
141.89352	5.5262456	7.0200896
9089.543	11.883577	13.132001
101.27459	4.820256	6.525826
139.65259	5.654806	6.9942865
14.707797	2.3362613	3.7406547
69.36718	4.2717037	5.9935274
1393.184	8.333685	10.329739
833.7137	8.0334015	9.562805
3427.4297	10.045324	11.665617
14.56127	2.6893737	3.7234473
120.12522	5.3429136	6.7766504
17.448551	2.6596496	3.9910195
9735.85	11.223192	13.232197
9653.794	11.965288	13.217901
404.84995	7.0920563	8.504509
19.673998	2.3362613	4.1634502
17890.895	12.652382	14.13166
122.43422	5.6959944	6.8041363
117.352776	5.491089	6.742565
23.539972	3.3185463	4.4229856
170.79918	6.065675	7.2818117
3932.7593	10.447777	11.880449
1042.7278	8.830162	9.889282
2415.7373	10.082672	11.140989
14.642102	2.701619	3.7316837
75.46969	4.6247554	6.1122074
14.04662	2.3362613	3.6681533
41.144264	3.8319764	5.228799
165.92201	5.02295	7.241699
24.261957	2.3362613	4.4670744
5517.9307	11.120149	12.386343
29.903515	2.8548965	4.7625113
14.534499	2.3362613	3.7211368
74.704025	3.8506079	6.101054
80.02065	5.023819	6.193098
47.074947	4.0440054	5.4246798
153.22786	5.914378	7.129116
6108.1387	11.3917265	12.537767
61.01989	4.7842135	5.8090887
28.696796	3.6056747	4.7020397
5802.1533	11.2228985	12.467655
205.35155	6.4396257	7.542712
6746.4443	11.110522	12.687433
406.38873	6.6477046	8.51024
690.35077	7.5800924	9.295668
1948.3661	9.280838	10.82904
737.1089	8.1236725	9.385947
219.04935	6.560589	7.6341643
397.00275	7.3108935	8.476312
187.90376	6.27857	7.4211025
4221.126	10.268536	11.981413
73.20013	4.9748287	6.0693893
580.65814	7.997225	9.032421
3201.3179	10.548702	11.559933
16.18571	2.3362613	3.8762393
37.679085	2.5901191	5.096986
216.68338	5.5997543	7.61844

A_24_P196704	2.9264933	down	NM_182931	chr7:104715141-104	MLL5
A_23_P8961	2.388476	down	NM_000880	chr8:079645431-079	IL7
A_33_P3665777	2.5422167	down	NM_001017963	chr14:102547454-10	HSP90AA1
A_32_P204376	2.9797672	down	NM_001012421	chr13:019409033-01	ANKRD20A2
A_33_P3229161	2.347529	down	NM_021645	chr13:052605988-05	UTP14C
A_23_P401	5.5765235	down	NM_016343	chr1:214837308-214	CENPF
A_23_P140035	2.1636715	down	NM_007187	chr13:041657065-04	WBP4
A_23_P364478	2.026518	down	NM_032182	chr10:126524866-12	FAM175B
A_33_P3386429	3.796811	down	NM_015571	chr6:076427665-076	SENP6
A_33_P3249354	2.3024614	down	NM_017785	chr5:169031146-169	CCDC99
A_23_P99027	2.3649063	down	NM_002834	chr12:112946884-11	PTPN11
A_23_P253464	2.323478	down	NM_139076	chr4:084382373-084	FAM175A
A_33_P3304824	2.7447067	down		chr14:97411668-97411727	
A_23_P13740	2.8315363	down	NM_014903	chr12:078606196-07	NAV3
A_33_P3319140	2.0146994	down	BC039117	chr12:31274321-312	OVOS2
A_33_P3673310	2.1181483	down		chr15:035234990-035234931	
A_33_P3420747	2.8630126	down	NM_022828	chr5:112930732-112	YTHDC2
A_33_P3436646	2.5336513	down	AK091114	chr3:107752757-107	LOC151657
A_23_P252052	2.3764466	down	NM_182909	chr3:099567410-099	FILIP1L
A_24_P929369	2.0889023	down	NM_007347	chr15:051297921-05	AP4E1
A_23_P39517	2.0898929	down	NM_018079	chr2:045616162-045	SRBD1
A_33_P3365750	4.9288764	down	NM_183387	chr14:089081388-08	EML5
A_23_P33856	3.388665	down	NM_001042749	chrX:123227937-12[illegible]	STAG2
A_33_P3362498	3.7904347	down	NM_001184	chr3:142185226-142	ATR
A_33_P3292337	3.4402443	down	NM_004521	chr10:032306139-03	KIF5B
A_23_P429491	2.0305478	down	NM_145018	chr11:082645351-08	C11orf82
A_23_P53193	2.1147997	down	NM_206927	chr11:085406136-08	SYTL2
A_33_P3293114	2.0630946	down	NM_004768	chr1:070715651-070	SFRS11
A_24_P126417	4.4550833	down	NM_014709	chr2:061415492-061	USP34
A_24_P261383	2.6460714	down	NM_024116	chr11:093469411-09	TAF1D
A_33_P3243878	2.8369525	down	NM_198181	chr15:082810832-08	GOLGA6L9
A_23_P93938	2.0651437	down	NM_001146334	chr7:045120849-045	NACAD
A_33_P3326210	2.160311	down	NM_001017420	chr8:027660830-027	ESCO2
A_33_P3325087	4.0704012	down	NM_032141	chr1:078314501-078	CCDC55
A_24_P288915	2.9969919	down	AK093811	chr17:16615092-16615151	
A_33_P3307810	2.0131819	down	NM_138803	chr2:159028665-159	CCDC148
A_23_P148463	2.2906102	down	NM_003588	chrX:119659059-11[illegible]	CUL4B
A_32_P207428	2.0678909	down	NM_138374	chr19:053857741-05	ZNF845
A_32_P70056	9.6293252	down	XR_041426	chr1:109400785-109	LOC642864
A_33_P3328026	2.0720977	down	NM_015076	chr6:110931295-110	CDC2L6
A_23_P59388	4.1423251	down	NM_001723	chr6:056480038-056	DST
A_33_P3322450	2.7654129	down		chr12:70190287-70190346	
A_23_P254025	2.7528118	down	NM_003408	chr9:115804911-115	ZFP37
A_33_P3404097	3.9356748	down	NR_002836	chr9:069080401-069	PGM5P2
A_23_P26094	3.4909181	down	NM_024755	chr15:059171417-05	SLTM
A_23_P31315	2.1688819	down	NM_016587	chr7:026251960-026	CBX3
A_24_P285501	2.4332997	down	NM_172070	chr2:170940151-170	UBR3
A_23_P398172	2.2139878	down	NM_020819	chr6:071269898-071	FAM135A
A_23_P28948	2.2185454	down	NM_014012	chr20:030065637-03	REM1
A_33_P3213089	2.8379005	down		chr13:019380513-019380454	
A_24_P658427	2.0241006	down	NM_005596	chr9:014081998-014	NFIB
A_23_P350895	2.9258512	down	AK022408	chr9:125752685-125752744	
A_23_P250404	3.9908687	down	NM_005732		RAD50
A_33_P3210278	3.6083828	down	NM_182914	chr14:064693075-06	SYNE2
A_23_P212522	2.7422441	down	NM_014616	chr3:182638622-182	ATP11B
A_33_P3348234	5.5153074	down	AK024925	chr1:144990583-144990642	
A_24_P418637	2.6660429	down	NM_012090		MACF1
A_23_P397347	2.30803	down	NM_153255	chr6:119232007-119	MCM9
A_33_P3415623	2.0592704	down	NM_032165	chr12:085518417-08	LRRIQ1
A_33_P3374723	4.2584901	down	NM_001128128	chr10:031818063-03	ZEB1

Homo sapiens myelo	GO:0002446|GO:001	NM_182931	Hs.592262	55904	581.2734
Homo sapiens interle	GO:0005515|GO:001	NM_000880	Hs.591873	3574	35.388725
Homo sapiens heat sl	GO:0006986|GO:004	NM_001017963	Hs.525600	3320	44514.242
Homo sapiens ankyrin repeat domain 20 fa		NM_001012421	Hs.632663	441430	7.1359296
Homo sapiens UTP1	GO:0007126|GO:000	NM_021645	Hs.512963	9724	69.17813
Homo sapiens centro	GO:0007094|GO:004	NM_016343	Hs.497741	1063	4441.7188
Homo sapiens WW d	GO:0008380|GO:000	NM_007187	Hs.411300	11193	521.5459
Homo sapiens family	GO:0005515|GO:007	NM_032182	Hs.280695	23172	443.40448
Homo sapiens SUMO	GO:0019941|GO:000	NM_015571	Hs.485784	26054	6.3625665
Homo sapiens coiled	GO:0005515|GO:000	NM_017785	Hs.368710	54908	1763.2244
Homo sapiens protei	GO:0006629|GO:000	NM_002834	Hs.506852	5781	2037.0406
Homo sapiens family	GO:0005515|GO:007	NM_139076	Hs.334772	84142	280.64465
					111.64091
Homo sapiens neuro	GO:0005640|GO:001	NM_014903	Hs.655301	89795	283.1742
Homo sapiens ovosta	GO:0005515|GO:0004867|GO:0005576|G		Hs.524331	144203	21.432322
					55.436058
Homo sapiens YTH	GO:0016787|GO:000	NM_022828	Hs.231942	64848	418.42026
Homo sapiens cDNA FLJ33795 fis, clone CTONG1000097 [AK			Hs.587187	151657	7.7609935
Homo sapiens filami	GO:0008150|GO:000	NM_182909	Hs.104672	11259	14.597212
Homo sapiens adapto	GO:0005515|GO:003	NM_007347	Hs.413366	23431	677.6507
Homo sapiens S1 RN	GO:0006139|GO:000	NM_018079	Hs.14229	55133	305.0349
Homo sapiens echino	GO:0005737|GO:000	NM_183387	Hs.558671	161436	9.208855
Homo sapiens stroma	GO:0005515|GO:000	NM_001042749	Hs.496710	10735	1109.5272
Homo sapiens ataxia	GO:0001741|GO:000	NM_001184	Hs.271791	545	54.400417
Homo sapiens kinesi	GO:0005515|GO:000	NM_004521	Hs.327736	3799	468.244
Homo sapiens chrom	GO:0007049|GO:000	NM_145018	Hs.165607	220042	308.7965
Homo sapiens synapt	GO:0042470|GO:001	NM_206927	Hs.369520	54843	45.461315
Homo sapiens splicin	GO:0008380|GO:000	NM_004768	Hs.479693	9295	2051.8606
Homo sapiens ubiqui	GO:0006511|GO:000	NM_014709	Hs.644708	9736	88.15613
Homo sapiens TATA	GO:0005515|GO:000	NM_024116	Hs.355750	79101	2024.6965
Homo sapiens golgi autoantigen, golgin su		NM_198181	Hs.630181	440295	629.54816
Homo sapiens NAC	GO:0005737|GO:000	NM_001146334	Hs.96633	23148	5.0926776
Homo sapiens establi	GO:0003684|GO:000	NM_001017420	Hs.99480	157570	126.62332
Homo sapiens coiled	GO:0016607	NM_032141	Hs.462663	84081	292.2357
Coiled-coil domain-containing protein 144A [Source:UniProtK			Hs.649499		55.82778
Homo sapiens coiled-coil domain containi		NM_138803	Hs.668597	130940	32.209656
Homo sapiens cullin	GO:0005515|GO:003	NM_003588	Hs.102914	8450	480.4334
Homo sapiens zinc fi	GO:0005622|GO:000	NM_138374	Hs.720672	91664	112.57782
PREDICTED: Homo sapiens spermatogen		XR_041426	Hs.372658	642864	4.9999995
Homo sapiens cell di	GO:0005515|GO:000	NM_015076	Hs.584867	23097	446.432
Homo sapiens dystor	GO:0005515|GO:003	NM_001723	Hs.604915	667	53.598053
RAB3A-interacting protein (Rabin-3)(SSX2-interacting protein) [Source:UniProtKB/Swiss-Prot;Acc:Q96Q					4.9999995
Homo sapiens zinc fi	GO:0005622|GO:000	NM_003408	Hs.150406	7539	28.064596
Homo sapiens phosphoglucomutase 5 pseu		NR_002836	Hs.434753	595135	8977.246
Homo sapiens SAFB	GO:0000166|GO:000	NM_024755	Hs.512932	79811	576.4913
Homo sapiens chrom	GO:0016565|GO:000	NM_016587	Hs.381189	11335	7428.5493
Homo sapiens ubiqui	GO:0007608|GO:000	NM_172070	Hs.379548	130507	172.26335
Homo sapiens family with sequence simila		NM_020819	Hs.211700	57579	88.11374
Homo sapiens RAS (	GO:0007165|GO:000	NM_014012	Hs.247729	28954	4.9999995
					20.262968
Homo sapiens nuclea	GO:0006260|GO:000	NM_005596	Hs.644095	4781	502.208
Rab GTPase-activating protein 1 (Rab6 GTPase-activating prote			Hs.271341		23.606853
Homo sapiens RAD5	GO:0008408|GO:003	NM_005732	Hs.655835	10111	108.61178
Homo sapiens spectr	GO:0031965|GO:000	NM_182914	Hs.525392	23224	190.65613
Homo sapiens ATPa	GO:0000287|GO:001	NM_014616	Hs.478429	23200	210.44814
CDNA: FLJ21272 fis, clone COL01753 [Source:UniProtKB/Tr			Hs.612891		4.9999995
Homo sapiens micro	GO:0005515|GO:000	NM_012090	Hs.580782	23499	660.55884
Homo sapiens minicl	GO:0006260|GO:001	NM_153255	Hs.279008	254394	78.397804
Homo sapiens leucin	GO:0005515	NM_032165	Hs.402200	84125	4.9999995
Homo sapiens zinc fi	GO:0007389|GO:000	NM_001128128	Hs.124503	6935	457.01016

2006.5494	9.318753	10.867926
101.87931	5.2789426	6.535033
113382.03	15.441366	16.787453
26.332296	3.0101247	4.5853243
195.06679	6.2420406	7.4731836
25551.662	12.170576	14.649942
1354.5813	9.1711855	10.284667
1084.9459	8.941769	9.960772
30.147564	2.8518708	4.776659
4572.652	10.891332	12.094509
5319.48	11.085105	12.326888
803.2748	8.290165	9.506451
373.83282	6.930965	8.387617
987.06836	8.302612	9.804197
51.596466	4.556235	5.5667996
139.90323	5.9150944	6.997898
1434.476	8.85532	10.372854
24.3692	3.131065	4.472283
41.742672	4.000991	5.249797
1682.3674	9.544408	10.607153
785.49243	8.411135	9.474564
55.40847	3.3644052	5.665664
4265.0693	10.233931	11.994648
249.45966	5.8888273	7.8111906
1915.8989	9.018762	10.801273
771.62134	8.428689	9.450558
115.02402	5.63335	6.713871
4686.973	11.095704	12.140514
480.1451	6.5989146	8.754367
5854.8174	11.076317	12.480169
2105.1191	9.437455	10.941797
13.37239	2.5452416	3.5914838
333.30963	7.117223	8.228462
1433.51	8.346366	10.371537
200.1669	5.9255342	7.5090494
77.96497	5.1460075	6.155485
1319.2483	9.053576	10.249308
283.5983	6.9421425	7.9903026
52.979546	2.3362613	5.603696
1116.6829	8.952173	10.003265
269.07278	5.8680925	7.9185333
15.403119	2.3362613	3.8037562
93.63413	4.952309	6.413215
35485.395	13.147848	15.124459
2362.2642	9.308311	11.1119175
16492.39	12.8907175	14.007669
510.95932	7.565753	8.848667
237.71857	6.5980787	7.744726
12.46595	2.3362613	3.4858754
68.71628	4.474989	5.979813
1223.0062	9.118004	10.135285
83.250435	4.701907	6.2507634
524.7863	6.8934402	8.890143
839.893	7.7203226	9.571675
707.83856	7.875108	9.330465
30.597954	2.3362613	4.7997026
2083.013	9.512074	10.926774
219.63106	6.43083	7.637492
11.624208	2.3362613	3.3783946
2302.7266	8.98583	11.076172

A_24_P782308	2.087957	down	NM_001144967	chr18:056068043-05	NEDD4L
A_32_P58614	3.87971	down	NM_020802	chr11:101871701-10	KIAA1377
A_33_P3307307	2.771825	down	NM_005933	chr11:118395871-11	MLL
A_24_P941708	2.0286852	down	NM_001042417	chr10:070137124-07	RUFY2
A_23_P110661	2.3862728	down	NM_015360	chr5:054720638-054	SKIV2L2
A_33_P3407691	4.3118282	down		chrUn_gl000212:000001369-000001310	
A_33_P3861385	3.325338	down	AK096159	chr9:68743601-6874	PGM5P1
A_33_P3851023	2.0539214	down	NM_004796	chr14:080328254-08	NRXN3
A_23_P211797	3.7436278	down	NM_130837	chr3:193414874-193	OPA1
A_33_P3685572	2.5977302	down	BX649145	chr8:102179109-102	LOC157562
A_23_P76799	2.566987	down	NM_013448	chr14:035222268-03	BAZ1A
A_33_P3257187	2.1350162	down	NM_206907	chr5:040759657-040	PRKAA1
A_23_P205646	2.8306512	down	NM_198794	chr14:050885844-05	MAP4K5
A_23_P43248	2.2413004	down	NM_003184	chr8:120743869-120	TAF2
A_33_P3462692	2.0912981	down	BE467637	chr3:131198300-131	SNORA58
A_23_P18123	2.0211783	down	NM_014932	chr3:174000648-174	NLGN1
A_33_P3221753	2.6415047	down	NM_199352	chr11:062931390-06	SLC22A25
A_33_P3221253	4.0517299	down	NM_005578	chr3:188597359-188	LPP
A_23_P85004	2.0582986	down	NM_007309	chrX:096369895-09(	DIAPH2
A_33_P3758973	2.8146456	down	AL137340	chr11:84095347-840	DKFZP761C1711
A_33_P3242798	2.3229861	down	NM_001875	chr2:211543741-211	CPS1
A_23_P501080	3.0677196	down	NM_007139	chr7:064865861-064	ZNF92
A_23_P99452	3.622555	down	NM_000059	chr13:032972996-03	BRCA2
A_23_P384329	2.2871637	down	NM_001144823	chr15:065953564-06	DENND4A
A_23_P376686	2.3496295	down	NM_019602	chr6:032362725-032	BTNL2
A_23_P99604	3.0992514	down	NM_017769	chr14:031084686-03	G2E3
A_23_P59637	2.6405444	down	NM_014705	chr7:111366325-111	DOCK4
A_23_P57268	2.3950432	down	NM_001338	chr21:018938329-01	CXADR
A_24_P187056	3.0887532	down	XM_001715342	chrUn_gl000214:69734-53799	
A_33_P3421183	2.3589032	down		chr7:074615591-074615532	
A_23_P254733	2.6759961	down	NM_024629	chr4:185616396-185	MLF1IP
A_23_P420269	2.6727268	down	NM_020748	chr17:059943270-05	INTS2
A_33_P3321275	3.0532512	down	NM_170606	chr1:148888167-148	MLL3
A_24_P921366	2.4406831	down	NM_033138	chr7:134655197-134	CALD1
A_33_P3243717	2.1790758	down	NM_021045	chr10:038120602-03	ZNF248
A_24_P778906	2.854901	down	XR_078607	chrUn_gl000214:000	LOC100289026
A_24_P66125	3.9404353	down	NM_001042749	chrX:123235840-12:	STAG2
A_24_P325176	7.3745141	down	NM_015312	chr4:123283212-123	KIAA1109
A_24_P175176	2.106679	down	NM_020432	chr7:077586179-077	PHTF2
A_33_P3591761	2.4811174	down	NM_144963	chr8:124824840-124	FAM91A1
A_23_P320829	2.8083121	down	NM_152446		C14orf145
A_33_P3221528	2.4351759	down	NM_004136	chr15:078793507-07	IREB2
A_33_P3649472	2.3018329	down	NR_027078	chr16:056127040-05	LOC283856
A_33_P3306452	4.5321911	down	XM_002347052	chr9:70912728-7091	LOC653510
A_33_P3232493	2.5240911	down	AK130794	chr8:066645279-066645220	
A_32_P358887	2.5735177	down	NM_003759	chr4:072437442-072	SLC4A4
A_33_P3415625	4.1349753	down	NM_032165	chr12:085518235-08	LRRIQ1
A_23_P124427	4.6073666	down	NM_012224	chr4:170315411-170	NEK1
A_24_P409126	2.352228	down	AK056071	chr13:49720040-497	FNDC3A
A_23_P213883	4.5506133	down	NM_133433	chr5:037064923-037	NIPBL
A_32_P11450	2.0061605	down	NM_014753		BMS1
A_33_P3421490	2.6323786	down	NM_015206	chr15:079764573-07	KIAA1024
A_33_P3361746	2.5475946	down	NM_014112	chr8:116424922-116	TRPS1
A_23_P110473	2.6138186	down	NM_004536	chr5:069389205-069	NAIP
A_23_P398836	2.3595996	down	NM_020784	chr14:052897879-05	TXNDC16
A_32_P150086	3.6497686	down	NR_027995	chr21:015306517-01	LOC284232
A_23_P101811	2.2910914	down	NM_021030	chr19:019821542-01	ZNF14
A_23_P63681	2.3886972	down	NM_004969		IDE
A_23_P203255	2.4651185	down	NM_006595	chrX:115238407-11:	API5
A_33_P3222630	2.0527124	down	NM_001077528	chr8:101146035-101	FBXO43

Homo sapiens neural	GO:0005515|GO:000	NM_001144967	Hs.185677	23327	349.02798
Homo sapiens KIAA	GO:0005515	NM_020802	Hs.156352	57562	11.3279705
Homo sapiens myelo	GO:0006355|GO:000	NM_005933	Hs.258855	4297	65.94588
Homo sapiens RUN a	GO:0008270|GO:000	NM_001042417	Hs.653144	55680	76.07852
Homo sapiens superk	GO:0005515|GO:000	NM_015360	Hs.274531	23517	677.7981
					6.0311074
Homo sapiens cDNA FLJ38840 fis, clone MESAN2003490 [AK			Hs.650460	653394	10.293868
Homo sapiens neurex	GO:0005515|GO:001	NM_004796	Hs.368307	9369	6.9836354
Homo sapiens optic a	GO:0000287|GO:000	NM_130837	Hs.594504	4976	411.31454
Homo sapiens mRNA; cDNA DKFZp686N0244 (from clone DK			Hs.27371	157562	22.02647
Homo sapiens bromo	GO:0005515|GO:000	NM_013448	Hs.509140	11177	417.6109
Homo sapiens protei	GO:0005515|GO:000	NM_206907	Hs.43322	5562	303.53116
Homo sapiens mitoge	GO:0005515|GO:000	NM_198794	Hs.130491	11183	573.91486
Homo sapiens TAF2	GO:0005515|GO:001	NM_003184	Hs.122752	6873	824.5236
hz67c08.x1 NCI_CGAP_Lu24 Homo sapiens cDNA clone IMA			Hs.673861	677836	4.9999995
Homo sapiens neurol	GO:0016080|GO:003	NM_014932	Hs.478289	22871	48.510605
Homo sapiens solute	GO:0016020|GO:001	NM_199352	Hs.332119	387601	4.9999995
Homo sapiens LIM d	GO:0005515|GO:000	NM_005578	Hs.444362	4026	12.571389
Homo sapiens diapha	GO:0005794|GO:003	NM_007309	Hs.226483	1730	76.61616
Homo sapiens mRNA; cDNA DKFZp761C1711 (from clone DK			Hs.675490	57796	5.3600173
Homo sapiens carbar	GO:0019240|GO:004	NM_001875	Hs.149252	1373	29.720655
Homo sapiens zinc fi	GO:0005622|GO:000	NM_007139	Hs.9521	168374	186.86156
Homo sapiens breast	GO:0005515|GO:005	NM_000059	Hs.34012	675	31.474934
Homo sapiens DENN	GO:0005515|GO:000	NM_001144823	Hs.654567	10260	7.9659896
Homo sapiens butyro	GO:0016020|GO:001	NM_019602	Hs.534471	56244	81.07454
Homo sapiens G2/M-	GO:0000209|GO:000	NM_017769	Hs.509008	55632	106.407364
Homo sapiens dedica	GO:0005515|GO:000	NM_014705	Hs.654652	9732	182.11778
Homo sapiens coxsac	GO:0005515|GO:006	NM_001338	Hs.634837	1525	52.589607
Putative uncharacterized protein FLJ0031(		XM_001715342	Hs.709909		6.3521914
					4.9999995
Homo sapiens MLF1	GO:0005515|GO:000	NM_024629	Hs.575032	79682	3333.3098
Homo sapiens integra	GO:0005622|GO:000	NM_020748	Hs.279646	57508	84.06851
Homo sapiens myelo	GO:0005515|GO:000	NM_170606	Hs.647120	58508	70.310196
Homo sapiens caldes	GO:0005516|GO:000	NM_033138	Hs.490203	800	1601.8105
Homo sapiens zinc fi	GO:0005622|GO:000	NM_021045	Hs.528423	57209	32.404274
PREDICTED: Homo sapiens similar to hC		XR_078607	Hs.609146	100289026	4.9999995
Homo sapiens stroma	GO:0005515|GO:000	NM_001042749	Hs.496710	10735	459.69644
Homo sapiens KIAA	GO:0016020|GO:001	NM_015312	Hs.408142	84162	42.02796
Homo sapiens putativ	GO:0005783|GO:000	NM_020432	Hs.203965	57157	901.1457
Homo sapiens family with sequence simila		NM_144963	Hs.459174	157769	187.89116
Homo sapiens chromosome 14 open readir		NM_152446	Hs.162889	145508	22.76981
Homo sapiens iron-re	GO:0010468|GO:005	NM_004136	Hs.436031	3658	1078.859
Homo sapiens hypothetical protein LOC28		NR_027078	Hs.707433	283856	4.9999995
COBW domain-containing protein 3 (Coba		XM_002347052	Hs.635227	653510	29.386543
Homo sapiens cDNA FLJ27284 fis, clone TMS01544 [AK1307			Hs.660492		21.267763
Homo sapiens solute	GO:0006820|GO:000	NM_003759	Hs.5462	8671	19.071955
Homo sapiens leucin	GO:0005515	NM_032165	Hs.402200	84125	4.9999995
Homo sapiens NIMA	GO:0005515|GO:000	NM_012224	Hs.481181	4750	46.64317
Homo sapiens cDNA	GO:0012506|GO:0009566|GO:0016337|G		Hs.508010	22862	12.37326
Homo sapiens Nippe	GO:0007049|GO:003	NM_133433	Hs.481927	25836	192.02415
Homo sapiens BMS1	GO:0000166|GO:004	NM_014753	Hs.10848	9790	147.22258
Homo sapiens KIAA	GO:0016020|GO:001	NM_015206	Hs.301654	23251	70.76318
Homo sapiens trichor	GO:0043565|GO:001	NM_014112	Hs.657018	7227	7.8811126
Homo sapiens NLR f	GO:0005622|GO:001	NM_004536	Hs.654500	4671	108.65698
Homo sapiens thiore	GO:0045454|GO:000	NM_020784	Hs.532609	57544	59.8519
Homo sapiens ankyrin repeat domain 20 fa		NR_027995	Hs.633153	284232	14.88744
Homo sapiens zinc fi	GO:0005622|GO:000	NM_021030	Hs.659932	7561	111.0777
Homo sapiens insulir	GO:0005515|GO:003	NM_004969	Hs.500546	3416	288.26373
Homo sapiens apopto	GO:0005737|GO:001	NM_006595	Hs.435771	8539	190.16808
Homo sapiens F-box	GO:0007126|GO:001	NM_001077528	Hs.339577	286151	180.11934

895.03625	8.602514	9.664606
52.98557	3.6478577	5.6038065
220.3509	6.1701994	7.6410356
185.86105	6.385001	7.405546
1913.7053	9.544695	10.799454
32.716537	2.7813878	4.8896875
41.8372	3.519157	5.252658
17.747885	2.978558	4.016939
1834.146	8.833665	10.738102
68.29766	4.594183	5.9714346
1288.8597	8.853576	10.213652
796.4662	8.402088	9.496335
1917.6665	9.301447	10.802581
2173.9907	9.826901	10.991237
11.79574	2.3362613	3.40066
116.8881	5.721821	6.7370176
14.698168	2.3362613	3.7376213
61.2042	3.79326	5.811798
190.12723	6.3948097	7.436262
19.01085	2.6192322	4.1121855
83.1676	5.0334215	6.249402
697.12445	7.6902704	9.307437
136.95988	5.11106	6.9680676
22.469309	3.164341	4.3579006
231.96844	6.480362	7.7127953
401.28577	6.8613377	8.4932575
588.1794	7.6539726	9.054808
150.61919	5.84025	7.1003017
24.443333	2.8506224	4.477647
13.219861	2.3362613	3.5743775
9452.043	11.76612	13.186196
275.03543	6.5295296	7.947842
261.8787	6.2640343	7.8743806
4393.8003	10.748528	12.035813
84.94307	5.153696	6.2774124
15.874273	2.3362613	3.849702
2153.227	8.994458	10.972813
376.70068	5.518813	8.401361
2217.2979	9.9426365	11.017607
570.9625	7.697135	9.008125
76.55389	4.643731	6.1334343
3001.9067	10.191631	11.475657
12.925451	2.3362613	3.5390444
161.54657	5.0171103	7.197319
64.26067	4.544735	5.880499
58.482697	4.387396	5.7511377
22.903234	2.3362613	4.38414
261.67746	5.6692076	7.87315
35.3661	3.7690687	5.0030966
1059.4879	7.731772	9.917833
359.48508	7.331168	8.335605
225.42505	6.276109	7.672476
24.788097	3.149996	4.4991317
346.7247	6.894643	8.280802
168.76651	6.0260324	7.2645745
64.766365	4.0278983	5.8957033
310.08353	6.923277	8.119312
846.7627	8.327698	9.583922
574.0522	7.715372	9.017029
453.56598	7.6337805	8.671312

A_24_P365767	2.7867191	down	NM_000397	chrX:037672561-037	CYBB
A_23_P115842	3.5768113	down	NM_018237	chr10:070550998-07	CCAR1
A_33_P3223632	2.1923553	down	X69637	chr16:32782241-32782300	
A_33_P3217819	2.0090104	down	NM_057749	chr8:095894420-095	CCNE2
A_23_P204269	2.1766874	down	NM_006313	chr12:062799655-06	USP15
A_23_P359588	4.4759493	down	NM_032098	chr5:140769762-140	PCDHGB4
A_24_P497226	2.2360493	down	NM_003161	chr17:058027510-05	RPS6KB1
A_23_P343808	3.1425622	down	NM_005633	chr2:039213330-039	SOS1
A_33_P3342792	2.2226457	down		chr3:176136071-176136012	
A_24_P388786	4.5618095	down	NM_001369	chr5:013690667-013	DNAH5
A_23_P203920	2.3081326	down	NM_005086	chr12:026385695-02	SSPN
A_33_P3366824	2.2723074	down	NM_001128835	chrX:013734302-013	TRAPPC2
A_32_P114284	2.4528741	down	NM_001079526	chr2:213864595-213	IKZF2
A_33_P3642648	2.0489901	down	NM_006635	chr19:057805041-05	ZNF460
A_24_P282309	2.3944395	down	NM_133337	chr10:095162101-09	MYOF
A_33_P3375528	2.0263753	down	NM_152622	chr5:056233467-056	MIER3
A_23_P121356	2.463851	down	NM_020235	chr3:107524534-107	BBX
A_23_P301572	2.2128814	down	NM_015688	chr4:017634145-017	FAM184B
A_33_P3234010	2.7788892	down	NR_027754	chr19:045041376-04	FLJ41856
A_33_P3851513	2.1966682	down	NM_194282	chr4:083847040-083	LIN54
A_23_P2601	2.235431	down	NM_003299	chr12:104341536-10	HSP90B1
A_24_P109082	2.1224662	down	NM_020935	chr2:219315159-219	USP37
A_23_P117546	3.1213664	down	NM_006939	chr14:050584382-05	SOS2
A_24_P849801	2.1100644	down	NM_000983	chr1:006246098-006	RPL22
A_24_P289383	2.3398726	down	NM_017780	chr8:061779398-061	CHD7
A_24_P399942	2.5549286	down	NM_173694	chrX:138808858-138	ATP11C
A_23_P58862	3.7839932	down	NM_020819		FAM135A
A_33_P3238685	3.3441325	down	NM_001127208	chr4:106193920-106	TET2
A_33_P3396951	2.1282151	down	NM_004331	chr8:026252758-026	BNIP3L
A_33_P3724750	2.7779392	down	BC014063	chr3:65707579-6570	LOC151878
A_23_P89921	2.1389355	down	NM_013256	chr19:044980178-04	ZNF180
A_33_P3268129	2.8552414	down	EU250749	chr1:200343203-200343144	
A_33_P3294985	2.3466593	down	NR_024582	chrX:073289703-073	LOC554203
A_24_P16249	3.0075299	down	NM_033273	chr7:057188441-057	ZNF479
A_23_P101623	2.443836	down	NM_022103	chr19:056952634-05	ZNF667
A_24_P203964	2.0926456	down	NR_029426		LOC653391
A_23_P41948	2.3157123	down	NM_017785	chr5:169031233-169	CCDC99
A_24_P935682	4.97375	down	AY358248	chr11:95988484-959	UNQ6228
A_33_P3666346	2.8148002	down	NM_015446	chr1:247058043-247	AHCTF1
A_33_P3353502	2.1916837	down	NM_182797	chr20:009319625-00	PLCB4
A_33_P3268124	2.4640047	down	NM_018293	chr3:088193550-088	ZNF654
A_33_P3236517	2.004673	down	NM_152401	chr4:056448367-056	PDCL2
A_32_P78491	3.2208204	down	NM_004956	chr7:013931096-013	ETV1
A_24_P174775	3.4279852	down		chr5:43706388-4370	NNT
A_33_P3251332	2.8478424	down	NM_015473	chr14:031762417-03	HEATR5A
A_33_P3393456	2.0579348	down	AK127184	chr16:85893584-858	LOC100131129
A_33_P3291594	2.3019447	down	M19301	chr1:100676204-100676145	
A_24_P225468	2.0871655	down	NM_030920	chr1:150191136-150	ANP32E
A_23_P136909	2.6197928	down	NM_030763	chrX:080370173-080	HMGN5
A_33_P3372526	2.5039279	down		chr2:207606083-207606024	
A_24_P251661	2.2715998	down	NM_171828		KCNMB3
A_23_P39602	2.0597378	down	NM_147233	chr2:024980905-024	NCOA1
A_32_P95729	2.7060284	down	NM_018193	chr15:089858528-08	FANCI
A_23_P11507	2.4047718	down	NM_015534	chr1:078030621-078	ZZZ3
A_24_P264664	2.1271813	down	NM_001271	chr15:093570825-09	CHD2
A_24_P652700	4.916642	down	NM_014985	chr15:049030758-04	CEP152
A_33_P3351474	3.5884952	down	NM_001128429	chr4:095210929-095	SMARCAD1
A_33_P3369311	2.242085	down	NM_001142568	chr3:107524368-107	BBX
A_23_P309779	3.3120306	down	NM_018177	chr4:040156199-040	N4BP2
A_32_P35800	2.8503419	down	NM_015325	chr5:005489893-005	KIAA0947

Homo sapiens cytoch	GO:0045730|GO:000	NM_000397	Hs.292356	1536	4.9999995
Homo sapiens cell di	GO:0048471|GO:000	NM_018237	Hs.49853	55749	1003.94275
H.sapiens mRNA sequence (16p11.2) [X69637]			Hs.633945		6.3389187
Homo sapiens cyclin	GO:0005515|GO:000	NM_057749	Hs.567387	9134	405.03973
Homo sapiens ubiqui	GO:0005515|GO:000	NM_006313	Hs.434951	9958	73.98856
Homo sapiens protoc	GO:0016339|GO:000	NM_032098	Hs.368160	8641	4.9999995
Homo sapiens riboso	GO:0005515|GO:001	NM_003161	Hs.463642	6198	431.9235
Homo sapiens son of	GO:0005622|GO:000	NM_005633	Hs.709893	6654	13.93429
					203.8274
Homo sapiens dyneir	GO:0008150|GO:000	NM_001369	Hs.212360	1767	4.9999995
Homo sapiens sarcos	GO:0042383|GO:003	NM_005086	Hs.183428	8082	46.25129
Homo sapiens traffic	GO:0006888|GO:000	NM_001128835	Hs.592238	6399	12.19307
Homo sapiens IKAR	GO:0005622|GO:000	NM_001079526	Hs.604950	22807	21.537504
Homo sapiens zinc fi	GO:0005622|GO:000	NM_006635	Hs.99971	10794	198.18846
Homo sapiens myofe	GO:0034605|GO:000	NM_133337	Hs.602086	26509	210.55118
Homo sapiens mesod	GO:0005634|GO:000	NM_152622	Hs.657594	166968	33.420483
Homo sapiens bobby	GO:0005634|GO:000	NM_020235	Hs.124366	56987	1110.3967
Homo sapiens family with sequence simila		NM_015688	Hs.720110	27146	11.377299
Homo sapiens hypothetical LOC388550 (F		NR_027754	Hs.446909	388550	4.9999995
Homo sapiens lin-54	GO:0005694|GO:000	NM_194282	Hs.96952	132660	341.1546
Homo sapiens heat sl	GO:0042470|GO:000	NM_003299	Hs.192374	7184	21584.027
Homo sapiens ubiqui	GO:0006511|GO:000	NM_020935	Hs.166068	57695	75.94986
Homo sapiens son of	GO:0005622|GO:000	NM_006939	Hs.291533	6655	111.60977
Homo sapiens riboso	GO:0005622|GO:000	NM_000983	Hs.515329	6146	1023.92554
Homo sapiens chrom	GO:0060123|GO:006	NM_017780	Hs.20395	55636	471.98523
Homo sapiens ATPa	GO:0006754|GO:000	NM_173694	Hs.88252	286410	559.3895
Homo sapiens family with sequence simila		NM_020819	Hs.211700	57579	23.404463
Homo sapiens tet onc	GO:0007049|GO:000	NM_001127208	Hs.367639	54790	77.24267
Homo sapiens BCL2	GO:0008634|GO:004	NM_004331	Hs.131226	665	267.7904
Homo sapiens hypothetical protein LOC151878, mRNA (cDNA			Hs.680377	151878	69.99752
Homo sapiens zinc fi	GO:0005622|GO:000	NM_013256	Hs.22305	7733	291.06525
Putative uncharacterized protein C1orf98 [Source:UniProtKB/S			Hs.575858		4.9999995
Homo sapiens alanyl-tRNA synthetase dom		NR_024582	Hs.648316	554203	4.9999995
Homo sapiens zinc fi	GO:0005622|GO:000	NM_033273	Hs.616660	90827	62.58007
Homo sapiens zinc fi	GO:0005622|GO:000	NM_022103	Hs.676605	63934	20.316645
Homo sapiens glucuronidase, beta pseudog		NR_029426	Hs.652536	653391	116.26218
Homo sapiens coiled	GO:0005515|GO:000	NM_017785	Hs.368710	54908	2074.371
Homo sapiens clone DNA166629 MRSS6228 (UNQ6228) mRN			Hs.661972	100131541	25.550728
Homo sapiens AT ho	GO:0000910|GO:000	NM_015446	Hs.300887	25909	42.93253
Homo sapiens phospl	GO:0016042|GO:000	NM_182797	Hs.472101	5332	8.63981
Homo sapiens zinc fi	GO:0005622|GO:000	NM_018293	Hs.591650	55279	124.73057
Homo sapiens phosducin-like 2 (PDCL2),		NM_152401	Hs.223712	132954	4.9999995
Homo sapiens ets var	GO:0043565|GO:000	NM_004956	Hs.22634	2115	9.34955
NAD(P) transhydrog	GO:0050661|GO:0006099|GO:0008750|GO:0005739|GO:0005			23530	61.67094
Homo sapiens HEAT	GO:0005488	NM_015473	Hs.370299	25938	24.874132
Homo sapiens cDNA FLJ45249 fis, clone BRHIP2006921. [AK			Hs.640143	100131129	4.9999995
Lipoamide acyltransferase component of branched-chain alpha-k			Hs.709187		13.95133
Homo sapiens acidic	GO:0005515|GO:000	NM_030920	Hs.656466	81611	3706.7102
Homo sapiens high-n	GO:0006355|GO:000	NM_030763	Hs.282204	79366	94.77846
Putative malate dehydrogenase 1B (EC 1.1.1.-) [Source:UniProtKB/Swiss-Prot;Acc:Q5I0G3] [ENST00000					462.83554
Homo sapiens potass	GO:0005515|GO:000	NM_171828	Hs.591285	27094	16.730862
Homo sapiens nuclea	GO:0003713|GO:003	NM_147233	Hs.596314	8648	152.85304
Homo sapiens Fanco	GO:0005515|GO:000	NM_018193	Hs.513126	55215	1015.30914
Homo sapiens zinc fi	GO:0008270|GO:000	NM_015534	Hs.480506	26009	883.1498
Homo sapiens chrom	GO:0004003|GO:001	NM_001271	Hs.220864	1106	384.85013
Homo sapiens centro	GO:0005813|GO:000	NM_014985	Hs.597323	22995	99.2192
Homo sapiens SWI/S	GO:0005515|GO:000	NM_001128429	Hs.410406	56916	116.776695
Homo sapiens bobby	GO:0005634|GO:000	NM_001142568	Hs.124366	56987	937.2076
Homo sapiens NEDI	GO:0005515|GO:004	NM_018177	Hs.391463	55728	86.85141
Homo sapiens KIAA0947 (KIAA0947), m		NM_015325	Hs.449296	23379	258.79004

15.517722	2.3362613	3.8148289
4084.274	10.093925	11.932599
17.32529	2.8483238	3.9808054
995.4366	8.812648	9.819133
193.78868	6.3429804	7.4651146
24.785082	2.3362613	4.498455
1161.9221	8.900551	10.061503
52.48596	3.9375982	5.5895395
558.4847	7.823499	8.975777
25.260881	2.3362613	4.5258675
127.19252	5.6571302	6.8638563
33.658287	3.746695	4.930853
63.111656	4.5611672	5.8556404
499.21786	7.778018	8.812931
620.8389	7.875756	9.135444
81.188446	5.1943016	6.213203
3140.84	10.23464	11.535555
30.598581	3.6538239	4.79975
15.485231	2.3362613	3.8107696
924.53973	8.571301	9.706618
47580.918	14.380119	15.540672
194.03723	6.3813357	7.4670773
424.55786	6.9305153	8.572693
2506.473	10.120001	11.197288
1328.1973	9.031967	10.258397
1693.7795	9.264362	10.617645
107.34459	4.688674	6.6085835
316.35944	6.406502	8.148134
697.5504	8.219158	9.308802
235.51472	6.25878	7.732795
763.0829	8.338901	9.435794
15.875679	2.3362613	3.849874
13.152497	2.3362613	3.5668697
226.094	6.0883675	7.6769466
59.194553	4.478361	5.7675085
296.72647	6.992539	8.057867
5312.2163	11.112348	12.323804
153.06104	4.813479	7.127813
144.2219	5.5508795	7.043912
23.28112	3.27529	4.4073296
376.26117	7.098032	8.399037
11.343269	2.3362613	3.3396282
37.096275	3.3864863	5.0739145
256.642	6.0702915	7.8476524
85.30977	4.773968	6.2838373
11.615529	2.3362613	3.3774586
38.846306	3.9393473	5.1422005
8250.792	11.919101	12.980646
303.4373	6.6995993	8.089052
1391.9817	9.004747	10.32894
45.74276	4.2003493	5.384058
384.9562	7.3887563	8.431217
3157.7568	10.107985	11.544162
2480.865	9.917833	11.183733
1003.2581	8.74289	9.831833
592.4401	6.7658777	9.063551
508.31808	6.998076	8.841455
2448.4907	9.995696	11.160537
352.8844	6.57794	8.305656
908.05707	8.171379	9.682514

A_33_P3243332	2.0583856	down	NM_152432	chr11:100859541-10	FLJ32810
A_33_P3211423	3.5914888	down	BX648891	chr5:167975662-167	PANK3
A_23_P7048	2.2295802	down	NM_005750	chr4:005527140-005	C4orf6
A_24_P106794	2.2742092	down	NM_032869	chr8:110253441-110	NUDCD1
A_23_P360769	2.1529771	down	NM_002372		MAN2A1
A_23_P395595	2.5808763	down	NM_015308	chr11:047738209-04	FNBP4
A_33_P3273309	4.2204247	down	NM_001040185	chr19:053911268-05	ZNF765
A_24_P220771	2.1251166	down	NM_032217	chr4:074005746-074	ANKRD17
A_23_P250813	2.411385	down	NM_000553	chr8:031030776-031	WRN
A_23_P133470	2.0264794	down	NM_014819	chr5:108671278-108	PJA2
A_23_P70746	2.8617491	down	NM_017651		AHI1
A_33_P3424067	3.2444873	down	NM_017635	chr11:067923566-06	SUV420H1
A_33_P3268900	4.0209935	down	XM_001715099	chr6:115444402-115	LOC100129791
A_33_P3353622	2.440818	down	NM_021163	chr7:005104801-005	RBAK
A_23_P217098	6.0969115	down	NM_033305	chr9:080032127-080	VPS13A
A_33_P3288159	5.1469936	down	NM_018136	chr1:197057458-197	ASPM
A_32_P3998	2.0192408	down	NM_001004301	chr19:053996095-05	ZNF813
A_33_P3294031	3.6453585	down	NR_002728	chr11:002661862-00	KCNQ1OT1
A_33_P3252499	2.9375286	down	NM_001164496	chr3:113013644-113	WDR52
A_23_P151459	4.2752892	down	NM_015057	chr13:077619287-07	MYCBP2
A_24_P203622	2.4601366	down	NM_033407	chr1:063084458-063	DOCK7
A_32_P231086	2.6492513	down	NM_198181	chr15:083009557-08	GOLGA6L9
A_33_P3287562	2.5361077	down	NM_018844	chr7:107260832-107	BCAP29
A_23_P427217	6.0375407	down	NM_032776	chr10:064928279-06	JMJD1C
A_23_P420256	2.83246	down	NM_021925	chr2:020885301-020	C2orf43
A_24_P323967	2.6024001	down	NM_001145434	chr19:052888043-05	ZNF880
A_33_P3828101	2.0486024	down	NM_152549	chr5:114603559-114	CCDC112
A_23_P320530	2.003271	down	NM_001010880	chr19:040579524-04	ZNF780A
A_23_P429689	4.2748358	down	NM_148894	chr4:013570795-013	BOD1L
A_33_P3377719	2.0139287	down	AK123839	chr17:60083527-600	LOC100129112
A_33_P3343957	2.3644244	down	NM_015252	chr2:063272576-063	EHBP1
A_23_P115838	3.0777102	down	NM_015652	chr10:098745056-09	C10orf12
A_33_P3337034	2.4449941	down	NM_003913	chr6:004056609-004	PRPF4B
A_33_P3312246	5.9976902	down	NM_017934	chr6:079650912-079	PHIP
A_23_P212383	2.2983333	down	NM_014016	chr3:045786011-045	SACM1L
A_24_P53595	3.2401383	down	NM_016592	chr20:057415463-05	GNAS
A_33_P3414122	2.3530239	down	NM_001012756	chr19:037001663-03	ZNF260
A_33_P3216621	2.2801473	down	XM_001719288	chr16:054371169-05	LOC728792
A_23_P94397	6.139278	down	NM_005014	chr9:095177617-095	OMD
A_33_P3503937	2.0501579	down	AK094426	chr1:205861553-205861612	
A_33_P3235766	3.809324	down	NM_001076786	chr11:033001700-03	QSER1
A_33_P3420327	37.0722404	down	CA413366	chr14:20811622-208	RPPH1
A_33_P3346526	2.0026769	down		chr4:049564457-049564516	
A_33_P3645805	2.3074086	down	AL137339	chr1:120792257-120	LOC149401
A_24_P712350	4.0641284	down	NM_001821	chr1:241792606-241	CHML
A_33_P3249574	2.2918053	down	NM_004866	chr5:077776394-077	SCAMP1
A_23_P125748	3.9623491	down	NM_032441	chrX:101138913-101	ZMAT1
A_23_P154411	3.8207954	down	NM_004792	chr2:170493828-170	PPIG
A_23_P209032	2.0823766	down	NM_018443	chr19:035176592-03	ZNF302
A_23_P30069	2.9476612	down	NM_001012967	chr4:169278384-169	DDX60L
A_23_P200551	2.2780415	down	NM_032236	chr1:022005812-022	USP48
A_32_P89679	2.1032826	down	NM_001013620	chr12:038722948-03	ALG10B
A_24_P244952	4.3817829	down	NM_015092	chr16:018816853-01	SMG1
A_23_P311144	2.1375379	down	NM_144978	chr2:109473349-109	CCDC138
A_23_P66694	2.7395292	down	NM_006495	chr17:029631839-02	EVI2B
A_24_P221968	2.8710192	down	XM_001716860	chr16:053404723-05	LOC643802
A_23_P132784	2.7785438	down	NM_001013439	chr3:180694169-180	FXR1
A_23_P22364	3.4238512	down	NM_000844	chr3:007721812-007	GRM7
A_32_P221822	3.1396614	down	NM_016052	chr1:218511102-218	RRP15
A_33_P3210139	2.8941847	down	NM_015885	chr11:082896736-08	PCF11

Homo sapiens Rho-ty	GO:0005622|GO:000	NM_152432	Hs.269837	143872	45.490578
Pantothenate kinase 3	GO:0004594|GO:0005737|GO:0000166|G		Hs.388400	79646	1371.0907
Homo sapiens chrom	GO:0007399	NM_005750	Hs.177972	10141	5.804082
Homo sapiens NudC	GO:0006955	NM_032869	Hs.380291	84955	458.1729
Homo sapiens mannc	GO:0005794|GO:004	NM_002372	Hs.432822	4124	795.2825
Homo sapiens formir	GO:0005515	NM_015308	Hs.6834	23360	855.18414
Homo sapiens zinc fi	GO:0005622|GO:000	NM_001040185	Hs.720797	91661	7.9505296
Homo sapiens ankyri	GO:0030983|GO:000	NM_032217	Hs.719092	26057	20.17641
Homo sapiens Werne	GO:0008408|GO:000	NM_000553	Hs.632050	7486	172.56703
Homo sapiens praja r	GO:0005515|GO:000	NM_014819	Hs.483036	9867	474.5211
Homo sapiens Abelson helper integration s		NM_017651	Hs.386684	54806	42.46352
Homo sapiens suppre	GO:0005515|GO:000	NM_017635	Hs.632120	51111	6.597473
PREDICTED: Homo sapiens hypothetical		XM_001715099	Hs.646497	100129791	2858.4778
Homo sapiens RB-as	GO:0016564|GO:000	NM_021163	Hs.396178	57786	21.885246
Homo sapiens vacuol	GO:0005622|GO:000	NM_033305	Hs.459790	23230	124.61902
Homo sapiens asp (al	GO:0005516|GO:000	NM_018136	Hs.121028	259266	415.04242
Homo sapiens zinc fi	GO:0005622|GO:000	NM_001004301	Hs.710780	126017	283.44913
Homo sapiens KCNQ1 overlapping transcı		NR_002728		10984	30.75449
Homo sapiens WD repeat domain 52 (WD		NM_001164496	Hs.584936	55779	6.917643
Homo sapiens MYC	GO:0005515|GO:003	NM_015057	Hs.591221	23077	377.69754
Homo sapiens dedica	GO:0030424|GO:004	NM_033407	Hs.406156	85440	24.36871
Homo sapiens golgi autoantigen, golgin su		NM_198181	Hs.630181	440295	165.30609
Homo sapiens B-cell	GO:0016020|GO:000	NM_018844	Hs.303787	55973	279.80905
Homo sapiens jumon	GO:0046966|GO:000	NM_032776	Hs.413416	221037	153.74774
Homo sapiens chrom	GO:0003824	NM_021925	Hs.187823	60526	43.88925
Homo sapiens zinc fi	GO:0005622|GO:000	NM_001145434	Hs.502314	400713	74.04185
Homo sapiens coiled-coil domain containi		NM_152549	Hs.436121	153733	170.69708
Homo sapiens zinc fi	GO:0005622|GO:000	NM_001010880	Hs.115140	284323	24.511843
Homo sapiens biorie	GO:0003677	NM_148894	Hs.444517	259282	155.2862
Homo sapiens cDNA FLJ41845 fis, clone		NT2RI3003095. [AK	Hs.659741	100129112	20.079866
Homo sapiens EH do	GO:0005515|GO:000	NM_015252	Hs.271667	23301	232.38683
Homo sapiens chromosome 10 open readir		NM_015652	Hs.427927	26148	43.38664
Homo sapiens PRP4	GO:0008380|GO:000	NM_003913	Hs.159014	8899	306.68472
Homo sapiens plecks	GO:0008286|GO:000	NM_017934	Hs.511817	55023	251.67233
Homo sapiens SAC1	GO:0008150|GO:000	NM_014016	Hs.156509	22908	936.5839
Homo sapiens GNAS	GO:0005515|GO:000	NM_016592	Hs.125898	2778	4.9999995
Homo sapiens zinc fi	GO:0005622|GO:000	NM_001012756	Hs.18103	339324	363.6904
PREDICTED: Homo sapiens hypothetical		XM_001719288		728792	41.901745
Homo sapiens osteon	GO:0005515|GO:000	NM_005014	Hs.94070	4958	7.981417
Putative uncharacterized protein FLJ37107	[Source:UniProtKB/Swiss-Prot;Acc:Q8N9I1] [ENST0000414				260.21298
Homo sapiens glutamine and serine rich 1		NM_001076786	Hs.369368	79832	599.41815
UI-H-EU1-bae-h-06-0-UI.s1 NCI_CGAP_Ct1 Homo sapiens cD			Hs.613102	85495	4.9999995
					5.0453186
Homo sapiens mRNA; cDNA DKFZp434O039 (from clone DK			Hs.675489	149401	7286.127
Homo sapiens choroi	GO:0005968|GO:000	NM_001821	Hs.654545	1122	320.52164
Homo sapiens secret	GO:0005802|GO:000	NM_004866	Hs.482587	9522	49.291912
Homo sapiens zinc fi	GO:0005622|GO:000	NM_032441	Hs.496512	84460	19.357622
Homo sapiens peptid	GO:0008380|GO:001	NM_004792	Hs.470544	9360	702.99695
Homo sapiens zinc fi	GO:0005622|GO:000	NM_018443	Hs.436350	55900	239.66791
Homo sapiens DEAL	GO:0008026|GO:001	NM_001012967	Hs.535011	91351	162.02216
Homo sapiens ubiqui	GO:0006511|GO:000	NM_032236	Hs.467524	84196	750.5
Homo sapiens aspara	GO:0016758|GO:000	NM_001013620	Hs.259305	144245	73.20579
Homo sapiens SMG1	GO:0005515|GO:003	NM_015092	Hs.460179	23049	243.71365
Homo sapiens coiled-coil domain containi		NM_144978	Hs.362702	165055	414.23712
Homo sapiens ecotro	GO:0005737|GO:001	NM_006495	Hs.5509	2124	387.35825
PREDICTED: Homo sapiens similar to M-		XM_001716860	Hs.451336	643802	156.9871
Homo sapiens fragile	GO:0005515|GO:000	NM_001013439	Hs.478407	8087	1025.7893
Homo sapiens glutan	GO:0007608|GO:003	NM_000844	Hs.660131	2917	4.9999995
Homo sapiens riboso	GO:0005515	NM_016052	Hs.660109	51018	163.95876
Homo sapiens PCF11	GO:0006379|GO:000	NM_015885	Hs.128959	51585	314.9562

112.424515	5.634638	6.6761513
5481.525	10.532186	12.376768
16.306517	2.7278066	3.8845787
1257.5675	8.99106	10.176425
2041.0938	9.782272	10.888605
2583.2976	9.876001	11.243862
41.46903	3.1620994	5.2394876
51.333984	4.469386	5.556928
507.39557	7.568658	8.83852
1158.3638	9.037894	10.0568695
145.00581	5.5338583	7.0507555
26.53263	2.8995771	4.5975676
12315.92	11.564665	13.572217
63.828846	4.5853243	5.872689
923.0577	7.0962024	9.704281
2531.9617	8.846664	11.210394
701.18414	8.304317	9.31813
134.9793	5.0822043	6.948265
25.148094	2.9650395	4.5196424
1928.3099	8.715986	10.812008
71.90904	4.7470226	6.045761
534.26575	7.5063863	8.911971
874.4575	8.286302	9.628918
1108.0137	7.39605	9.990011
148.6376	5.579525	7.0815806
233.89583	6.3446636	7.7245064
428.57883	7.552163	8.586803
58.775455	4.75537	5.7577276
804.27686	7.411602	9.507471
48.57159	4.4615827	5.4715953
673.1503	8.016896	9.258385
159.86272	5.562598	7.1844554
925.46735	8.419039	9.70887
1807.0632	8.131865	10.716272
2502.784	9.994839	11.195427
17.92992	2.3362613	4.0323167
1046.4642	8.659744	9.89426
114.3112	5.5134597	6.7025867
59.998783	3.166861	5.78493
655.4632	8.178004	9.213739
2681.2783	9.367926	11.297461
205.94106	2.3362613	7.5485287
12.85935	2.5297456	3.5316753
17143.266	12.8643465	14.07062
1570.689	8.479923	10.502869
133.93619	5.741169	6.9376535
92.4259	4.408856	6.395212
3143.8916	9.604105	11.537978
614.5326	8.059383	9.117614
581.4817	7.4749823	9.034553
2035.389	9.698185	10.885979
184.80165	6.3259673	7.39861
1291.1879	8.084955	10.216473
1073.581	8.843723	9.939673
1280.9454	8.750797	10.204725
549.1114	7.428608	8.950171
3280.3892	10.124355	11.598684
19.003513	2.3362613	4.1118813
624.7681	7.491928	9.142537
1105.7301	8.453618	9.986775

A_23_P354805	2.7710236	down	NM_007249	chr13:074260392-07 KLF12
A_33_P3321055	2.4785035	down	BC035796	chr13:24168423-241 TNFRSF19
A_24_P942694	2.918425	down	NM_018017	chr10:115882172-11 C10orf118
A_24_P194688	2.3374111	down	NM_181723	chr8:016979882-016 EFHA2
A_24_P187921	2.2382854	down	NM_024641	chr6:096034591-096 MANEA
A_23_P303718	6.9928487	down	NM_015548	chr6:056323594-056 DST
A_23_P317347	3.5297901	down	NM_052911	chr18:019109806-01 ESCO1
A_23_P88119	2.2555074	down	NM_006644	chr13:031710855-03 HSPH1
A_23_P212595	2.0185053	down	NM_014703	chr3:051433957-051 VPRBP
A_33_P3390580	2.8891468	down	NM_014695	chr17:016608610-01 CCDC144A
A_33_P3370615	4.5609729	down		chr2:36759042-36758983
A_23_P37654	2.6531587	down	NM_001164273	chr15:042061855-04 MGA
A_33_P3238177	2.6673109	down	NM_001007094	chr10:038412110-03 ZNF37A
A_23_P259586	2.6403131	down	NM_003318	chr6:080749430-080 TTK
A_24_P101047	2.3674573	down	NM_020931	chr6:056919360-056 KIAA1586
A_23_P394323	4.1431579	down	AK095745	chr15:38775061-387 FAM98B
A_24_P912372	2.2308698	down	NM_015038	chr1:039881763-039 KIAA0754
A_33_P3333712	2.8986007	down	NM_017988	chr2:202275326-202 SCYL2
A_33_P3344399	2.2656874	down	NM_017691	chr15:071341786-07 LRRC49
A_23_P212792	2.8318434	down	NM_025009	chr4:056899111-056 CEP135
A_32_P375286	2.2921615	down	NM_173658	chr3:044636619-044 ZNF660
A_33_P3382105	3.7159961	down		chr3:032305943-032306002
A_33_P3313825	3.9767294	down		chr3:30703057-30703116
A_23_P211136	4.2460172	down	NM_018963	chr21:040558504-04 BRWD1
A_33_P3364869	2.4544805	down		chr7:105900620-105900561
A_33_P3376454	2.0401751	down	AY358688	chr5:14342798-1434 UNQ1870
A_32_P524014	4.8892257	down	NM_007124	chr6:145173938-145 UTRN
A_23_P11652	2.2440114	down	NM_003368	chr1:062917270-062 USP1
A_23_P318581	2.2086181	down	NM_020827	chr4:186081462-186 KIAA1430
A_24_P291973	2.4464351	down	NM_001011553	chr7:035913298-035 SEPT7
A_23_P132956	5.2988662	down	NM_004181	chr4:041270340-041 UCHL1
A_24_P929322	2.9629344	down	NR_024413	chr3:187898304-187 FLJ42393
A_23_P38457	2.2212627	down	NM_020791	chr17:027871269-02 TAOK1
A_23_P346048	2.7983511	down	NR_002824	chr15:023282829-02 HERC2P2
A_33_P3306973	3.1668736	down		chr12:007590340-007590281
A_24_P402836	2.5223663	down	NM_003441	chr4:000367324-000 ZNF141
A_33_P3259620	3.7729291	down	NM_014639	chr5:094800285-094 TTC37
A_33_P3212092	2.0299206	down	NM_145341	chr10:112659602-11 PDCD4
A_33_P3234859	7.0522993	down	NM_007124	chr6:144795835-144 UTRN
A_23_P48029	3.7531639	down	NM_016184	chr12:008290933-00 CLEC4A
A_32_P43812	2.0679295	down	NM_001040402	chr4:052782383-052 DCUN1D4
A_23_P56938	3.0147555	down	NM_002908	chr2:061149596-061 REL
A_32_P86245	2.9504567	down	NM_018100	chr6:052360211-052 EFHC1
A_33_P3264524	4.0164725	down		chrY:025012909-025012850
A_33_P3277110	2.5196337	down	NM_006933	chr21:035478440-03 SLC5A3
A_24_P282762	2.0057884	down	NM_012290	chr2:171863068-171 TLK1
A_23_P90845	2.4995805	down	NM_022173	chr2:070439815-070 TIA1
A_33_P3231908	2.3083417	down	NM_006236	chr2:105473077-105 POU3F3
A_23_P89710	2.7956292	down	NM_032142	chr18:013124695-01 CEP192
A_23_P93629	2.7000236	down	NM_015905	chr7:138270224-138 TRIM24
A_23_P156061	2.2787305	down	NM_005575	chr5:096362390-096 LNPEP
A_33_P3243897	2.4012407	down	NM_024813	chr1:092853630-092 RPAP2
A_23_P99693	2.5001303	down	NM_014950	chr14:065000010-06 ZBTB1
A_32_P178966	2.2005273	down	NM_001100829	chr6:011583573-011 TMEM170B
A_23_P141651	2.0401846	down	NM_006311	chr17:015942883-01 NCOR1
A_23_P214168	3.0227214	down	NM_004370	chr6:075794348-075 COL12A1
A_24_P693321	2.3032484	down	NR_024456	chr16:021444288-02 LOC100190986
A_23_P211056	2.0945404	down	NM_013329	C21orf66
A_32_P46191	2.7504049	down	NM_001159522	chr7:063538596-063 ZNF727
A_32_P141488	2.0374557	down		chr3:125628716-125628775

Homo sapiens Krupp	GO:0005622|GO:000	NM_007249	Hs.373857	11278	76.53021
Homo sapiens tumor	GO:0005739|GO:0005515|GO:0005031|G		Hs.149168	55504	19.77323
Homo sapiens chromosome 10 open readir		NM_018017	Hs.159066	55088	42.360714
Homo sapiens EF-ha	GO:0016020|GO:000	NM_181723	Hs.403594	286097	60.380802
Homo sapiens manno	GO:0005794|GO:001	NM_024641	Hs.533323	79694	48.170704
Homo sapiens dystor	GO:0005515|GO:003	NM_015548	Hs.604915	667	148.43695
Homo sapiens establi	GO:0003684|GO:000	NM_052911	Hs.464733	114799	61.335182
Homo sapiens heat sl	GO:0006986|GO:000	NM_006644	Hs.36927	10808	3926.4521
Homo sapiens Vpr (I	GO:0005737|GO:000	NM_014703	Hs.716623	9730	44.646492
Homo sapiens coiled-coil domain containi		NM_014695	Hs.721149	9720	76.04687
Putative uncharacterized protein ENSP00000385370 [Source:UniProtKB/TrEMBL;Acc:B5MCU5] [ENST					5.7561235
Homo sapiens MAX	GO:0006355|GO:000	NM_001164273	Hs.187569	23269	838.83936
Homo sapiens zinc fi	GO:0005622|GO:000	NM_001007094	Hs.292575	7587	323.84805
Homo sapiens TTK p	GO:0005515|GO:000	NM_003318	Hs.169840	7272	895.9467
Homo sapiens KIAA1586 (KIAA1586), m		NM_020931	Hs.709521	57691	65.673134
Protein FAM98B [S	GO:0005515		Hs.6799	283742	10.53192
Homo sapiens KIAA0754 (KIAA0754), m		NM_015038	Hs.658760	643314	27.15351
Homo sapiens SCY1	GO:0048471|GO:000	NM_017988	Hs.506481	55681	31.282507
Homo sapiens leucin	GO:0005515|GO:000	NM_017691	Hs.12692	54839	73.82051
Homo sapiens centro	GO:0010457|GO:000	NM_025009	Hs.518767	9662	113.477875
Homo sapiens zinc fi	GO:0005622|GO:000	NM_173658	Hs.646854	285349	18.722607
					1820.8563
TGF-beta receptor type-2 Precursor (EC 2.7.11.30)(Transforming growth factor-beta receptor type II)(TGF					13.4756
Homo sapiens bromo	GO:0005515|GO:000	NM_018963	Hs.654740	54014	15.92008
					147.90755
Homo sapiens clone DNA62312 GALI1870 (UNQ1870) mRNA			Hs.679984	100133299	129.0876
Homo sapiens utroph	GO:0005515|GO:003	NM_007124	Hs.133135	7402	419.78552
Homo sapiens ubiqui	GO:0005515|GO:000	NM_003368	Hs.35086	7398	3981.9175
Homo sapiens KIAA1430 (KIAA1430), m		NM_020827	Hs.535734	57587	848.2551
Homo sapiens septin	GO:0005515|GO:003	NM_001011553	Hs.191346	989	991.78735
Homo sapiens ubiqui	GO:0005515|GO:004	NM_004181	Hs.518731	7345	4.9999995
Homo sapiens hypothetical LOC401105 (F		NR_024413		401105	15.539578
Homo sapiens TAO 1	GO:0005737|GO:001	NM_020791	Hs.631758	57551	414.27737
Homo sapiens hect domain and RLD 2 pse		NR_002824	Hs.531509	400322	362.0406
					70.58313
Homo sapiens zinc fi	GO:0005622|GO:000	NM_003441	Hs.654355	7700	200.02834
Homo sapiens tetratri	GO:0005488	NM_014639	Hs.482868	9652	944.1876
Homo sapiens progra	GO:0005515|GO:000	NM_145341	Hs.711490	27250	63.21364
Homo sapiens utroph	GO:0005515|GO:003	NM_007124	Hs.133135	7402	4.9999995
Homo sapiens C-type	GO:0006955|GO:001	NM_016184	Hs.504657	50856	4.9999995
Homo sapiens DCN1, defective in cullin n		NM_001040402	Hs.605388	23142	563.0389
Homo sapiens v-rel r	GO:0005515|GO:004	NM_002908	Hs.631886	5966	69.25976
Homo sapiens EF-ha	GO:0019861|GO:000	NM_018100	Hs.403171	114327	4.9999995
					4.9999995
Homo sapiens solute	GO:0005215|GO:000	NM_006933	Hs.302742	6526	872.85834
Homo sapiens tousle	GO:0005515|GO:000	NM_012290	Hs.596942	9874	78.95702
Homo sapiens TIA1	GO:0042036|GO:001	NM_022173	Hs.413123	7072	267.48276
Homo sapiens POU c	GO:0016564|GO:000	NM_006236	Hs.673855	5455	7.4628434
Homo sapiens centro	GO:0005737|GO:000	NM_032142	Hs.100914	55125	958.84784
Homo sapiens tripart	GO:0016564|GO:001	NM_015905	Hs.490287	8805	1672.7631
Homo sapiens leucyl	GO:0005515|GO:000	NM_005575	Hs.656905	4012	67.498085
Homo sapiens RNA	GO:0016020|GO:001	NM_024813	Hs.444421	79871	300.67624
Homo sapiens zinc fi	GO:0005622|GO:000	NM_014950	Hs.655536	22890	34.077045
Homo sapiens transm	GO:0016020|GO:001	NM_001100829	Hs.146317	100113407	53.58397
Homo sapiens nuclea	GO:0005515|GO:000	NM_006311	Hs.462323	9611	42.797966
Homo sapiens collag	GO:0005515|GO:000	NM_004370	Hs.101302	1303	1273.7286
Homo sapiens hypothetical LOC10019098		NR_024456	Hs.676485	100190986	148.63127
Homo sapiens chrom	GO:0006355|GO:000	NM_013329	Hs.644004	94104	139.2445
Homo sapiens zinc fi	GO:0005622|GO:000	NM_001159522	Hs.640774	442319	3682.0864
					4.9999995

259.87244	6.393628	7.864047
58.434914	4.4401217	5.749591
147.87099	5.530114	7.075304
168.6104	6.0381765	7.263088
128.30243	5.712917	6.875311
1234.2034	7.3435287	10.149409
262.9825	6.062162	7.8817444
9380.139	12.003664	13.177116
107.95661	5.604366	6.6176534
268.7569	6.3836775	7.914321
33.089294	2.7161531	4.9054947
2613.1357	9.851774	11.259485
1055.7546	8.494364	9.90975
2755.2673	9.936413	11.337122
186.2569	6.1645956	7.407934
52.836205	3.549437	5.6001678
72.86508	4.905855	6.0634613
109.5456	5.1041164	6.639473
201.71208	6.3387346	7.5186834
390.97598	6.9545655	8.456307
51.31363	4.3591413	5.55585
7397.731	10.928164	12.821913
64.448555	3.8945231	5.8861055
81.23206	4.1278524	6.2139626
442.0445	7.3377743	8.633192
321.3301	7.143508	8.172201
2426.5708	8.858761	11.148367
9491.055	12.025063	13.191143
2207.6846	9.867388	11.010532
2802.9036	10.075596	11.366277
29.494291	2.3362613	4.741945
55.1979	4.0942683	5.661295
1112.1075	8.843937	9.995317
1223.8287	8.651781	10.136358
271.9104	6.2701845	7.9332438
618.4828	7.7936172	9.128395
4048.2288	10.004301	11.919986
153.00912	6.1052027	7.126626
39.132584	2.3362613	5.154355
20.822084	2.3362613	4.2443686
1384.9539	9.273909	10.322096
254.23332	6.243372	7.835413
16.45179	2.3362613	3.8971996
22.22179	2.3362613	4.3421903
2570.2449	9.904863	11.238077
191.07469	6.4406443	7.4448137
819.2352	8.21747	9.539156
21.350172	3.0739536	4.2808104
3077.2039	10.028147	11.51132
5018.1475	10.809511	12.242483
184.26973	6.2060847	7.394315
888.3931	8.389994	9.653774
102.83808	5.224392	6.5463953
140.3548	5.8678617	7.005711
104.76007	5.544243	6.5729427
4350.3047	10.425163	12.021011
417.12534	7.345574	8.549244
356.4277	7.2556763	8.32231
10700.745	11.907524	13.367168
11.51033	2.3362613	3.36303

A_23_P205098	2.9654822	down	NM_015032	chr13:033351525-03 PDS5B
A_23_P78458	2.0899972	down	NM_021632	chr19:052467706-05 ZNF350
A_33_P3372640	2.0155249	down	XR_040663	chr12:133696638-13 hCG_1646157
A_33_P3599591	2.6236111	down	NM_002581	chr9:119164007-119 PAPPA
A_32_P309404	2.1944331	down	NM_021977	chr6:160875313-160 SLC22A3
A_23_P25873	2.8270884	down	NM_007086	chr14:055408024-05 WDHD1
A_23_P155969	2.6557052	down	NM_014264	chr4:128816182-128 PLK4
A_32_P352697	2.007483	down	NM_001031732	chr4:069176348-069 YTHDC1
A_24_P181422	2.219162	down	NM_172097	chr15:043931908-04 CATSPER2
A_24_P99046	2.0726136	down	NM_015000	chr12:027478395-02 STK38L
A_33_P3217230	4.0496331	down	NM_003430	chr19:023540740-02 ZNF91
A_33_P3319134	2.0905211	down	AF289610	chr2:91833775-91833716
A_32_P142881	2.0082056	down	NM_015092	SMG1
A_33_P3287567	2.2952082	down	NM_018844	chr7:107263682-107 BCAP29
A_33_P3363537	2.2472942	down		chr7_gl000195_random:000059862-00005
A_23_P150950	2.6661926	down	NM_144982	chr12:072003855-07 ZFC3H1
A_33_P3245188	4.4636998	down		chr12:069934484-069934543
A_33_P3374833	2.3245702	down	NM_012388	chr15:045901829-04 PLDN
A_33_P3339375	2.4471923	down	NM_001039841	chr15:030927761-03 ARHGAP11B
A_23_P385427	2.4641242	down	XM_002343622	chr19:19946094-19946153
A_23_P211047	2.7888841	down	NM_206866	chr21:030717980-03 BACH1
A_24_P166663	2.8283771	down	NM_001259	chr7:092234502-092 CDK6
A_33_P3244998	3.8555215	down	NM_001164436	chr6:086604773-086 TMEM212
A_24_P170234	2.0275922	down	NM_198085	chr7:122342035-122 RNF148
A_23_P218637	3.2094543	down	NM_005054	chr2:107020331-107 RGPD5
A_33_P3401452	2.8566942	down	NM_032221	chr20:040068700-04 CHD6
A_23_P115872	2.2514009	down	NM_018131	chr10:095288754-09 CEP55
A_33_P3313680	4.3480083	down	NM_004080	chr7:014184734-014 DGKB
A_23_P47058	2.1335924	down	NM_022034	chr10:124591875-12 CUZD1
A_33_P3274930	2.1979273	down		chr17:15138596-15138537
A_33_P3285809	2.0142182	down	NM_001166417	chr1:085116133-085 SSX2IP
A_33_P3230876	2.037169	down	NM_001004127	chr13:052603719-05 ALG11
A_24_P90878	2.20189	down	NM_017736	chr16:020745705-02 THUMPD1
A_33_P3275487	2.1029247	down	NM_144682	chr17:033762291-03 SLFN13
A_24_P161764	2.4312226	down	BG107090	chr14:106471482-106471423
A_32_P21579	3.9528913	down	NM_004412	chr10:017185471-01 TRDMT1
A_23_P305210	3.3490472	down	NM_178558	chr7:063980328-063 ZNF680
A_24_P198820	2.1457426	down	NM_016507	chr17:037690454-03 CRKRS
A_24_P13041	3.3155762	down	NM_145307	chr10:063953284-06 RTKN2
A_23_P85903	2.3401052	down	NM_003268	chr1:223284126-223 TLR5
A_32_P514599	2.276302	down		chr3:036809424-036809365
A_33_P3354394	2.0830544	down	NM_198698	chr21:046074190-04 KRTAP12-4
A_33_P3311917	2.335441	down		chr8:007180352-007180293
A_24_P101812	3.7649484	down	XM_001715424	chr12:106565918-10 LOC341378
A_33_P3256083	2.354756	down		chr7:056752846-056752787
A_23_P166663	2.3685941	down	NM_012096	chr3:057306405-057 APPL1
A_33_P3230548	4.964791	down	NM_014875	chr1:200521383-200 KIF14
A_23_P373799	3.0981958	down	NM_020943	chr2:180810231-180 CWC22
A_23_P379327	2.5298485	down	NM_001040450	chr15:059149158-05 FAM63B
A_23_P25503	2.2967375	down	NM_001079673	chr13:049783462-04 FNDC3A
A_33_P3258274	2.0035062	down		chr2:188349577-188349518
A_33_P3423401	2.2682626	down	NM_206894	chr19:037308486-03 ZNF790
A_23_P18465	2.6745237	down	NM_002913	chr4:039290134-039 RFC1
A_24_P277155	2.4067712	down	NM_003071	chr3:148748450-148 HLTF
A_24_P940666	2.44002	down	NM_004774	chr17:037561073-03 MED1
A_24_P212072	2.2536102	down	NM_032290	chr5:093966300-093 ANKRD32
A_33_P3255627	2.5929793	down	NM_001142579	chr19:040578823-04 ZNF780A
A_23_P117971	2.0355773	down	XM_002344092	chr16:058588342-05 LOC100287593
A_23_P151634	2.7516844	down	NM_007192	chr14:021820393-02 SUPT16H
A_33_P3647427	2.4614093	down	X52357	chr17:15578835-155 ZNF29

Homo sapiens PDS5,	GO:0007067|GO:00(	NM_015032	Hs.646051	23047	149.24803
Homo sapiens zinc fi	GO:0005515|GO:001	NM_021632	Hs.407694	59348	211.93584
PREDICTED: Homo sapiens hCG164615		XR_040663	Hs.614816	440122	4.9999995
Homo sapiens pregna	GO:0007565|GO:001	NM_002581	Hs.643599	5069	186.18268
Homo sapiens solute	GO:0005515|GO:00(	NM_021977	Hs.567337	6581	9.717747
Homo sapiens WD re	GO:0005737|GO:00(	NM_007086	Hs.385998	11169	655.18115
Homo sapiens polo-li	GO:0005515|GO:00(	NM_014264	Hs.172052	10733	382.25284
Homo sapiens YTH (	GO:0008380|GO:00(	NM_001031732	Hs.175955	91746	27.800245
Homo sapiens cation	GO:0005515|GO:00(	NM_172097	Hs.662284	117155	26.463932
Homo sapiens serine/	GO:0005515|GO:00(	NM_015000	Hs.184523	23012	21.18236
Homo sapiens zinc fi	GO:0005622|GO:00(	NM_003430	Hs.654471	7644	32.430363
Homo sapiens clone pp9372 unknown mRNA. [AF289610]			Hs.675915		40.052044
Homo sapiens SMG1	GO:0005515|GO:00:	NM_015092	Hs.460179	23049	258.94287
Homo sapiens B-cell	GO:0016020|GO:00(	NM_018844	Hs.303787	55973	334.01962
9803					34.458675
Homo sapiens zinc fi	GO:0005622|GO:00(	NM_144982	Hs.527874	196441	117.95872
					5.595071
Homo sapiens pallidi	GO:0016081|GO:00(	NM_012388	Hs.7037	26258	1322.0681
Homo sapiens Rho G	GO:0005622|GO:00(	NM_001039841	Hs.659621	89839	246.04527
Putative zinc finger protein 56 (Putatibe zi		XM_002343622	Hs.721709		468.26813
Homo sapiens BTB a	GO:0043565|GO:00(	NM_206866	Hs.154276	571	369.81714
Homo sapiens cyclin-	GO:0010468|GO:00:	NM_001259	Hs.119882	1021	2136.4868
Homo sapiens transm	GO:0016020|GO:001	NM_001164436	Hs.580330	389177	8748.24
Homo sapiens ring fi	GO:0005515|GO:001	NM_198085	Hs.675191	378925	4.9999995
Homo sapiens RANE	GO:0005488|GO:004	NM_005054	Hs.469630	84220	504.35284
Homo sapiens chrom	GO:0008026|GO:00(	NM_032221	Hs.720576	84181	4.9999995
Homo sapiens centro	GO:0007067|GO:00(	NM_018131	Hs.14559	55165	5137.28
Homo sapiens diacyl	GO:0005515|GO:00(	NM_004080	Hs.567255	1607	4.9999995
Homo sapiens CUB a	GO:0006931|GO:00(	NM_022034	Hs.647182	50624	20.356052
Peripheral myelin protein 22 (PMP-22)(Growth arrest-specific protein 3)(GAS-3) [Source:UniProtKB/Swis					21.370329
Homo sapiens synovi	GO:0005515|GO:004	NM_001166417	Hs.22587	117178	495.6328
Homo sapiens aspara	GO:0005515|GO:001	NM_001004127	Hs.512963	440138	195.91315
Homo sapiens THUMP domain containing		NM_017736	Hs.460232	55623	210.25798
Homo sapiens schlaf	GO:0005622|GO:00(	NM_144682	Hs.462833	146857	22.461714
Putative uncharacterized protein ENSP00000375004 [Source:U			Hs.721648		7.0922875
Homo sapiens tRNA	GO:0016428|GO:00(	NM_004412	Hs.351665	1787	10.298889
Homo sapiens zinc fi	GO:0005622|GO:00(	NM_178558	Hs.520886	340252	37.496983
Homo sapiens Cdc2-	GO:0046777|GO:001	NM_016507	Hs.416108	51755	250.72824
Homo sapiens rhotek	GO:0030097|GO:00(	NM_145307	Hs.58559	219790	41.433628
Homo sapiens toll-lik	GO:0005515|GO:00(	NM_003268	Hs.604542	7100	4.9999995
					2272.058
Homo sapiens keratir	GO:0045095	NM_198698	Hs.689664	386684	4.9999995
					18.41344
PREDICTED: Homo sapiens hypothetical		XM_001715424		341378	16.402868
					5.0712476
Homo sapiens adapto	GO:0005515|GO:00(	NM_012096	Hs.476415	26060	1225.7086
Homo sapiens kinesi	GO:0005737|GO:00(	NM_014875	Hs.3104	9928	766.2365
Homo sapiens CWC2	GO:0008380|GO:00(	NM_020943	Hs.311363	57703	226.17427
Homo sapiens family	GO:0008150|GO:00(	NM_001040450	Hs.591122	54629	117.160706
Homo sapiens fibron	GO:0012506|GO:00(	NM_001079673	Hs.508010	22862	823.148
Tissue factor pathway inhibitor Precursor (TFPI)(Lipoprotein-associated coagulation inhibitor)(LACI)(Extr					228.38768
Homo sapiens zinc fi	GO:0005622|GO:00(	NM_206894	Hs.282067	388536	54.729027
Homo sapiens replica	GO:0005515|GO:001	NM_002913	Hs.507475	5981	472.80432
Homo sapiens helica	GO:0005515|GO:00(	NM_003071	Hs.3068	6596	1083.65
Homo sapiens media	GO:0005515|GO:00(	NM_004774	Hs.643754	5469	129.52356
Homo sapiens ankyri	GO:0005622	NM_032290	Hs.657315	84250	254.38126
Homo sapiens zinc fi	GO:0005622|GO:00(	NM_001142579	Hs.115140	284323	6.2127466
PREDICTED: Homo sapiens hypothetical		XM_002344092	Hs.654375	100287593	15779.602
Homo sapiens suppre	GO:0006260|GO:00(	NM_007192	Hs.213724	11198	437.13397
Human Kox26 mRNA for zinc finger protein, partial [X52357]			Hs.684886	7577	62.943085

536.9804	7.3495893	8.917856
548.3101	7.884061	8.947562
11.39823	2.3362613	3.3474169
597.36633	7.6832466	9.0748005
26.119995	3.4379067	4.571755
2181.504	9.497217	10.996534
1230.0381	8.733906	10.143001
67.21086	4.9404435	5.9458313
70.282135	4.862536	6.012551
52.484985	4.5379853	5.5894365
158.71982	5.155108	7.1728992
100.76751	5.455415	6.5192776
640.2319	8.172201	9.178108
944.46857	8.540464	9.739089
93.34488	5.2409716	6.4091606
383.4684	7.008682	8.423463
31.397339	2.676165	4.834405
3479.809	10.482575	11.699539
738.55646	8.098067	9.3891945
1384.1201	9.01931	10.320385
1248.0388	8.683989	10.163677
6608.8726	11.159065	12.6590395
33670.465	13.115986	15.062912
11.456209	2.3362613	3.3560288
1921.4174	9.123814	10.806142
15.887338	2.3362613	3.8506079
12003.468	12.370912	13.541735
24.10002	2.3362613	4.456616
51.886833	4.4811134	5.574398
56.153854	4.5516696	5.6878133
1202.8235	9.101131	10.111351
491.4292	7.7601433	8.786709
572.433	7.8744006	9.013143
56.48893	4.6248503	5.6972475
21.394684	3.0021935	4.2838755
49.41063	3.5195484	5.5024567
151.01013	5.360711	7.1044617
661.6671	8.126981	9.228458
164.45378	5.4973774	7.226637
13.12815	2.3362613	3.5628347
5633.419	11.239398	12.42609
11.75375	2.3362613	3.3949618
51.354755	4.334051	5.557746
73.77314	4.1715374	6.0841675
15.052578	2.536272	3.7718496
3319.1345	10.372854	11.616885
4393.9106	9.724442	12.036175
865.11847	7.9787707	9.610199
360.876	7.001733	8.340784
2225.1282	9.823611	11.023197
566.44836	7.992916	8.995443
148.1605	5.8957033	7.077291
1519.9287	9.03405	10.453332
2982.5266	10.197922	11.465021
385.54907	7.146787	8.43368
702.35126	8.148134	9.320372
20.147755	2.8224573	4.197068
31960.844	13.958423	14.983861
1442.9994	8.922564	10.382879
184.77832	6.098498	7.3979826

A_33_P3390576	2.5760222	down	NM_005933	chr11:118362577-11	MLL
A_23_P430201	3.4585972	down	NM_152446	chr14:080963137-08	C14orf145
A_33_P3271530	2.2869118	down	NM_024989	chr2:197757965-197	PGAP1
A_33_P3259443	2.192258	down	XR_018174	chr15:76079262-76079321	
A_33_P3285235	4.2950627	down	NM_001136156	chr19:032878432-03	ZNF507
A_23_P251785	2.6240023	down	NM_024561	chr13:041951067-04	NARG1L
A_23_P60079	2.1695963	down	NM_001147	chr8:006360349-006	ANGPT2
A_24_P337058	2.3478257	down	NM_020307		CCNL1
A_33_P3235690	4.2456643	down	NM_001081550	chrX:122748019-122	THOC2
A_33_P3522511	4.3802311	down	AB007954	chr1:61596906-6159	KIAA0485
A_33_P3379841	2.5383612	down	NR_026928	chr13:033078702-03	CG030
A_23_P82990	3.6563501	down	NM_033014	chr9:095146439-095	OGN
A_32_P76853	2.6052256	down	XM_001126407	chr15:23266738-23266797	
A_33_P3241596	2.1383026	down		chrX:003819762-003819703	
A_23_P202587	2.0769435	down	NM_018330	chr10:118644665-11	KIAA1598
A_23_P215051	2.1023021	down	NM_018479	chr6:127609954-127	ECHDC1
A_24_P360078	3.1703223	down	NM_006726	chr4:151186250-151	LRBA
A_33_P3345743	2.7733375	down	NR_003242	chr1:148573523-148	C1orf152
A_24_P312325	2.9280768	down	AJ312027	chr8:10980986-10980927	
A_24_P918384	2.3187251	down	NM_022455	chr5:176726576-176	NSD1
A_33_P3251347	2.2223733	down	AL832071	chrX:30864110-30864051	
A_24_P150874	2.2331977	down	NM_006572	chr17:063005770-06	GNA13
A_33_P3358104	2.4263371	down	NM_181449	chr17:072613358-07	CD300E
A_33_P3355747	2.0586587	down		chr2:075889436-075889377	
A_32_P2392	2.6516843	down	NM_181077	chr15:032737088-03	GOLGA8A
A_33_P3244863	3.5593737	down	NM_014810	chr1:179961297-179	CEP350
A_32_P113584	4.8503323	down	NM_015021	chr6:087973262-087	ZNF292
A_23_P502797	2.1911095	down	NM_020830	chr2:224740990-224	WDFY1
A_23_P46045	3.642951	down	NM_003617	chr1:163112189-163	RGS5
A_23_P3204	2.0975933	down	NM_002748	chr15:052358055-05	MAPK6
A_24_P127928	3.1917286	down	NM_012414	chr1:220323885-220	RAB3GAP2
A_24_P412512	3.3242203	down	NM_016350	chr14:051204925-05	NIN
A_32_P203430	2.4611906	down	NM_194325	chr19:035435800-03	ZNF30
A_33_P3357049	3.4071189	down	NR_026768	chr1:046111983-046	RPS15AP10
A_24_P82032	2.3006479	down	NM_020663	chr14:063757601-06	RHOJ
A_23_P133236	2.098319	down	NM_018934	chr5:140605528-140	PCDHB14
A_23_P61171	11.6598668	down	NM_012283	chr18:077659406-07	KCNG2
A_24_P401787	2.3790263	down	NM_173353	chr12:072338117-07	TPH2
A_23_P332713	2.0329736	down	NM_015174	chr19:003804152-00	ZFR2
A_23_P315964	2.9007459	down	NM_173568	chr21:043562715-04	UMODL1
A_33_P3840630	4.4849721	down	NM_015030	chr4:048501356-048	FRYL
A_33_P3400653	2.1428116	down	AK125533	chr15:59951495-599	BNIP2
A_33_P3247933	4.4736674	down	NM_181453	chr2:109086421-109	GCC2
A_24_P723735	2.4859226	down		chr16:31613274-31613215	
A_33_P3408983	2.5005821	down	NM_001127511	chr5:112103018-112	APC
A_33_P3323392	2.2521756	down	XM_001716667	chr13:047032723-04	LOC731932
A_23_P411335	4.441928	down	NM_152524	chr2:201438513-201	SGOL2
A_33_P3369716	3.1765356	down		chr12:068825700-068825641	
A_33_P3374085	2.9652752	down	NM_001137552	chr2:238672724-238	LRRFIP1
A_32_P104478	2.5526452	down	NM_018351	chr12:095470741-09	FGD6
A_23_P41327	2.0964341	down	NM_017816	chr4:004270336-004	LYAR
A_33_P3282175	4.0777482	down		chr6:077232276-077232217	
A_33_P3218649	2.1573837	down	NM_001001995	chrX:013789216-013	GPM6B
A_23_P71855	3.0241387	down	NM_001735	chr9:123715126-123	C5
A_33_P3305973	2.1751235	down		chr12:064154387-064154328	
A_24_P333663	2.2989349	down	NM_002748	chr15:052356907-05	MAPK6
A_32_P51894	2.4504284	down	NM_031844	chr1:245014677-245	HNRNPU
A_33_P3292829	2.0732082	down	NM_017988	chr12:100717325-10	SCYL2
A_23_P25720	2.7981546	down	NM_006215	chr14:095033453-09	SERPINA4
A_33_P3412468	2.8762718	down	NM_004277	chr6:046645803-046	SLC25A27

Homo sapiens myelo	GO:0006355|GO:000	NM_005933	Hs.258855	4297	23.197803
Homo sapiens chromosome 14 open readir		NM_152446	Hs.162889	145508	106.71128
Homo sapiens post-C	GO:0042578|GO:000	NM_024989	Hs.229988	80055	44.258003
Putative uncharacterized protein ENSP000		XR_018174	Hs.635230		14.501768
Homo sapiens zinc fi	GO:0005622|GO:000	NM_001136156	Hs.205392	22847	16.622726
Homo sapiens NMD	GO:0005515|GO:000	NM_024561	Hs.512914	79612	503.3703
Homo sapiens angio	GO:0007165|GO:001	NM_001147	Hs.583870	285	6.625564
Homo sapiens cyclin	GO:0016607|GO:000	NM_020307	Hs.4859	57018	218.75568
Homo sapiens THO	GO:0008380|GO:000	NM_001081550	Hs.149991	57187	101.6394
Homo sapiens mRNA, chromosome 1 specific transcript KIAA0			Hs.604754	57235	14.371429
Homo sapiens hypothetical CG030 (CG03		NR_026928	Hs.658751	116828	30.074425
Homo sapiens osteog	GO:0005515|GO:000	NM_033014	Hs.109439	4969	12.01642
Golgin subfamily A member 8-like protein		XM_001126407	Hs.525905		71.6181
					14193.475
Homo sapiens KIAA	GO:0030424|GO:000	NM_018330	Hs.501140	57698	154.20857
Homo sapiens enoyl	GO:0008152|GO:000	NM_018479	Hs.486410	55862	1997.5936
Homo sapiens LPS-r	GO:0005515|GO:000	NM_006726	Hs.480938	987	124.67979
Homo sapiens chromosome 1 open reading		NR_003242	Hs.657186	767846	29.596367
Homo sapiens mRNA for hypothetical protein (C8ORF15) [AJ3			Hs.661517		9.005401
Homo sapiens nuclea	GO:0005515|GO:001	NM_022455	Hs.106861	64324	6.9220824
Mitogen-activated protein kinase kinase kinase 7-interacting pro			Hs.188256		6.471375
Homo sapiens guanir	GO:0005515|GO:000	NM_006572	Hs.515018	10672	677.75464
Homo sapiens CD30	GO:0006955|GO:000	NM_181449	Hs.158954	342510	4.9999995
					7.3888397
Homo sapiens golgi	GO:0005794|GO:001	NM_181077	Hs.720151	23015	23.149681
Homo sapiens centro	GO:0005813|GO:000	NM_014810	Hs.413045	9857	19.547747
Homo sapiens zinc fi	GO:0005622|GO:000	NM_015021	Hs.485892	23036	16.068937
Homo sapiens WD re	GO:0008150|GO:000	NM_020830	Hs.368359	57590	203.15247
Homo sapiens regula	GO:0008277|GO:000	NM_003617	Hs.24950	8490	28.582062
Homo sapiens mitog	GO:0007165|GO:000	NM_002748	Hs.411847	5597	1940.6024
Homo sapiens RAB3	GO:0005515|GO:000	NM_012414	Hs.654849	25782	1539.4606
Homo sapiens ninein	GO:0005515|GO:005	NM_016350	Hs.310429	51199	87.915375
Homo sapiens zinc fi	GO:0005622|GO:000	NM_194325	Hs.657402	90075	159.1682
Homo sapiens ribosomal protein S15a pseu		NR_026768		728963	8.683765
Homo sapiens ras ho	GO:0005622|GO:000	NM_020663	Hs.656339	57381	4.9999995
Homo sapiens protoc	GO:0016339|GO:000	NM_018934	Hs.658497	56122	43.777683
Homo sapiens potass	GO:0005515|GO:000	NM_012283	Hs.247905	26251	4.9999995
Homo sapiens trypto	GO:0042427|GO:000	NM_173353	Hs.376337	121278	4.9999995
Homo sapiens zinc fi	GO:0005622|GO:000	NM_015174	Hs.65750	23217	4.9999995
Homo sapiens uromo	GO:0005737|GO:000	NM_173568	Hs.242520	89766	8.100474
Homo sapiens FRY-l	GO:0005515|GO:004	NM_015030	Hs.595553	285527	373.8086
BCL2/adenovirus E1	GO:0005515|GO:0048471|GO:0005737|G		Hs.592515	663	669.4092
Homo sapiens GRIP	GO:0005737|GO:000	NM_181453	Hs.436505	9648	67.375046
Putative uncharacterized protein ZNF720P1 Fragment [Source:UniProtKB/TrEMBL;Acc:A8MY22] [ENS					21.750153
Homo sapiens adeno	GO:0008017|GO:000	NM_001127511	Hs.158932	324	281.36316
PREDICTED: Homo sapiens hypothetical		XM_001716667	Hs.539723	731932	107.889404
Homo sapiens shugo	GO:0005515|GO:000	NM_152524	Hs.655182	151246	271.23254
					4.9999995
Homo sapiens leucin	GO:0016564|GO:000	NM_001137552	Hs.471779	9208	319.853
Homo sapiens FYVE	GO:0005515|GO:000	NM_018351	Hs.506381	55785	66.90529
Homo sapiens Ly1 a	GO:0005515|GO:000	NM_017816	Hs.425427	55646	3412.2517
					4.9999995
Homo sapiens glycop	GO:0003674|GO:001	NM_001001995	Hs.495710	2824	5.599768
Homo sapiens compl	GO:0008009|GO:000	NM_001735	Hs.494997	727	35.434113
					4.9999995
Homo sapiens mitog	GO:0007165|GO:000	NM_002748	Hs.411847	5597	163.4051
Homo sapiens hetero	GO:0005515|GO:000	NM_031844	Hs.106212	3192	1421.9639
Homo sapiens SCY1	GO:0048471|GO:000	NM_017988	Hs.506481	55681	158.52312
Homo sapiens serpin	GO:0005515|GO:000	NM_006215	Hs.719893	5267	4.9999995
Homo sapiens solute	GO:0005739|GO:000	NM_004277	Hs.40510	9481	37.833977

71.545685	4.673849	6.038994
447.69064	6.864505	8.654692
120.933395	5.592128	6.7855287
38.38646	3.9941669	5.1265845
85.81629	4.1900244	6.2927036
1579.7866	9.120783	10.512552
17.796968	2.9045718	4.0219984
631.6913	7.9279327	9.159258
523.0364	6.7993937	8.885384
75.306404	3.9783	6.109307
92.300125	5.0486126	6.39251
52.782288	3.7280622	5.5984664
225.74908	6.2932887	7.674697
30340.355	13.809967	14.906433
390.40137	7.400391	8.454853
4647.332	11.055618	12.127588
482.39594	7.0972915	8.761921
99.50991	5.0263968	6.49802
32.576954	3.3348296	4.884783
19.895163	2.9656692	4.179001
17.85013	2.8733242	4.0254254
1793.0736	9.544544	10.703655
13.59162	2.3362613	3.6150413
18.820679	3.0579083	4.099613
73.51771	4.6703615	6.0772705
83.442	4.4229856	6.254609
93.95924	4.140671	6.4187546
549.1132	7.8191004	8.950762
125.63972	4.979804	6.8449116
4554.895	11.018896	12.087631
5454.066	10.693579	12.367917
358.13147	6.595024	8.32804
478.02075	7.4478416	8.747198
36.498505	3.2824693	5.0510216
12.91399	2.3362613	3.5383015
109.90402	5.575575	6.644809
64.22706	2.3362613	5.8797407
13.340151	2.3362613	3.5866325
11.482231	2.3362613	3.3598528
29.096302	3.1862886	4.7227125
2000.4651	8.698325	10.863424
1705.7616	9.528421	10.627926
367.19168	6.203336	8.364794
64.603195	4.578101	5.8918824
867.37	8.292945	9.615209
295.80386	6.8827477	8.054067
1448.893	8.237644	10.38883
17.591452	2.3362613	4.0037155
1149.846	8.477158	10.045324
205.598	6.193194	7.545187
7640.8633	11.799868	12.8678055
22.5926	2.3362613	4.364034
15.210031	2.6772215	3.7865043
128.54909	5.2813177	6.877842
12.242479	2.3362613	3.4573586
458.5744	7.4867954	8.687761
3921.7751	10.584169	11.877203
401.58322	7.442499	8.494364
15.572441	2.3362613	3.820737
130.6219	5.3742957	6.8984957

A_23_P428999	3.5127325	down	NM_006540	chr8:071024424-071	NCOA2
A_33_P3213086	2.3108224	down	BX457161	chr21:15304666-15304609	
A_23_P159390	3.1838854	down	NM_007027	chr3:133329966-133	TOPBP1
A_23_P359457	2.4137703	down	BC034225	chr17:7646539-7646598	
A_23_P424269	2.9750576	down	AK095025	chr9:98775835-98775894	
A_33_P3330453	2.2869899	down	NM_018313	chr3:052579570-052	PBRM1
A_23_P17103	2.3344287	down	NM_025244	chr2:099614532-099	TSGA10
A_33_P3341429	2.708329	down	NM_144573	chr1:078383898-078	NEXN
A_33_P3234864	2.1028113	down	NM_007124	chr6:145051508-145	UTRN
A_32_P326819	2.5215856	down	NM_007043	chr12:075892085-07	KRR1
A_24_P281801	2.0982826	down	NM_001137608	chr4:000265611-000	ZNF732
A_33_P3843358	2.4256636	down	BG944179	chr14:21865360-218	SNORD8
A_23_P154447	2.7307969	down	NM_015934	chr16:060058587-06	NOP58
A_33_P3271410	3.7849887	down	NM_024672	chr4:083840982-083	THAP9
A_23_P339480	2.0180487	down	NM_003642	chr2:172848187-172	HAT1
A_33_P3230558	2.4683509	down	NM_183387	chr14:089091442-08	EML5
A_33_P3396612	2.3414042	down		chr16:033489569-033489510	
A_24_P783008	2.3502571	down	NM_001102659		hCG_1657980
A_23_P347059	2.9240034	down	NM_173468	chr4:071853584-071	MOBKL1A
A_23_P336198	2.5770194	down	NM_138426	chr7:008128578-008	GLCCI1
A_23_P357811	2.9135781	down	NM_021038	chr3:152182837-152	MBNL1
A_24_P331560	2.1001343	down	NM_000351	chrX:007271727-007	STS
A_33_P3305243	2.0710177	down		chr9:123586467-123586408	
A_33_P3315060	2.4983181	down	CR627439	chr1:203771403-203	ZC3H11A
A_33_P3439765	2.0457162	down	NM_024824	chr14:089079266-08	ZC3H14
A_32_P178945	2.0002398	down	NM_018566	chr1:207217382-207	YOD1
A_33_P3796201	4.6412863	down	BX093265	chr17:14934977-149	CDRT7
A_24_P52004	2.5004331	down	NM_015200	chr4:039825142-039	PDS5A
A_32_P94160	3.6244211	down	NM_006252	chr1:057180117-057	PRKAA2
A_23_P129058	2.8354859	down	NM_005154	chr15:050791323-05	USP8
A_33_P3274299	2.2784674	down	NM_001080529	chr7:029945732-029	WIPF3
A_24_P54178	2.0246001	down	NM_016040	chr1:093617749-093	TMED5
A_23_P417942	2.0644135	down	NM_001024948	chr1:094019908-094	FNBP1L
A_33_P3339361	2.0284321	down	NM_199357	chr15:032928084-03	ARHGAP11A
A_33_P3239101	2.0010188	down	NM_001164465	chr15:085055940-08	GOLGA6L10
A_33_P3291831	2.503756	down	NM_018131	chr10:095279478-09	CEP55
A_24_P219114	2.9579089	down	AB020335	chr14:81938120-819	SEL1L
A_33_P3474538	3.7982567	down	NM_007138	chr19:020231608-02	ZNF90
A_33_P3640690	4.3022966	down	NM_001128128	chr10:031816261-03	ZEB1
A_33_P3321781	3.2281889	down	XR_040497	chr17:056066470-05	FLJ44342
A_33_P3221443	2.3955561	down	NM_203282	chr19:024312428-02	ZNF254
A_24_P16378	2.0849642	down	NM_152419	chr8:043014085-043	HGSNAT
A_33_P3268798	2.5722634	down	NM_182503	chr6:143744180-143	ADAT2
A_33_P3340828	2.7978986	down	XM_001723580	chr6:044445578-044	LOC100128935
A_33_P3340007	2.3874789	down	NR_003268	chr13:059104642-05	LOC220429
A_33_P3287502	2.1726097	down	NM_000251	chr2:047709947-047	MSH2
A_33_P3218178	2.2662354	down	NM_001012755	chrX:103343992-10	MCART6
A_33_P3341474	2.2932881	down	NM_001080412	chr3:141168501-141	ZBTB38
A_32_P54242	5.0694014	down	NM_153686	chr4:017845497-017	LCORL
A_32_P93149	2.084342	down	NM_004398	chr9:084701750-084	DDX10
A_33_P3279475	2.2569532	down	AF116680	chr8_gl000196_random:12472-12413	
A_23_P92727	2.7899959	down	NM_015577	chr5:034831952-034	RAI14
A_33_P3227842	2.806723	down	NM_001166006	chr1:029391669-029	EPB41
A_23_P313734	4.9842779	down	NM_033395		KIAA1731
A_23_P56298	2.5967791	down	NM_025189	chr19:021240629-02	ZNF430
A_24_P935986	3.2566033	down	NM_005504	chr12:024964511-02	BCAT1
A_32_P95914	2.9359879	down	NM_198468	chr6:097590265-097	C6orf167
A_33_P3249135	2.9714961	down	NM_001114132	chr2:204082537-204	NBEAL1
A_33_P3311750	3.2014141	down	NM_014695	chr17:018483084-01	CCDC144A
A_33_P3576797	3.6121194	down	AL110203	chrX:73457560-734	LOC158863

Homo sapiens nuclea	GO:0016564|GO:000	NM_006540	Hs.446678	10499	4.9999995
BX457161 Homo sapiens THYMUS Homo sapiens cDNA clon			Hs.721707		72.00563
Homo sapiens topois	GO:0005622|GO:000	NM_007027	Hs.593379	11073	160.6341
Dynein heavy chain 2, axonemal (Axonemal beta dynein heavy c			Hs.367649		4.9999995
Putative DNA repair and recombination protein RAD26-like (EC			Hs.432364		139.60168
Homo sapiens polybr	GO:0007507|GO:000	NM_018313	Hs.189920	55193	1210.7657
Homo sapiens testis s	GO:0043005|GO:003	NM_025244	Hs.120267	80705	19.899069
Homo sapiens nexilir	GO:0030334|GO:000	NM_144573	Hs.612385	91624	291.3642
Homo sapiens utroph	GO:0005515|GO:003	NM_007124	Hs.133135	7402	20.004732
Homo sapiens KRR1	GO:0006364|GO:000	NM_007043	Hs.645517	11103	50.782578
Homo sapiens zinc fi	GO:0005622|GO:000	NM_001137608	Hs.698668	654254	535.6382
ax47d11.x1 Proliferating Human Erythroid Cells (LCB:ax librar			Hs.680078	319103	9.487289
Homo sapiens NOP5	GO:0005515|GO:003	NM_015934	Hs.471104	51602	5167.681
Homo sapiens THAP	GO:0008270|GO:000	NM_024672	Hs.582050	79725	14.776742
Homo sapiens histon	GO:0005515|GO:000	NM_003642	Hs.632532	8520	4647.2524
Homo sapiens echinc	GO:0005737|GO:000	NM_183387	Hs.558671	161436	6.188051
					2483.2146
Homo sapiens hCG1657980 (LOC200726)		NM_001102659	Hs.144769	200726	4.9999995
Homo sapiens MOB1	GO:0005515|GO:001	NM_173468	Hs.700445	92597	443.0699
Homo sapiens glucoc	GO:0005515|GO:000	NM_138426	Hs.131673	113263	46.441727
Homo sapiens muscle	GO:0003725|GO:000	NM_021038	Hs.478000	4154	4595.816
Homo sapiens steroic	GO:0006629|GO:001	NM_000351	Hs.522578	412	12.0053005
26S proteasome non-ATPase regulatory subunit 5 (26S proteasome subunit S5B)(26S protease subunit S5 b					6.4474583
Homo sapiens mRNA; cDNA DKFZp781G2455 (from clone DF			Hs.532399	9877	40.507698
Homo sapiens zinc fi	GO:0003723|GO:000	NM_024824	Hs.325846	79882	8368.095
Homo sapiens YOD1	GO:0005622|GO:001	NM_018566	Hs.567533	55432	568.8544
BX093265 Soares_NFL_T_GBC_S1 Homo sapiens cDNA clon			Hs.147654	94150	4.9999995
Homo sapiens PDS5,	GO:0007067|GO:000	NM_015200	Hs.331431	23244	342.19446
Homo sapiens protei	GO:0006695|GO:000	NM_006252	Hs.437039	5563	5.123829
Homo sapiens ubiqui	GO:0005622|GO:000	NM_005154	Hs.644563	9101	261.89514
Homo sapiens WAS/	GO:0017124|GO:000	NM_001080529	Hs.709280	644150	4.9999995
Homo sapiens transm	GO:0005793|GO:001	NM_016040	Hs.482873	50999	1485.3701
Homo sapiens formir	GO:0005737|GO:000	NM_001024948	Hs.134060	54874	918.64386
Homo sapiens Rho G	GO:0005622|GO:000	NM_199357	Hs.591130	9824	337.7446
Homo sapiens golgi autoantigen, golgin su		NM_001164465	Hs.630181	647042	13.44457
Homo sapiens centro	GO:0007067|GO:000	NM_018131	Hs.14559	55165	2528.75
Protein sel-1 homolo	GO:0005515|GO:0016020|GO:0007219|G		Hs.181300	6400	12.475869
Homo sapiens zinc fi	GO:0005622|GO:000	NM_007138	Hs.55304	7643	31.241207
Homo sapiens zinc fi	GO:0007389|GO:000	NM_001128128	Hs.124503	6935	296.98648
PREDICTED: Homo sapiens hypothetical		XR_040497	Hs.710026	645460	1052.1708
Homo sapiens zinc fi	GO:0005622|GO:000	NM_203282	Hs.434406	9534	58.62095
Homo sapiens hepara	GO:0015019|GO:001	NM_152419	Hs.600384	138050	25.616972
Homo sapiens adeno	GO:0016787|GO:000	NM_182503	Hs.709561	134637	19.796398
PREDICTED: Homo sapiens similar to hC		XM_001723580		100128935	6.1274805
Homo sapiens CTAGE family, member 5 p		NR_003268	Hs.646637	220429	16.9507
Homo sapiens mutS l	GO:0019724|GO:001	NM_000251	Hs.597656	4436	3942.1501
Homo sapiens mitocl	GO:0005739|GO:001	NM_001012755	Hs.660022	401612	14.693559
Homo sapiens zinc fi	GO:0005622|GO:000	NM_001080412	Hs.518301	253461	20.86266
Homo sapiens ligand	GO:0006366|GO:000	NM_153686	Hs.446201	254251	7.672708
Homo sapiens DEAD	GO:0008026|GO:000	NM_004398	Hs.591931	1662	202.67273
PRO2007 [Source:UniProtKB/TrEMBL;Acc:Q9P1F5] [ENST00000391554]					26.389627
Homo sapiens retinoi	GO:0005739|GO:000	NM_015577	Hs.431400	26064	389.35834
Homo sapiens erythr	GO:0008091|GO:000	NM_001166006		2035	4.9999995
Homo sapiens KIAA1731 (KIAA1731), m		NM_033395	Hs.458418	85459	389.37634
Homo sapiens zinc fi	GO:0005622|GO:000	NM_025189	Hs.466289	80264	48.28268
Homo sapiens branch	GO:0005737|GO:000	NM_005504	Hs.438993	586	2506.988
Homo sapiens chrom	GO:0005515	NM_198468	Hs.444292	253714	523.5275
Homo sapiens neurobeachin-like 1 (NBEA		NM_001114132	Hs.648846	65065	7.3989143
Homo sapiens coiled-coil domain containi		NM_014695	Hs.721149	9720	16.28609
Homo sapiens mRNA; cDNA DKFZp586J1922 (from clone DK			Hs.138411	158863	7.4897027

19.489473	2.3362613	4.148855
200.55742	6.3023806	7.510787
619.4247	7.4593906	9.130179
13.51474	2.3362613	3.6075497
505.73868	7.2602234	8.833141
3166.3665	10.354705	11.548155
55.622013	4.449937	5.6730065
971.9035	8.341211	9.778614
50.368404	4.456616	5.5289354
152.65805	5.788286	7.1226172
1344.9521	9.206785	10.275994
28.326838	3.4052505	4.68363
14712.553	12.378879	13.828201
66.855415	4.0176506	5.9379396
9793.827	12.231051	13.244012
19.101938	2.8177805	4.121328
6309.3877	11.365013	12.592387
13.171499	2.3362613	3.5690799
1553.6832	8.940903	10.488848
142.84688	5.6634016	7.029105
13916.302	12.213781	13.756573
30.527557	3.7261014	4.796583
16.64473	2.8679311	3.918271
121.34103	5.4704013	6.7913585
17327.756	13.055758	14.088364
1358.6184	9.287863	10.288036
25.718937	2.3362613	4.550786
1048.5726	8.576233	9.898411
23.382853	2.5549364	4.412687
914.11035	8.187	9.690596
12.806299	2.3362613	3.524325
3426.4011	10.646963	11.6646
2207.8713	9.965181	11.010913
843.3992	8.555952	9.576317
32.73754	3.8907213	4.891456
6908.6294	11.392773	12.716867
44.54093	3.7801118	5.3446894
142.66588	5.1019616	7.027299
1540.9623	8.372	10.477107
3854.9275	10.159978	11.850703
167.76427	5.9956694	7.25603
64.097015	4.8174934	5.877516
60.892982	4.441928	5.8049664
21.433008	2.8019683	4.286312
48.665836	4.220174	5.475662
9075.224	12.009266	13.128695
40.059284	4.0103383	5.190636
57.081123	4.51429	5.7117076
48.110558	3.1152673	5.4570827
519.536	7.815447	8.875039
71.17866	4.8569345	6.031311
1309.5449	8.757385	10.237648
15.63122	2.3362613	3.825148
2300.437	8.757828	11.0752125
149.8481	5.7157984	7.0925217
8815.976	11.381943	13.085311
1824.3901	9.177042	10.730888
27.225151	3.060944	4.6321335
62.401615	4.1612377	5.839947
33.700565	3.0802934	4.933139

A_23_P2203	2.1739082	down	NM_016281	chr12:118610340-11	TAOK3
A_33_P3230254	2.9483525	down	NM_022346	chr4:017846426-017	NCAPG
A_33_P3503537	2.5741641	down	AK091571	chr2:217081345-217	LOC285178
A_23_P213085	2.5297521	down	NM_001029998	chr4:147175938-147	SLC10A7
A_33_P3217218	2.1831307	down	NM_018361	chr8:006618891-006	AGPAT5
A_24_P476086	2.1791004	down		chr6:117056632-117	KPNA5
A_33_P3339231	2.140851	down	NM_173569	chr7:138992885-138	UBN2
A_33_P3251205	4.3010285	down	XM_002345474	chr22:23162022-23162081	
A_23_P169278	2.6079858	down	NM_015239	chr9:088161772-088	AGTPBP1
A_24_P102920	3.2220105	down		chr2:128521238-128	WDR33
A_33_P3267651	2.8142744	down	NM_014648	chr3:108351859-108	DZIP3
A_23_P384748	2.0145486	down	NM_172069	chr2:043994693-043	PLEKHH2
A_23_P5679	2.0427873	down	NM_032545	chr2:131357031-131	CFC1
A_33_P3319943	6.0115687	down	NM_000489	chrX:076872141-076	ATRX
A_33_P3299796	2.3948105	down	AK126926	chr9:139997703-139997762	
A_23_P166408	2.1431739	down	NM_020530	chr22:030659028-03	OSM
A_33_P3257568	2.2464811	down	AK097636	chr9:123912863-123912922	
A_23_P54006	2.6612287	down	NM_015382	chr14:031569500-03	HECTD1
A_33_P3268868	2.4079651	down		chr1:143738162-143738103	
A_32_P138617	3.2431802	down	NM_001009899	chr3:113367573-113	KIAA2018
A_33_P3423590	2.2857093	down	AK126260	chr3:119553782-119	LOC100129275
A_33_P3264771	2.1290386	down	NM_001134462	chr2:073437932-073	NOTO
A_23_P251268	2.0044309	down	NR_026829	chr3:010327031-010	C3orf42
A_33_P3319940	4.9446795	down	NM_000489	chrX:076944381-076	ATRX
A_23_P99292	2.0941169	down	NM_006479	chr12:004668182-00	RAD51AP1
A_23_P355447	2.074993	down	NM_174976	chr14:077597916-07	ZDHHC22
A_23_P127195	2.212461	down	NM_003675	chr10:013672451-01	PRPF18
A_23_P82975	4.3452368	down	NM_181661		VPS13B
A_24_P190541	4.0603852	down	NM_018963	chr21:040568557-04	BRWD1
A_33_P3416142	4.1672741	down	XM_001722941	chr1:198868184-198	LOC100131234
A_32_P74366	2.7846683	down	CR936659	chr8:67540976-6754	VCPIP1
A_23_P48835	2.3134429	down	NM_138555	chr15:069740524-06	KIF23
A_23_P149189	2.7101077	down	NM_021186		ZP4
A_24_P918843	3.9163989	down	NM_194301		RALGAPA1
A_23_P211007	2.8174414	down	NM_003489	chr21:016333963-01	NRIP1
A_23_P319133	2.8141476	down	NM_018981	chr2:183643075-183	DNAJC10
A_23_P18649	2.6769745	down	NM_024582	chr4:126413229-126	FAT4
A_23_P212728	2.2863591	down	NM_018309	chr3:100043741-100	TBC1D23
A_33_P3274397	2.2853577	down	NM_001145414	chrX:085224229-085	CHM
A_33_P3361811	2.8049829	down	NM_024561	chr13:041936235-04	NARG1L
A_33_P3526315	2.5646446	down	BC005372	chr3:33590679-3359	MGC12488
A_33_P3304293	2.902395	down	NM_001042751	chrX:123159708-123	STAG2
A_33_P3270657	3.7851409	down	NM_198947	chr11:058894801-05	FAM111B
A_33_P3308949	2.0793183	down	NM_001918	chr1:100652543-100	DBT
A_23_P26124	2.1622254	down	NM_134260	chr15:060789716-06	RORA
A_33_P3408212	2.6911522	down	NM_003299	chr15:099799844-09	HSP90B1
A_23_P70201	3.6634324	down	NM_001270	chr5:098191985-098	CHD1
A_33_P3234814	2.9179163	down			
A_33_P3221114	3.9574296	down	NM_144720	chr4:006055837-006	JAKMIP1
A_32_P127350	2.980829	down	NR_024384	chr5:087960601-087	LOC645323
A_23_P434430	2.3712767	down	NM_152262	chr19:011979911-01	ZNF439
A_33_P3243997	2.0999791	down	NM_014614	chr2:054091284-054	PSME4
A_33_P3277173	3.3876389	down		chr7:149496111-149496170	
A_33_P3382959	3.758339	down	NM_012115	chr6:090581049-090	CASP8AP2
A_24_P942017	3.2596912	down	XR_078985	chr5:043065679-043	hCG_2039148
A_33_P3369520	2.0842578	down	NM_152900	chr1:114228405-114	MAGI3
A_33_P3412035	2.3076845	down	NM_018227	chr4:068481613-068	UBA6
A_23_P128663	6.8034806	down	NM_014363	chr13:023903098-02	SACS
A_23_P127140	2.03525	down	NM_014904	chr10:119765427-11	RAB11FIP2
A_24_P341923	2.4038089	down	NM_020964	chr18:043445638-04	KIAA1632

Homo sapiens TAO l	GO:0005886|GO:004	NM_016281	Hs.644420	51347	66.123276
Homo sapiens non-SI	GO:0005515|GO:000	NM_022346	Hs.567567	64151	110.19267
Homo sapiens cDNA FLJ34252 fis, clone FCBBF5000061 [AK			Hs.586683	285178	541.6925
Homo sapiens solute	GO:0016020|GO:000	NM_001029998	Hs.659209	84068	11.050041
Homo sapiens 1-acyl	GO:0005739|GO:001	NM_018361	Hs.624002	55326	153.35188
Importin subunit alph	GO:0005515|GO:0005737|GO:0005643|GO:0008565|GO:0005			3841	10.599139
Homo sapiens ubinuclein 2 (UBN2), mRN		NM_173569	Hs.153458	254048	56.94065
V2-6 protein Fragment [Source:UniProtK		XM_002345474	Hs.719643		4.9999995
Homo sapiens ATP/C	GO:0005737|GO:000	NM_015239	Hs.719980	23287	1387.1583
WD repeat-containin	GO:0005515|GO:0005634|GO:0007283|GO:0006301			55339	8.449263
Homo sapiens DAZ i	GO:0005515|GO:000	NM_014648	Hs.409210	9666	75.9597
Homo sapiens plecks	GO:0005737|GO:001	NM_172069	Hs.164162	130271	11.57003
Homo sapiens cripto,	GO:0003674|GO:000	NM_032545	Hs.567542	55997	5.57686
Homo sapiens alpha	GO:0005515|GO:000	NM_000489	Hs.533526	546	108.157394
Endoplasmic reticulum mannosyl-oligosaccharide 1,2-alpha-mar			Hs.279881		13.42789
Homo sapiens oncost	GO:0042506|GO:000	NM_020530	Hs.248156	5008	7.0370183
Centriolin (110 kDa centrosomal protein)(Centrosomal protein 1			Hs.653263		18.341948
Homo sapiens HECT	GO:0005622|GO:000	NM_015382	Hs.708017	25831	1629.1877
					12.712602
Homo sapiens KIAA	GO:0016020|GO:003	NM_001009899	Hs.632570	205717	56.155693
Homo sapiens cDNA FLJ44272 fis, clone TOVAR2000649. [Al			Hs.689537	100129275	4.9999995
Homo sapiens notocl	GO:0043565|GO:000	NM_001134462	Hs.694384	344022	6.148184
Homo sapiens chromosome 3 open reading		NR_026829	Hs.302131	84657	5.8833323
Homo sapiens alpha	GO:0005515|GO:000	NM_000489	Hs.533526	546	31.894493
Homo sapiens RAD5	GO:0005515|GO:000	NM_006479	Hs.504550	10635	453.96222
Homo sapiens zinc fi	GO:0016020|GO:000	NM_174976	Hs.525485	283576	23.568605
Homo sapiens PRP18	GO:0008380|GO:000	NM_003675	Hs.161181	8559	533.318
Homo sapiens vacuol	GO:0015031	NM_181661	Hs.191540	157680	10.07485
Homo sapiens bromo	GO:0005515|GO:000	NM_018963	Hs.654740	54014	52.639114
PREDICTED: Homo sapiens familial acut		XM_001722941	Hs.711077	100131234	13.916278
Deubiquitinating pro	GO:0007067|GO:0005737|GO:0016567|G		Hs.632066	80124	51.847733
Homo sapiens kinesi	GO:0005515|GO:000	NM_138555	Hs.270845	9493	3942.4707
Homo sapiens zona p	GO:0007338|GO:000	NM_021186	Hs.136241	57829	4.9999995
Homo sapiens Ral G'	GO:0005739|GO:000	NM_194301	Hs.113150	253959	5.7990303
Homo sapiens nuclea	GO:0042826|GO:001	NM_003489	Hs.155017	8204	94.778145
Homo sapiens DnaJ (	GO:0006457|GO:000	NM_018981	Hs.516632	54431	1168.4885
Homo sapiens FAT tu	GO:0005515|GO:001	NM_024582	Hs.563205	79633	47.801384
Homo sapiens TBC1	GO:0005622|GO:003	NM_018309	Hs.477003	55773	529.9821
Homo sapiens choroi	GO:0005515|GO:001	NM_001145414	Hs.496449	1121	82.70191
Homo sapiens NMD/	GO:0005515|GO:000	NM_024561	Hs.512914	79612	113.67387
Homo sapiens, clone IMAGE:3932794, mRNA [BC005372]			Hs.659351	84786	45.17212
Homo sapiens stroma	GO:0005515|GO:000	NM_001042751	Hs.496710	10735	17.452559
Homo sapiens family	GO:0003824	NM_198947	Hs.186579	374393	313.0317
Homo sapiens dihydr	GO:0042645|GO:000	NM_001918	Hs.709187	1629	137.61238
Homo sapiens RAR-r	GO:0005515|GO:000	NM_134260	Hs.560343	6095	23.872183
Homo sapiens heat sh	GO:0042470|GO:000	NM_003299	Hs.192374	7184	1873.3387
Homo sapiens chrom	GO:0004003|GO:001	NM_001270	Hs.643465	1105	596.391
					4.9999995
Homo sapiens janus l	GO:0005737|GO:001	NM_144720	Hs.479066	152789	4.9999995
Homo sapiens hypothetical LOC645323 (L		NR_024384	Hs.12827	645323	10.99592
Homo sapiens zinc fi	GO:0005622|GO:000	NM_152262	Hs.528731	90594	161.44719
Homo sapiens protea	GO:0000502|GO:001	NM_014614	Hs.413801	23198	545.71533
					4.9999995
Homo sapiens caspas	GO:0008625|GO:000	NM_012115	Hs.558218	9994	51.219482
PREDICTED: Homo sapiens hypothetical		XR_078985	Hs.649468	100132356	4.9999995
Homo sapiens memb	GO:0005515|GO:000	NM_152900	Hs.486189	260425	220.47722
Homo sapiens ubiqui	GO:0005737|GO:000	NM_018227	Hs.719086	55236	69.21255
Homo sapiens spastic	GO:0003674|GO:000	NM_014363	Hs.159492	26278	704.77484
Homo sapiens RAB1	GO:0005515|GO:000	NM_014904	Hs.173656	22841	231.54279
Homo sapiens KIAA1632 (KIAA1632), m		NM_020964	Hs.514843	57724	12.51114

172.13142	6.173519	7.29381
395.431	6.912049	8.471958
1662.9133	9.223907	10.588011
34.202644	3.615371	4.954367
408.5438	7.392428	8.5188265
28.303637	3.558669	4.6824017
145.43001	5.955867	7.0540514
23.843637	2.3362613	4.440943
4089.2905	10.551064	11.934
33.590595	3.2396278	4.927589
261.85516	6.381436	7.874199
28.364555	3.675838	4.6862946
14.379891	2.6716576	3.7021966
785.3543	6.8866005	9.474342
38.990387	3.8892808	5.1491923
18.696367	2.9904861	4.090235
49.218575	4.3291454	5.4968123
4825.3623	10.7722025	12.184295
37.204933	3.8096735	5.077488
218.94302	5.9359293	7.6333385
12.831609	2.3362613	3.5289032
16.45435	2.8071442	3.8973463
14.824982	2.7475562	3.7507489
190.32024	5.131745	7.437622
1147.8229	8.976032	10.042374
58.572685	4.6996775	5.752784
1409.5394	9.202097	10.347749
53.083786	3.4874568	5.6068916
259.86197	5.8423314	7.863948
69.430305	3.9358587	5.9949627
172.44713	5.8187065	7.296212
9665.096	12.009851	13.2198925
15.07642	2.3362613	3.7746115
28.569244	2.7260227	4.6955504
325.7469	6.6993914	8.193777
3723.6707	10.304921	11.797619
152.69368	5.702392	7.1229954
1447.5593	9.194069	10.387121
229.89214	6.508006	7.700426
388.59827	6.958728	8.44672
138.00847	5.6206446	6.9794035
60.667423	4.2629604	5.8002043
1427.6573	8.445719	10.366066
349.86465	7.2382064	8.294317
61.910603	4.7159524	5.8284693
5568.1733	10.970455	12.398679
2562.6106	9.358141	11.231337
16.248777	2.3362613	3.8811998
21.912113	2.3362613	4.320825
39.896866	3.6081274	5.183841
467.74435	7.469269	8.714933
1367.9628	9.232883	10.303258
18.790266	2.3362613	4.0965414
231.83301	5.8017435	7.7118387
18.00344	2.3362613	4.0409966
567.3995	7.9387593	8.998293
191.60466	6.24242	7.448866
5474.3477	9.607632	12.373905
582.1767	8.012373	9.037579
36.501522	3.7859428	5.051265

A_23_P326142	2.113431	down	NR_027330	chr7:127639207-127	C7orf54
A_24_P380022	2.0444383	down	NM_020390	chr3:170606975-170	EIF5A2
A_23_P162378	3.5721323	down	NM_016122	chr12:094702242-09	CCDC41
A_33_P3821494	2.2885657	down	AK094442	chr22:51052343-510	LOC284939
A_24_P321752	2.1978911	down	NM_015072	chr14:076330039-07	TTLL5
A_33_P3275998	2.2964192	down	NM_004703	chr17:005286459-00	RABEP1
A_23_P357794	2.2154826	down	NM_022045		MTBP
A_24_P538403	3.9839789	down	NM_005406	chr18:018530291-01	ROCK1
A_23_P380379	3.2585361	down	AF351611	chr12:75671463-75671404	
A_23_P256561	2.2531635	down	NM_006068	chr4:038828592-038	TLR6
A_23_P336796	3.4789521	down	NM_173601	chr12:042476664-04	GLT8D3
A_33_P3313250	2.0388265	down	NM_001004477	chr1:158548776-158	OR10X1
A_23_P18447	2.2292438	down	NM_013261	chr4:023793996-023	PPARGC1A
A_33_P3579984	2.6722997	down	AK094521	chr2:8861406-88613	LOC285147
A_33_P3380161	5.4270229	down	NR_026778	chr1:245004000-245	NCRNA00201
A_33_P3559138	2.4677427	down	AK124194	chr21:25862261-258	FLJ42200
A_24_P115932	3.7030954	down	NM_004778	chr11:060618787-06	GPR44
A_33_P3548768	2.6996469	down	CU449054	chr1:152629017-152	C1orf46
A_24_P879740	2.1458987	down	NM_005909	chr5:071504756-071	MAP1B
A_33_P3285585	6.1549494	down	XR_041134	chr16:024674226-02	FLJ45256
A_24_P246091	2.6263604	down	NR_026835	chr10:091451582-09	FLJ37201
A_33_P3360157	2.628189	down	NM_001001921	chr11:055798701-05	OR5AS1
A_24_P93633	2.6934183	down	AK000627	chr9:19357200-19357259	
A_33_P3402414	2.5204197	down	NM_001788	chr7:036013450-036	SEPT7
A_33_P3237250	2.0323575	down	NM_001029964	chr15:090802237-09	TTLL13
A_32_P68533	2.1726067	down	NM_032180	chr2:062052180-062	FAM161A
A_33_P3531828	2.1356852	down	NM_020117	chr5:145557170-145	LARS
A_33_P3311755	2.4423907	down	NM_138555	chr15:069738370-06	KIF23
A_32_P129214	5.498933	down	NR_024090	chr21:022115409-02	C21orf131
A_23_P135977	2.0923497	down	NM_001008938	chr11:046765524-04	CKAP5
A_33_P3310625	3.2671713	down		chr14:22783292-22783351	
A_23_P23611	4.4857686	down	NM_001008219	chr1:104230192-104	AMY1C
A_24_P99090	2.5654662	down	NM_018204	chr13:053035863-05	CKAP2
A_33_P3225600	2.0717839	down	NM_006164	chr2:178095752-178	NFE2L2
A_24_P179611	6.1997498	down	NM_003292	chr1:186324888-186	TPR
A_33_P3336233	2.0220346	down	NM_014235	chrX:153714276-153	UBL4A
A_23_P404606	2.2363362	down	NM_153607	chr5:172563805-172	C5orf41
A_23_P99405	2.5350402	down	NM_003453	chr13:020660278-02	ZMYM2
A_33_P3400324	3.3849277	down	NM_024947	chr3:169805493-169	PHC3
A_33_P3340404	3.0819351	down	NM_144643	chr4:129805401-129	SCLT1
A_23_P58967	2.7596265	down	NM_014827	chr1:219781485-219	ZC3H11A
A_24_P187119	2.1970354	down		chr17:5334888-5334947	
A_33_P3287028	2.3203067	down	NM_001788	chr4:120381922-120	SEPT7
A_33_P3217332	2.10756	down	NM_031218	chr19:020045657-02	ZNF93
A_23_P359870	5.3432025	down	AJ312026	chr8:10984299-10984240	
A_33_P3221925	3.6049061	down	NM_152730	chr6:121400687-121	C6orf170
A_33_P3352877	3.2149322	down	NM_015087	chr13:036875912-03	SPG20
A_33_P3323718	5.8092278	down	NM_001008224	chr15:070947058-07	UACA
A_33_P3375657	4.081445	down		chrX:101604021-101603962	
A_33_P3322400	5.3521598	down	AK095331	chr16:84226915-84226856	
A_33_P3414012	2.3051138	down	BG182298	chr4:174243347-174243288	
A_33_P3867584	2.2043413	down	BC037331	chr6:114189283-114	LOC285758
A_33_P3232354	2.3194822	down	NM_183242	chr1:092604920-092	BTBD8
A_33_P3422614	2.3354663	down		chr10:16769124-16769065	
A_33_P3348164	2.4003926	down	NM_024686	chr1:084373257-084	TTLL7
A_33_P3399881	2.4952407	down	XM_002342834	chr8:012319372-012	LOC646344
A_24_P256337	2.7427307	down	NM_201269	chr1:091381899-091	ZNF644
A_32_P199429	2.3996675	down		chr21:22915446-229	NCAM2
A_33_P3355232	2.1996348	down	NM_145049	chr5:158712890-158	UBLCP1
A_24_P205252	2.0098349	down	NM_001001971	chr10:061006363-06	FAM13C

Homo sapiens chromosome 7 open reading		NR_027330	Hs.657377	27099	46.68509
Homo sapiens eukary	GO:0005515|GO:000	NM_020390	Hs.164144	56648	176.188
Homo sapiens coiled-coil domain containi		NM_016122	Hs.279209	51134	163.4063
Homo sapiens cDNA FLJ37123 fis, clone BRACE2022450 [AK			Hs.656617	284939	4.9999995
Homo sapiens tubuli	GO:0005929|GO:000	NM_015072	Hs.709609	23093	49.423237
Homo sapiens rabapt	GO:0005515|GO:000	NM_004703	Hs.592121	9135	348.34155
Homo sapiens Mdm2	GO:0005515|GO:000	NM_022045	Hs.657656	27085	783.7269
Homo sapiens Rho-a	GO:0007242|GO:003	NM_005406	Hs.306307	6093	380.62097
Calcyphosin-2 (Calcyphosine-2) [Source:UniProtKB/Swiss-Prot			Hs.407154		16.856977
Homo sapiens toll-lik	GO:0045410|GO:000	NM_006068	Hs.662185	10333	6.841802
Homo sapiens glycos	GO:0016020|GO:001	NM_173601	Hs.259347	283464	101.79418
Homo sapiens olfact	GO:0007608|GO:000	NM_001004477	Hs.553589	128367	4.9999995
Homo sapiens peroxi	GO:0008380|GO:000	NM_013261	Hs.527078	10891	23.220364
Homo sapiens cDNA FLJ37202 fis, clone BRALZ2006734 [AK			Hs.467627	285147	30.173273
Homo sapiens non-protein coding RNA 20		NR_026778		284702	147.14299
Homo sapiens cDNA FLJ42200 fis, clone THYMU2034647. [A			Hs.652040	400860	13140.054
Homo sapiens G prot	GO:0045745|GO:001	NM_004778	Hs.299567	11251	4.9999995
CU449054 Homo sapiens ORESTES from keratinocytes Homo			Hs.516420	388699	6.4648137
Homo sapiens micro	GO:0008017|GO:003	NM_005909	Hs.335079	4131	338.6162
PREDICTED: Homo sapiens hypothetical		XR_041134	Hs.592028	400511	4.9999995
Homo sapiens tigger transposable element		NR_026835	Hs.679445	283011	4.9999995
Homo sapiens olfact	GO:0007608|GO:000	NM_001001921	Hs.553625	219447	6.323269
DENN domain-containing protein 4C [Source:UniProtKB/Swis			Hs.249591		9.341291
Homo sapiens septin	GO:0005515|GO:003	NM_001788	Hs.191346	989	1203.4653
Homo sapiens tubuli	GO:0004835|GO:001	NM_001029964	Hs.632164	440307	4.9999995
Homo sapiens family with sequence simila		NM_032180	Hs.440466	84140	178.52258
Homo sapiens leucyl	GO:0005737|GO:000	NM_020117	Hs.432674	51520	277.63956
Homo sapiens kinesi	GO:0005515|GO:000	NM_138555	Hs.270845	9493	1477.4049
Homo sapiens chromosome 21 open readir		NR_024090	Hs.576551	387486	4.9999995
Homo sapiens cytosk	GO:0051297|GO:000	NM_001008938	Hs.201253	9793	500.58728
Putative uncharacterized protein ENSP00000374974 [Source:UniProtKB/TrEMBL;Acc:A6NHK5] [ENST					4.9999995
Homo sapiens amyla	GO:0005515|GO:003	NM_001008219	Hs.655232	278	4.9999995
Homo sapiens cytosk	GO:0007049|GO:000	NM_018204	Hs.444028	26586	138.98018
Homo sapiens nuclea	GO:0045995|GO:004	NM_006164	Hs.155396	4780	105.891
Homo sapiens transl	GO:0005515|GO:000	NM_003292	Hs.279640	7175	1036.607
Homo sapiens ubiqui	GO:0019787|GO:000	NM_014235	Hs.76480	8266	30.944979
Homo sapiens chrom	GO:0043565|GO:000	NM_153607	Hs.484195	153222	35.987656
Homo sapiens zinc fi	GO:0005515|GO:000	NM_003453	Hs.644041	7750	228.51959
Homo sapiens polyh	GO:0008270|GO:000	NM_024947	Hs.529592	80012	367.90964
Homo sapiens sodiu	GO:0005737|GO:004	NM_144643	Hs.654690	132320	178.07718
Homo sapiens zinc fi	GO:0005515|GO:000	NM_014827	Hs.532399	9877	3891.3806
CDNA FLJ25625 fis, clone STM02974 [Source:UniProtKB/TrEMBL;Acc:Q8N1H0] [ENST0000039957					20.545698
Homo sapiens septin	GO:0005515|GO:003	NM_001788	Hs.191346	989	251.28194
Homo sapiens zinc fi	GO:0005622|GO:000	NM_031218	Hs.301059	81931	86.34725
Homo sapiens mRNA for hypothetical protein (C8ORF16) [AJ3			Hs.660259		11.085389
Homo sapiens chrom	GO:0005622|GO:003	NM_152730	Hs.121396	221322	4.9999995
Homo sapiens spasti	GO:0008219	NM_015087	Hs.440414	23111	55.218353
Homo sapiens uveal	GO:0005515|GO:004	NM_001008224	Hs.108049	55075	88.64617
					5.886236
cDNA FLJ41588 fis, clone CTONG2020806 [Source:UniProtK			Hs.614079		4.9999995
RST1163 Athersys RAGE Library Homo sapiens cDNA, mRNA			Hs.722099		18.976969
Homo sapiens cDNA clone IMAGE:5264351 [BC037331]			Hs.359399	285758	34.314404
Homo sapiens BTB (	GO:0005515	NM_183242	Hs.676102	284697	8.806761
Ras suppressor protein 1 (Rsu-1)(RSP-1) [Source:UniProtKB/Swiss-Prot;Acc:Q15404] [ENST0000037790					13.25695
Homo sapiens tubuli	GO:0005929|GO:000	NM_024686	Hs.445826	79739	19.414713
PREDICTED: Homo	GO:0019031|GO:001	XM_002342834	Hs.711000	646344	4.9999995
Homo sapiens zinc fi	GO:0005622|GO:000	NM_201269	Hs.173001	84146	297.11725
Neural cell adhesion	GO:0005515|GO:0030424|GO:0007608|GO:0007413|GO:0005			4685	4.9999995
Homo sapiens ubiqui	GO:0016787|GO:000	NM_145049	Hs.591733	134510	322.75726
Homo sapiens family with sequence simila		NM_001001971	Hs.607594	220965	4.9999995

117.95389	5.670391	6.749978
441.3738	7.5987005	8.630405
703.9586	7.4869475	9.323733
12.85188	2.3362613	3.530705
129.03061	5.7463064	6.8824263
983.2652	8.598857	9.798243
2057.3027	9.756519	10.90414
1814.0096	8.727927	10.722137
65.7264	4.212224	5.916448
19.12259	2.9505184	4.1224704
432.03714	6.8016562	8.600309
11.514159	2.3362613	3.3640003
62.017387	4.6760406	5.832595
97.77802	5.0536427	6.4717245
964.96216	7.3302	9.770361
32100.186	13.690616	14.993808
20.562227	2.3362613	4.224993
21.699587	2.8715296	4.3043003
892.79553	8.559222	9.660804
34.260605	2.3362613	4.9580083
14.623162	2.3362613	3.7293262
20.742905	2.844726	4.238795
30.988775	3.3856187	4.815057
3445.1702	10.346488	11.680152
11.48148	2.3362613	3.3594155
475.98035	7.621335	8.740762
728.3628	8.273295	9.367994
4078.4707	10.640205	11.928499
30.49839	2.3362613	4.795413
1260.4396	9.114167	10.179291
18.041744	2.3362613	4.0443034
24.82047	2.3362613	4.5016165
434.99454	7.251119	8.61034
267.0951	6.853904	7.9047775
7165.29	10.140053	12.772263
75.08449	5.090825	6.1066327
97.46813	5.3057985	6.4669356
710.4764	7.9934254	9.335434
1496.9324	8.675972	10.435097
666.5615	7.6164455	9.240282
11269.959	11.988132	13.452605
54.04239	4.4945755	5.6301336
714.72626	8.129095	9.3434105
221.33974	6.569855	7.6454287
71.47443	3.6187143	6.036419
19.979815	2.3362613	4.186223
212.5387	5.9094067	7.594195
625.48676	6.6063776	9.144724
30.148092	2.7480206	4.7771006
29.784712	2.3362613	4.7563825
52.353363	4.380825	5.585663
91.17834	5.2351604	6.375508
25.065948	3.3018508	4.5156536
37.579327	3.8694844	5.093195
55.75369	4.4138002	5.6770706
13.942099	2.3362613	3.6554403
1001.4519	8.37421	9.829823
13.436889	2.3362613	3.5990958
873.9322	8.489862	9.627126
11.368122	2.3362613	3.3433383

A_23_P14062	2.0394484	down	NM_020401	chr12:069136376-06	NUP107
A_33_P3419460	2.0075064	down	NM_003574	chr18:009959825-00	VAPA
A_33_P3323929	3.1796922	down	XM_001713966	chr10:126915976-12	LOC642622
A_24_P255986	4.6684855	down	NM_001128076		SPDYE4
A_24_P36285	2.4376267	down	NM_013398	chr19:044612163-04	ZNF224
A_33_P3226605	3.1966549	down	NM_033222	chr9:015464389-015	PSIP1
A_23_P57697	2.5900357	down	NM_020865	chr3:153993757-153	DHX36
A_33_P3296858	3.3410695	down	NR_026777	chr10:043009403-04	ZNF37B
A_23_P171388	4.2125789	down	NM_004202	chrY:015816004-015	TMSB4Y
A_33_P3737979	3.5623891	down	DW407923	chr14:91506376-915	SCARNA23
A_33_P3228325	2.3835997	down	NM_001080391	chr2:231410216-231	SP100
A_33_P3299081	2.6136767	down		chr1:1041415-10413	C1orf159
A_32_P75284	2.0626171	down	NM_032116	chr13:030776968-03	KATNAL1
A_23_P91334	2.145693	down	NM_052970	chr20:003733399-00	HSPA12B
A_23_P38304	2.0461301	down	NM_145273	chr17:041926188-04	CD300LG
A_23_P403398	2.5897937	down	NR_002186	chr7:030411448-030	DKFZP586I1420
A_23_P68868	2.2422638	down	NR_002323	chr22:031371927-03	TUG1
A_23_P73809	3.0982469	down	NM_020871	chrX:114345721-114	LRCH2
A_33_P3324137	2.2676162	down	NR_002764	chr20:039667260-03	PRO0628
A_24_P706314	2.6780372	down	NM_194247	chr2:178087920-178	HNRNPA3
A_33_P3280521	2.1887937	down	NM_021647	chr4:170907809-170	MFAP3L
A_32_P54274	2.5344755	down	NM_000798	chr1:148903924-148	DRD5
A_32_P101689	2.0559255	down	NM_014888	chr7:120989898-120	FAM3C
A_23_P30098	3.3441503	down	NM_000670	chr4:100045550-100	ADH4
A_33_P3313785	2.5843922	down	NM_022757	chr3:123633295-123	CCDC14
A_33_P3242833	3.1378302	down	NM_006256	chr1:089270157-089	PKN2
A_23_P74059	7.5676937	down	NM_006172	chr1:011905940-011	NPPA
A_33_P3214625	4.8947155	down	NM_003866	chr4:142949267-142	INPP4B
A_33_P3346338	2.2631443	down	NM_001145088	chr8:124141382-124	WDR67
A_33_P3214343	2.2156403	down	NM_001134478	chr3:111445734-111	PLCXD2
A_32_P192615	3.4071366	down	NM_004606	chrX:070685417-070	TAF1
A_33_P3323803	2.0910066	down	NM_014644	chr1:144851887-144	PDE4DIP
A_23_P20392	2.9549752	down	NM_015310	chr8:018385112-018	PSD3
A_33_P3346669	2.8891418	down	NM_016341	chr10:096084735-09	PLCE1
A_33_P3235701	3.3079378	down	BX648783	chr1:52929446-52929387	
A_23_P328323	2.2107784	down	NM_018211	chr1:065298644-065	RAVER2
A_24_P104538	3.7662409	down	XM_001726269	chr17:65959543-65959602	
A_33_P3617190	2.2460431	down	XR_079077	chr9:140671107-140	LOC651337
A_23_P129332	8.5784007	down	NM_001076780	chr16:081209282-08	PKD1L2
A_32_P99100	2.2521985	down	NM_002844	chr6:128290037-128	PTPRK
A_33_P3671647	3.7919065	down	BC039667	chr4:76287610-7628	LOC441025
A_24_P108311	2.4413904	down	NM_015277	chr18:056065317-05	NEDD4L
A_32_P58937	2.6171663	down	NM_017643	chr17:049255399-04	MBTD1
A_33_P3358312	2.4802635	down	NM_016023	chr8:092099128-092	OTUD6B
A_33_P3323535	3.4619649	down	AK295961	chr14:22574044-22574103	
A_33_P3366659	4.4663555	down	AY255794	chr12:48882957-48883016	
A_23_P216038	3.8201238	down	NM_032205	chr8:133837383-133	PHF20L1
A_33_P3342160	4.015668	down	NM_018036	chr14:096747798-09	ATG2B
A_33_P3367062	2.343642	down	NM_017673	chr1:185260080-185	C1orf26
A_23_P359636	2.0056752	down	NM_144695		C1orf58
A_33_P3401146	3.5718357	down		chrX:065297986-065297927	
A_33_P3441031	2.0017257	down	XM_002343227	chr12:127220071-12	LOC100288701
A_23_P214627	4.1915304	down	NM_004847	chr6:031584674-031	AIF1
A_33_P3270192	3.79516	down		chr2:232996514-232996573	
A_33_P3245290	3.05686	down	NR_002817	chr9:067270274-067	AQP7P1
A_23_P133386	2.9483825	down	NM_006909	chr5:080521773-080	RASGRF2
A_33_P3374718	5.6434848	down		chr10:028808905-028808846	
A_33_P3372985	3.8754926	down	NR_024457	chr12:130518440-13	LOC100190940
A_23_P328766	3.0159358	down	NM_145287	chr18:014105476-01	ZNF519
A_23_P20225	2.0654631	down	NM_015713	chr8:103217234-103	RRM2B

Homo sapiens nuclec	GO:0005515|GO:00(	NM_020401	Hs.524574	57122	3710.6697
Homo sapiens VAMI	GO:0005515|GO:004	NM_003574	Hs.699980	9218	14.826381
PREDICTED: Homo sapiens similar to hC		XM_001713966	Hs.576560	642622	13.665002
Homo sapiens speedy homolog E4 (Xenop		NM_001128076	Hs.368429	388333	5.6501274
Homo sapiens zinc fi	GO:0005622|GO:00(	NM_013398	Hs.720059	7767	34.84505
Homo sapiens PC4 a	GO:0019047|GO:004	NM_033222	Hs.658434	11168	1019.8846
Homo sapiens DEAF	GO:0008026|GO:00(	NM_020865	Hs.446270	170506	422.30075
Homo sapiens zinc finger protein 37B (pse		NR_026777		100129482	29.250984
Homo sapiens thymo	GO:0042989|GO:00(	NM_004202	Hs.159201	9087	4.9999995
HHAGE000653 Human liver regeneration	after partial hepatect(		Hs.689695	677773	13.155261
Homo sapiens SP100	GO:0005694|GO:00(	NM_001080391	Hs.369056	6672	792.0182
Uncharacterized prot	GO:0016020|GO:0016021			54991	4.9999995
Homo sapiens katani	GO:0008568|GO:001	NM_032116	Hs.243596	84056	657.1639
Homo sapiens heat sl	GO:0000166|GO:00(	NM_052970	Hs.516854	116835	4.9999995
Homo sapiens CD30	GO:0006955|GO:00(	NM_145273	Hs.657365	146894	4.9999995
Homo sapiens hypothetical protein DKFZp		NR_002186	Hs.112423	222161	91.936134
Homo sapiens taurine upregulated 1 (non-p		NR_002323	Hs.554829	55000	5734.4004
Homo sapiens leucin	GO:0005515	NM_020871	Hs.65366	57631	17.465
Homo sapiens hypothetical LOC29053 (PI		NR_002764	Hs.714122	29053	53.516323
Homo sapiens hetero	GO:0008380|GO:00(	NM_194247	Hs.516539	220988	1473.2183
Homo sapiens microt	GO:0005886|GO:001	NM_021647	Hs.593942	9848	238.56888
Homo sapiens dopan	GO:0005886|GO:00(	NM_000798	Hs.380681	1816	4.9999995
Homo sapiens family	GO:0008150|GO:00(	NM_014888	Hs.434053	10447	1647.1418
Homo sapiens alcohc	GO:0005503|GO:00(	NM_000670	Hs.1219	127	6.9102983
Homo sapiens coiled-coil domain containi		NM_022757	Hs.645028	64770	282.19278
Homo sapiens protei	GO:0005622|GO:00(	NM_006256	Hs.440833	5586	177.35535
Homo sapiens natriu	GO:0048471|GO:003	NM_006172	Hs.75640	4878	4.9999995
Homo sapiens inositc	GO:0007165|GO:001	NM_003866	Hs.658245	8821	84.242355
Homo sapiens WD re	GO:0005622|GO:003	NM_001145088	Hs.492716	93594	64.51793
Homo sapiens phospl	GO:0016042|GO:00(	NM_001134478	Hs.477114	257068	13.75022
Homo sapiens TAF1	GO:0006368|GO:003	NM_004606	Hs.158560	6872	184.9096
Homo sapiens phospl	GO:0005813|GO:00(	NM_014644	Hs.568247	9659	94.87477
Homo sapiens plecks	GO:0005622|GO:00(	NM_015310	Hs.434255	23362	368.7311
Homo sapiens phospl	GO:0045859|GO:001	NM_016341	Hs.655033	51196	52.709225
Zinc finger CCHC domain-containing protein 11 [Source:UniPr			Hs.655407		7.9223137
Homo sapiens ribonu	GO:0005515|GO:00(	NM_018211	Hs.591443	55225	112.939995
Nucleosome-remodeling factor subunit BP		XM_001726269	Hs.559259		12.803959
PREDICTED: Homo sapiens hypothetical		XR_079077	Hs.603195	651337	13.55202
Homo sapiens polycy	GO:0016020|GO:00(	NM_001076780	Hs.413525	114780	4.9999995
Homo sapiens protei	GO:0010839|GO:003	NM_002844	Hs.155919	5796	1512.135
Homo sapiens cDNA clone IMAGE:5164155 [BC039667]			Hs.407667	441025	6.194879
Homo sapiens neural	GO:0005515|GO:00(	NM_015277	Hs.185677	23327	4315.0767
Homo sapiens mbt d	GO:0008270|GO:00(	NM_017643	Hs.656803	54799	133.82973
Homo sapiens OTU domain containing 6B		NM_016023	Hs.30532	51633	143.88979
Putative uncharacterized protein ENSP00000374960 [Source:U			Hs.508885		4.9999995
Uncharacterized protein C12orf54 [Source:UniProtKB/Swiss-P			Hs.98202		4.9999995
Homo sapiens PHD f	GO:0005515|GO:00(	NM_032205	Hs.304362	51105	50.585506
Homo sapiens ATG2 autophagy related 2 I		NM_018036	Hs.168241	55102	266.88983
Homo sapiens chromosome 1 open reading		NM_017673	Hs.134183	54823	11.7422
Homo sapiens chrom	GO:0016020|GO:003	NM_144695	Hs.552608	148362	157.7568
					4.9999995
PREDICTED: Homo sapiens hypothetical		XM_002343227	Hs.721231	100288701	4.9999995
Homo sapiens allogr	GO:0051015|GO:00(	NM_004847	Hs.76364	199	13.606959
DIS3-like exonuclease 2 (EC 3.1.13.-) [Source:UniProtKB/Swiss-Prot;Acc:Q8IYB7] [ENST00000409401]					39.640736
Homo sapiens aquaporin 7 pseudogene 1 (		NR_002817		375719	4.9999995
Homo sapiens Ras pr	GO:0005515|GO:005	NM_006909	Hs.162129	5924	4.9999995
					4.9999995
Homo sapiens hypothetical LOC10019094		NR_024457		100190940	4.9999995
Homo sapiens zinc fi	GO:0005622|GO:00(	NM_145287	Hs.352635	162655	33.428005
Homo sapiens ribonu	GO:0009186|GO:00(	NM_015713	Hs.512592	50484	153.3747

8102.9204	11.921038	12.949217
35.91224	4.0215964	5.027001
52.14594	3.9119225	5.5808096
33.20287	2.6874714	4.910426
102.48059	5.257574	6.5430512
3698.1516	10.114457	11.79102
1312.8472	8.868681	10.241653
117.91366	5.009247	6.749557
23.339628	2.3362613	4.410965
56.22238	3.8571239	5.689969
2228.8542	9.773467	11.026609
14.551118	2.3362613	3.722342
1619.2959	9.503799	10.548275
12.084871	2.3362613	3.437705
11.548091	2.3362613	3.3691592
291.29602	6.6586018	8.031439
13297.636	12.525149	13.690105
64.74209	4.2637906	5.8952427
144.46912	5.865153	7.0463295
4464.6533	10.635399	12.056575
641.23566	8.051416	9.181552
14.14125	2.3362613	3.6779485
3783.1196	10.78708	11.826868
28.727886	2.9633603	4.705
896.39514	8.297082	9.666907
673.901	7.6102867	9.260054
41.94735	2.3362613	5.256115
502.35013	6.532218	8.823443
174.64867	6.138483	7.3168116
36.920017	3.9196973	5.067421
767.1239	7.6745152	9.443075
240.83249	6.701869	7.7660666
1314.743	8.680098	10.243244
181.86984	5.843318	7.373959
32.561375	3.1574316	4.8833637
303.96024	6.9472466	8.091801
57.767426	3.8192642	5.7323895
36.969177	3.9015107	5.0688963
47.451515	2.3362613	5.43697
3834.1035	10.670709	11.842043
29.49742	2.819513	4.7424364
11008.764	12.130632	13.418335
429.2982	7.2013254	8.589331
435.2494	7.3010736	8.611567
19.198597	2.3362613	4.1278524
24.748817	2.3362613	4.4953594
232.18913	5.781478	7.7150974
1294.7708	8.214659	10.220299
33.54218	3.6969094	4.9256616
387.2095	7.436122	8.44021
19.813713	2.3362613	4.172927
11.32736	2.3362613	3.3375056
68.41829	3.9061089	5.973586
180.5301	5.4395323	7.363693
16.952778	2.3362613	3.9483118
16.439096	2.3362613	3.896185
31.356335	2.3362613	4.8328476
21.49919	2.3362613	4.290641
121.096306	5.194683	6.7872887
387.05954	7.3929377	8.439403

A_24_P65941	2.9807885	down	NR_026812	chr21:036410835-03	C21orf96
A_24_P241792	2.4450133	down	NM_198893	chr19:053569960-05	ZNF160
A_23_P40805	7.5378182	down	NM_014079	chr3:126062515-126	KLF15
A_33_P3382147	3.4230717	down		chr13:033712626-033712567	
A_33_P3351559	2.1221255	down	NM_018638	chr12:022843337-02	ETNK1
A_33_P3350453	8.4848532	down	XM_001722773	chr20:049307422-04	LOC100130454
A_33_P3246699	4.0968251	down	XM_001717722		LOC100133890
A_23_P329261	4.1906941	down	NM_000891	chr17:068176016-06	KCNJ2
A_33_P3356031	2.481751	down		chr1:183485149-183485208	
A_33_P3403232	3.249626	down	XM_001715173	chr21:047572467-04	LOC100128336
A_24_P358619	2.5726669	down	NM_001080409	chr19:022940739-02	ZNF99
A_32_P180210	2.3355932	down	NM_019054	chr10:046898787-04	FAM35A
A_32_P187663	2.1221336	down	NM_001042416	chr8:000195612-000	ZNF596
A_23_P44942	2.356109	down	NM_025004	chr11:124910525-12	CCDC15
A_33_P3313417	4.528109	down		chr19:36270781-36270840	
A_23_P397320	2.659782	down	NM_144616	chr19:002252615-00	JSRP1
A_33_P3293668	2.4997947	down	NM_017925	chr9:019374048-019	DENND4C
A_33_P3403560	3.5255787	down	NM_001136508	chr1:051613668-051	C1orf185
A_33_P3422035	2.1814494	down		chr19:35654951-35655010	
A_33_P3218768	2.4740398	down	NM_080599	chr10:012041965-01	UPF2
A_33_P3403773	3.152221	down	NM_152484	chr19:037902138-03	ZNF569
A_23_P170534	2.0572236	down	NM_004479	chr9:139925373-139	FUT7
A_23_P115246	2.2176763	down	NM_003665	chr1:027695885-027	FCN3
A_33_P3233764	2.4271743	down	NM_004690	chr6:150022975-150	LATS1
A_23_P349310	2.2406099	down	NM_014494	chr16:024835610-02	TNRC6A
A_32_P96000	7.1426216	down	NM_001004341	chr1:157062195-157	ETV3L
A_24_P205120	2.3882502	down	NM_199367	chr16:089603327-08	SPG7
A_23_P45185	2.0921264	down	NM_004469	chrX:015363842-015	FIGF
A_23_P8482	2.8356648	down	NM_001011553	chr7:035943947-035	SEPT7
A_33_P3280694	2.2360358	down		chr7:76958930-76958871	
A_23_P30243	2.5101991	down	NM_022350	chr5:096253257-096	ERAP2
A_33_P3316835	2.3043494	down		chr4:113527199-113527140	
A_33_P3409447	4.5184448	down	NM_016248	chr13:042877096-04	AKAP11
A_33_P3251801	3.8782363	down	NM_198508	chr7:139138147-139	KLRG2
A_33_P3287338	3.1825335	down	NM_175767	chr5:055256322-055	IL6ST
A_33_P3275878	2.0808842	down	NM_000542	chr2:085886044-085	SFTPB
A_24_P333571	4.3704472	down	BC022483	chr1:94667593-94667534	
A_24_P146670	2.9049997	down	NM_014720	chr10:105786654-10	SLK
A_23_P16817	2.2099642	down	NM_004071		CLK1
A_23_P377996	2.0723657	down	NM_178532	chr5:063510055-063	RNF180
A_23_P160200	2.2313715	down	NM_023070	chr1:040929073-040	ZNF643
A_23_P215980	2.0165737	down	NM_183009	chr8:095523940-095	KIAA1429
A_33_P3333507	3.8722836	down	NM_020654	chr3:101044770-101	SENP7
A_33_P3241984	2.0070067	down	NM_015967	chr1:114356520-114	PTPN22
A_23_P106127	2.6141302	down	NM_014749	chr14:059014698-05	KIAA0586
A_23_P137896	2.6922245	down	NM_022120	chr1:040236107-040	OXCT2
A_24_P288890	5.3071753	down	NM_181709	chr12:124799557-12	FAM101A
A_24_P255218	2.6156502	down	NM_000259	chr15:052643522-05	MYO5A
A_23_P154488	2.0382486	down	NM_033109		PNPT1
A_23_P74663	2.1743633	down	NM_005681	chr1:222734784-222	TAF1A
A_33_P3331351	5.0111951	down	NM_001105575	chr4:165981647-165	TRIM75
A_23_P42855	4.2550067	down	NM_152706	chr7:088423569-088	C7orf62
A_23_P217659	2.6273675	down	NM_001018055	chrX:154351125-154	BRCC3
A_24_P215475	2.2370216	down	NM_015394	chr12:133735529-13	ZNF10
A_33_P3308905	2.018467	down	NM_001083535	chr6:109484879-109	C6orf182
A_24_P385336	2.2512958	down	NM_152696	chr1:114512610-114	HIPK1
A_33_P3337549	9.783351	down	NM_001163942	chr7:020699676-020	ABCB5
A_33_P3733417	2.9767067	down	NM_000795	chr11:113280398-11	DRD2
A_23_P201996	2.0158216	down	NM_016628	chr10:028909391-02	WAC
A_23_P214766	2.1342364	down	NM_006734	chr6:143072910-143	HIVEP2

Homo sapiens chrom	GO:0005829	NR_026812	Hs.672131	80215	34.161995
Homo sapiens zinc fi	GO:0030097|GO:000	NM_198893	Hs.655967	90338	165.87256
Homo sapiens Krupp	GO:0005622|GO:000	NM_014079	Hs.272215	28999	6.4068756
					77.17514
Homo sapiens ethanc	GO:0004305|GO:000	NM_018638	Hs.29464	55500	231.54163
PREDICTED: Homo sapiens hypothetical		XM_001722773		100130454	4.9999995
PREDICTED: Homo sapiens similar to Sh		XM_001717722		100133890	4.9999995
Homo sapiens potass	GO:0005515|GO:000	NM_000891	Hs.1547	3759	4.9999995
Protein SMG7 (SMG-7 homolog)(EST1-like protein C) [Source:UniProtKB/Swiss-Prot;Acc:Q92540] [ENS					28.781574
PREDICTED: Homo sapiens hypothetical		XM_001715173	Hs.570448	100128336	4.9999995
Homo sapiens zinc fi	GO:0005622|GO:000	NM_001080409	Hs.653115	7652	150.19698
Homo sapiens family with sequence simila		NM_019054	Hs.500419	54537	139.5515
Homo sapiens zinc fi	GO:0005622|GO:000	NM_001042416	Hs.591388	169270	48.45012
Homo sapiens coiled-coil domain containi		NM_025004	Hs.287555	80071	37.509922
TC10/CDC42 GTPase-activating protein (Sorting nexin-26) [Source:UniProtKB/Swiss-Prot;Acc:O14559] [					4.9999995
Homo sapiens junctic	GO:0016020|GO:003	NM_144616	Hs.712901	126306	6.040262
Homo sapiens DENN	GO:0016020|GO:001	NM_017925	Hs.249591	55667	227.50032
Homo sapiens chrom	GO:0016020|GO:001	NM_001136508	Hs.176177	284546	4.9999995
FXYD domain-containing ion transport regulator 5 Precursor (Dysadherin) [Source:UniProtKB/Swiss-Prot;					4.9999995
Homo sapiens UPF2	GO:0048471|GO:000	NM_080599	Hs.370689	26019	83.95109
Homo sapiens zinc fi	GO:0005622|GO:000	NM_152484	Hs.511848	148266	182.27008
Homo sapiens fucosy	GO:0046920|GO:004	NM_004479	Hs.457	2529	15.82769
Homo sapiens ficolin	GO:0007165|GO:000	NM_003665	Hs.333383	8547	39.1771
Homo sapiens LATS	GO:0005515|GO:000	NM_004690	Hs.716697	9113	13.448379
Homo sapiens trinucl	GO:0035278|GO:000	NM_014494	Hs.655057	27327	230.98105
Homo sapiens ets var	GO:0043565|GO:000	NM_001004341	Hs.127138	440695	4.9999995
Homo sapiens spastic	GO:0008219|GO:001	NM_199367	Hs.185597	6687	10.558049
Homo sapiens c-fos i	GO:0048010|GO:000	NM_004469	Hs.11392	2277	19.484089
Homo sapiens septin	GO:0005515|GO:003	NM_001011553	Hs.191346	989	2444.9277
Protein pigeon homolog [Source:UniProtKB/Swiss-Prot;Acc:A4D1B5] [ENST00000334003]					28.35941
Homo sapiens endop	GO:0006955|GO:001	NM_022350	Hs.482910	64167	111.8572
Uncharacterized protein C4orf21 [Source:UniProtKB/Swiss-Prot;Acc:Q86YA3] [ENST00000264370]					167.89001
Homo sapiens A kina	GO:0005813|GO:000	NM_016248	Hs.105105	11215	23.45263
Homo sapiens killer c	GO:0016020|GO:000	NM_198508	Hs.17572	346689	4.9999995
Homo sapiens interle	GO:0005138|GO:000	NM_175767	Hs.532082	3572	132.45827
Homo sapiens surfac	GO:0006665|GO:000	NM_000542	Hs.512690	6439	4.9999995
Rho GTPase-activating protein 29 (Rho-type GTPase-activating			Hs.483238		37.11943
Homo sapiens STE20	GO:0005737|GO:000	NM_014720	Hs.591922	9748	702.9737
Homo sapiens CDC-l	GO:0046777|GO:001	NM_004071	Hs.433732	1195	1040.7335
Homo sapiens ring fi	GO:0005515|GO:000	NM_178532	Hs.657843	285671	17.680782
Homo sapiens zinc fi	GO:0005622|GO:000	NM_023070	Hs.133034	65243	119.907684
Homo sapiens KIAA	GO:0008380|GO:000	NM_183009	Hs.202238	25962	43.86202
Homo sapiens SUMO	GO:0005622|GO:001	NM_020654	Hs.529551	57337	27.872616
Homo sapiens protein	GO:0005515|GO:005	NM_015967	Hs.535276	26191	4.9999995
Homo sapiens KIAA0586 (KIAA0586), m		NM_014749	Hs.232532	9786	855.24976
Homo sapiens 3-oxoa	GO:0005739|GO:004	NM_022120	Hs.472491	64064	4.9999995
Homo sapiens family with sequence simila		NM_181709	Hs.432901	144347	4.9999995
Homo sapiens myosin	GO:0030048|GO:000	NM_000259	Hs.21213	4644	164.27951
Homo sapiens polyril	GO:0005739|GO:000	NM_033109	Hs.388733	87178	1658.9563
Homo sapiens TATA	GO:0005515|GO:000	NM_005681	Hs.153088	9015	259.8659
Homo sapiens tripart	GO:0005622|GO:000	NM_001105575	Hs.705357	391714	4.9999995
Homo sapiens hypothetical protein MGC2		NM_152706	Hs.112877	219557	4.9999995
Homo sapiens BRCA	GO:0005515|GO:000	NM_001018055	Hs.558537	79184	739.79663
Homo sapiens zinc fi	GO:0005622|GO:000	NM_015394	Hs.507355	7556	76.13137
Homo sapiens chrom	GO:0008150|GO:000	NM_001083535	Hs.632616	285753	102.51978
Homo sapiens homec	GO:0005515|GO:000	NM_152696	Hs.532363	204851	18.48581
Homo sapiens ATP-b	GO:0005886|GO:000	NM_001163942		340273	4.9999995
Homo sapiens dopan	GO:0005515|GO:000	NM_000795	Hs.73893	1813	4.9999995
Homo sapiens WW d	GO:0005515|GO:001	NM_016628	Hs.435610	51322	2780.6619
Homo sapiens humar	GO:0005622|GO:000	NM_006734	Hs.510172	3097	283.16446

122.40868	5.228147	6.803841
495.67606	7.5109577	8.8008
59.502087	2.85996	5.774107
323.12274	6.4054995	8.180791
606.9909	8.012301	9.097811
46.966564	2.3362613	5.421151
22.707378	2.3362613	4.3707676
23.231964	2.3362613	4.4034505
86.4246	4.9892626	6.300621
17.97687	2.3362613	4.036535
471.75723	7.3631263	8.726391
398.1915	7.258881	8.48267
122.66125	5.7203507	6.8058662
106.502014	5.3613014	6.5977077
25.054361	2.3362613	4.51517
20.10322	2.783114	4.194422
697.15857	7.9863434	9.308153
19.564106	2.3362613	4.1541214
12.27322	2.3362613	3.4615483
253.9931	6.5272493	7.834118
699.0427	7.6553383	9.311707
39.246464	4.1196737	5.1603723
104.79481	5.4241858	6.5732346
39.55608	3.8913193	5.170597
637.25214	8.007294	9.1711855
39.593113	2.3362613	5.172715
30.819666	3.552886	4.80884
48.914093	4.4195447	5.4845147
7567.52	11.346945	12.850632
76.34898	4.967262	6.1282053
341.30347	6.9337482	8.26155
474.142	7.5292425	8.733602
127.52519	4.6918917	6.867718
21.509987	2.3362613	4.291662
513.10693	7.1872463	8.857422
11.74887	2.3362613	3.393458
195.2425	5.3467646	7.4745455
2419.6848	9.603937	11.142475
2666.544	10.144811	11.288834
44.197117	4.279252	5.3305306
325.20663	7.0337443	8.191675
105.930695	5.5780516	6.5899577
130.50116	4.9434314	6.896616
11.35366	2.3362613	3.3413067
2619.0413	9.876547	11.262878
14.979239	2.3362613	3.76506
29.53003	2.3362613	4.7442055
521.869	7.4941974	8.881367
3781.4595	10.799004	11.826334
690.7301	8.176278	9.296871
27.796736	2.3362613	4.661416
23.578228	2.3362613	4.4254227
2287.1362	9.673831	11.067449
205.75572	6.3858566	7.5474358
252.24495	6.812788	7.826048
49.664066	4.339597	5.5103526
53.889477	2.3362613	5.62659
16.560793	2.3362613	3.9099784
6073.7417	11.518547	12.529915
742.0079	8.302232	9.395952

A_33_P3294524	4.9600408	down	NM_015208	chr18:009255735-00	ANKRD12
A_23_P30162	2.0009224	down	NM_152778	chr4:128843091-128	MFSD8
A_24_P98251	3.3906268	down	NM_017890	chr8:100889712-100	VPS13B
A_23_P254702	3.0725511	down	NM_003472	chr6:018225006-018	DEK
A_23_P160751	8.1367333	down	NM_030764	chr1:157716519-157	FCRL2
A_23_P151405	2.655096	down	NM_018204	chr13:053050394-05	CKAP2
A_23_P41470	3.4975968	down	NM_017631	chr4:169142950-169	DDX60
A_23_P359647	3.2828572	down	NM_138714	chr16:069738311-06	NFAT5
A_33_P3212490	2.9413531	down	NM_177438	chr14:095552674-09	DICER1
A_24_P261734	2.5486195	down	NM_030674	chr12:046577120-04	SLC38A1
A_24_P555510	4.6449289	down	NM_006197	chr8:017887294-017	PCM1
A_33_P3465247	2.2335716	down	NM_007054	chr5:132032073-132	KIF3A
A_23_P155939	2.2520789	down	NM_182524	chr4:000086842-000	ZNF595
A_33_P3375736	4.5486685	down	NM_170710	chr4:177102634-177	WDR17
A_33_P3275674	2.8051661	down	XR_037883	chr8:054886106-054	LOC100131254
A_23_P362893	2.6431458	down	NM_021961	chr11:012965225-01	TEAD1
A_23_P139388	3.8161635	down	NM_016578	chr11:077377685-07	RSF1
A_24_P380679	2.2008126	down	NM_182597	chr7:112130859-112	C7orf53
A_23_P345678	2.211341	down	NM_033084	chr3:010140927-010	FANCD2
A_23_P20172	3.3195622	down	NM_005295	chr7:107115743-107	GPR22
A_33_P3370222	4.8358105	down		chr9:97427458-97427399	
A_23_P394545	3.7255153	down	NM_015275	chr12:105540848-10	KIAA1033
A_23_P257417	2.1133684	down	NM_152385		C2orf63
A_33_P3242649	3.0944317	down	NM_031217	chr11:028042475-02	KIF18A
A_33_P3520835	4.1256774	down	AK123449	chr9:45447622-4544	FLJ41455
A_33_P3217731	3.1507725	down		chr9:84214882-84214823	
A_33_P3266550	2.3553951	down	NM_020469	chr9:136130807-136	ABO
A_23_P86550	3.118988	down	NM_003750	chr2:070499218-070	EIF3A
A_33_P3318596	2.2188836	down	NM_003419	chr19:037369023-03	ZNF345
A_23_P218068	2.0755879	down	NM_019012	chr12:019529106-01	PLEKHA5
A_33_P3350744	2.8773737	down		chr20:043345294-043345235	
A_23_P54447	4.0690113	down	NR_026813	chr15:077516360-07	C15orf5
A_23_P338372	2.189856	down	NM_003269	chr6:108509456-108	NR2E1
A_32_P83465	2.4851329	down	NM_001039703	chr1:148298225-148	NBPF10
A_32_P4018	2.3178224	down	AK000776	chr1:64646792-6464	ROR1
A_33_P3312384	8.6993928	down	NR_027790	chr21:017979483-01	C21orf34
A_23_P66593	2.2247682	down	NM_002055	chr17:042983374-04	GFAP
A_24_P222684	2.4070798	down	NM_001042369	chr1:193053711-193	TROVE2
A_33_P3301955	2.0961907	down	NM_002604	chr8:066635836-066	PDE7A
A_24_P410516	2.6446064	down	NM_198181	chr15:082810838-08	GOLGA6L9
A_33_P3473108	2.4184265	down	Z39353	chr1:93306531-93306590	
A_33_P3269453	3.328172	down		chr17:65955936-65955995	
A_33_P3290335	2.1570053	down		chr1:226589743-226589684	
A_33_P3340769	2.0137094	down	NM_003816	chr8:038962718-038	ADAM9
A_24_P200848	3.2452043	down	NM_015132	chr7:017831097-017	SNX13
A_23_P416751	2.6031936	down	NM_173530	chr19:052870021-05	ZNF610
A_33_P3335022	3.9135549	down	XM_002348274	chr2:096516938-096	LOC400986
A_33_P3308740	4.1666985	down	NM_006267	chr2:109379969-109	RANBP2
A_23_P216622	2.7106696	down	NM_001079802	chr9:108402444-108	FKTN
A_33_P3257683	2.014561	down		chr5:095551237-095551296	
A_33_P3349299	3.7953802	down	BC038573	chrX:46746920-46746861	
A_24_P397903	2.1972606	down	NM_016010	chr8:079631797-079	FAM164A
A_32_P144421	2.5955855	down	NM_053042	chr4:010441743-010	ZNF518B
A_33_P3418380	9.5398858	down		chr16:034724566-034724625	
A_33_P3258772	2.0908321	down		chr2:178587952-178588011	
A_23_P91250	2.8185712	down	NM_199441	chr20:045129999-04	ZNF334
A_33_P3283480	2.0891369	down	NM_148170	chr11:088054087-08	CTSC
A_33_P3369550	2.5337339	down	NM_004768	chr1:070716305-070	SFRS11
A_23_P140328	4.5543116	down	NM_004713	chr14:050250771-05	SDCCAG1
A_32_P144908	2.8538292	down	NM_203282	chr19:024309844-02	ZNF254

Homo sapiens ankyri	GO:0005634	NM_015208	Hs.464585	23253	85.85621
Homo sapiens major	GO:0008219|GO:001	NM_152778	Hs.480701	256471	40.73391
Homo sapiens vacuol	GO:0015031	NM_017890	Hs.191540	157680	227.50499
Homo sapiens DEK (	GO:0042393|GO:000	NM_003472	Hs.484813	7913	6990.173
Homo sapiens Fc rec	GO:0005886|GO:000	NM_030764	Hs.437393	79368	4.9999995
Homo sapiens cytosk	GO:0007049|GO:000	NM_018204	Hs.444028	26586	2000.0645
Homo sapiens DEAD	GO:0008026|GO:001	NM_017631	Hs.591710	55601	202.39386
Homo sapiens nuclea	GO:0005515|GO:000	NM_138714	Hs.371987	10725	417.59406
Homo sapiens dicer 1	GO:0030423|GO:000	NM_177438	Hs.87889	23405	398.08728
Homo sapiens solute	GO:0015804|GO:000	NM_030674	Hs.533770	81539	3378.3188
Homo sapiens perice	GO:0051297|GO:000	NM_006197	Hs.491148	5108	101.11081
Homo sapiens kinesi	GO:0005515|GO:000	NM_007054	Hs.43670	11127	412.85223
Homo sapiens zinc fi	GO:0005622|GO:000	NM_182524	Hs.709469	152687	134.1974
Homo sapiens WD repeat domain 17 (WD		NM_170710	Hs.532056	116966	4.9999995
PREDICTED: Homo sapiens similar to AI		XR_037883	Hs.710233	100131254	4.9999995
Homo sapiens TEA c	GO:0007507|GO:000	NM_021961	Hs.655331	7003	1368.5914
Homo sapiens remod	GO:0016564|GO:000	NM_016578	Hs.420229	51773	132.16112
Homo sapiens chrom	GO:0016020|GO:001	NM_182597	Hs.396189	286006	60.752705
Homo sapiens Fanco	GO:0005515|GO:000	NM_033084	Hs.208388	2177	32.364166
Homo sapiens G prot	GO:0007165|GO:000	NM_005295	Hs.657277	2845	9.169728
Putative uncharacterized protein C9orf118	[Source:UniProtKB/Swiss-Prot;Acc:A6NHY6] [ENST0000037				4.9999995
Homo sapiens KIAA1033 (KIAA1033), m		NM_015275	Hs.12144	23325	207.60559
Homo sapiens chrom	GO:0005488	NM_152385	Hs.347014	130162	46.745106
Homo sapiens kinesi	GO:0005515|GO:000	NM_031217	Hs.301052	81930	199.22629
Homo sapiens cDNA FLJ41455 fis, clone BRSTN2012284 [AK			Hs.494023	441441	6.0192037
Transducin-like enhancer protein 1 (Enhancer of split groucho-like protein 1)(ESG1)(E(Sp1) homolog) [So					19.022448
Homo sapiens ABO	GO:0030173|GO:003	NM_020469	Hs.654423	28	26.860516
Homo sapiens eukary	GO:0005515|GO:000	NM_003750	Hs.523299	8661	18461.727
Homo sapiens zinc fi	GO:0005622|GO:000	NM_003419	Hs.362324	25850	57.25918
Homo sapiens plecks	GO:0005515|GO:000	NM_019012	Hs.188614	54477	276.5795
					10.801561
Homo sapiens chromosome 15 open readir		NR_026813	Hs.656938	81698	20.773066
Homo sapiens nuclea	GO:0043565|GO:000	NM_003269	Hs.157688	7101	4.9999995
Homo sapiens neurol	GO:0005737	NM_001039703	Hs.607640	100132406	17322.682
Tyrosine-protein kin	GO:0005515|GO:0005737|GO:0005886|G		Hs.128753	4919	457.95187
Homo sapiens chromosome 21 open readir		NR_027790	Hs.473394	388815	4.9999995
Homo sapiens glial fi	GO:0005882|GO:000	NM_002055	Hs.514227	2670	4.9999995
Homo sapiens TROV	GO:0005515|GO:000	NM_001042369	Hs.288178	6738	1160.7568
Homo sapiens phosp	GO:0007165|GO:001	NM_002604	Hs.527119	5150	34.394714
Homo sapiens golgi autoantigen, golgin su		NM_198181	Hs.630181	440295	710.23505
HSC18A052 normalized infant brain cDNA Homo sapiens cDN			Hs.657439		27.055422
Nucleosome-remodeling factor subunit BPTF (Bromodomain and PHD finger-containing transcription facto					257.23203
Poly [ADP-ribose] polymerase 1 (PARP-1)(EC 2.4.2.30)(ADPRT)(NAD(+) ADP-ribosyltransferase 1)(Pol					4.9999995
Homo sapiens ADAM	GO:0042542|GO:005	NM_003816	Hs.591852	8754	147.6057
Homo sapiens sorting	GO:0005515|GO:000	NM_015132	Hs.487648	23161	167.42809
Homo sapiens zinc fi	GO:0005622|GO:000	NM_173530	Hs.147025	162963	6.713925
PREDICTED: Homo	GO:0008200|GO:003	XM_002348274		400986	29.272165
Homo sapiens RAN	GO:0005515|GO:000	NM_006267	Hs.199561	5903	43.10725
Homo sapiens fukuti	GO:0007517|GO:000	NM_001079802	Hs.55777	2218	114.3756
					36.702946
Putative uncharacterized protein CXorf31	[Source:UniProtKB/S		Hs.679235		11.92417
Homo sapiens family with sequence simila		NM_016010	Hs.271876	51101	36.364162
Homo sapiens zinc fi	GO:0005622|GO:000	NM_053042	Hs.455089	85460	291.53143
					4.9999995
					10.37136
Homo sapiens zinc fi	GO:0005622|GO:000	NM_199441	Hs.584933	55713	42.716465
Homo sapiens cathep	GO:0004197|GO:003	NM_148170	Hs.128065	1075	331.60513
Homo sapiens splicin	GO:0008380|GO:000	NM_004768	Hs.479693	9295	2343.3225
Homo sapiens serolo	GO:0005737|GO:000	NM_004713	Hs.655964	9147	467.3086
Homo sapiens zinc fi	GO:0005622|GO:000	NM_203282	Hs.434406	9534	3918.821

518.41254	6.562004	8.872356
98.1616	5.4769435	6.4776087
950.05273	7.986659	9.748211
21528.738	12.804117	14.423554
45.02704	2.3362613	5.360711
5805.679	11.059622	12.468386
870.96497	7.813946	9.62031
1640.0753	8.85326	10.568212
1408.6079	8.79052	10.347
9095.36	11.78282	13.132536
570.6694	6.7913585	9.007015
1114.2871	8.838126	9.9974785
371.01694	7.2052016	8.376459
25.176777	2.3362613	4.5217056
15.625442	2.3362613	3.8243475
4083.834	10.530153	11.932409
613.44403	7.181862	9.113985
160.162	6.0482273	7.1862636
86.18439	5.1522875	6.297209
37.498753	3.358335	5.089328
26.803492	2.3362613	4.610019
952.0488	7.854932	9.752372
118.08308	5.67185	6.7513943
753.90106	7.7869625	9.416637
31.196857	2.7795286	4.8241596
71.53627	4.382737	6.0384426
76.20941	4.8891444	6.1251135
56253.62	14.163916	15.804994
151.9392	5.962694	7.112528
701.8194	8.265892	9.319412
37.902454	3.5838428	5.1085954
101.68497	4.5075397	6.532218
12.316641	2.3362613	3.4670973
42612.53	14.074126	15.387449
1278.4491	8.989556	10.202326
48.111355	2.3362613	5.457176
12.50373	2.3362613	3.4899163
3209.87	10.297381	11.564665
86.74793	5.2379665	6.3057365
2223.1948	9.618946	11.021999
78.77229	4.8989363	6.173005
1048.7072	8.164057	9.898787
12.1508	2.3362613	3.445291
361.98987	7.334564	8.3444195
659.656	7.5244427	9.222752
21.672092	2.9225338	4.3028164
137.96364	5.0100803	6.97856
215.96004	5.555397	7.6143017
378.0365	6.9675007	8.40615
89.02973	5.3322515	6.342717
54.510246	3.7171702	5.6414146
96.64052	5.320381	6.456087
931.9878	8.342436	9.718496
52.510597	2.3362613	5.5902333
26.44113	3.5274851	4.5915623
143.47672	5.542164	7.037128
854.64844	8.531581	9.594488
6460.7544	11.286342	12.627607
2514.593	9.015706	11.202939
11761.946	12.000608	13.513507

A_33_P3294951	3.7691628	down	NM_001097643	chr12:011285979-01	TAS2R30
A_33_P3567967	4.015643	down	AK023417	chr5:86511671-8651	FLJ11292
A_24_P826348	2.3942487	down	NM_198581	chr2:113097441-113	ZC3H6
A_32_P226786	2.6010272	down	NM_173822	chr2:201838745-201	FAM126B
A_33_P3238651	2.0478394	down	NM_001039783	chr3:126290860-126	TXNRD3IT1
A_23_P99424	2.1284438	down	NM_003291	chr13:103330625-10	TPP2
A_24_P85511	6.323081	down	NM_014915	chr10:027294208-02	ANKRD26
A_33_P3221458	3.2045936	down	NR_002722	chr6:027325667-027	ZNF204
A_33_P3345469	2.8439873	down	NM_005284	chr6:110301859-110	GPR6
A_33_P3212232	7.8279943	down	NM_001039396	chr11:058978295-05	MPEG1
A_33_P3415336	2.3913394	down		chr2:202630165-202630106	
A_33_P3307980	6.7033832	down	AK131345	chr7:92319353-92319294	
A_23_P388855	3.3708798	down	NM_012330	chr10:076790506-07	MYST4
A_33_P3405966	5.2713636	down	NM_174963	chr1:044395815-044	ST3GAL3
A_23_P377411	4.3306104	down	AF130105	chr10:46358850-463	LOC100133130
A_24_P359441	2.5440187	down	NM_004076		CRYBB3
A_33_P3330323	2.5610198	down	NM_001080541	chr15:042021388-04	MGA
A_23_P211064	2.1280818	down	NM_013329	chr21:034131469-03	C21orf66
A_33_P3335030	3.5520798	down	NM_001164315	chr2:096576182-096	ANKRD36
A_33_P3248992	2.6289761	down	NM_001609	chr10:124817580-12	ACADSB
A_24_P206317	8.9787842	down	NM_058177	chr7:018801801-018	HDAC9
A_33_P3223749	2.1742231	down	NM_004229	chrX:040508901-04(	MED14
A_24_P227069	2.266814	down	NM_020918	chr10:113909886-11	GPAM
A_23_P18939	2.8722633	down	NM_002890	chr5:086686765-086	RASA1
A_24_P96780	5.5368852	down	NM_016343	chr1:214826239-214	CENPF
A_24_P167877	2.2668407	down	NM_001135865	chr16:021413808-02	LOC100132247
A_33_P3248948	3.6243822	down		chr11:008008895-008008836	
A_33_P3330952	2.8152115	down	NM_006095	chr4:042410508-042	ATP8A1
A_23_P58031	2.1646653	down	NM_004721	chr3:185200168-185	MAP3K13
A_33_P3216557	3.9612855	down	XM_001721247	chr3:187451578-187	LOC100131635
A_24_P345540	2.5766841	down	NM_032494		ZC3H8
A_33_P3273822	2.3434904	down		chr2:48016520-48016461	
A_33_P3348973	2.7294103	down	NM_152671	chr2:209168982-209	PIKFYVE
A_33_P3346936	2.262145	down		chr12:062902218-062902277	
A_23_P26021	2.6568854	down	NM_004236	chr15:049420020-04	COPS2
A_33_P3398727	2.1086103	down	BQ441254	chr22:16148815-16148756	
A_23_P350045	2.8118975	down	NM_005669	chr5:112212209-112	REEP5
A_33_P3238196	2.7226037	down	NM_006954	chr10:038345429-03	ZNF33A
A_33_P3395713	4.5139721	down	NM_018963	chr21:040574389-04	BRWD1
A_33_P3290800	2.0840179	down	NM_182540	chrX:134715008-13	DDX26B
A_33_P3338011	4.4652774	down	NM_144684	chr19:052826052-05	ZNF480
A_32_P45168	2.3889555	down	NR_002211	chr17:015692683-01	MEIS3P1
A_33_P3402725	3.3964462	down	NM_018489	chr1:155305915-155	ASH1L
A_33_P3342992	2.9594617	down	NR_004847	chr15:082707625-08	UBE2QP2
A_23_P316582	5.1998359	down	NM_152355	chr19:011892951-01	ZNF441
A_24_P50368	3.8334451	down	NM_001001786	chr11:121986373-12	BLID
A_33_P3408443	2.141111	down		chr16:032617163-032617222	
A_33_P3424384	2.9441803	down	AF380578	chr2:39996103-39996044	
A_24_P126060	3.0412217	down	NM_001356	chrX:041208627-041	DDX3X
A_23_P167818	2.1846099	down	NM_024581	chr6:119281299-119	FAM184A
A_33_P3311653	2.2294822	down		chr4:039838843-039838784	
A_33_P3375527	2.3807333	down	NM_152622	chr5:056231557-056	MIER3
A_33_P3335740	2.1352009	down	NR_026891	chr15:050641323-05	FLJ10038
A_24_P7202	3.7683945	down	NM_020738		KIDINS220
A_23_P38723	3.598416	down	NM_015295	chr18:002802664-00	SMCHD1
A_23_P412029	2.6715963	down	NM_144709	chr2:061168287-061	PUS10
A_33_P3626360	3.7867469	down	AF547222	chr4:47939716-47939775	
A_23_P341700	2.7900375	down	NM_001007101	chr9:095608515-095	ZNF484
A_32_P83784	2.4611277	down	NM_015230	chr4:036068160-036	ARAP2
A_23_P98930	3.8989699	down	NM_018169	chr12:032145764-03	C12orf35

Homo sapiens taste r	GO:0007165|GO:005	NM_001097643	Hs.679464	259293	4.9999995
Homo sapiens cDNA FLJ13355 fis, clone PLACE1000048. [AK			Hs.719629	55338	2929.5654
Homo sapiens zinc fi	GO:0008150|GO:000	NM_198581	Hs.190477	376940	26.318174
Homo sapiens family	GO:0005622	NM_173822	Hs.24701	285172	160.13036
Homo sapiens thioredoxin reductase 3 intr		NM_001039783	Hs.477475	645840	7.2727294
Homo sapiens tripept	GO:0005737|GO:000	NM_003291	Hs.432424	7174	303.8825
Homo sapiens ankyrin repeat domain 26 (A		NM_014915	Hs.361041	22852	4.9999995
Homo sapiens zinc finger protein 204 pseu		NR_002722	Hs.8198	7754	21.181007
Homo sapiens G prot	GO:0007165|GO:000	NM_005284	Hs.46332	2830	4.9999995
Homo sapiens macro	GO:0016020|GO:001	NM_001039396	Hs.709439	219972	4.9999995
					4.9999995
Homo sapiens cDNA FLJ16364 fis, clone THYMU2032976. [AK131345]					24.885916
Homo sapiens MYST	GO:0016564|GO:007	NM_012330	Hs.35758	23522	19.57776
Homo sapiens ST3 b	GO:0030173|GO:000	NM_174963	Hs.597915	6487	9.916216
Homo sapiens clone FLB4246 PRO1102 mRNA, complete cds.			Hs.499496	100133130	6.348145
Homo sapiens crystal	GO:0005515|GO:000	NM_004076	Hs.533022	1417	4.9999995
Homo sapiens MAX	GO:0006355|GO:000	NM_001080541	Hs.187569	23269	61.151226
Homo sapiens chrom	GO:0006355|GO:000	NM_013329	Hs.644004	94104	65.92962
Homo sapiens ankyrin repeat domain 36 (A		NM_001164315	Hs.646318	375248	744.524
Homo sapiens acyl-C	GO:0006629|GO:000	NM_001609	Hs.81934	36	234.10164
Homo sapiens histon	GO:0005667|GO:003	NM_058177	Hs.196054	9734	4.9999995
Homo sapiens media	GO:0046966|GO:000	NM_004229	Hs.407604	9282	451.49564
Homo sapiens glycer	GO:0005743|GO:000	NM_020918	Hs.42586	57678	307.91287
Homo sapiens RAS p	GO:0051056|GO:000	NM_002890	Hs.664080	5921	313.9989
Homo sapiens centro	GO:0007094|GO:004	NM_016343	Hs.497741	1063	108.93959
Homo sapiens similar to Uncharacterized p		NM_001135865	Hs.720286	100132247	12409.992
					4.9999995
Homo sapiens ATPas	GO:0000287|GO:003	NM_006095	Hs.435052	10396	22.905088
Homo sapiens mitoge	GO:0000287|GO:001	NM_004721	Hs.656069	9175	7.29584
PREDICTED: Homo sapiens similar to hC		XM_001721247	Hs.434448	100131635	10.16293
Homo sapiens zinc fi	GO:0016566|GO:000	NM_032494	Hs.418416	84524	23.525139
F-box only protein 11 (Vitiligo-associated protein 1)(VIT-1) [Source:UniProtKB/Swiss-Prot;Acc:Q86XK2]					13.66487
Homo sapiens phospl	GO:0005515|GO:000	NM_152671	Hs.720192	200576	26.130901
					4.9999995
Homo sapiens COP9	GO:0005515|GO:000	NM_004236	Hs.369614	9318	1144.5444
AGENCOURT_7904926 NIH_MGC_82 Homo sapiens cDNA c			Hs.650636		158.90816
Homo sapiens recept	GO:0005515|GO:000	NM_005669	Hs.429608	7905	4130.174
Homo sapiens zinc fi	GO:0005622|GO:000	NM_006954	Hs.435774	7581	120.84881
Homo sapiens bromo	GO:0005515|GO:000	NM_018963	Hs.654740	54014	17.258091
Homo sapiens DEAD/H (Asp-Glu-Ala-Asp		NM_182540	Hs.496829	203522	40.27168
Homo sapiens zinc fi	GO:0005622|GO:000	NM_144684	Hs.147025	147657	32.245018
Homo sapiens Meis h	GO:0043565|GO:000	NR_002211	Hs.532082	4213	1195.492
Homo sapiens ash1 (	GO:0005515|GO:003	NM_018489	Hs.491060	55870	169.5322
Homo sapiens ubiquitin-conjugating enzym		NR_004847	Hs.641964	100134869	26.668615
Homo sapiens zinc fi	GO:0005622|GO:000	NM_152355	Hs.675132	126068	5.88141
Homo sapiens BH3-l	GO:0005739|GO:000	NM_001001786	Hs.686109	414899	18.35103
					4.9999995
THUMP domain-containing protein 2 [Source:UniProtKB/Swis			Hs.468254		9.369702
Homo sapiens DEAD	GO:0005515|GO:000	NM_001356	Hs.380774	1654	818.2399
Homo sapiens family with sequence simila		NM_024581	Hs.443789	79632	14.646959
					201.28378
Homo sapiens mesod	GO:0005634|GO:000	NM_152622	Hs.657594	166968	14.428941
Homo sapiens hypothetical protein FLJ100		NR_026891		55056	16.799438
Homo sapiens kinase	GO:0007242|GO:001	NM_020738	Hs.9873	57498	44.109615
Homo sapiens structu	GO:0005515|GO:005	NM_015295	Hs.8118	23347	512.27185
Homo sapiens pseud	GO:0016853|GO:000	NM_144709	Hs.368348	150962	14.300341
Putative CNGA1-overlapping antisense gene protein (Anti-CNG			Hs.686897		5.592717
Homo sapiens zinc fi	GO:0005622|GO:000	NM_001007101	Hs.668378	83744	32.84016
Homo sapiens ArfGA	GO:0005622|GO:000	NM_015230	Hs.479451	116984	108.74051
Homo sapiens chromosome 12 open readir		NM_018169	Hs.445129	55196	154.81969

20.911467	2.3362613	4.2505054
12562.588	11.597727	13.603358
75.45792	4.8522997	6.1118727
506.16577	7.4555845	8.834666
18.375029	3.0350444	4.069147
794.4654	8.403173	9.492972
35.238857	2.3362613	4.996889
81.39006	4.537633	6.2177744
15.825909	2.3362613	3.8441763
43.458282	2.3362613	5.304904
13.40276	2.3362613	3.5940802
201.86101	4.774454	7.5193434
79.05991	4.425003	6.1781282
63.430378	3.4650755	5.8632517
34.426937	2.8498652	4.9644356
14.2078905	2.3362613	3.6833706
186.95917	6.057521	7.4142394
168.1622	6.1696434	7.259197
3085.7346	9.686085	11.514749
755.6248	8.02627	9.420771
49.42904	2.3362613	5.5027814
1187.3265	8.970311	10.090811
862.87964	8.425304	9.60597
1093.603	8.44915	9.971338
727.7771	6.8981304	9.367205
27857.176	13.611692	14.792375
20.099388	2.3362613	4.1939964
77.34427	4.6524997	6.145743
19.555876	3.039534	4.153678
48.972507	3.5004103	5.486379
72.842155	4.6973677	6.0628834
38.786625	3.9117277	5.1403866
85.74955	4.843401	6.2919903
12.7146	2.3362613	3.5139527
3460.9475	10.278187	11.687923
409.87872	7.4454765	8.521769
12268.896	12.076601	13.568145
400.5363	7.045333	8.49032
94.032684	4.24545	6.4198475
100.87471	5.461843	6.5212107
173.5208	5.1472692	7.306019
3265.4846	10.337065	11.593445
697.3217	7.544444	9.30847
95.77564	4.8781657	6.4435005
38.359924	2.7471986	5.1256647
84.287476	4.329856	6.2684975
12.064621	2.3362613	3.4346209
34.056496	3.389997	4.947863
2903.8242	9.816456	11.421107
38.58248	4.005279	5.1326547
553.9735	7.8040543	8.960763
41.380486	3.985626	5.237032
43.32688	4.2062855	5.3006573
199.40897	5.5881424	7.5020924
2175.611	9.145115	10.992477
45.821445	3.968934	5.386636
26.505398	2.6747794	4.5957384
110.41711	5.17194	6.6522245
326.05258	6.89624	8.1955595
729.0015	7.406662	9.369755

A_24_P26073	2.5728601	down	NM_133259	chr2:044117011-044	LRPPRC
A_23_P334218	3.0441976	down	NM_145647	chr8:124164161-124	WDR67
A_33_P3256695	3.7336793	down	NM_017892	chr2:153508166-153	PRPF40A
A_23_P333138	3.8049988	down	NM_015026	chr12:062990309-06	MON2
A_33_P3302861	2.9778346	down	NM_015272	chr16:053633893-05	RPGRIP1L
A_32_P99055	2.5499156	down	NM_020922	chrX:054223592-05	WNK3
A_33_P3349474	2.1338688	down		chr2:53759872-53759813	
A_23_P58877	2.1895555	down	NM_020399	chr6:117881675-117	GOPC
A_33_P3377744	4.2376317	down	XM_001720949	chr6:104242172-104	FLJ10088
A_23_P165574	2.1470589	down	NM_017969		IWS1
A_33_P3337981	2.466572	down	NM_014845	chr6:110098202-110	FIG4
A_23_P310086	2.4498703	down	NM_152731	chr6:056891269-056	BEND6
A_24_P234792	2.4565524	down	NM_001044723	chr5:122952072-122	CSNK1G3
A_33_P3289371	2.5407748	down	BC036834	chr20:58411414-584	PHACTR3
A_23_P76374	4.1848407	down	NM_153694		SYCP3
A_24_P680908	2.1638643	down	NM_021269	chr19:021474065-02	ZNF708
A_32_P152437	2.4801906	down	NM_005100	chr6:151679428-151	AKAP12
A_23_P158997	3.881539	down	XM_002342180	chr1:247353404-247	LOC729806
A_33_P3283231	2.012681	down	AK091850	chrX:21876702-218	YY2
A_24_P198629	2.2989019	down	NM_018148	chr15:101109837-10	LINS1
A_24_P109071	3.1735831	down	NM_017576	chr9:086451820-086	KIF27
A_23_P65789	2.9134386	down	NM_018349	chr15:095023163-09	MCTP2
A_23_P18684	2.475277	down	NM_004362	chr4:141310434-141	CLGN
A_33_P3314623	2.4325887	down		chr3:155755416-155755475	
A_33_P3269588	2.2441825	down	NM_002040	chr21:027144188-02	GABPA
A_23_P70387	3.5027138	down	NM_006789	chr6:041032145-041	APOBEC2
A_33_P3269598	3.3348896	down		chr9:098734804-098734863	
A_33_P3382910	2.4016671	down	NM_017676	chr5:102440372-102	GIN1
A_24_P897062	2.3150019	down	XM_001128828	chr7:63809153-6380	LOC728927
A_33_P3762918	2.7727995	down	AK123595	chr7:104622285-104	LOC100216546
A_23_P159255	2.1541743	down	NM_002845	chr18:008406398-00	PTPRM
A_23_P16762	2.9017194	down	NM_017892	chr2:153512882-153	PRPF40A
A_33_P3260377	3.0037614	down	NM_031461	chr8:075946730-075	CRISPLD1
A_23_P203173	2.1589438	down	NM_001558	chr11:117872070-11	IL10RA
A_23_P385938	2.0108661	down	NM_175854	chr13:028868784-02	PAN3
A_23_P213199	2.3142553	down	NM_001031723	chr4:100822095-100	DNAJB14
A_33_P3397323	3.6032873	down	NM_152355	chr19:011894786-01	ZNF441
A_23_P373927	2.5548181	down	NM_015051	chr9:102741776-102	ERP44
A_23_P147199	2.8897444	down	NR_024565	chr18:032887664-03	ZNF271
A_33_P3325502	2.9788356	down	NM_004815	chr1:094634524-094	ARHGAP29
A_33_P3405980	5.9610551	down	NM_001032377	chr2:037402361-037	SULT6B1
A_23_P334173	2.1470384	down	NM_002349	chr2:160660247-160	LY75
A_33_P3402188	2.71843	down	AF116728	chr12:11230356-112	TAS2R19
A_23_P43150	2.4131763	down	NM_001017926	chr8:124261498-124	ZHX1
A_33_P3780901	3.170808	down	AK092518	chr12:123776783-12	LOC283378
A_33_P3383261	2.0246717	down	NM_001102396	chr1:115316203-115	SIKE1
A_23_P396328	2.3341394	down	NM_003829	chr9:013105942-013	MPDZ
A_33_P3364089	2.0922707	down	NM_019844	chr12:021036476-02	SLCO1B3
A_24_P481375	2.4862476	down	AL832786	chr1:93803884-9380	LOC100131564
A_33_P3326225	3.3918867	down	NM_001101676	chr8:119201758-119	SAMD12
A_33_P3375140	2.4997988	down	NM_024596	chr8:006505948-006	MCPH1
A_23_P317324	2.9161006	down	NM_005241	chr3:168806898-168	MECOM
A_33_P3334826	2.1579903	down		chr12:049783707-049783766	
A_23_P14708	3.6437715	down	NM_017661	chr15:056923077-05	ZNF280D
A_23_P393880	2.1958425	down	NM_020340	chr6:138659313-138	KIAA1244
A_24_P405430	2.871142	down	NM_022173	chr2:070436757-070	TIA1
A_33_P3334743	2.9509188	down	NM_014247	chr4:160263014-160	RAPGEF2
A_33_P3374205	4.0853212	down	NM_002417	chr10:129913252-12	MKI67
A_23_P139687	3.5620795	down	NM_152321	chr12:015067229-01	ERP27
A_33_P3330712	2.2791154	down	NR_024504	chr11:050379413-05	LOC646813

Homo sapiens leucin	GO:0042645|GO:000	NM_133259	Hs.368084	10128	2791.5576
Homo sapiens WD re	GO:0005622|GO:003	NM_145647	Hs.492716	93594	481.85797
Homo sapiens PRP4(	GO:0008380|GO:000	NM_017892	Hs.643580	55660	53.65759
Homo sapiens MON2	GO:0005488|GO:001	NM_015026	Hs.389378	23041	4.9999995
Homo sapiens RPGR	GO:0007368|GO:002	NM_015272	Hs.298382	23322	12.885679
Homo sapiens WNK	GO:0005515|GO:000	NM_020922	Hs.92423	65267	13.008173
Ankyrin repeat and SOCS box protein 3 (ASB-3) [Source:UniProtKB/Swiss-Prot;Acc:Q9Y575] [ENST000					25181.842
Homo sapiens golgi a	GO:0050430|GO:000	NM_020399	Hs.191539	57120	1063.4769
PREDICTED: Homo sapiens similar to hy		XM_001720949	Hs.458459	389419	9.460898
Homo sapiens IWS1	GO:0005634	NM_017969	Hs.469879	55677	197.32053
Homo sapiens FIG4 I	GO:0030384|GO:000	NM_014845	Hs.529959	9896	15.080151
Homo sapiens BEN domain containing 6 (		NM_152731	Hs.582993	221336	18.22051
Homo sapiens casein	GO:0007165|GO:000	NM_001044723	Hs.129206	1456	378.93195
Homo sapiens phospl	GO:0004864|GO:0005634|GO:0003779|G		Hs.473218	116154	4.9999995
Homo sapiens synapt	GO:0007049|GO:000	NM_153694	Hs.506504	50511	4.9999995
Homo sapiens zinc fi	GO:0005622|GO:000	NM_021269	Hs.466296	7562	6.458207
Homo sapiens A kina	GO:0007165|GO:000	NM_005100	Hs.371240	9590	130.66862
PREDICTED: Homo sapiens similar to hC		XM_002342180	Hs.635482	729806	16.404512
Homo sapiens cDNA	GO:0005622|GO:0008270|GO:0005634|G		Hs.673601	404281	174.32098
Homo sapiens lines homolog 1 (Drosophila		NM_018148	Hs.105633	55180	22.491526
Homo sapiens kinesi	GO:0000166|GO:000	NM_017576	Hs.697514	55582	40.79048
Homo sapiens multip	GO:0005544|GO:001	NM_018349	Hs.33368	55784	4.9999995
Homo sapiens calme	GO:0007338|GO:001	NM_004362	Hs.86368	1047	694.6285
GB					4.9999995
Homo sapiens GA bi	GO:0043565|GO:000	NM_002040	Hs.473470	2551	232.55202
Homo sapiens apolip	GO:0006397|GO:001	NM_006789	Hs.555915	10930	4.9999995
					79.940445
Homo sapiens gypsy	GO:0015074|GO:000	NM_017676	Hs.24088	54826	30.281742
cDNA FLJ57041, moderately similar to Zi		XM_001128828	Hs.535881	728927	140.60403
Homo sapiens cDNA FLJ41601 fis, clone CTONG2027327 [AK			Hs.369356	100216546	97.76464
Homo sapiens protei	GO:0031290|GO:000	NM_002845	Hs.49774	5797	511.25427
Homo sapiens PRP4(	GO:0008380|GO:000	NM_017892	Hs.643580	55660	3171.2554
Homo sapiens cystei	GO:0005576	NM_031461	Hs.436542	83690	5.107056
Homo sapiens interle	GO:0005515|GO:000	NM_001558	Hs.504035	3587	4.9999995
Homo sapiens PAN3	GO:0005737|GO:000	NM_175854	Hs.645015	255967	434.28116
Homo sapiens DnaJ (	GO:0016020|GO:000	NM_001031723	Hs.577426	79982	254.1509
Homo sapiens zinc fi	GO:0005622|GO:000	NM_152355	Hs.675132	126068	46.057995
Homo sapiens endop	GO:0006986|GO:000	NM_015051	Hs.154023	23071	39.62038
Homo sapiens zinc finger protein 271 (ZN		NR_024565	Hs.314246	10778	182.06584
Homo sapiens Rho G	GO:0005622|GO:000	NM_004815	Hs.483238	9411	162.54341
Homo sapiens sulfotr	GO:0005737|GO:000	NM_001032377	Hs.631892	391365	4.9999995
Homo sapiens lymph	GO:0006955|GO:000	NM_002349	Hs.153563	4065	27.949516
Homo sapiens MSTP	GO:0007165|GO:0050909|GO:0016020|G		Hs.687025	259294	16.63024
Homo sapiens zinc fi	GO:0043565|GO:000	NM_001017926	Hs.612084	11244	162.35371
Homo sapiens cDNA FLJ35199 fis, clone PLACE6018031 [AK			Hs.594149	283378	252.34184
Homo sapiens suppre	GO:0005737	NM_001102396	Hs.709277	80143	491.7626
Homo sapiens multip	GO:0005515|GO:003	NM_003829	Hs.169378	8777	76.063034
Homo sapiens solute	GO:0005215|GO:000	NM_019844	Hs.504966	28234	156.3196
Homo sapiens mRNA; cDNA DKFZp667D2123 (from clone DK			Hs.648647	100131564	64.80964
Homo sapiens sterile alpha motif domain c		NM_001101676	Hs.359393	401474	74.37886
Homo sapiens micro	GO:0005622|GO:000	NM_024596	Hs.708770	79648	20.900259
Homo sapiens MDS1	GO:0005622|GO:000	NM_005241	Hs.656395	2122	286.98892
					41.96861
Homo sapiens zinc fi	GO:0005622|GO:000	NM_017661	Hs.511477	54816	36.252125
Homo sapiens KIAA	GO:0005622|GO:000	NM_020340	Hs.194408	57221	53.96903
Homo sapiens TIA1	GO:0042036|GO:001	NM_022173	Hs.413123	7072	126.03446
Homo sapiens Rap g	GO:0005515|GO:005	NM_014247	Hs.113912	9693	10.306209
Homo sapiens antige	GO:0005622|GO:000	NM_002417	Hs.80976	4288	67.2746
Homo sapiens endop	GO:0005783|GO:000	NM_152321	Hs.162143	121506	4.9999995
Homo sapiens hypothetical LOC646813 (L		NR_024504	Hs.684179	646813	8.90402

7755.619	11.526367	12.88974
1751.4047	9.058749	10.664811
241.69681	5.869636	7.770234
21.136375	2.3362613	4.2641573
46.452366	3.8286934	5.402957
40.069588	3.8413067	5.1917562
52863.953	14.61022	15.7036915
2694.5225	10.174328	11.304966
48.930378	3.4019198	5.485178
518.9779	7.7710752	8.873437
44.563637	4.0429583	5.3454657
53.25516	4.320141	5.6128464
1127.5131	8.721541	10.018176
14.184461	2.3362613	3.6815298
23.204062	2.3362613	4.401434
17.359419	2.869766	3.983376
396.10834	7.163166	8.473617
76.354996	4.1717377	6.1283665
429.911	7.5829115	8.59203
61.83698	4.6263714	5.8273163
154.88904	5.4787154	7.144828
16.21716	2.3362613	3.8789842
2042.65	9.582245	10.889835
13.612411	2.3362613	3.6187537
641.9452	8.017886	9.184076
19.430428	2.3362613	4.1447344
326.40906	6.459055	8.196694
87.83257	5.0597296	6.3237658
397.9728	7.2697506	8.480764
330.66257	6.7433157	8.214659
1319.2693	9.142537	10.249672
9739.757	11.697389	13.234297
19.311592	2.5495548	4.136325
12.15461	2.3362613	3.446587
1061.6571	8.911821	9.919638
721.73486	8.147015	9.357563
198.84624	5.649766	7.4990797
121.38771	5.4384604	6.791681
641.92694	7.6531096	9.1840515
587.70984	7.4788275	9.053576
33.22844	2.3362613	4.911829
72.15803	4.947064	6.049412
54.207687	4.190775	5.6335487
478.33496	7.4777927	8.748726
984.8192	8.1367855	9.801636
1198.1589	9.088352	10.10604
214.66342	6.384267	7.6071577
399.39862	7.4211025	8.486172
192.82475	6.1454206	7.4593906
309.28964	6.352812	8.1149
62.413826	4.518438	5.84025
1025.4103	8.3196125	9.863653
108.60851	5.5156736	6.625362
159.44147	5.3160295	7.181462
141.08221	5.879242	7.0140166
442.34967	7.1124973	8.634122
37.303944	3.5204284	5.0815926
334.14795	6.2011595	8.231609
19.752192	2.3362613	4.168981
24.86422	3.316794	4.505268

A_33_P3368555	2.1912622	down	BC050395	chr2:61415552-61415611
A_23_P29855	2.1857782	down	NM_003715	chr4:076734902-076 USO1
A_24_P822931	2.5858991	down	BC034811	chrX:111146766-111146825
A_24_P144881	3.7768733	down	NM_005977	chr13:026793657-02 RNF6
A_24_P419177	2.0719885	down	NM_014497	ZNF638
A_33_P3268919	2.4297531	down	NM_001098668	chr10:081315673-08 SFTPA2
A_24_P341089	2.1129388	down	NR_015424	chr2:089100699-089 FLJ40330
A_23_P363399	2.1058549	down	NM_030674	chr12:046582066-04 SLC38A1
A_24_P280378	2.1237913	down	NM_014691	AQR
A_33_P3390008	2.6228013	down		chr22:22556436-22556495
A_33_P3270019	2.5474627	down	NM_006540	chr8:071036274-071 NCOA2
A_33_P3214879	2.7740183	down	NM_002264	chr3:122140844-122 KPNA1
A_33_P3226202	3.325752	down	NM_015091	chr14:045543533-04 FAM179B
A_23_P331943	2.1976643	down	NM_020823	chr6:159055923-159 TMEM181
A_23_P75071	4.9107702	down	NM_016195	chr10:091534055-09 KIF20B
A_23_P39814	2.5325452	down	NM_004882	chr2:175213308-175 CIR1
A_33_P3542886	2.6596603	down	BU536871	SNAR-G1
A_24_P46953	2.4180489	down	NM_013257	chr8:067773851-067 SGK3
A_23_P339687	2.622504	down	NM_138330	chr19:023836893-02 ZNF675
A_23_P350187	2.8134227	down	NM_001127208	chr4:106200570-106 TET2
A_23_P134925	2.0150393	down	NM_004331	chr8:026270286-026 BNIP3L
A_33_P3236703	2.9833162	down	XM_001718334	chr9:33568831-33568890
A_23_P300867	2.37636	down	NM_138731	MIPOL1
A_33_P3331437	2.3184429	down	NM_014803	chr10:097923206-09 ZNF518A
A_33_P3713357	2.773241	down	NM_001627	chr3:105294613-105 ALCAM
A_23_P204736	2.1204794	down	NM_005276	chr12:050504625-05 GPD1
A_23_P254594	3.1879191	down	NM_000825	chr8:025276966-025 GNRH1
A_33_P3310232	2.4135672	down	NM_024884	chr14:050710535-05 L2HGDH
A_33_P3295543	3.0205004	down	DA825750	chr4:121086981-121087040
A_24_P159702	2.4661417	down	NM_005570	chr18:056997928-05 LMAN1
A_33_P3314436	2.5213212	down	NM_001163315	chr5:107356686-107 FBXL17
A_23_P400449	8.3832055	down	NM_020927	chr16:078013635-07 VAT1L
A_33_P3357322	4.1255058	down	NM_001042550	chr9:106903624-106 SMC2
A_33_P3396469	2.0962198	down		chr20:59339522-59339581
A_33_P3265564	2.563519	down	XM_001714497	chr8:049826771-049 LOC644334
A_33_P3243667	3.7746778	down	NM_178549	chr1:227843936-227 ZNF678
A_23_P424002	2.2206931	down	NM_002697	chr1:167385244-167 POU2F1
A_23_P398449	2.5174074	down	NR_028291	chr6:133055517-133 VNN3
A_23_P144677	3.112988	down		chr5:96372835-9637 LNPEP
A_33_P3331911	2.0900446	down	XR_039365	chr16:022418699-02 LOC100129767
A_33_P3340639	2.3466572	down		chr10:113913749-113913690
A_23_P168240	2.3700213	down	NM_003913	chrX:115882820-11: PRPF4B
A_33_P3628409	2.3864473	down	AK125369	chr2:217084790-217084849
A_23_P17012	2.1441696	down	NM_024583	chr2:175293369-175 SCRN3
A_24_P913115	2.084683	down	NM_000314	chr10:089727925-08 PTEN
A_23_P216935	2.2267963	down	NR_026677	chr9:125872202-125 C9orf45
A_33_P3366431	3.8817325	down	NM_002297	chr9:136101557-136 LCN1
A_32_P193322	3.4853511	down	NM_152756	chr5:038938366-038 RICTOR
A_33_P3378689	2.4656175	down	NM_016628	chr10:028905152-02 WAC
A_32_P117170	2.1239105	down	NM_001122838	chr7:102740667-102 NAPEPLD
A_33_P3411907	2.0949497	down	NM_033143	chr4:081188093-081 FGF5
A_33_P3333900	2.159967	down	AK096041	chr4:148653608-148 LOC100129572
A_23_P152066	2.383917	down	NM_174916	chr15:043235340-04 UBR1
A_23_P136964	2.0227156	down	NM_000328	chrX:038128962-038 RPGR
A_23_P145437	5.9237346	down	NM_017934	chr6:079650509-079 PHIP
A_24_P123720	3.1323091	down	NM_007214	chr6:108189385-108 SEC63
A_33_P3363500	2.7409105	down		chr22:022645324-022645265
A_33_P3354499	2.0272823	down	XR_040305	chr8:142400328-142 LOC401480
A_23_P52017	5.5904096	down	NM_018136	chr1:197053513-197 ASPM
A_24_P923757	2.6553328	down	NM_018179	chr12:014577680-01 ATF7IP

Activator of 90 kDa heat shock protein ATPase homolog 2 [Sou			Hs.655602		11.121759
Homo sapiens USO1	GO:0005515|GO:000	NM_003715	Hs.292689	8615	1581.6372
Homo sapiens cDNA clone IMAGE:4822062 [BC034811]			Hs.247868		4.9999995
Homo sapiens ring fi	GO:0005515|GO:000	NM_005977	Hs.136885	6049	11.55691
Homo sapiens zinc fi	GO:0008380|GO:000	NM_014497	Hs.434401	27332	83.30901
Homo sapiens surfac	GO:0007585|GO:000	NM_001098668	Hs.535295	729238	4.9999995
Homo sapiens hypothetical LOC645784 (F		NR_015424	Hs.105323	645784	281.34512
Homo sapiens solute	GO:0015804|GO:000	NM_030674	Hs.533770	81539	2242.0664
Homo sapiens aquari	GO:0008380|GO:000	NM_014691	Hs.510958	9716	348.4049
V4-6 protein Fragment [Source:UniProtKB/TrEMBL;Acc:Q5NV87] [ENST00000390286]					4.9999995
Homo sapiens nuclea	GO:0016564|GO:000	NM_006540	Hs.446678	10499	4.9999995
Homo sapiens karyo	GO:0005515|GO:000	NM_002264	Hs.161008	3836	68.02507
Homo sapiens family	GO:0005488	NM_015091	Hs.371078	23116	146.98521
Homo sapiens transm	GO:0016020|GO:001	NM_020823	Hs.99145	57583	1162.5752
Homo sapiens kinesi	GO:0007088|GO:000	NM_016195	Hs.240	9585	126.791885
Homo sapiens corepr	GO:0008380|GO:000	NM_004882	Hs.632531	9541	568.131
AGENCOURT_10224340 NIH_MGC_141 Homo sapiens cDN			Hs.621635	100126780	17.0981
Homo sapiens serum	GO:0005515|GO:000	NM_013257	Hs.613417	23678	58.03936
Homo sapiens zinc fi	GO:0005515|GO:001	NM_138330	Hs.264345	171392	1020.2548
Homo sapiens tet onc	GO:0007049|GO:000	NM_001127208	Hs.367639	54790	81.33856
Homo sapiens BCL2	GO:0008634|GO:004	NM_004331	Hs.131226	665	1038.7197
Ankyrin repeat domain-containing protein		XM_001718334	Hs.493710		14.431459
Homo sapiens mirror-image polydactyly 1		NM_138731	Hs.660396	145282	35.513935
Homo sapiens zinc fi	GO:0005622|GO:000	NM_014803	Hs.600823	9849	14.732211
Homo sapiens activa	GO:0030424|GO:000	NM_001627	Hs.591293	214	2996.623
Homo sapiens glycer	GO:0009331|GO:000	NM_005276	Hs.524418	2819	74.206505
Homo sapiens gonad	GO:0010468|GO:000	NM_000825	Hs.82963	2796	27.781012
Homo sapiens L-2-hy	GO:0005739|GO:001	NM_024884	Hs.256034	79944	17504.451
DA825750 PERIC2 Homo sapiens cDNA clone PERIC2006931			Hs.650800		7.348367
Homo sapiens lectin,	GO:0005792|GO:000	NM_005570	Hs.465295	3998	57.125763
Homo sapiens F-box	GO:0019941	NM_001163315	Hs.721914	64839	17.69965
Homo sapiens vesicl	GO:0005488|GO:001	NM_020927	Hs.461405	57687	4.9999995
Homo sapiens structu	GO:0005515|GO:000	NM_001042550	Hs.119023	10592	303.03296
Putative uncharacterized protein ENSP00000383186 Fragment [Source:UniProtKB/TrEMBL;Acc:A8MW					8.115716
PREDICTED: Homo sapiens similar to hC		XM_001714497	Hs.632062	644334	8.671775
Homo sapiens zinc fi	GO:0005622|GO:000	NM_178549	Hs.30323	339500	6.046688
Homo sapiens POU	GO:0043565|GO:001	NM_002697	Hs.493649	5451	585.8472
Homo sapiens vanin	GO:0017159|GO:000	NR_028291		55350	4.9999995
Leucyl-cystinyl aminopeptidase (Cystinyl aminopeptidase)(EC 3.4.11.3)(Oxytocinase				4012	342.2544
PREDICTED: Homo sapiens similar to rC		XR_039365	Hs.639607	100129767	47.9349
Glycerol-3-phosphate acyltransferase 1, mitochondrial Precursor (GPAT)(EC 2.3.1.15) [Source:UniProtKB					19.097527
Homo sapiens PRP4	GO:0008380|GO:000	NM_003913	Hs.159014	8899	319.01556
Putative uncharacterized protein DKFZp434H1419 Fragment [S			Hs.56876		112.62189
Homo sapiens secernin 3 (SCRN3), mRNA		NM_024583	Hs.470679	79634	18.523388
Homo sapiens phospl	GO:0051898|GO:000	NM_000314	Hs.500466	5728	272.79706
Homo sapiens chromosome 9 open reading		NR_026677	Hs.708072	81571	14.749519
Homo sapiens lipoca	GO:0005515|GO:000	NM_002297	Hs.530311	3933	4.9999995
Homo sapiens RPTO	GO:0005622|GO:000	NM_152756	Hs.407926	253260	351.61862
Homo sapiens WW d	GO:0005515|GO:001	NM_016628	Hs.435610	51322	542.31915
Homo sapiens N-acy	GO:0006629|GO:001	NM_001122838	Hs.324271	222236	46.98397
Homo sapiens fibrob	GO:0008543|GO:000	NM_033143	Hs.37055	2250	8.599288
Homo sapiens cDNA FLJ38722 fis, clone KIDNE2010084. [AK			Hs.368631	100129572	4.9999995
Homo sapiens ubiqui	GO:0005515|GO:003	NM_174916	Hs.591121	197131	506.90073
Homo sapiens retiniti	GO:0005515|GO:000	NM_000328	Hs.61438	6103	212.13655
Homo sapiens plecks	GO:0008286|GO:000	NM_017934	Hs.511817	55023	476.00522
Homo sapiens SEC6	GO:0016020|GO:000	NM_007214	Hs.26904	11231	25.468176
					10.933111
PREDICTED: Homo sapiens hypothetical		XR_040305	Hs.680571	401480	2195.467
Homo sapiens asp (al	GO:0005516|GO:000	NM_018136	Hs.121028	259266	1864.3834
Homo sapiens activa	GO:0006306|GO:000	NM_018179	Hs.714407	55729	21.60218

29.760633	3.6236079	4.75537
3869.2163	10.729748	11.857895
14.42202	2.3362613	3.7069273
52.52905	3.673709	5.5909014
208.74403	6.516986	7.568002
13.603762	2.3362613	3.617071
730.24054	8.292807	9.372058
5234.9536	11.225622	12.300028
911.4338	8.599443	9.686085
14.599678	2.3362613	3.7273698
14.2223	2.3362613	3.6853223
228.04153	6.2169523	7.6889296
591.90106	7.3285294	9.06221
2925.689	10.299664	11.435635
753.1469	7.1184587	9.414408
1704.6559	9.286497	10.627085
54.54038	4.23235	5.643592
167.70407	5.9816136	7.255457
3066.5105	10.115139	11.506084
281.28952	6.4858923	7.9782186
2435.8167	10.142061	11.152869
51.485928	3.9859264	5.5628433
101.80888	5.2853966	6.53415
41.08302	4.0135365	5.2266927
8892.785	11.628759	13.100332
189.60768	6.3482	7.4325905
107.5414	4.9393225	6.6119375
41702.3	14.0868	15.357967
27.461905	3.0505867	4.6453743
168.53128	5.9601603	7.262416
53.338326	4.2814007	5.6155806
46.49476	2.3362613	5.4037633
1509.4725	8.400127	10.444698
21.013426	3.1886263	4.2564163
27.335785	3.2806084	4.638734
28.668196	2.7840621	4.7004156
1547.4186	9.330965	10.481975
14.04718	2.3362613	3.6682
1289.3923	8.576679	10.214979
119.61785	5.705886	6.7694197
53.59134	4.3900375	5.6206446
932.0038	8.473694	9.718594
326.54242	6.942918	8.1977825
47.602036	4.3427644	5.4431834
696.7131	8.246695	9.306523
39.51978	4.01466	5.1696296
21.52495	2.3362613	4.292962
1470.4862	8.611851	10.413155
1595.3013	9.225185	10.527134
119.34268	5.678883	6.765606
22.078978	3.266427	4.3333426
12.16264	2.3362613	3.4472706
1443.1577	9.130041	10.383375
529.2046	7.8855295	8.901823
3298.679	9.041954	11.608461
96.518585	4.806678	6.4539046
36.57046	3.5994878	5.054143
4908.8276	11.193731	12.213278
11224.198	10.963034	13.445988
68.54012	4.5668716	5.9757643

A_23_P171132	2.2599947	down	NM_021783	chrX:065819703-065	EDA2R
A_32_P90483	4.0925958	down	NM_178509	chr17:053240764-05	STXBP4
A_33_P3286774	3.1722089	down		chr1:144863361-144863302	
A_24_P141736	2.513464	down	NM_006838	chr12:095909063-09	METAP2
A_23_P128201	3.8324405	down	NM_005337	chr12:054925973-05	NCKAP1L
A_32_P198330	2.0356196	down	NM_173829	chr5:064017496-064	SFRS12IP1
A_33_P3314828	5.1061882	down	XM_001717151	chr6:052630131-052	LOC647169
A_23_P48339	2.48675	down	NM_175605	chr13:021237644-02	IFT88
A_23_P371076	3.6079766	down		chr13:074387119-074387060	
A_33_P3278911	2.8080215	down	NM_032205	chr8:133837306-133	PHF20L1
A_33_P3260016	2.2833848	down	NM_152905	chr12:097346926-09	NEDD1
A_24_P388433	2.8243913	down	NM_002718	chr3:135866561-135	PPP2R3A
A_32_P191004	2.1605573	down	NM_017552	chr2:023971968-023	ATAD2B
A_33_P3222698	2.0590837	down	NM_006991	chr3:044686678-044	ZNF197
A_33_P3275968	2.4958721	down	NM_005065	chr14:081939299-08	SEL1L
A_23_P16354	2.4107023	down	NM_032825	chr19:037118399-03	ZNF382
A_33_P3414669	2.4412736	down	NM_183353	chrX:073810112-073	RLIM
A_33_P3263392	2.4067782	down	AK126149	chr8:74657259-7465	LOC729696
A_24_P201879	2.4026217	down	NM_021645	chr13:052607064-05	UTP14C
A_33_P3805085	3.170406	down	NM_133372	chr5:130979125-130	FNIP1
A_33_P3767927	2.3712619	down	NM_001018077	chr5:142660788-142	NR3C1
A_23_P58819	2.4249949	down	NM_022897	chr5:170725916-170	RANBP17
A_24_P101921	3.8307307	down		chr14:57672677-57672736	
A_23_P167081	2.5031149	down	NM_005612	chr4:057798055-057	REST
A_23_P250002	2.0155449	down	NM_020771	chr6:105176417-105	HACE1
A_23_P420610	2.2148951	down	NM_138782	chr5:072286310-072	FCHO2
A_24_P163920	2.9172736	down	NM_032228	chr11:013732254-01	FAR1
A_33_P3260941	4.1410835	down	NM_015159	chr11:073117227-07	FAM168A
A_32_P121303	2.0354856	down	NM_007172	chr14:065063718-06	NUP50
A_23_P126212	2.5153731	down	NM_022111	chr1:036204176-036	CLSPN
A_23_P158851	3.0732382	down	NM_032961	chr4:134112390-134	PCDH10
A_32_P209208	3.6009516	down	NM_032606	chr12:075669900-07	CAPS2
A_23_P60002	2.2157099	down	NM_014673	chr8:109498816-109	TTC35
A_23_P74981	2.0812914	down	NM_033213	chr1:247200908-247	ZNF670
A_23_P162279	2.2791028	down	NM_018318	chr12:028702878-02	CCDC91
A_23_P135548	2.1564451	down	NM_000110	chr1:097543959-097	DPYD
A_33_P3374952	2.1676187	down	NM_173484	chr1:044600730-044	KLF17
A_32_P19539	2.6057318	down	NM_152487	chr1:095662586-095	TMEM56
A_32_P80597	2.4748589	down	AK026192	chr4:110967485-110	ELOVL6
A_33_P3371270	2.3436563	down	NR_002323	chr22:031375241-03	TUG1
A_24_P231025	5.6544813	down	NM_033656	chr21:040562878-04	BRWD1
A_33_P3298128	4.0076747	down	NM_002223	chr12:026488383-02	ITPR2
A_33_P3357337	2.8793447	down	BC026272	chr6:100005205-100005146	
A_23_P395555	2.3197189	down	NM_001032372	chr19:044681704-04	ZNF226
A_24_P312692	2.312836	down	NM_006595	chr11:043365367-04	API5
A_24_P272967	2.2605385	down	NM_015060	chr7:032598953-032	AVL9
A_24_P413941	2.0294109	down	NM_153689	chr2:200792670-200	C2orf69
A_33_P3409337	2.0742906	down	NM_025057	chr14:074523896-07	C14orf45
A_33_P3341007	2.3822113	down	AK097452	chr15:80728714-80728655	
A_24_P168726	2.2197608	down	NM_181831	chr22:030077460-03	NF2
A_23_P16908	2.205203	down	NM_198963	chr2:039025498-039	DHX57
A_33_P3367356	3.1246771	down	AK057017	chr15:32962714-32962773	
A_24_P83379	2.6734904	down	NM_178583	chr4:085730194-085	WDFY3
A_23_P43095	2.5558827	down	NM_024721	chr8:077776697-077	ZFHX4
A_24_P235783	2.2913169	down	NM_201997	chr11:064543888-06	SF1
A_33_P3248519	3.45988	down	NM_005496	chr3:160132206-160	SMC4
A_23_P348749	2.362799	down	NM_145025	chr6:109953475-109	AKD1
A_23_P79661	2.3531349	down	NM_019044	chr2:118673442-118	CCDC93
A_24_P224998	2.7365302	down		chrX:100650477-100650536	
A_33_P3376239	2.2360881	down	NM_182931	chr7:104754468-104	MLL5

Homo sapiens ectody	GO:0005515|GO:00:	NM_021783	Hs.302017	60401	9.099693
Homo sapiens syntax	GO:0008286|GO:00(	NM_178509	Hs.35199	252983	36.070217
					5.452549
Homo sapiens methic	GO:0005515|GO:00(	NM_006838	Hs.444986	10988	174.8314
Homo sapiens NCK-:	GO:0005886|GO:00(	NM_005337	Hs.182014	3071	4.9999995
Homo sapiens SFRS	GO:0008380|GO:00(	NM_173829	Hs.69504	285672	22.97287
PREDICTED: Homo sapiens similar to glu		XM_001717151		647169	4.9999995
Homo sapiens intrafl	GO:0005515|GO:00(	NM_175605	Hs.187376	8100	138.48555
					24.616707
Homo sapiens PHD f	GO:0005515|GO:00(	NM_032205	Hs.304362	51105	54.274612
Homo sapiens neural	GO:0007067|GO:00(	NM_152905	Hs.270084	121441	1512.417
Homo sapiens protei	GO:0005515|GO:00(	NM_002718	Hs.518155	5523	480.24097
Homo sapiens ATPa:	GO:0017111|GO:00(	NM_017552	Hs.467862	54454	68.607124
Homo sapiens zinc fi	GO:0005622|GO:00(	NM_006991	Hs.157035	10168	67.57123
Homo sapiens sel-1 s	GO:0005515|GO:001	NM_005065	Hs.181300	6400	762.8277
Homo sapiens zinc fi	GO:0016564|GO:00(	NM_032825	Hs.631591	84911	12.12577
Homo sapiens ring fi	GO:0005515|GO:00(	NM_183353	Hs.653288	51132	75.21295
Homo sapiens cDNA FLJ44161 fis, clone THYMU2033070. [A			Hs.561815	729696	10.811498
Homo sapiens UTP1	GO:0007126|GO:00(	NM_021645	Hs.512963	9724	49.74348
Homo sapiens follicu	GO:0005737	NM_133372	Hs.591273	96459	127.21396
Homo sapiens nuclea	GO:0005515|GO:00(	NM_001018077	Hs.122926	2908	2768.76
Homo sapiens RAN l	GO:0005737|GO:00(	NM_022897	Hs.410810	64901	105.432594
PRO1912 [Source:UniProtKB/TrEMBL;Acc:Q9UHT0] [ENST00000391612]					7.634161
Homo sapiens RE1-s	GO:0016564|GO:00(	NM_005612	Hs.631513	5978	32.626434
Homo sapiens HECT	GO:0005622|GO:00(	NM_020771	Hs.434340	57531	201.81071
Homo sapiens FCH domain only 2 (FCHO		NM_138782	Hs.719247	115548	33.842903
Homo sapiens fatty a	GO:0005515|GO:00(	NM_032228	Hs.501991	84188	91.07858
Homo sapiens family with sequence simila		NM_015159	Hs.475334	23201	4.9999995
Homo sapiens nuclec	GO:0005515|GO:001	NM_007172	Hs.475103	10762	1975.4174
Homo sapiens claspi	GO:0006260|GO:00(	NM_022111	Hs.175613	63967	32.08447
Homo sapiens protoc	GO:0005515|GO:00(	NM_032961	Hs.192859	57575	7.787524
Homo sapiens calcyp	GO:0005509	NM_032606	Hs.407154	84698	11.7415
Homo sapiens tetratri	GO:0005783|GO:00(	NM_014673	Hs.654351	9694	605.69946
Homo sapiens zinc fi	GO:0005622|GO:00(	NM_033213	Hs.710279	93474	752.3895
Homo sapiens coiled	GO:0005794|GO:001	NM_018318	Hs.653125	55297	152.70615
Homo sapiens dihydr	GO:0050661|GO:001	NM_000110	Hs.335034	1806	369.89218
Homo sapiens Krupp	GO:0005622|GO:00(	NM_173484	Hs.567674	128209	11162.046
Homo sapiens transm	GO:0008150|GO:00(	NM_152487	Hs.483512	148534	18.454384
Elongation of very lo	GO:0005739|GO:0016747|GO:0016020|G		Hs.17519	79071	49.8192
Homo sapiens taurine upregulated 1 (non-p		NR_002323		55000	409.8359
Homo sapiens bromo	GO:0005515|GO:00(	NM_033656	Hs.654740	54014	4.9999995
Homo sapiens inositc	GO:0030424|GO:00(	NM_002223	Hs.512235	3709	65.810356
Cyclin-C (SRB11 homolog)(hSRB11) [Source:UniProtKB/Swis			Hs.430646		20.064407
Homo sapiens zinc fi	GO:0005622|GO:00(	NM_001032372	Hs.145956	7769	301.65732
Homo sapiens apopto	GO:0005737|GO:001	NM_006595	Hs.435771	8539	434.6344
Homo sapiens AVL9	GO:0016020|GO:001	NM_015060	Hs.128056	23080	128.09048
Homo sapiens chrom	GO:0005576	NM_153689	Hs.471040	205327	908.46716
Homo sapiens chromosome 14 open readir		NM_025057	Hs.644621	80127	24.688898
cDNA FLJ40133 fis, clone TESTI2012231 [Source:UniProtKB			Hs.683816		4.9999995
Homo sapiens neurof	GO:0008092|GO:004	NM_181831	Hs.187898	4771	4.9999995
Homo sapiens DEAI	GO:0008026|GO:00(	NM_198963	Hs.468226	90957	88.962555
Homo sapiens cDNA FLJ32455 fis, clone SKNMC1000010 [AI			Hs.671908		5.683113
Homo sapiens WD re	GO:0005737|GO:001	NM_178583	Hs.480116	23001	451.76642
Homo sapiens zinc fi	GO:0043565|GO:00(	NM_024721	Hs.458973	79776	51.53317
Homo sapiens splicir	GO:0005515|GO:00(	NM_201997	Hs.502829	7536	111.118095
Homo sapiens structu	GO:0005515|GO:00(	NM_005496	Hs.58992	10051	4011.6877
Homo sapiens adenyl	GO:0006139|GO:00(	NM_145025	Hs.205144	221264	5.612378
Homo sapiens coiled-coil domain containi		NM_019044	Hs.107845	54520	858.164
60S ribosomal protein L36a (60S ribosomal protein L44)(Cell migration-inducing gene 6 protein) [Source:l					52.8074
Homo sapiens myelo	GO:0002446|GO:001	NM_182931	Hs.592262	55904	369.85675

25.247007	3.3489776	4.525297
177.3972	5.3085566	7.3415728
21.739523	2.6417508	4.3072386
536.4156	7.58664	8.916317
21.265211	2.3362613	4.2745247
55.968967	4.656637	5.682105
28.412354	2.3362613	4.688508
420.72617	7.2460685	8.56033
107.589676	4.761482	6.612672
182.28671	5.886409	7.375963
3877.9146	10.671013	11.862187
1622.7345	9.053124	10.551064
177.35635	6.2297325	7.341136
166.88753	6.2084045	7.250407
2245.2478	9.720147	11.039691
35.447083	3.7390075	5.008461
223.02316	6.36845	7.656084
31.704426	3.5851965	4.8522997
141.9054	5.755597	7.0202065
491.59912	7.1226664	8.787334
7083.4126	11.510662	12.756317
311.6146	6.8482203	8.126202
36.347385	3.106814	5.0444336
98.75351	5.162051	6.4857755
501.80417	7.8091216	8.8202915
90.29003	5.212643	6.3598814
324.45596	6.6438293	8.18845
22.945179	2.3362613	4.3862696
4488.955	11.040633	12.066006
97.75676	5.1406536	6.471426
29.778994	3.1364021	4.7561617
50.918007	3.6968448	5.545223
1597.9873	9.382492	10.530261
1860.5791	9.701287	10.758766
425.74414	7.38785	8.576316
980.14703	8.684488	9.793143
24085.857	13.458187	14.574298
57.32413	4.3370194	5.718708
146.71681	5.758009	7.0653553
1158.975	8.828512	10.057273
31.4275	2.3362613	4.835656
320.92273	6.1672306	8.169996
69.0249	4.4608965	5.986637
864.1623	8.394403	9.608353
1214.7693	8.914856	10.124519
353.43884	7.1314955	8.308162
2154.1353	9.952383	10.973444
61.419636	4.7655306	5.8181486
13.345908	2.3362613	3.5885627
12.475709	2.3362613	3.4866655
238.52449	6.6119375	7.752849
22.18325	2.6962929	4.34
1449.4601	8.97084	10.3895645
157.3643	5.8102074	7.164029
310.29926	6.923687	8.119864
14698.661	12.035044	13.825766
16.67173	2.6802773	3.9207742
2372.1836	9.882323	11.116907
172.60063	5.8458347	7.2981825
1013.644	8.68422	9.845197

A_33_P3227899	2.0256821	down	NR_015361	chr9:068836390-068	LOC440896
A_33_P3305117	2.6823509	down		chr9:032444251-032444192	
A_23_P21473	2.5381028	down	NM_024491	chr3:138219364-138	CEP70
A_32_P155811	2.9040414	down	NM_012120	chr6:047594826-047	CD2AP
A_33_P3233005	2.6211961	down	NM_017643	chr17:049270257-04	MBTD1
A_33_P3423504	2.5899867	down	XM_001130425	chrX:16189035-1618	MAGEB17
A_23_P34930	2.079615	down	NM_005872	chr1:115110626-115	BCAS2
A_33_P3289422	2.2133537	down	NM_001040185	chr19:053915117-05	ZNF765
A_33_P3285018	2.5521965	down		chr19:053409388-053409329	
A_33_P3878772	2.3904882	down	NM_004972	chr9:005127355-005	JAK2
A_24_P941787	2.7907425	down	NM_003913	chr6:004065127-004	PRPF4B
A_24_P167052	2.0312897	down	NM_139312	chr10:027399712-02	YME1L1
A_33_P3297160	2.8272738	down		chr11:055956560-055956501	
A_32_P105195	2.6598216	down	NM_014829	chr5:134166478-134	DDX46
A_33_P3342481	2.0171404	down		chr15:020346394-020346335	
A_33_P3232294	3.1774596	down	NM_002223	chr12:026875390-02	ITPR2
A_32_P174908	2.1572569	down	NM_015045	chr10:088195144-08	WAPAL
A_23_P256391	5.9886408	down	NM_002078	chr3:037407741-037	GOLGA4
A_24_P72394	2.9559728	down	NM_021163	chr7:005106063-005	RBAK
A_33_P3241651	2.1143038	down	AL832596	chrX:99940070-99940011	
A_24_P56281	2.5998468	down	NM_001002259	chr12:030867985-03	CAPRIN2
A_24_P79855	2.2086927	down	AL050271	chr1:8441038-84409	DKFZp566H0824
A_33_P3305521	4.8421152	down		chr19:032847630-032847689	
A_23_P138631	4.8670244	down	NM_005445	chr2:099718927-099	SMC3
A_33_P3384617	2.1403752	down	BX114012	chr18:14255484-14255543	
A_33_P3216297	2.1357222	down	NM_001018077	chr5:142657577-142	NR3C1
A_24_P941866	3.1451131	down	NM_001013717	chr5:131796925-131	C5orf56
A_24_P177585	2.322485	down	NM_182625	chr2:017963080-017	GEN1
A_23_P357985	2.9681918	down	NM_001037866	chr2:088093643-088	RGPD6
A_23_P70007	3.0613903	down	NM_012484	chr5:162918837-162	HMMR
A_33_P3335865	2.6346969	down	NM_001006657	chr2:020110205-020	WDR35
A_33_P3375889	12.9264866	down		chr8:079578721-079578662	
A_23_P129225	2.3669404	down	NM_002420	chr15:031294001-03	TRPM1
A_23_P349083	2.0798104	down	NM_138782	chr5:072385939-072	FCHO2
A_33_P3386591	2.2321915	down		chrX:101481532-101481591	
A_33_P3349466	4.6295138	down	NM_015208	chr18:009284825-00	ANKRD12
A_23_P161686	2.5473029	down	NM_014715	chr11:128838460-12	RICS
A_33_P3210875	2.390164	down	NM_000052	chrX:077302314-077	ATP7A
A_33_P3268318	5.1268046	down		chr4:17875071-17875012	
A_24_P296254	3.5327246	down	NM_014783	chr15:032931232-03	ARHGAP11A
A_24_P173746	2.1018963	down	NM_152663		RALGPS2
A_33_P3306948	2.7818642	down	NM_002336	chr12:012269123-01	LRP6
A_33_P3367087	2.0162769	down	NM_212554	chr10:126447544-12	METTL10
A_33_P3415744	2.6735321	down	NM_025190	chr2:098121431-098	ANKRD36B
A_23_P44684	3.2147064	down	NM_018098	chr3:172538330-172	ECT2
A_24_P290502	4.8941069	down	NM_181453	chr2:109087469-109	GCC2
A_33_P3763412	4.1661916	down	AK097428	chr7:115915720-115	tcag7.929
A_33_P3223082	2.4871129	down	NM_014763	chr2:075889260-075	MRPL19
A_33_P3807593	2.4636604	down	AK093652	chrX:103367139-103	LOC286437
A_33_P3333992	4.4664035	down	AK127414	chr1:120569212-120	LOC100132495
A_33_P3409944	2.5698524	down	NM_005272	chr1:110145969-110	GNAT2
A_23_P60499	2.291849	down	NM_021224	chr9:109773650-109	ZNF462
A_33_P3387300	3.1807831	down	NM_001013438	chr3:180688091-180	FXR1
A_23_P149818	3.4119665	down	NM_080599	chr10:011962903-01	UPF2
A_24_P148151	2.1413209	down	NM_005999	chr1:231701692-231	TSNAX
A_23_P405110	2.9823802	down	NM_033313	chr1:100949903-100	CDC14A
A_33_P3397418	2.5809604	down	NM_020119	chr7:138728410-138	ZC3HAV1
A_33_P3320443	2.1544486	down	NM_001145146	chr17:043913133-04	CRHR1
A_33_P3420259	2.090968	down	NR_003287	chr12:127650951-12	LOC100008589
A_33_P3316800	3.9685822	down	NM_001621	chr7:017382763-017	AHR

Homo sapiens hypothetical LOC440896 (I	NR_015361	Hs.643596	440896		29.539362
					1751.8007
Homo sapiens centro	GO:0005515|GO:000	NM_024491	Hs.531962	80321	182.6153
Homo sapiens CD2-a	GO:0048259|GO:004	NM_012120	Hs.485518	23607	312.8966
Homo sapiens mbt do	GO:0008270|GO:000	NM_017643	Hs.656803	54799	33.75762
Putative MAGE domain-containing protein		XM_001130425	Hs.632792	645864	4.9999995
Homo sapiens breast	GO:0008380|GO:000	NM_005872	Hs.22960	10286	2183.7734
Homo sapiens zinc fi	GO:0005622|GO:000	NM_001040185	Hs.720797	91661	745.0665
					47.304367
Homo sapiens Janus	GO:0033160|GO:004	NM_004972	Hs.656213	3717	39.08627
Homo sapiens PRP4	GO:0008380|GO:000	NM_003913	Hs.159014	8899	620.12195
Homo sapiens YME1	GO:0005739|GO:001	NM_139312	Hs.499145	10730	1286.7971
					4.9999995
Homo sapiens DEAD	GO:0008380|GO:000	NM_014829	Hs.406549	9879	46.462498
					6.1905613
Homo sapiens inosito	GO:0030424|GO:000	NM_002223	Hs.512235	3709	17.937904
Homo sapiens wings	GO:0005515|GO:000	NM_015045	Hs.203099	23063	2932.9678
Homo sapiens golgi a	GO:0005802|GO:000	NM_002078	Hs.344151	2803	206.13342
Homo sapiens RB-as	GO:0016564|GO:000	NM_021163	Hs.396178	57786	20.450518
Synaptotagmin-like protein 4 (Exophilin-2)(Granuphilin) [Sourc			Hs.592224		16.94754
Homo sapiens caprin	GO:0005739|GO:003	NM_001002259	Hs.234355	65981	27.41221
Homo sapiens mRNA; cDNA DKFZp566H0824 (from clone DK			Hs.607837	54744	8.637847
					17.00706
Homo sapiens structu	GO:0046982|GO:000	NM_005445	Hs.24485	9126	1434.8999
BX114012 Soares_testis_NHT Homo sapiens cDNA clone IMA			Hs.558816		28.870579
Homo sapiens nuclea	GO:0005515|GO:000	NM_001018077	Hs.122926	2908	694.6042
Homo sapiens chromosome 5 open reading		NM_001013717	Hs.658288	441108	4.9999995
Homo sapiens Gen ho	GO:0006281|GO:000	NM_182625	Hs.467793	348654	104.62581
Homo sapiens RANB	GO:0005737|GO:000	NM_001037866	Hs.645445	729540	69.48012
Homo sapiens hyalur	GO:0005737|GO:000	NM_012484	Hs.720052	3161	353.0526
Homo sapiens WD repeat domain 35 (WD		NM_001006657	Hs.205427	57539	934.2367
					4.9999995
Homo sapiens transie	GO:0005262|GO:000	NM_002420	Hs.155942	4308	4.9999995
Homo sapiens FCH domain only 2 (FCHO		NM_138782	Hs.719247	115548	365.00204
Novel protein similar to t-complex 11 homolog (Mouse) TCP11 [Source:UniProtKB/TrEMBL;Acc:Q5H9J					4.9999995
Homo sapiens ankyri	GO:0005634	NM_015208	Hs.464585	23253	4.9999995
Homo sapiens Rho G	GO:0005515|GO:003	NM_014715	Hs.440379	9743	43.58255
Homo sapiens ATPas	GO:0005802|GO:005	NM_000052	Hs.496414	538	39.402485
Putative uncharacterized protein ENSP00000382790 [Source:UniProtKB/Swiss-Prot;Acc:A8MVM7] [ENS					18.876266
Homo sapiens Rho G	GO:0005622|GO:000	NM_014783	Hs.591130	9824	100.1991
Homo sapiens Ral Gl	GO:0005622|GO:000	NM_152663	Hs.644008	55103	72.40858
Homo sapiens low de	GO:0005515|GO:000	NM_002336	Hs.584775	4040	376.72534
Homo sapiens methy	GO:0008168|GO:000	NM_212554	Hs.706064	399818	84.04184
Homo sapiens ankyrin repeat domain 36B		NM_025190	Hs.532921	57730	225.80893
Homo sapiens epithe	GO:0005622|GO:000	NM_018098	Hs.518299	1894	614.8682
Homo sapiens GRIP	GO:0005737|GO:000	NM_181453	Hs.436505	9648	67.02682
Homo sapiens cDNA FLJ40109 fis, clone TESTI2007685 [AK0			Hs.667624	286009	21.168514
Homo sapiens mitoch	GO:0005739|GO:000	NM_014763	Hs.44024	9801	876.0063
Homo sapiens cDNA FLJ36333 fis, clone THYMU2006252 [AI			Hs.656786	286437	60.391857
Homo sapiens cDNA FLJ45506 fis, clone BRTHA2020695. [AI			Hs.689499	100132495	6.289232
Homo sapiens guanin	GO:0005886|GO:004	NM_005272	Hs.36973	2780	25.717043
Homo sapiens zinc fi	GO:0005622|GO:004	NM_021224	Hs.370379	58499	1134.3152
Homo sapiens fragile	GO:0005515|GO:000	NM_001013438	Hs.478407	8087	1326.8528
Homo sapiens UPF2	GO:0048471|GO:000	NM_080599	Hs.370689	26019	56.67433
Homo sapiens transli	GO:0043565|GO:004	NM_005999	Hs.96247	7257	405.16156
Homo sapiens CDC1	GO:0005515|GO:000	NM_033313	Hs.127411	8556	6.7973986
Homo sapiens zinc fi	GO:0005737|GO:000	NM_020119	Hs.133512	56829	951.17505
Homo sapiens cortico	GO:0007190|GO:001	NM_001145146	Hs.417628	1394	102.26144
Homo sapiens 28S ribosomal RNA (LOC1		NR_003287	Hs.552217	100008589	412.02423
Homo sapiens aryl hy	GO:0003700|GO:000	NM_001621	Hs.171189	196	58.375282

71.70773	5.0243025	6.0427103
5248.2344	10.88098	12.304478
568.8089	7.6577435	9.001494
1102.7891	8.444663	9.982725
106.58329	5.208603	6.5988283
14.441062	2.3362613	3.709206
5015.2227	11.18515	12.2414665
1954.9043	9.687002	10.833236
143.64578	5.687068	7.0388074
112.572014	5.420719	6.6780243
2052.2595	9.417641	10.89829
2987.4004	10.444698	11.467094
15.746281	2.3362613	3.8356729
147.91449	5.664439	7.0757685
15.690401	2.8186493	3.8309608
68.13616	4.299996	5.9678698
6857.2705	11.599002	12.7082
1482.8499	7.8419924	10.424221
72.2662	4.4880075	6.0516405
43.306267	4.2197523	5.299935
86.19843	4.9189234	6.29735
23.47137	3.2750683	4.418261
99.704216	4.224993	6.5006304
7711.603	10.596955	12.879995
74.16362	4.993555	6.0914187
1765.9727	9.582183	10.676907
17.430428	2.3362613	3.9893732
295.55386	6.8372917	8.052961
250.47885	6.2470307	7.816615
1305.2169	8.618143	10.23233
2852.4302	9.991077	11.388714
71.03353	2.3362613	6.0285196
13.266519	2.3362613	3.5792847
933.3133	8.664549	9.721001
12.541838	2.3362613	3.4947221
25.66588	2.3362613	4.547122
132.14806	5.5682735	6.917244
113.279526	5.432436	6.6895456
116.45362	4.3734884	6.7315483
431.492	6.7775707	8.598352
183.10788	6.311657	7.3833485
1265.7039	8.711353	10.187405
204.96396	6.529031	7.5407248
741.5627	7.97674	9.395487
2326.3943	9.40658	11.091267
399.7055	6.1958704	8.486916
106.360214	4.5369434	6.5956726
2552.5715	9.90872	11.223192
177.01732	6.0384426	7.339246
35.233818	2.8375628	4.9966764
79.533875	4.8238335	6.185519
2974.932	10.265079	11.461591
4734.6924	10.485632	12.155014
233.02852	5.9488835	7.719487
1056.9619	8.812994	9.911495
25.084612	2.9397402	4.5162044
2843.2021	10.015038	11.382946
268.9268	6.809489	7.9168077
1049.3083	8.835201	9.899372
281.35553	5.990147	7.9787707

A_23_P14464	2.2278313	down	NM_006544	chr14:057675270-05	EXOC5
A_24_P321581	3.0884314	down	NM_018018	chr12:047159136-04	SLC38A4
A_32_P117354	2.4877715	down	NM_014988	chr4:041700725-041	LIMCH1
A_32_P43826	3.5914151	down	NR_024380	chr10:042830073-04	LOC441666
A_33_P3401252	2.6544905	down	NR_003130	chr1:092109276-092	HSP90B3P
A_33_P3402495	2.9307939	down	XM_002343881	chrY:013336165-01:	LOC100287092
A_23_P34578	2.4925631	down	NM_013285		GNL2
A_24_P942002	2.7760403	down	NM_012287	chr3:194996507-194	ACAP2
A_23_P44244	2.5390767	down	NM_003069	chrX:128580725-128	SMARCA1
A_23_P328836	2.9448252	down	NM_032440	chr10:098718006-09	LCOR
A_32_P162150	2.3789089	down	NM_152787	chrX:030846272-03(	MAP3K7IP3
A_33_P3252141	2.2078146	down	NM_019022	chr18:066341020-06	TMX3
A_23_P7697	2.2247049	down	NM_003100	chr5:122165292-122	SNX2
A_23_P20683	2.683396	down	NM_014878	chr9:002804293-002	KIAA0020
A_33_P3546070	2.9734772	down	NM_016018	chr8:133859020-133	PHF20L1
A_32_P874898	4.7088723	down	BI910600	chr7:142021193-142021252	
A_33_P3300837	2.3620639	down	NM_001290	chr4:016510299-016	LDB2
A_33_P3212037	3.487375	down	AB012143	chr6:89388060-89388001	
A_23_P170959	3.0751377	down	NM_033029		LMLN
A_23_P7853	4.5843789	down	NM_007058	chr6:044150895-044	CAPN11
A_24_P139208	2.3820536	down	NM_013396	chr21:017251556-01	USP25
A_23_P365060	3.1442292	down	NM_014611		MDN1
A_24_P332862	2.6171634	down	NM_001076678	chr19:023405826-02	ZNF493
A_33_P3237403	2.6691474	down	NM_173531	chr19:021906939-02	ZNF100
A_33_P3359508	8.9623316	down		chr20:031384813-031384872	
A_32_P118372	2.6475226	down	NM_015693	chr4:128635137-128	INTU
A_33_P3374210	4.4579023	down	NM_002417	chr10:129907639-12	MKI67
A_23_P98350	2.2165256	down	NM_001165	chr11:102208356-10	BIRC3
A_33_P3262138	2.4674614	down	NM_022662	chr2:112540019-112	ANAPC1
A_33_P3725324	229.872281	down	NM_004654	chrY:014971489-01	USP9Y
A_33_P3342942	7.9577286	down	CR749827	chr2:66688955-66689014	
A_23_P8664	2.9258159	down	NM_021145	chr7:086825190-086	DMTF1
A_23_P171107	2.7413452	down	NM_033031	chrX:050094691-05(	CCNB3
A_23_P366230	2.1729064	down	NM_004690	chr6:149982442-149	LATS1
A_33_P3358541	2.3377428	down	AK097792	chr3:128336121-128336180	
A_33_P3234849	3.5504709	down	NM_007124	chr6:145157529-145	UTRN
A_33_P3230588	5.122199	down		chr9:068743660-068743719	
A_33_P3306834	3.0584326	down	NM_001039884	chr19:020507059-02	ZNF826
A_24_P940803	2.4344958	down	NM_033505	chr2:026618451-026	SELI
A_33_P3353791	2.6255719	down	NM_181501	chr5:052249249-052	ITGA1
A_33_P3233834	3.2970597	down	NM_175767	chr5:055260096-055	IL6ST
A_33_P3813818	3.6068399	down	AB088847	chr1:65765366-6576	BPA-1
A_33_P3329740	2.0133737	down		chrX:040749868-040749809	
A_24_P97687	2.4828731	down	NM_000524	chr5:063256373-063	HTR1A
A_24_P926195	2.1006881	down	NM_006699	chr1:118067852-118	MAN1A2
A_24_P627984	2.4071249	down	NM_014805	chr3:037027665-037	EPM2AIP1
A_33_P3392192	2.4735036	down	AF176921	chr8:32625320-32625379	
A_24_P323815	2.8550836	down	NM_015057	chr13:077672355-07	MYCBP2
A_33_P3413523	2.1710967	down	NM_006716	chr10:065930152-06	DBF4
A_33_P3216083	2.0433092	down	NM_173082	chr6:146209221-146	SHPRH
A_23_P57007	2.2443012	down	XM_001721104	chr20:039665854-03	PRO0628
A_23_P358221	2.0515174	down	NM_015562	chr3:196081140-196	UBXN7
A_23_P166910	4.0548569	down	NM_015268	chr3:132257167-132	DNAJC13
A_33_P3305620	2.4322002	down	CR749647	chr3:156393033-156	TIPARP
A_23_P358995	3.4866221	down	NM_015525	chr6:082880099-082	IBTK
A_33_P3320082	2.3416337	down	NM_005596	chr9:014307048-014	NFIB
A_33_P3221989	2.1090227	down	NM_001005747	chr2:152694239-152	CACNB4
A_24_P183094	2.5478406	down	NM_024524	chr3:194123543-194	ATP13A3
A_33_P3305203	3.0706886	down	XR_041539	chr14:091314296-09	LOC283588
A_33_P3267460	3.1791822	down	NM_134428	chr9:003346727-003	RFX3

Homo sapiens exocy	GO:0005737|GO:00(	NM_006544	Hs.645295	10640	275.93594
Homo sapiens solute	GO:0015171|GO:00(	NM_018018	Hs.446077	55089	11.66595
Homo sapiens LIM a	GO:0031032|GO:00(	NM_014988	Hs.335163	22998	3301.9775
Homo sapiens zinc finger protein 91 pseud		NR_024380	Hs.721896	441666	41.386272
Homo sapiens heat shock protein 90kDa b		NR_003130	Hs.591435	343477	3406.4236
PREDICTED: Homo sapiens hypothetical		XM_002343881		100287092	64.68665
Homo sapiens guanir	GO:0005622|GO:00(	NM_013285	Hs.75528	29889	2783.4224
Homo sapiens ArfGA	GO:0008060|GO:00:	NM_012287	Hs.593373	23527	124.720184
Homo sapiens SWI/S	GO:0005515|GO:00	NM_003069	Hs.152292	6594	248.27568
Homo sapiens ligand	GO:0003700|GO:00(	NM_032440	Hs.500695	84458	176.28084
Homo sapiens mitog	GO:0005622|GO:00(	NM_152787	Hs.188256	257397	348.13806
Homo sapiens thiore	GO:0016020|GO:00(	NM_019022	Hs.440534	54495	1269.2303
Homo sapiens sortin	GO:0005515|GO:00(	NM_003100	Hs.713554	6643	4431.7734
Homo sapiens KIAA	GO:0005783|GO:00(	NM_014878	Hs.493309	9933	3497.1895
Homo sapiens PHD f	GO:0005515|GO:00(	NM_016018	Hs.304362	51105	208.34976
V_segment translation product Fragment [Source:UniProtKB/T			Hs.511729		5.500079
Homo sapiens LIM d	GO:0003712|GO:00:	NM_001290	Hs.714330	9079	9.713106
mRNA-capping enzyme (HCE)(HCAP1) [Includes Polynucleoti			Hs.567378		22.523603
Homo sapiens leishm	GO:0007067|GO:00(	NM_033029	Hs.518540	89782	4.9999995
Homo sapiens calpai	GO:0005622|GO:00(	NM_007058	Hs.225953	11131	4.9999995
Homo sapiens ubiqui	GO:0006511|GO:00(	NM_013396	Hs.719972	29761	66.720695
Homo sapiens MDN	GO:0000166|GO:00	NM_014611	Hs.529948	23195	13.612031
Homo sapiens zinc fi	GO:0005622|GO:00(	NM_001076678	Hs.656558	284443	394.19107
Homo sapiens zinc fi	GO:0005622|GO:00(	NM_173531	Hs.365142	163227	219.53642
					4.9999995
Homo sapiens inturn	GO:0005515	NM_015693	Hs.391481	27152	29.639694
Homo sapiens antige	GO:0005622|GO:00(	NM_002417	Hs.80976	4288	778.60034
Homo sapiens baculo	GO:0005515|GO:00(	NM_001165	Hs.127799	330	271.57574
Homo sapiens anaph	GO:0005515|GO:00(	NM_022662	Hs.436527	64682	162.01321
Homo sapiens ubiqui	GO:0006511|GO:001	NM_004654	Hs.598540	8287	4.9999995
Homeobox protein Meis1 [Source:UniProtKB/Swiss-Prot;Acc:(			Hs.526754		4.9999995
Homo sapiens cyclin	GO:0007049|GO:00(	NM_021145	Hs.196129	9988	466.36707
Homo sapiens cyclin	GO:0007049|GO:00(	NM_033031	Hs.130310	85417	27.32026
Homo sapiens LATS	GO:0005515|GO:00(	NM_004690	Hs.716697	9113	62.954884
Homo sapiens cDNA FLJ40473 fis, clone TESTI2042806. [AK097792]					5.067229
Homo sapiens utroph	GO:0005515|GO:00:	NM_007124	Hs.133135	7402	11.106568
					4.9999995
Homo sapiens zinc fi	GO:0005622|GO:00(	NM_001039884	Hs.631635	664701	16.388493
Homo sapiens seleno	GO:0008430|GO:00:	NM_033505	Hs.189073	85465	1342.1519
Homo sapiens integri	GO:0043005|GO:00(	NM_181501	Hs.720078	3672	47.74323
Homo sapiens interle	GO:0005138|GO:00(	NM_175767	Hs.532082	3572	82.33826
Homo sapiens BPA-1 mRNA for brain peptide A1, complete cd			Hs.684094	100131534	11.512021
					1848.5881
Homo sapiens 5-hyd	GO:0060259|GO:00(	NM_000524	Hs.247940	3350	6.5251217
Homo sapiens mann	GO:0005794|GO:00(	NM_006699	Hs.435938	10905	220.19226
Homo sapiens EPM2	GO:0005783	NM_014805	Hs.28020	9852	40.0629
MSTP131 [Source:UniProtKB/TrEMBL;Acc:Q7Z2R7] [ENST			Hs.668810		631.36145
Homo sapiens MYC	GO:0005515|GO:00:	NM_015057	Hs.591221	23077	198.08585
Homo sapiens DBF4	GO:0006260|GO:00(	NM_006716	Hs.485380	10926	432.732
Homo sapiens SNF2	GO:0005515|GO:001	NM_173082	Hs.124537	257218	30.792938
PREDICTED: Homo sapiens uncharacteri		XM_001721104		100127886	18.4467
Homo sapiens UBX	GO:0005515	NM_015562	Hs.518524	26043	98.39782
Homo sapiens DnaJ (	GO:0005488|GO:00:	NM_015268	Hs.12707	23317	165.31561
TCDD-inducible pol	GO:0005515|GO:0030097|GO:0006807|G		Hs.12813	25976	43.776814
Homo sapiens inhibit	GO:0005515|GO:00(	NM_015525	Hs.306425	25998	620.42334
Homo sapiens nuclea	GO:0006260|GO:00(	NM_005596	Hs.644095	4781	30.11223
Homo sapiens calciu	GO:0005515|GO:00	NM_001005747	Hs.120725	785	5.225282
Homo sapiens ATPa	GO:0006754|GO:00(	NM_024524	Hs.529609	79572	4793.101
PREDICTED: Homo sapiens hypothetical		XR_041539	Hs.46519	283588	6.4273834
Homo sapiens regula	GO:0016564|GO:00(	NM_134428	Hs.136829	5991	5.1628284

754.07996	8.261648	9.417288
43.72711	3.688264	5.3151383
8733.951	11.754846	13.0697
177.24132	5.4960256	7.340578
9617.844	11.798491	13.206926
228.71204	6.142518	7.6938095
7480.7246	11.52055	12.83818
423.95294	7.0978155	8.570844
775.81525	8.112083	9.456387
631.1561	7.600066	9.158248
1015.97107	8.598352	9.848652
3205.7861	10.419489	11.562108
10329.168	12.16687	13.320484
9893.619	11.831867	13.255927
761.2908	7.859713	9.431864
32.658195	2.6526914	4.888073
28.181278	3.43716	4.677208
94.936226	4.628555	6.4306965
17.01938	2.3362613	3.9569123
25.380909	2.3362613	4.5329876
190.67326	6.1890697	7.4412756
51.42431	3.9071796	5.559886
1248.751	8.777154	10.165158
717.4177	7.932013	9.348392
49.334274	2.3362613	5.5001354
95.12836	5.0290413	6.4336843
3998.889	9.748495	11.90486
738.145	8.239695	9.387995
488.42023	7.4745455	8.777573
1261.9452	2.3362613	10.18095
44.145466	2.3362613	5.328618
1633.4764	9.013143	10.561982
90.775764	4.913776	6.36866
163.54207	6.098836	7.218462
14.91874	2.5344663	3.7595825
47.851162	3.6212761	5.4492865
28.505737	2.3362613	4.6930246
59.892662	4.170228	5.7830205
3691.4546	10.50493	11.788553
149.83273	5.699787	7.0924187
332.44067	6.502139	8.223319
50.053417	3.6689742	5.5197096
4180.86	10.95116	11.960775
20.181673	2.8875368	4.1995473
570.71466	7.936222	9.007084
115.831024	5.45571	6.723021
1848.2681	9.442357	10.748913
687.93225	7.776577	9.29011
1131.4478	8.903883	10.022307
75.623825	5.0840435	6.114951
49.420753	4.336383	5.5026493
245.30627	6.7528896	7.789581
813.85754	7.5066895	9.5263405
126.6916	5.575054	6.857316
2550.2559	9.418993	11.220823
85.02197	5.0510216	6.278537
13.957139	2.582375	3.6589496
12790.171	12.27844	13.627715
24.529251	2.8642955	4.4828577
20.673004	2.5645473	4.233203

A_33_P3780572	2.104453	down	AK095968	chr4:189459645-189	LOC401164
A_24_P227091	2.7385173	down	NM_004523	chr10:094414627-09	KIF11
A_24_P16730	2.4604834	down	NM_030650	chr2:176791499-176	KIAA1715
A_33_P3420048	2.3822277	down	NM_024969	chr2:166536204-166	CSRNP3
A_23_P204929	4.0592058	down	NM_016248	chr13:042897130-04	AKAP11
A_23_P133095	2.0707451	down	NM_014247	chr4:160281049-160	RAPGEF2
A_33_P3356846	2.3674484	down	NM_001010891	chr5:079284459-079	MTX3
A_24_P396327	3.6917112	down	NM_138467	chr1:075230968-075	TYW3
A_33_P3358397	3.4328902	down	NM_018176	chr4:025000598-025	LGI2
A_23_P357966	2.1400841	down	NM_052937	chr8:052730327-052	PCMTD1
A_33_P3322328	2.1888565	down	NM_001981	chr1:051820011-051	EPS15
A_24_P75158	2.0882566	down	NM_001099666	chr9:072324577-072	PTAR1
A_23_P87532	2.1802763	down	NM_001002259	chr12:030862819-03	CAPRIN2
A_24_P85942	2.5103955	down	NM_181453	chr2:109116109-109	GCC2
A_23_P337790	3.7759098	down	NM_001042683	chr6:146206540-146	SHPRH
A_24_P205045	2.5764125	down	NM_015576	chr3:055542485-055	ERC2
A_33_P3388006	3.2377606	down	NM_144718	chr3:113161839-113	CCDC52
A_32_P57057	2.3300561	down	NM_006313	chr12:062688025-06	USP15
A_23_P46396	2.2594976	down	NM_021190	chr1:097279985-097	PTBP2
A_24_P82142	2.477311	down	NM_207038	chr15:057579954-05	TCF12
A_33_P3296333	2.2055623	down	NM_001144058	chr11:132206492-13	NTM
A_24_P226210	2.2076318	down	NM_153223	chr5:122681225-122	CEP120
A_33_P3388067	2.8597416	down	NM_001159524	chr7:063680485-063	ZNF735
A_33_P3386219	2.2216219	down	NM_001033858	chr10:014976762-01	DCLRE1C
A_23_P168669	2.2180603	down	NM_021151	chr7:087027998-087	CROT
A_32_P68050	4.4002149	down	NM_012224		NEK1
A_23_P256342	2.5268027	down	NM_015132	chr7:017833194-017	SNX13
A_23_P255663	2.6143303	down	NM_024641	chr6:096056776-096	MANEA
A_33_P3210338	3.0217697	down	NM_022841	chr15:056382860-05	RFX7
A_24_P38143	3.9247972	down	NM_017651	chr6:135605470-135	AHI1
A_23_P865	4.2236023	down	NM_001013660	chr1:100174467-100	FRRS1
A_33_P3221009	2.7729867	down	AK127863	chr4:8514278-8514337	
A_33_P3287680	2.3070921	down	NM_001077657	chr3:196233970-196	C3orf43
A_23_P312246	3.3136104	down	NM_024725	chr11:096086559-09	CCDC82
A_33_P3212244	3.787678	down	NR_028045	chr12:010741667-01	KLRA1
A_24_P330633	2.2777211	down	NM_000353	chr16:071601470-07	TAT
A_23_P253446	3.2516798	down	NM_002045	chr3:115439985-115	GAP43
A_23_P88470	3.1758914	down	NM_017672	chr15:050853618-05	TRPM7
A_33_P3235004	2.023468	down	NM_005964	chr17:008377599-00	MYH10
A_24_P20120	4.1312289	down	NM_018084	chr2:055515393-055	CCDC88A
A_33_P3327961	2.6863364	down	NM_198480	chr19:052494807-05	ZNF615
A_33_P3382217	2.4152526	down		chr5:132044659-132044600	
A_33_P3210880	4.4975552	down		chrX:077267846-077267905	
A_33_P3400424	3.2728612	down	NM_198480	chr19:052496192-05	ZNF615
A_23_P92057	2.6161368	down	NM_006218	chr3:178951880-178	PIK3CA
A_23_P419213	2.6818544	down	NM_020817	chr3:113683324-113	KIAA1407
A_33_P3277659	3.9794215	down	NM_001145029	chr21:014756683-01	ANKRD30B
A_33_P3301286	2.4503316	down	NM_003478	chr11:107978373-10	CUL5
A_33_P3380311	2.8616864	down	NM_000489	chrX:076778809-07(	ATRX
A_33_P3349716	2.0945854	down	NM_001010851	chr19:052795893-05	ZNF766
A_32_P34046	2.8317449	down	NM_001017975	chr1:091727946-091	HFM1
A_33_P3219469	4.8859768	down	NM_004719	chr12:046313030-04	SFRS2IP
A_33_P3243524	4.8714113	down	NM_001164389	chr5:130771703-130	RAPGEF6
A_23_P13364	2.5405168	down	NM_005013	chr11:017351767-01	NUCB2
A_33_P3225983	4.1387325	down	XM_001714921	chr2:096548086-096	LOC100133923
A_33_P3423700	2.8224024	down	AK124002	chr4:102227201-102	LOC100131829
A_33_P3232532	2.0002489	down	NM_025191	chr1:184663261-184	EDEM3
A_23_P37623	3.0493478	down	NM_181077	chr15:034672224-03	GOLGA8A
A_33_P3347976	2.5036948	down	NM_020774	chr18:019450664-01	MIB1
A_24_P48403	2.2211372	down	NM_005433	chr18:000722336-00	YES1

Homo sapiens cDNA FLJ38649 fis, clone HHDPC2007302 [AK			Hs.435756	401164	6.174259
Homo sapiens kinesi	GO:0005871|GO:00(	NM_004523	Hs.8878	3832	593.3392
Homo sapiens KIAA	GO:0005515|GO:001	NM_030650	Hs.209561	80856	32.009384
Homo sapiens cystei	GO:0043065|GO:00(	NM_024969	Hs.470479	80034	12.303419
Homo sapiens A kina	GO:0005813|GO:00(	NM_016248	Hs.105105	11215	298.6443
Homo sapiens Rap gi	GO:0005515|GO:005	NM_014247	Hs.113912	9693	756.36145
Homo sapiens metax	GO:0005739|GO:001	NM_001010891	Hs.531418	345778	20.73505
Homo sapiens tRNA-	GO:0008168|GO:001	NM_138467	Hs.348411	127253	46.87629
Homo sapiens leucin	GO:0005515|GO:00(	NM_018176	Hs.12488	55203	4.9999995
Homo sapiens protei	GO:0004719|GO:00(	NM_052937	Hs.671268	115294	35.21052
Homo sapiens epider	GO:0005515|GO:001	NM_001981	Hs.83722	2060	204.17996
Homo sapiens protei	GO:0018346|GO:00(	NM_001099666	Hs.494100	375743	1690.8799
Homo sapiens caprin	GO:0005739|GO:003	NM_001002259	Hs.234355	65981	295.13803
Homo sapiens GRIP	GO:0005737|GO:00(	NM_181453	Hs.436505	9648	189.7303
Homo sapiens SNF2	GO:0005515|GO:001	NM_001042683	Hs.124537	257218	25.691801
Homo sapiens ELKS	GO:0005515|GO:004	NM_015576	Hs.476389	26059	7.536014
Homo sapiens coiled-coil domain containi		NM_144718	Hs.477144	152185	4.9999995
Homo sapiens ubiqui	GO:0005515|GO:00(	NM_006313	Hs.434951	9958	195.74907
Homo sapiens polyp	GO:0008380|GO:00(	NM_021190	Hs.269895	58155	591.3899
Homo sapiens transc	GO:0005515|GO:00(	NM_207038	Hs.511504	6938	634.59375
Homo sapiens neurot	GO:0005515|GO:00(	NM_001144058	Hs.504352	50863	8.511539
Homo sapiens centro	GO:0032880|GO:00(	NM_153223	Hs.483209	153241	160.97871
Homo sapiens zinc fi	GO:0005622|GO:00(	NM_001159524	Hs.723116	730291	20.168436
Homo sapiens DNA (	GO:0051276|GO:00(	NM_001033858	Hs.656065	64421	16.548044
Homo sapiens carniti	GO:0006629|GO:00(	NM_021151	Hs.125039	54677	29.641977
Homo sapiens NIMA	GO:0005515|GO:00(	NM_012224	Hs.481181	4750	19.98284
Homo sapiens sorting	GO:0005515|GO:00(	NM_015132	Hs.487648	23161	227.59148
Homo sapiens manno	GO:0005794|GO:001	NM_024641	Hs.533323	79694	72.24817
Homo sapiens regula	GO:0006355|GO:00(	NM_022841	Hs.282855	64864	177.83545
Homo sapiens Abelson helper integration s		NM_017651	Hs.386684	54806	20.969696
Homo sapiens ferric-	GO:0004500|GO:001	NM_001013660	Hs.454779	391059	4.9999995
Putative uncharacterized protein ENSP00000371928 [Source:U			Hs.518622		5.612928
Homo sapiens chrom	GO:0016020|GO:001	NM_001077657	Hs.631933	255798	25.484505
Homo sapiens coiled	GO:0005515	NM_024725	Hs.525088	79780	611.6442
Homo sapiens killer cell lectin-like recepto		NR_028045	Hs.159297	10748	4.9999995
Homo sapiens tyrosi	GO:0006559|GO:001	NM_000353	Hs.161640	6898	11.120379
Homo sapiens growtl	GO:0005516|GO:003	NM_002045	Hs.134974	2596	4.9999995
Homo sapiens transie	GO:0005515|GO:00(	NM_017672	Hs.512894	54822	55.29318
Homo sapiens myosi	GO:0030048|GO:00(	NM_005964	Hs.16355	4628	1428.9631
Homo sapiens coiled	GO:0008017|GO:00(	NM_018084	Hs.292925	55704	350.44284
Homo sapiens zinc fi	GO:0005622|GO:00(	NM_198480	Hs.368355	284370	106.3179
Kinesin-like protein KIF3A (Microtubule plus end-directed kinesin motor 3A) [Source:UniProtKB/Swiss-P					4.9999995
					5.1296563
Homo sapiens zinc fi	GO:0005622|GO:00(	NM_198480	Hs.368355	284370	13.49446
Homo sapiens phospl	GO:0048015|GO:004	NM_006218	Hs.85701	5290	262.8574
Homo sapiens KIAA1407 (KIAA1407), m		NM_020817	Hs.477159	57577	5.8799357
Homo sapiens ankyri	GO:0008150|GO:00(	NM_001145029	Hs.567889	374860	4.9999995
Homo sapiens cullin	GO:0005515|GO:003	NM_003478	Hs.440320	8065	283.53156
Homo sapiens alpha	GO:0005515|GO:00(	NM_000489	Hs.533526	546	17.974339
Homo sapiens zinc fi	GO:0005622|GO:00(	NM_001010851	Hs.439662	90321	179.2532
Homo sapiens HFM1	GO:0008026|GO:001	NM_001017975	Hs.454818	164045	4.9999995
Homo sapiens splicin	GO:0008380|GO:00(	NM_004719	Hs.210367	9169	359.3254
Homo sapiens Rap gi	GO:0005622|GO:00(	NM_001164389	Hs.483329	51735	12.0292
Homo sapiens nucleo	GO:0005793|GO:00(	NM_005013	Hs.654599	4925	766.729
PREDICTED: Homo sapiens similar to UF		XM_001714921		100133923	262.6106
Homo sapiens cDNA FLJ42008 fis, clone SPLEN2031724. [AK			Hs.657991	100131829	74.664085
Homo sapiens ER de	GO:0006986|GO:001	NM_025191	Hs.523811	80267	29.131386
Homo sapiens golgi a	GO:0005794|GO:001	NM_181077	Hs.720151	23015	50.130356
Homo sapiens mindb	GO:0005515|GO:00(	NM_020774	Hs.140903	57534	134.06848
Homo sapiens v-yes-	GO:0005515|GO:00(	NM_005433	Hs.194148	7525	673.4815

16.336012	2.8147972	3.8882425
1921.3385	9.351817	10.805212
95.30254	5.138028	6.4369698
35.54206	3.760529	5.0128403
1461.5985	8.3813095	10.402507
1860.434	9.708385	10.758535
58.371887	4.504798	5.748131
207.3744	5.6751876	7.5594773
19.04711	2.3362613	4.115685
90.78036	5.271435	6.3691025
551.962	7.8265166	8.956694
3947.128	10.823891	11.88619
791.56024	8.361445	9.485956
583.1962	7.7127953	9.04071
117.1067	4.822424	6.7392483
24.046345	3.087286	4.4526496
17.916218	2.3362613	4.0312576
559.7687	7.7586813	8.979046
1589.3699	9.3454	10.521402
1861.567	9.451653	10.760428
23.047424	3.2501845	4.391331
433.65118	7.4628134	8.605313
68.92523	4.4681044	5.9839892
44.268463	4.18295	5.3345633
79.09679	5.029481	6.1787796
106.13971	4.454986	6.59256
704.8326	7.987419	9.324732
228.82153	6.308687	7.6951284
653.54913	7.6148233	9.210217
99.44711	4.5238214	6.4964395
23.410954	2.3362613	4.4147353
19.52738	2.6803513	4.151792
70.33923	4.807395	6.0134706
2381.099	9.395487	11.123891
21.039118	2.3362613	4.257575
30.883	3.6234126	4.8110037
17.98315	2.3362613	4.0374465
210.31935	5.9113374	7.578499
3295.9673	10.590855	11.607685
1738.7686	8.607143	10.653714
348.32843	6.860662	8.286302
13.528351	2.3362613	3.6084354
29.136604	2.55596	4.725101
53.112408	3.8973463	5.6078987
844.31006	8.191675	9.579113
19.769499	2.7465794	4.1698103
22.01935	2.3362613	4.32882
856.9588	8.304533	9.59751
61.486725	4.3028164	5.819682
460.63577	7.6275473	8.694212
15.76827	2.3362613	3.8379526
2092.267	8.64373	10.932377
70.34802	3.7293262	6.013666
2288.464	9.725177	11.070299
1310.3511	8.190548	10.239737
258.18195	6.358298	7.8552217
69.96893	5.0052395	6.005419
182.12248	5.7666063	7.375107
411.53455	7.2034383	8.527497
1775.1439	9.5351925	10.686491

A_33_P3330468	3.0722338	down	AK001878	chrX:106307819-106307760	
A_32_P29118	3.7068959	down	NM_152754	chr7:084625062-084	SEMA3D
A_23_P201567	2.2365841	down	NM_018061	chr1:109243867-109	PRPF38B
A_24_P229871	2.0075494	down	BC101214	chr17:71746683-717	C17orf54
A_24_P316005	2.4772303	down	NM_014857		RABGAP1L
A_33_P3343962	2.1759925	down	AK090920	chr20:34738394-347	LOC100130373
A_33_P3388192	2.2671743	down	NM_144594	chr12:054849804-05	GTSF1
A_23_P307400	2.1533622	down	NM_138363	chr17:062533931-06	CCDC45
A_23_P319792	3.4606578	down	NM_019001	chr3:142025671-142	XRN1
A_33_P3235340	2.0543165	down	NM_006773	chr2:118589873-118	DDX18
A_33_P3369262	2.1023925	down	NM_015633	chr12:027117547-02	FGFR1OP2
A_24_P255845	2.4582276	down		chr12:016552865-016552804	
A_33_P3212615	2.1549927	down	NM_006287	chr2:188329067-188	TFPI
A_24_P303145	2.5219039	down	NM_054027	chr5:014705088-014	ANKH
A_23_P79360	2.1148703	down	NM_052946	chr2:169721381-169	NOSTRIN
A_32_P168464	2.1426898	down	NM_003688	chrX:041374317-041	CASK
A_23_P138910	2.4019163	down	NM_013264	chr11:125791100-12	DDX25
A_33_P3301915	3.0001221	down	NM_201286	chrX:055513049-05	USP51
A_24_P943358	2.3163939	down	NM_006716	chr7:087538554-087	DBF4
A_33_P3334630	2.4749531	down	NM_001128834	chrX:103047357-10	PLP1
A_33_P3223208	2.7437089	down	NM_016122	chr12:094763789-09	CCDC41
A_33_P3322348	2.1448826	down	XM_001714270	chr17:76267456-762	LOC100128942
A_23_P347468	2.5067689	down	NM_017412	chr8:028421786-028	FZD3
A_33_P3359713	2.5648343	down	CK825926	chr7:65226732-65226791	
A_23_P138805	2.268521	down	NM_012124	chr11:089935104-08	CHORDC1
A_32_P149060	2.5203921	down	NR_024092	chr21:026955240-02	C21orf71
A_24_P276102	2.3582043	down	NM_183404	chr20:035635888-03	RBL1
A_33_P3375556	4.5632276	down		chr9:068749912-068749971	
A_33_P3319231	2.1070295	down		chr10:71858227-718	AIFM2
A_23_P502470	2.8461952	down	NM_002184	chr17:015688312-01	IL6ST
A_23_P386450	3.3395958	down	NM_016218	chr5:074894691-074	POLK
A_23_P51117	2.3266061	down	NM_019002	chr2:067637076-067	ETAA1
A_33_P3252695	2.4725444	down	NM_018659	chr4:005018622-005	CYTL1
A_33_P3346498	2.337507	down	NM_130831	chr3:193333496-193	OPA1
A_24_P208045	2.22075	down	NM_025191	chr1:184660017-184	EDEM3
A_24_P236235	2.1940789	down	NM_013231	chr14:086093644-08	FLRT2
A_33_P3281716	2.0197308	down		chr2:133020552-133020610	
A_23_P73114	2.3636591	down	NM_000313	chr3:093592208-093	PROS1
A_24_P25080	2.1175455	down	NM_001024916		CBWD5
A_32_P12580	2.3400754	down	NM_001003652	chr18:045360247-04	SMAD2
A_24_P364807	2.1179259	down	NM_017839	chr16:055620157-05	LPCAT2
A_33_P3279660	2.3421817	down	XM_002347281	chrUn_gl000211:103518-103576	
A_33_P3348639	5.6923564	down	NM_004986	chr14:056146327-05	KTN1
A_23_P31858	3.8041702	down	NM_014682	chr8:053024119-053	ST18
A_24_P397247	2.9971016	down	NM_018115	chr4:076879062-076	SDAD1
A_24_P3973	2.0715993	down	NM_002137	chr7:026232975-026	HNRNPA2B1
A_32_P387648	2.6531501	down	NM_002016	chr1:152274918-152	FLG
A_33_P3345549	2.5540869	down	NM_024884	chr14:050709230-05	L2HGDH
A_33_P3587611	2.7064569	down	BC020828	chr9:35508625-3550	RPL36AP33
A_32_P171313	2.0959481	down	NM_021629	chr3:179113988-179	GNB4
A_32_P155460	2.5478331	down	NR_024089	chr21:046419455-04	NCRNA00162
A_33_P3300312	3.2938615	down	NM_007329	chr10:124373689-12	DMBT1
A_33_P3305348	3.0480414	down	NM_014939	chr18:029409305-02	KIAA1012
A_33_P3317225	2.2734776	down		chr1:248185781-248185840	
A_23_P309865	2.5635322	down	NM_152695	chrX:134496752-13	ZNF449
A_24_P942773	2.3619739	down	NM_007159	chr3:057914804-057	SLMAP
A_23_P71591	3.3839776	down	NM_017948		NOL8
A_23_P200310	2.32866	down	NM_017779	chr1:068943641-068	DEPDC1
A_23_P60565	2.0179807	down	NM_005649	chr5:178139032-178	ZNF354A
A_24_P12435	2.1735074	down	NM_181782	chr6:126251879-126	NCOA7

RNA-binding protein 41 (RNA-binding motif protein 41) [Sourc			Hs.139053		52.6036
Homo sapiens sema (	GO:0016020|GO:000	NM_152754	Hs.201340	223117	18.390602
Homo sapiens PRP38	GO:0008380|GO:000	NM_018061	Hs.342307	55119	365.2055
Homo sapiens chromosome 17 open reading frame 54, mRNA (c			Hs.464079	283982	83.117256
Homo sapiens RAB (	GO:0005622|GO:003	NM_014857	Hs.585378	9910	25.495565
Homo sapiens cDNA FLJ33601 fis, clone BRAMY2013975 [AI			Hs.570339	100130373	5.9897795
Homo sapiens gamet	GO:0005737|GO:000	NM_144594	Hs.524476	121355	4.9999995
Homo sapiens coiled	GO:0005515	NM_138363	Hs.569713	90799	2317.5576
Homo sapiens 5'-3' e:	GO:0005622|GO:000	NM_019001	Hs.435103	54464	204.52599
Homo sapiens DEAD	GO:0004004|GO:001	NM_006773	Hs.720044	8886	9418.083
Homo sapiens FGFR	GO:0005737	NM_015633	Hs.591162	26127	221.44833
					34.20288
Homo sapiens tissue	GO:0005886|GO:000	NM_006287	Hs.516578	7035	1566.5454
Homo sapiens ankylo	GO:0030500|GO:000	NM_054027	Hs.156727	56172	59.236397
Homo sapiens nitric	GO:0005515|GO:000	NM_052946	Hs.189780	115677	26.03261
Homo sapiens calciu	GO:0005516|GO:000	NM_003688	Hs.495984	8573	617.0698
Homo sapiens DEAD	GO:0003723|GO:000	NM_013264	Hs.420263	29118	4.9999995
Homo sapiens ubiqui	GO:0006511|GO:000	NM_201286	Hs.134289	158880	4.9999995
Homo sapiens DBF4	GO:0006260|GO:000	NM_006716	Hs.485380	10926	24.514061
Homo sapiens proteo	GO:0005515|GO:000	NM_001128834	Hs.1787	5354	4.9999995
Homo sapiens coiled-coil domain containi		NM_016122	Hs.279209	51134	45.00698
cDNA FLJ46600 fis, clone THYMU30471		XM_001714270	Hs.640074	100128942	4.9999995
Homo sapiens frizzle	GO:0001736|GO:000	NM_017412	Hs.40735	7976	83.627815
ik35e10.y5 HR85 islet Homo sapiens cDNA clone IMAGE:578			Hs.689932		21.642029
Homo sapiens cystei	GO:0008150|GO:000	NM_012124	Hs.22857	26973	2775.331
Homo sapiens chromosome 21 open readir		NR_024092	Hs.384586	282566	4.9999995
Homo sapiens retinol	GO:0005667|GO:000	NM_183404	Hs.207745	5933	107.059784
					6.083189
Apoptosis-inducing f	GO:0030261|GO:0050660|GO:0005741|GO:0006917|GO:0003			84883	4.9999995
Homo sapiens interle	GO:0005138|GO:000	NM_002184	Hs.532082	3572	86.10934
Homo sapiens polym	GO:0006260|GO:000	NM_016218	Hs.135756	51426	323.59106
Homo sapiens Ewing	GO:0005737	NM_019002	Hs.353022	54465	337.67627
Homo sapiens cytoki	GO:0007165|GO:005	NM_018659	Hs.13872	54360	4.9999995
Homo sapiens optic a	GO:0000287|GO:000	NM_130831	Hs.594504	4976	34.56974
Homo sapiens ER de	GO:0006986|GO:001	NM_025191	Hs.523811	80267	566.5315
Homo sapiens fibron	GO:0008150|GO:001	NM_013231	Hs.533710	23768	84.78322
					99.499916
Homo sapiens protei	GO:0004866|GO:000	NM_000313	Hs.64016	5627	758.82434
Homo sapiens COBV	GO:0000166|GO:000	NM_001024916	Hs.645337	220869	1089.8784
Homo sapiens SMAI	GO:0030618|GO:000	NM_001003652	Hs.12253	4087	189.56825
Homo sapiens lysoph	GO:0005794|GO:004	NM_017839	Hs.460857	54947	26.913399
LOC642204 protein	[Source:UniProtKB/	XM_002347281	Hs.653095		4.9999995
Homo sapiens kinect	GO:0003674|GO:001	NM_004986	Hs.509414	3895	2096.239
Homo sapiens suppre	GO:0016564|GO:000	NM_014682	Hs.655499	9705	22.94443
Homo sapiens SDA1	GO:0005515|GO:004	NM_018115	Hs.632604	55153	1012.3329
Homo sapiens hetero	GO:0005515|GO:000	NM_002137	Hs.487774	3181	6127.6274
Homo sapiens filaggi	GO:0005882|GO:003	NM_002016	Hs.654510	2312	19.43195
Homo sapiens L-2-hy	GO:0005739|GO:001	NM_024884	Hs.256034	79944	18.96758
Homo sapiens cDNA clone MGC:23914 IMAGE:4769647, com			Hs.684462	100191039	15.74398
Homo sapiens guanir	GO:0007165|GO:000	NM_021629	Hs.173030	59345	2374.2856
Homo sapiens non-protein coding RNA 16		NR_024089	Hs.534828	378825	18.856743
Homo sapiens delete	GO:0005576|GO:000	NM_007329	Hs.279611	1755	4.9999995
Homo sapiens KIAA	GO:0006888|GO:000	NM_014939	Hs.202001	22878	21.021706
Olfactory receptor 2L5 (Olfactory receptor OR1-53) [Source:UniProtKB/Swiss-Prot;Acc:Q8NG80] [ENST					6.6426935
Homo sapiens zinc fi	GO:0005622|GO:000	NM_152695	Hs.28780	203523	43.125065
Homo sapiens sarcol	GO:0005813|GO:000	NM_007159	Hs.476432	7871	2178.7803
Homo sapiens nuclec	GO:0006260|GO:000	NM_017948	Hs.442199	55035	1259.6067
Homo sapiens DEP d	GO:0005622|GO:000	NM_017779	Hs.445098	55635	2833.322
Homo sapiens zinc fi	GO:0005622|GO:000	NM_005649	Hs.484324	6940	118.44633
Homo sapiens nuclea	GO:0005622|GO:000	NM_181782	Hs.171426	135112	422.28568

193.09389	5.8413815	7.4606695
81.66057	4.3324637	6.2226753
1000.808	8.66648	9.827777
201.92213	6.514565	7.5200005
75.7795	4.808989	6.117717
16.405054	2.7721186	3.8937922
12.744398	2.3362613	3.5171566
5483.083	11.270833	12.377424
870.95575	7.8291817	9.620228
19491.416	13.2198925	14.258551
574.16266	7.945487	9.017519
101.32568	5.229514	6.5271325
3777.6096	10.716488	11.824171
177.97786	6.0110607	7.345574
65.978096	4.8398104	5.9203796
1578.5559	9.412271	10.511694
13.447152	2.3362613	3.6004472
16.676971	2.3362613	3.9212825
68.12242	4.7554684	5.967349
13.816681	2.3362613	3.6436625
147.12582	5.6135926	7.06972
12.078651	2.3362613	3.43716
256.54175	6.521156	7.846985
66.36967	4.5709314	5.929797
6798.285	11.515474	12.697226
14.06496	2.3362613	3.6699095
307.46887	6.8699913	8.10768
34.89678	2.7918324	4.981887
11.86926	2.3362613	3.4114718
300.37112	6.5654354	8.07447
1306.1542	8.4932575	10.232931
967.2785	8.555623	9.77385
13.80106	2.3362613	3.6422577
97.66129	5.2450123	6.469983
1496.4467	9.283684	10.434731
225.93637	6.541998	7.6756134
244.2642	6.7694907	7.7836537
2124.395	9.71295	10.953972
2667.1345	10.207448	11.289841
544.0743	7.711519	8.938074
68.47121	4.8916855	5.9743376
13.140119	2.3362613	3.5641143
12865.827	11.127914	13.63694
105.32557	4.6543617	6.5819435
3459.0332	10.103766	11.687334
13067.051	12.612576	13.663321
61.60426	4.4147353	5.8224416
57.754063	4.3793	5.7321076
51.096962	4.112767	5.5491724
5462.921	11.303552	12.371155
57.378098	4.3712206	5.7204914
18.18484	2.3362613	4.0560412
76.68664	4.5274725	6.135355
18.746792	2.9077415	4.0926423
131.78482	5.555875	6.914008
5620.9316	11.181198	12.421191
4775.7065	10.409344	12.168064
7152.4517	11.55006	12.76956
290.5055	7.0150576	8.02797
1106.6061	8.868502	9.988527

A_23_P389118	2.48785	down	NM_001025356	chr12:045825919-04	ANO6
A_23_P407112	2.23562	down	NM_145263	chr4:052963175-052	SPATA18
A_24_P208825	2.7953823	down	NM_018406	chr3:195474223-195	MUC4
A_33_P3240063	2.2208879	down	NR_024182	chr14:094473826-09	C14orf48
A_33_P3417745	3.1084966	down	NM_003870	chr15:091045415-09	IQGAP1
A_24_P205036	3.509691	down	NM_001001411	chr19:022362430-02	ZNF676
A_24_P873659	2.5626474	down	NR_002819	chr11:065272873-06	MALAT1
A_24_P82630	3.0246181	down	NM_015295	chr18:002804753-00	SMCHD1
A_33_P3236267	3.8217304	down	NM_172239	chr7:108580763-108	REXO1L1
A_24_P268993	2.1172821	down	NM_052971	chr5:132210265-132	LEAP2
A_33_P3343145	2.3694455	down	NM_005909	chr5:071500947-071	MAP1B
A_24_P299685	6.1804209	down	NM_198389	chr1:013940822-013	PDPN
A_33_P3261024	2.1189978	down		chr6:137520352-137520411	
A_33_P3275174	2.8623318	down	NM_001164457	chr8:007215557-007	ZNF705G
A_24_P941217	3.7908656	down	AK096323	chr2:223425440-223	SGPP2
A_24_P263703	2.0393019	down	AF318327	chr7:139077225-139077284	
A_23_P412409	3.5052856	down	NM_015172	chr1:171562050-171	BAT2D1
A_24_P278126	2.011453	down	NM_002485	chr8:090946443-090	NBN
A_33_P3373203	2.4260796	down		chrUn_gl000219:000073534-000073475	
A_33_P3344801	2.3271813	down		chr1:43396129-43396070	
A_33_P3412384	2.0795022	down	NM_002457	chr11:001080924-00	MUC2
A_33_P3370832	2.6878136	down		chr2:203589697-203589756	
A_33_P3256868	2.8796918	down	NM_001105549	chr19:053116330-05	ZNF83
A_23_P48495	2.483559	down	NM_199206	chr14:096158867-09	TCL1B
A_33_P3219770	2.0007515	down	NM_024529	chr1:193220997-193	CDC73
A_32_P115505	4.0946448	down	NM_015565	chr21:030301477-03	RNF160
A_23_P309207	2.4886287	down	NM_032679	chr19:052375475-05	ZNF577
A_23_P328642	2.6136361	down	NM_152525	chr2:202352500-202	ALS2CR11
A_33_P3294440	3.2784064	down		chr6:109629381-109629440	
A_32_P103669	2.2622024	down	NM_001012423	chr15:075565602-07	GOLGA8E
A_23_P48166	2.0907999	down	NM_002822	chr12:044187922-04	TWF1
A_23_P3302	5.235349	down	NM_018365	chr15:056721249-05	MNS1
A_33_P3344564	6.0627515	down	AK096581	chr5:37157521-37157462	
A_23_P252471	2.1098197	down	NM_000442	chr17:062400603-06	PECAM1
A_33_P3228872	2.3791671	down	NM_014472	chr10:100004047-10	C10orf28
A_32_P22338	2.2682804	down	NM_013374	chr15:023131920-02	PDCD6IP
A_23_P419038	3.1826158	down	NM_004898	chr4:056299165-056	CLOCK
A_23_P143952	2.3790558	down	NM_024548	chr3:101485649-101	CEP97
A_33_P3269019	2.2343184	down	XM_001719693	chr17:021814661-02	LOC100128559
A_32_P79190	2.7431258	down	XR_079090	chr4:49553097-49553156	
A_33_P3228072	2.3997776	down	NM_153240	chr3:132399705-132	NPHP3
A_24_P14932	5.1683479	down	NM_018338	chr3:113085121-113	WDR52
A_33_P3236426	2.2213983	down	AK131514	chr7:149901618-149	FLJ16734
A_23_P5586	3.2752999	down	NM_005791	chr2:071375141-071	MPHOSPH10
A_33_P3241541	3.1787256	down	CR936789	chr9:108314511-108314570	
A_23_P408455	2.4050268	down	NM_001104647	chr3:140698479-140	SLC25A36
A_24_P252364	2.3113756	down	NM_001037132	chr7:107788235-107	NRCAM
A_23_P373054	2.3425795	down	NM_173826	chr3:044450680-044	C3orf23
A_23_P136058	3.8499935	down	NM_001184	chr3:142168423-142	ATR
A_23_P73637	2.1306095	down	NM_031273	chrX:107224373-107	TEX13B
A_33_P3414127	3.1162945	down	NM_001012756	chr19:037004629-03	ZNF260
A_33_P3422374	2.7037523	down	NM_001010868	chr6:088075101-088	C6orf163
A_33_P3270354	2.774339	down	XM_001714341	chr11:49104590-49104649	
A_32_P517749	2.6590612	down	NM_004586	chrX:020168138-020	RPS6KA3
A_23_P71537	3.1159541	down	NM_001077204	chr8:068107632-068	CSPP1
A_33_P3330716	2.7671273	down	NM_017759	chr2:206858767-206	INO80D
A_23_P35617	3.4005026	down	NM_016341	chr10:096087933-09	PLCE1
A_33_P3327956	2.7269187	down	NM_183238	chr12:133498107-13	ZNF605
A_24_P75917	3.4885425	down	NM_182568	chr17:018509190-01	CCDC144B
A_23_P212511	2.8128693	down	NM_001042601	chr3:180327544-180	TTC14

Homo sapiens anocta	GO:0031404|GO:001	NM_001025356	Hs.505339	196527	2723.0215
Homo sapiens sperm	GO:0005737|GO:000	NM_145263	Hs.527090	132671	5.587649
Homo sapiens mucin	GO:0005515|GO:000	NM_018406	Hs.369646	4585	6.504586
Homo sapiens chromosome 14 open readir		NR_024182	Hs.143845	256369	23.57911
Homo sapiens IQ mo	GO:0051056|GO:000	NM_003870	Hs.430551	8826	1342.8832
Homo sapiens zinc fi	GO:0005622|GO:000	NM_001001411	Hs.55452	163223	39.3169
Homo sapiens metastasis associated lung a		NR_002819	Hs.621695	378938	1108.8688
Homo sapiens structu	GO:0005515|GO:005	NM_015295	Hs.8118	23347	179.46199
Homo sapiens REX1	GO:0005622|GO:000	NM_172239	Hs.373854	254958	3948.3362
Homo sapiens liver e	GO:0005576|GO:004	NM_052971	Hs.337588	116842	53.43451
Homo sapiens microt	GO:0008017|GO:003	NM_005909	Hs.335079	4131	117.791504
Homo sapiens podop	GO:0006693|GO:000	NM_198389	Hs.468675	10630	4.9999995
					13.663879
Homo sapiens zinc fi	GO:0005622|GO:000	NM_001164457	Hs.438536	100131980	20.020231
Sphingosine-1-phosp	GO:0016787|GO:0016020|GO:0005783|G		Hs.591604	130367	4.9999995
Putative uncharacterized protein pp12708		[Source:UniProtKB/T	Hs.684470		16.889023
Homo sapiens BAT2	GO:0008022	NM_015172	Hs.494614	23215	913.78656
Homo sapiens nibrin	GO:0007095|GO:000	NM_002485	Hs.492208	4683	451.04132
					4.9999995
Solute carrier family 2, facilitated glucose transporter member 1 (Glucose transporter type 1, erythrocyte/bra					12.163371
Homo sapiens mucin	GO:0005515|GO:007	NM_002457	Hs.315	4583	15.638089
Protein FAM117B (Amyotrophic lateral sclerosis 2 chromosomal region candidate gene 13 protein) [Source					4.9999995
Homo sapiens zinc fi	GO:0005622|GO:000	NM_001105549	Hs.467210	55769	85.11065
Homo sapiens T-cell	GO:0005515	NM_199206	Hs.632346	9623	28.198944
Homo sapiens cell di	GO:0005515|GO:000	NM_024529	Hs.378996	79577	65.46344
Homo sapiens ring fi	GO:0005515|GO:000	NM_015565	Hs.288773	26046	68.47548
Homo sapiens zinc fi	GO:0005622|GO:000	NM_032679	Hs.148322	84765	34.408405
Homo sapiens amyotrophic lateral sclerosi		NM_152525	Hs.335788	151254	5.726276
					4.9999995
Homo sapiens golgi autoantigen, golgin su		NM_001012423	Hs.454647	390535	234.92496
Homo sapiens twinfil	GO:0005622|GO:000	NM_002822	Hs.189075	5756	3712.7896
Homo sapiens meiosi	GO:0005882|GO:000	NM_018365	Hs.444483	55329	11.505018
Uncharacterized protein C5orf42	[Source:UniProtKB/Swiss-Pro		Hs.586199		4.9999995
Homo sapiens platele	GO:0005515|GO:000	NM_000442	Hs.514412	5175	4.9999995
Homo sapiens chromosome 10 open readir		NM_014472	Hs.419800	27291	60.94942
Homo sapiens progra	GO:0042470|GO:000	NM_013374	Hs.475896	10015	208.7893
Homo sapiens clock l	GO:0005515|GO:000	NM_004898	Hs.436975	9575	162.02087
Homo sapiens centro	GO:0005515|GO:000	NM_024548	Hs.444135	79598	39.188354
PREDICTED: Homo sapiens hypothetical		XM_001719693	Hs.711228	100128559	4.9999995
PREDICTED: Homo sapiens ankyrin repea		XR_079090	Hs.609146		12.161721
Homo sapiens nephro	GO:0006629|GO:000	NM_153240	Hs.511991	27031	85.42963
Homo sapiens WD repeat domain 52 (WD		NM_018338	Hs.584936	55779	5.838146
Homo sapiens cDNA FLJ16734 fis, clone BRACE2002589. [AK			Hs.537458	641928	8.397914
Homo sapiens M-pha	GO:0008380|GO:000	NM_005791	Hs.656208	10199	711.54474
FSD1-like protein (FSD1 N-terminal-like protein)(Coiled-coil do			Hs.136901		25.452087
Homo sapiens solute	GO:0005739|GO:000	NM_001104647	Hs.144130	55186	417.71057
Homo sapiens neuron	GO:0005515|GO:004	NM_001037132	Hs.21422	4897	22.636692
Homo sapiens chrom	GO:0005739	NM_173826	Hs.55131	285343	464.9465
Homo sapiens ataxia	GO:0001741|GO:000	NM_001184	Hs.271791	545	462.55048
Homo sapiens testis expressed 13B (TEX1		NM_031273	Hs.333130	56156	13.897621
Homo sapiens zinc fi	GO:0005622|GO:000	NM_001012756	Hs.18103	339324	11.299142
Homo sapiens chromosome 6 open reading		NM_001010868	Hs.646309	206412	40.844254
PREDICTED: Homo sapiens upstream bin		XM_001714341	Hs.711293		4.9999995
Homo sapiens riboso	GO:0000287|GO:000	NM_004586	Hs.445387	6197	777.2392
Homo sapiens centro	GO:0005813|GO:000	NM_001077204	Hs.370147	79848	165.8463
Homo sapiens INO80 complex subunit D (		NM_017759	Hs.445036	54891	232.27095
Homo sapiens phospl	GO:0045859|GO:001	NM_016341	Hs.655033	51196	163.85648
Homo sapiens zinc fi	GO:0005622|GO:000	NM_183238	Hs.720299	100289635	181.18059
Homo sapiens coiled-coil domain containi		NM_182568	Hs.721149	284047	55.445312
Homo sapiens tetratri	GO:0005488|GO:000	NM_001042601	Hs.43213	151613	42.34544

7311.486	11.4879875	12.802887
15.73048	2.673758	3.834433
22.621569	2.8825474	4.365593
62.920727	4.70026	5.8513966
4689.1733	10.505375	12.141592
165.85803	5.429741	7.241085
3254.5415	10.23304	11.590675
660.9895	7.629172	9.225925
15911.054	12.011444	13.94567
134.67497	5.863428	6.9456415
339.43622	7.0070505	8.2516
34.414967	2.3362613	4.9639664
35.18651	3.9115763	4.9949584
68.47401	4.4571905	5.9743814
21.065393	2.3362613	4.2587886
41.54433	4.214742	5.2428174
3646.2026	9.958817	11.768349
1099.5391	8.969152	9.97739
13.589361	2.3362613	3.6148882
34.326164	3.7421317	4.9607153
39.238487	4.1019764	5.1582146
14.9504795	2.3362613	3.7626944
300.21747	6.5481806	8.074095
84.44028	4.958228	6.270637
157.0361	6.161196	7.161738
340.94968	6.2257237	8.259462
103.346085	5.2386513	6.5540023
18.786589	2.710198	4.0962563
18.108307	2.3362613	4.049256
653.04297	8.030844	9.208572
8282.629	11.921665	12.98572
72.50471	3.6682	6.0564857
33.79322	2.3362613	4.936234
11.886489	2.3362613	3.413381
173.1807	6.0527134	7.30317
585.14246	7.86334	9.044939
625.57526	7.474678	9.144891
112.322815	5.4246044	6.6749935
12.5506	2.3362613	3.4960961
40.224426	3.7419436	5.1977644
250.4834	6.5540023	7.816903
37.849888	2.7365198	5.106223
22.9035	3.2328374	4.3843055
2748.0898	9.620828	11.332455
97.915596	4.8059635	6.474412
1209.3723	8.853751	10.119804
62.53762	4.6345663	5.843318
1309.383	9.009414	10.237512
2116.491	9.003601	10.948457
35.87768	3.9341602	5.0254264
42.715816	3.6424634	5.282295
131.8424	5.480132	6.915095
15.4497595	2.3362613	3.8084054
2444.5964	9.745665	11.156582
628.308	7.510787	9.150461
790.85626	8.016511	9.4849
671.9384	7.490966	9.256714
605.0696	7.6457653	9.093037
232.73279	5.915263	7.7178874
142.04147	5.5295405	7.021583

A_23_P426153	2.0257446	down	NM_182628	chr3:126155222-126	CCDC37
A_33_P3284252	2.9567495	down		chrY:14491577-14491518	
A_24_P940125	2.4002589	down	NM_015455	chr5:180004898-180	CNOT6
A_33_P3405285	3.317699	down	NM_001164436	chr6:079852179-079	TMEM212
A_24_P127691	2.928611	down		chr1:225311254-225311313	
A_33_P3392245	2.2234366	down	NM_001136123	chr10:102719240-10	FAM178A
A_33_P3213695	2.8740537	down	NM_001079670	chr13:050008282-05	CAB39L
A_33_P3242493	2.5509928	down	NM_006346	chr13:073357799-07	PIBF1
A_23_P69683	2.5008994	down	NM_006323	chr4:110460882-110	SEC24B
A_33_P3257297	3.8336483	down	NM_001008723	chr10:106214776-10	CCDC147
A_24_P68311	3.271894	down	NM_015180		SYNE2
A_23_P126241	2.4198825	down	NM_003760	chr1:021133992-021	EIF4G3
A_24_P387869	2.797492	down	NM_006256	chr1:089300815-089	PKN2
A_24_P269814	2.0922003	down	NM_001001974	chr10:124191798-12	PLEKHA1
A_32_P154342	2.0653682	down	NM_180991	chr5:101570034-101	SLCO4C1
A_23_P337424	2.4958223	down	NM_001018067	chr1:067874360-067	SERBP1
A_33_P3316052	2.6867932	down		chrX:015782975-015783034	
A_32_P205944	2.7623599	down	NM_005054	chr2:111273172-111	RGPD5
A_33_P3215788	2.5187833	down	NM_001164315	chr2:098127700-098	ANKRD36
A_23_P153183	4.6649399	down	AB020709	chrX:21659844-21659903	
A_33_P3407235	3.5436669	down	XR_037576	chrX:073351716-073	LOC100133180
A_23_P30582	5.2475816	down	NM_004174	chr5:000480011-000	SLC9A3
A_33_P3412353	2.8413489	down	NM_152943	chr12:133781677-13	ZNF268
A_32_P42780	2.9422736	down	NR_024565	chr18:032890387-03	ZNF271
A_33_P3348194	2.4737803	down		chr6:82897078-82897019	
A_33_P3311637	2.4419133	down	NM_016106	chr14:031191705-03	SCFD1
A_24_P206736	2.1896632	down	NM_003442	chr11:009549384-00	ZNF143
A_33_P3285430	3.7494714	down		chr12:32892958-32893017	
A_24_P316939	2.1454749	down	NM_001137550	chr2:238688558-238	LRRFIP1
A_33_P3809328	2.3508613	down	AF111846	chr3:152047851-152	PRO0471
A_33_P3292179	2.4075037	down	NM_080283	chr17:066971122-06	ABCA9
A_23_P22134	2.0811998	down	NM_001717	chr15:083925019-08	BNC1
A_23_P411947	2.4702083	down	NM_172000	chr1:182367833-182	TEDDM1
A_23_P309950	3.0869876	down	AL833126	chr10:102685825-102685884	
A_23_P54556	2.4493628	down	NM_014048	chr16:014360546-01	MKL2
A_23_P55515	2.2710752	down	NM_003799		RNMT
A_33_P3333975	2.2132747	down	NM_001024916	chr9:070182078-070	CBWD5
A_33_P3311493	3.5855941	down	NR_026836	chr12:072656394-07	LOC283392
A_33_P3390570	2.8485782	down	NM_005933	chr11:118348856-11	MLL
A_24_P323598	2.3964735	down	NM_001017420	chr8:027662101-027	ESCO2
A_23_P5342	3.6504188	down	NM_018557	chr2:140989434-140	LRP1B
A_24_P49298	2.0042984	down	AB023191	chr10:75003877-750	FAM149B1
A_23_P411723	2.1969693	down	NM_002655	chr8:057074013-057	PLAG1
A_32_P208403	6.516879	down	NM_053064	chr14:052436151-05	GNG2
A_33_P3651282	2.3768535	down	DW416857	chr6:160201278-160	SNORA20
A_23_P5703	5.2351878	down	NM_175735		LYG2
A_24_P367421	2.6263569	down	XM_001718703	chr15:032792025-03	LOC100132816
A_32_P96807	2.4330956	down	NM_172071	chr1:173900682-173	RC3H1
A_24_P397386	3.2204947	down	NM_002310	chr5:038476001-038	LIFR
A_33_P3364443	2.950341	down	XM_373688	chr16:054958929-05	LOC388279
A_23_P43175	2.0713825	down	NM_144710	chr2:110300744-110	SEPT10
A_33_P3375934	2.2475481	down	NM_005746	chr7:105888852-105	NAMPT
A_33_P3345643	2.2321971	down	XM_001716434	chr5:000711383-000	ZDHHC11B
A_32_P109604	3.117364	down	XM_001715342	chr21:009769614-00	LOC100132733
A_33_P3385461	2.7324801	down	NM_001018055	chrX:154348363-154	BRCC3
A_33_P3221788	2.2493872	down	NM_020673	chr20:056942283-05	RAB22A
A_33_P3314659	3.111994	down	NM_144722	chr5:035671592-035	SPEF2
A_33_P3325558	2.2908786	down	XM_001723874	chr1:174514552-174514611	
A_33_P3231277	2.3768848	down	NM_181054	chr14:062211472-06	HIF1A
A_23_P97632	2.8133051	down	NM_004446	chr1:220142463-220	EPRS

Homo sapiens coiled-coil domain containi		NM_182628	Hs.591305	348807	7.200738
Putative uncharacterized protein GYG2P [Source:UniProtKB/TrEMBL;Acc:A6NHG5] [ENST000003829					4.9999995
Homo sapiens CCR4	GO:0005515|GO:00(	NM_015455	Hs.654984	57472	485.81622
Homo sapiens transm	GO:0016020|GO:001	NM_001164436	Hs.256034	389177	2649.669
Dynein heavy chain 14, axonemal (Axonemal beta dynein heavy chain 14)(Ciliary dynein heavy chain 14) [					8.539371
Homo sapiens family with sequence simila		NM_001136123	Hs.447458	55719	21.737991
Homo sapiens calciu	GO:0005515|GO:004	NM_001079670	Hs.87159	81617	14.434412
Homo sapiens progesterone immunomodul		NM_006346	Hs.441926	10464	56.149773
Homo sapiens SEC24	GO:0005515|GO:00(	NM_006323	Hs.292472	10427	303.47778
Homo sapiens coiled-coil domain containi		NM_001008723	Hs.253576	159686	6.137914
Homo sapiens spectri	GO:0031965|GO:00(	NM_015180	Hs.525392	23224	17.325062
Homo sapiens eukary	GO:0005515|GO:00(	NM_003760	Hs.467084	8672	249.27405
Homo sapiens protei	GO:0005622|GO:00(	NM_006256	Hs.440833	5586	194.55432
Homo sapiens plecks	GO:0005886|GO:004	NM_001001974	Hs.643512	59338	211.47723
Homo sapiens solute	GO:0005215|GO:00(	NM_180991	Hs.127648	353189	22.306614
Homo sapiens SERPI	GO:0005515|GO:004	NM_001018067	Hs.719078	26135	66.8748
					6.275578
Homo sapiens RANE	GO:0005488|GO:004	NM_005054	Hs.469630	84220	216.79019
Homo sapiens ankyrin repeat domain 36 (A		NM_001164315	Hs.532921	375248	102.23031
Connector enhancer of kinase suppressor of ras 2 (Connector enl			Hs.555917		7.9171534
PREDICTED: Homo sapiens similar to DI		XR_037576	Hs.709917	100133180	36.099842
Homo sapiens solute	GO:0005515|GO:001	NM_004174	Hs.658120	6550	4.9999995
Homo sapiens zinc fi	GO:0005622|GO:00(	NM_152943	Hs.124047	10795	578.34094
Homo sapiens zinc finger protein 271 (ZN		NR_024565		10778	44.74281
Inhibitor of Bruton tyrosine kinase (IBtk) [Source:UniProtKB/Swiss-Prot;Acc:Q9P2D0] [ENST000003697					11.173882
Homo sapiens sec1 fa	GO:0005801|GO:00(	NM_016106	Hs.369168	23256	1099.1073
Homo sapiens zinc fi	GO:0005622|GO:00(	NM_003442	Hs.523471	7702	432.79636
Dynamin-1-like protein (EC 3.6.5.5)(Dynamin-like protein)(Dnm1p/Vps1p-like protein)(DVLP)(Dynamin 1					4.9999995
Homo sapiens leucin	GO:0016564|GO:00(	NM_001137550	Hs.471779	9208	195.45627
Homo sapiens PRO0471 mRNA, complete cds [AF111846]			Hs.621389	28994	18.67474
Homo sapiens ATP-b	GO:0016020|GO:00(	NM_080283	Hs.131686	10350	11634.885
Homo sapiens basonu	GO:0005622|GO:00(	NM_001717	Hs.459153	646	4.9999995
Homo sapiens transm	GO:0016020|GO:001	NM_172000	Hs.156977	127670	4.9999995
Protein FAM178A [Source:UniProtKB/Swiss-Prot;Acc:Q8IX21			Hs.447458		7.471847
Homo sapiens MKL/	GO:0003713|GO:004	NM_014048	Hs.49143	57496	420.31866
Homo sapiens RNA (	GO:0003723|GO:00(	NM_003799	Hs.592347	8731	75.26448
Homo sapiens COBW	GO:0000166|GO:00(	NM_001024916	Hs.645337	220869	1449.7466
Homo sapiens hypothetical LOC283392 (L		NR_026836		283392	4.9999995
Homo sapiens myelo	GO:0006355|GO:00(	NM_005933	Hs.258855	4297	1992.7994
Homo sapiens establi	GO:0003684|GO:00(	NM_001017420	Hs.99480	157570	663.2786
Homo sapiens low de	GO:0005515|GO:00(	NM_018557	Hs.656461	53353	4.9999995
Protein FAM149B1 [Source:UniProtKB/Swiss-Prot;Acc:Q96BI			Hs.408577	317662	4.9999995
Homo sapiens pleion	GO:0005622|GO:00(	NM_002655	Hs.14968	5324	77.027084
Homo sapiens guanin	GO:0007165|GO:00(	NM_053064	Hs.708109	54331	4.9999995
HHAGE017587 Human liver regeneration after partial hepatecto			Hs.689705	677806	15.333421
Homo sapiens lysozy	GO:0009253|GO:001	NM_175735	Hs.436468	254773	9.446556
PREDICTED: Homo sapiens similar to Pu		XM_001718703	Hs.711751	100132816	364.6015
Homo sapiens ring fi	GO:0005515|GO:00(	NM_172071	Hs.30258	149041	266.50586
Homo sapiens leuken	GO:0008284|GO:00(	NM_002310	Hs.133421	3977	63.17627
PREDICTED: Homo sapiens hypothetical		XM_373688	Hs.681782	388279	17.092348
Homo sapiens septin	GO:0031105|GO:00(	NM_144710	Hs.469615	151011	10749.565
Homo sapiens nicotin	GO:0007165|GO:004	NM_005746	Hs.489615	10135	1175.3389
PREDICTED: Homo	GO:0016020|GO:00(	XM_001716434		653082	39.873978
PREDICTED: Homo sapiens similar to FL		XM_001715342	Hs.709909	100132733	18.163593
Homo sapiens BRCA	GO:0005515|GO:00(	NM_001018055	Hs.558537	79184	118.9082
Homo sapiens RAB2	GO:0005515|GO:00(	NM_020673	Hs.529044	57403	320.6899
Homo sapiens sperm	GO:0046983	NM_144722	Hs.298863	79925	23.2382
RAB GTPase-activating protein 1-like [Sc		XM_001723874	Hs.585378		237.3713
Homo sapiens hypox	GO:0030949|GO:00(	NM_181054	Hs.597216	3091	438.62637
Homo sapiens glutan	GO:0005515|GO:00(	NM_004446	Hs.497788	2058	2325.1382

17.99908	3.021274	4.0397263
16.474861	2.3362613	3.9002733
1396.9197	9.072004	10.335194
9406.467	11.450146	13.180329
30.684574	3.2541084	4.804325
57.652103	4.577399	5.7301903
49.619125	3.986079	5.509166
171.37943	5.935837	7.2868958
933.8656	8.401361	9.723808
29.502188	2.80395	4.742668
67.86961	4.2505054	5.9606314
740.861	8.118959	9.393896
663.6396	7.749646	9.23378
547.7776	7.881305	8.946326
55.201878	4.614999	5.661398
200.7664	6.1924334	7.5119486
21.09384	2.834515	4.2604003
734.9443	7.9152727	9.381174
314.94162	6.808861	8.141588
45.5906	3.156774	5.3786325
153.80142	5.3091726	7.1344156
29.20422	2.3362613	4.727914
1936.9346	9.312914	10.81949
157.32837	5.6068916	7.163823
33.83456	3.6306329	4.9373503
3070.3787	10.220299	11.508311
1142.8676	8.904184	10.034893
20.806894	2.3362613	4.2429485
513.15375	7.756281	8.857578
52.464294	4.354953	5.5881424
27559.504	13.515106	14.782644
11.750349	2.3362613	3.3936768
13.792628	2.3362613	3.640894
28.696959	3.0760279	4.7022276
1236.525	8.8610935	10.1535
206.4416	6.3691025	7.552478
3620.6042	10.611445	11.7576275
19.886688	2.3362613	4.1784735
6189.5146	11.05176	12.562002
1881.8978	9.517166	10.778079
20.254503	2.3362613	4.2043233
11.340019	2.3362613	3.3393586
204.50246	6.40163	7.5371447
36.246433	2.3362613	5.0404425
43.843826	4.068589	5.317642
60.098198	3.3993287	5.78757
1158.1761	8.663521	10.056584
795.0538	8.211521	9.494314
245.59549	6.104152	7.7914343
60.389584	4.231975	5.7928567
22148.514	13.403244	14.453838
3013.6274	10.314306	11.482658
107.1509	5.448329	6.6067934
67.60811	4.314804	5.9551306
395.65247	7.022312	8.472523
887.0446	8.480814	9.650346
87.357735	4.6781607	6.316
666.73474	8.044644	9.240545
1256.7048	8.926744	10.175816
7150.14	11.276066	12.768332

A_33_P3849600	2.9230692	down	M30627	chr11:27911711-279	HSP90AA2
A_24_P137522	2.6700182	down	NM_019050	chr4:120215256-120	USP53
A_23_P201731	2.240533	down	NM_004619	chr1:211548188-211	TRAF5
A_33_P3420352	3.4411522	down	XM_001714648	chr6:027356142-027	LOC100128090
A_23_P5611	4.8474005	down	NM_018151	chr2:152331786-152	RIF1
A_23_P347777	2.8886754	down	NM_001040274		SYCP2L
A_24_P845072	2.0435506	down	NM_144963	chr1:149650197-149	FAM91A1
A_23_P99260	2.5877639	down	NM_006838	chr12:095908032-09	METAP2
A_23_P146325	5.0551132	down	NR_002765	chr8:131307736-131	DDEF1IT1
A_33_P3272395	4.2886156	down	NM_001008749	chr7:140107587-140	RAB19
A_33_P3296871	3.6168318	down	NM_006955	chr10:043084664-04	ZNF33B
A_24_P192627	2.163771	down	NM_004529	chr9:020345250-020	MLLT3
A_23_P127676	2.4086153	down	NM_014633	chr11:010800906-01	CTR9
A_23_P145424	3.5724764	down	NM_014895	chr6:084834053-084	KIAA1009
A_24_P3140	4.786713	down	NM_014497	chr2:071654285-071	ZNF638
A_33_P3732854	2.1202722	down	AY358215	chr1:247946900-247	OR9H1P
A_33_P3567961	7.8894028	down	AK023417	chr5:86534703-8653	FLJ11292
A_24_P134653	2.5167327	down	NM_003611		OFD1
A_24_P374759	2.5435993	down	NM_015576	chr3:056026254-056	ERC2
A_23_P51317	2.0680341	down	NM_019083	chr1:100615850-100	CCDC76
A_33_P3888629	2.2055936	down	NM_001164000	chr3:168801415-168	MECOM
A_33_P3336449	2.2175527	down		chr2:026894184-026894125	
A_23_P433369	2.5322415	down	NM_015473	chr14:031761197-03	HEATR5A
A_24_P354715	2.0807534	down	NM_002526	chr6:086204891-086	NT5E
A_33_P3416448	2.1963768	down	NM_001002860	chr14:093709105-09	BTBD7
A_33_P3219398	2.282178	down	NM_015461	chr18:022804452-02	ZNF521
A_24_P941922	3.7853629	down	BC035590	chr7:135071445-135071386	
A_33_P3242369	4.5288373	down	AF370399	chr1:243788824-243788765	
A_23_P206228	4.0448318	down	NM_020821	chr15:062144891-06	VPS13C
A_33_P3293529	3.271488	down	XM_001715665	chr8:73342072-7334	LOC100129527
A_32_P14721	3.8059283	down	NM_178504	chr3:057327856-057	DNAH12
A_33_P3230166	2.8456847	down	NM_052867	chr13:101706223-10	NALCN
A_32_P199252	3.3753193	down	NM_001017963	chr11:027911338-02	HSP90AA1
A_24_P710730	2.2811384	down	NR_024054	chr5:069438247-069	LOC100170939
A_33_P3347343	2.3546493	down	NM_001093729	chr18:066564598-06	CCDC102B
A_24_P136402	3.9130803	down	NM_005933		MLL
A_33_P3407479	6.5570173	down	XR_078491	chr15:079276828-07	LOC100129540
A_23_P151267	2.0219425	down	NM_016357	chr12:050569850-05	LIMA1
A_33_P3269824	2.8195027	down	NM_001024611	chr4:052859950-052	LRRC66
A_32_P178800	2.9524002	down	NM_002203	chr5:052389637-052	ITGA2
A_24_P97342	3.3479215	down	NM_021935	chr3:071820961-071	PROK2
A_33_P3305438	2.0204034	down		chr7:155684885-155684944	
A_33_P3289441	2.1667778	down	AK125976	chr2:130561141-130	LOC100131048
A_33_P3610138	2.3550249	down	BC043554	chr17:4520873-4520	LOC400568
A_24_P123119	2.0688502	down	NM_001966	chr3:184909483-184	EHHADH
A_23_P371011	2.3878194	down	NM_182490	chr19:044740960-04	ZNF227
A_23_P53646	2.1183486	down	NM_139207	chr12:076440082-07	NAP1L1
A_24_P865	2.0782781	down	NM_206921	chr6:118887116-118	C6orf204
A_24_P157370	2.4394885	down	NM_018725	chr3:053899592-053	IL17RB
A_33_P3365077	2.597933	down	NM_001001915	chr1:247752556-247	OR2G2
A_32_P126609	3.3311227	down	NM_015110	chr9:072968897-072	SMC5
A_33_P3352098	2.0112512	down	NM_021201	chr11:060163279-06	MS4A7
A_33_P3264419	2.4254358	down	NM_152758	chr8:064125285-064	YTHDF3
A_24_P931736	11.1285421	down	AY358216	chr5:169372909-169	UNQ9374
A_23_P14559	3.3609107	down	NM_006871	chr14:024806583-02	RIPK3
A_23_P200001	3.6517399	down	NM_144573	chr1:078408667-078	NEXN
A_23_P318420	2.1111661	down	NM_018704	chr1:113002131-113	CTTNBP2NL
A_33_P3376644	2.6034255	down	NM_015571	chr6:076426668-076	SENP6
A_23_P38408	2.3985092	down	NM_024835	chr17:034945927-03	GGNBP2
A_32_P109036	2.8521815	down	NM_001076678	chr19:021609167-02	ZNF493

Human heat shock protein 86 mRNA, 5'end [M30627]			Hs.719139	3324	11968.289
Homo sapiens ubiqui	GO:0008150|GO:000	NM_019050	Hs.431081	54532	37.541588
Homo sapiens TNF r	GO:0005515|GO:000	NM_004619	Hs.523930	7188	304.53766
PREDICTED: Homo sapiens hypothetical		XM_001714648	Hs.680366	100128090	4.9999995
Homo sapiens RAP1	GO:0007049|GO:000	NM_018151	Hs.655671	55183	199.21687
Homo sapiens synapt	GO:0005634	NM_001040274	Hs.720408	221711	9.15937
Homo sapiens family with sequence simila		NM_144963	Hs.459174	157769	426.59436
Homo sapiens methio	GO:0005515|GO:000	NM_006838	Hs.444986	10988	7233.009
Homo sapiens DDEF1 intronic transcript 1		NR_002765	Hs.639318	29065	107.8262
Homo sapiens RAB1	GO:0005886|GO:000	NM_001008749	Hs.583545	401409	4.9999995
Homo sapiens zinc fi	GO:0005622|GO:000	NM_006955	Hs.499453	7582	150.01009
Homo sapiens myelo	GO:0005515|GO:000	NM_004529	Hs.591085	4300	11.30331
Homo sapiens Ctr9, I	GO:0005515|GO:003	NM_014633	Hs.719124	9646	1493.5314
Homo sapiens KIAA	GO:0005515|GO:000	NM_014895	Hs.485865	22832	11.650699
Homo sapiens zinc fi	GO:0008380|GO:000	NM_014497	Hs.434401	27332	108.72531
Homo sapiens clone DNA175742 VNFT9373 (UNQ9373) mRN			Hs.446996	81439	4.9999995
Homo sapiens cDNA FLJ13355 fis, clone PLACE1000048. [AK			Hs.719629	55338	4.9999995
Homo sapiens oral-fa	GO:0008150|GO:000	NM_003611	Hs.6483	8481	232.54857
Homo sapiens ELKS	GO:0005515|GO:004	NM_015576	Hs.476389	26059	12.00791
Homo sapiens coiled	GO:0008168|GO:000	NM_019083	Hs.440371	54482	188.07388
Homo sapiens MDS1	GO:0005622|GO:000	NM_001164000	Hs.721616	2122	295.43903
					16.12809
Homo sapiens HEAT	GO:0005488	NM_015473	Hs.370299	25938	91.76028
Homo sapiens 5'-nuc	GO:0005886|GO:001	NM_002526	Hs.153952	4907	6081.6562
Homo sapiens BTB (	GO:0005515	NM_001002860	Hs.525549	55727	19.347471
Homo sapiens zinc finger protein 521 (ZN		NM_015461	Hs.116935	25925	8.036764
CCR4-NOT transcription complex subunit 4 (EC 6.3.2.-)(E3 ubi			Hs.490224		24.972374
Homo sapiens PP10881 mRNA, complete cds. [AF370399]			Hs.689808		4.9999995
Homo sapiens vacuol	GO:0008104	NM_020821	Hs.511668	54832	123.692314
Putative uncharacterized protein ENSP000		XM_001715665		100129527	294.06012
Homo sapiens dyneir	GO:0005929|GO:003	NM_178504	Hs.201378	201625	4.9999995
Homo sapiens sodiur	GO:0005244|GO:001	NM_052867	Hs.525146	259232	14.211499
Homo sapiens heat sl	GO:0006986|GO:004	NM_001017963	Hs.525600	3320	5204.18
Homo sapiens glucuronidase, beta pseudog		NR_024054	Hs.582500	100170939	675.67096
Homo sapiens coiled-coil domain containi		NM_001093729	Hs.280781	79839	4.9999995
Homo sapiens myelo	GO:0006355|GO:000	NM_005933	Hs.258855	4297	5.929096
PREDICTED: Homo sapiens hypothetical		XR_078491	Hs.628768	100129540	5.086332
Homo sapiens LIM d	GO:0003785|GO:003	NM_016357	Hs.525419	51474	1560.8912
Homo sapiens leucin	GO:0005515|GO:001	NM_001024611	Hs.661450	339977	4.9999995
Homo sapiens integri	GO:0014070|GO:000	NM_002203	Hs.482077	3673	93.94614
Homo sapiens prokin	GO:0001664|GO:000	NM_021935	Hs.528665	60675	4.9999995
					113.58583
Homo sapiens cDNA FLJ43988 fis, clone TESTI4019417. [AK			Hs.640186	100131048	12.64358
Homo sapiens cDNA clone IMAGE:5176687 [BC043554]			Hs.434312	400568	4.9999995
Homo sapiens enoyl-	GO:0006629|GO:005	NM_001966	Hs.429879	1962	9.948585
Homo sapiens zinc fi	GO:0005622|GO:000	NM_182490	Hs.371335	7770	73.63356
Homo sapiens nucleo	GO:0006260|GO:000	NM_139207	Hs.524599	4673	8023.036
Homo sapiens chromosome 6 open reading		NM_206921	Hs.656959	387119	47.298817
Homo sapiens interle	GO:0004896|GO:000	NM_018725	Hs.654970	55540	11.15915
Homo sapiens olfacto	GO:0007608|GO:000	NM_001001915	Hs.690208	81470	5.600748
Homo sapiens structu	GO:0006281|GO:000	NM_015110	Hs.534189	23137	84.44988
Homo sapiens memb	GO:0007165|GO:001	NM_021201	Hs.530735	58475	55.14079
Homo sapiens YTH domain family, memb		NM_152758	Hs.491861	253943	1308.9927
Homo sapiens clone DNA175753 VCEW9374 (UNQ9374) mRI			Hs.626043	100133106	4.9999995
Homo sapiens recept	GO:0005515|GO:000	NM_006871	Hs.268551	11035	4.9999995
Homo sapiens nexilir	GO:0030334|GO:000	NM_144573	Hs.612385	91624	205.50806
Homo sapiens CTTN	GO:0015629	NM_018704	Hs.485899	55917	109.60163
Homo sapiens SUM(	GO:0019941|GO:000	NM_015571	Hs.485784	26054	43.68675
Homo sapiens gamet	GO:0016023|GO:000	NM_024835	Hs.514116	79893	237.6334
Homo sapiens zinc fi	GO:0005622|GO:000	NM_001076678	Hs.656558	284443	37.49713

35054.29	13.559203	15.106687
120.265976	5.362659	6.7795086
838.7589	8.406728	9.57057
19.079773	2.3362613	4.119153
1163.8016	7.7865477	10.063759
32.63434	3.3568249	4.887233
1057.605	8.88245	9.913528
19205.893	12.85532	14.227026
657.5028	6.8807626	9.218506
23.77541	2.3362613	4.4367733
655.6482	7.3599825	9.214709
29.793673	3.6432247	4.7567725
4057.9546	10.654894	11.923098
50.196228	3.6868925	5.523817
629.4254	6.8958797	9.154915
11.94208	2.3362613	3.4205108
43.77839	2.3362613	5.3161774
718.08765	8.017833	9.349385
37.083195	3.7266102	5.0734816
478.18353	7.69936	8.74762
802.07666	8.362943	9.50411
43.224483	4.147394	5.2963624
284.61865	6.6556034	7.9960184
13028.652	12.601036	13.658142
50.841995	4.4079638	5.5430894
22.644558	3.1763852	4.3667965
113.9226	4.778595	6.6990266
25.058022	2.3362613	4.515402
607.90564	7.0838203	9.0999
1165.4221	8.357141	10.067088
21.137474	2.3362613	4.2645097
48.499577	3.9601932	5.468969
18079.69	12.391214	14.146238
1822.2809	9.539156	10.72891
13.19537	2.3362613	3.5717735
29.127882	2.7565088	4.7248135
41.89255	2.541969	5.2550087
3542.2097	10.710367	11.726109
15.697427	2.3362613	3.831702
339.005	6.6877337	8.249622
18.553038	2.3362613	4.079527
279.90042	6.957196	7.9718394
33.349983	3.8016858	4.917237
13.197439	2.3362613	3.5720036
25.131533	3.4696088	4.518438
212.05586	6.334603	7.5902967
17287.357	13.00204	14.08498
117.305466	5.686619	6.7420077
33.281624	3.6280007	4.9145794
18.17376	2.6781864	4.0555506
344.24316	6.5358205	8.271829
131.8942	5.907415	6.9155083
3595.7512	10.4692955	11.7475395
61.240726	2.3362613	5.812454
18.621038	2.3362613	4.0851135
924.0314	7.837434	9.706018
282.03613	6.9042034	7.9822435
135.29822	5.5715466	6.9519577
697.2185	8.046173	9.308311
128.08688	5.3607655	6.8728313

A_23_P358394	4.8018768	down	NM_015864	chr6:024843154-024	FAM65B
A_32_P126375	3.7227207	down	NM_198270	chrX:017753480-017	NHS
A_33_P3393650	2.3149812	down	NM_019012	chr12:019436601-01	PLEKHA5
A_33_P3282434	2.0417988	down	NM_001166496	chr1:113454562-113	SLC16A1
A_23_P319808	2.563088	down	NM_015054		UHRF1BP1L
A_33_P3323258	2.6839102	down		chr6:5847420-5847479	
A_33_P3250939	5.9698567	down	NM_138453	chr5:058147307-058	RAB3C
A_23_P44768	2.3238163	down	NM_013254	chr12:064895443-06	TBK1
A_23_P200936	2.1269233	down	NM_000254	chr1:237063523-237	MTR
A_24_P128145	2.0862478	down	NM_001880	chr2:175979528-175	ATF2
A_33_P3297655	4.5181078	down	XM_001721108	chr9:091625237-091	LOC100131540
A_33_P3470781	9.2673361	down	NM_020343	chr20:020475821-02	RALGAPA2
A_33_P3307457	2.5512817	down	NM_031963	chr17:039383845-03	KRTAP9-8
A_23_P403335	3.0579059	down	NM_015065	chr11:108379859-10	EXPH5
A_33_P3373654	2.1425062	down	XM_001715439	chr5:28927418-28927477	
A_33_P3503284	3.0210281	down	BC021740	chr18:54815440-548	BOD1P
A_33_P3210916	2.2165541	down		chr3:10354823-10354764	
A_24_P126892	2.4536849	down	AF116680	chr8:48282132-48282191	
A_33_P3691916	2.1228555	down	NM_014883	chr4:089647940-089	FAM13A
A_33_P3378514	2.4770895	down	NM_001083	chr4:120415615-120	PDE5A
A_33_P3286066	2.2305864	down	NM_001134438	chr3:111694456-111	PHLDB2
A_33_P3296240	5.2513331	down	NM_017738	chr9:017487004-017	CNTLN
A_33_P3269578	2.1349969	down	NM_018313	chr3:052582118-052	PBRM1
A_33_P3258561	3.5221906	down		chr7:107408063-107408004	
A_23_P161091	2.8437133	down	NM_024772	chr1:035581035-035	ZMYM1
A_23_P348159	9.1811865	down	NM_020388	chr6:056468910-056	DST
A_23_P408675	2.235228	down	NM_003972	chr10:093789192-09	BTAF1
A_23_P351168	2.8229399	down	NM_145174	chr22:041255827-04	DNAJB7
A_33_P3218760	2.3613616	down	NM_080599	chr10:012077296-01	UPF2
A_33_P3389603	2.4063096	down	AK131572	chr6:111630997-111	LOC100128477
A_23_P332584	3.5760297	down	NM_015237	chr1:092649667-092	KIAA1107
A_24_P415845	3.8366546	down	NM_002312	chr13:108860815-10	LIG4
A_24_P120109	2.0049742	down	NM_198963	chr2:039088500-039	DHX57
A_32_P139738	2.9328669	down	NR_002827	chr16:032163218-03	HERC2P4
A_24_P261052	2.3928461	down	NM_015458	chr8:011184845-011	MTMR9
A_33_P3348239	2.3395668	down	NM_000138	chr15:048700564-04	FBN1
A_32_P190951	2.2862469	down	XM_002347483	chr14:106967160-106967101	
A_23_P202170	2.6011958	down	NM_012215	chr10:103544394-10	MGEA5
A_33_P3220976	5.4051153	down	XM_001716704	chr3:019987470-019	LOC100130288
A_23_P303851	4.8959574	down	NM_176886		TAS2R45
A_23_P69941	2.4332158	down	NR_027706	chr5:111756497-111	FLJ11235
A_23_P212854	3.2538084	down	NM_002100		GYPB
A_23_P308839	7.6645214	down	NM_133448	chr12:129557409-12	TMEM132D
A_24_P135748	2.2682889	down	NM_024719		GRTP1
A_23_P200298	2.506935	down	NM_000028	chr1:100389229-100	AGL
A_24_P912366	16.3910276	down	NM_001109977	chr4:152498817-152	FAM160A1
A_23_P17275	2.057301	down	NM_016544	chr2:025180014-025	DNAJC27
A_23_P124570	2.8913345	down	NM_005246		FER
A_33_P3332724	2.2467396	down	NM_015120	chr2:073682362-073	ALMS1
A_23_P78018	2.9720636	down	NM_018672	chr17:067243589-06	ABCA5
A_33_P3326271	5.4220789	down	NM_001004481	chr9:107367131-107	OR13C2
A_33_P3366850	5.5944438	down	BC070343	chr14:22434092-22434151	
A_23_P144999	3.2239402	down	NM_016340	chr5:130762134-130	RAPGEF6
A_24_P307964	3.559914	down	NM_001012415	chr9:138585325-138	SOHLH1
A_23_P9603	2.2958796	down	NM_006904	chr8:048686360-048	PRKDC
A_24_P36944	3.1951917	down	NM_014812	chr1:243289868-243	CEP170
A_23_P30175	2.2436055	down	NM_018695	chr5:065375840-065	ERBB2IP
A_24_P86240	2.6459064	down	NM_198892	chr4:079833009-079	BMP2K
A_33_P3251283	2.5255425	down	NM_198495	chr6:132030195-132	CTAGE4
A_33_P3258117	2.1984519	down	NM_018063	chr10:096334433-09	HELLS

Homo sapiens family	GO:0005739|GO:000	NM_015864	Hs.559459	9750	4.9999995
Homo sapiens Nance	GO:0002088|GO:000	NM_198270	Hs.201623	4810	71.52187
Homo sapiens plecks	GO:0005515|GO:000	NM_019012	Hs.188614	54477	64.56428
Homo sapiens solute	GO:0015130|GO:000	NM_001166496	Hs.75231	6566	4.9999995
Homo sapiens UHRF1 binding protein 1-li		NM_015054	Hs.620701	23074	230.23546
Putative uncharacterized protein C6orf202 [Source:UniProtKB/Swiss-Prot;Acc:Q5TBU7] [ENST0000037					4.9999995
Homo sapiens RAB3	GO:0005515|GO:000	NM_138453	Hs.482173	115827	4.9999995
Homo sapiens TANK	GO:0005515|GO:000	NM_013254	Hs.505874	29110	562.12067
Homo sapiens 5-metl	GO:0005515|GO:003	NM_000254	Hs.498187	4548	877.8033
Homo sapiens activa	GO:0043565|GO:000	NM_001880	Hs.592510	1386	59.477333
PREDICTED: Homo sapiens hypothetical		XM_001721108	Hs.292737	100131540	4.9999995
Homo sapiens Ral G	GO:0005622|GO:005	NM_020343	Hs.472285	57186	4.9999995
Homo sapiens keratin	GO:0045095	NM_031963	Hs.307011	83901	4.9999995
Homo sapiens exoph	GO:0005515|GO:001	NM_015065	Hs.28540	23086	118.793495
LOC729862 protein [Source:UniProtKB/T		XM_001715439	Hs.646006		9.933096
Homo sapiens family with sequence similarity 44, member C, m			Hs.127882	284257	9.969341
Protein SEC13 homolog (SEC13-related protein)(SEC13-like protein 1) [Source:UniProtKB/Swiss-Prot;Ac					13.216379
Homo sapiens PRO2007 mRNA, complete cds. [AF116680]					4.9999995
Homo sapiens family with sequence simila		NM_014883	Hs.97270	10144	57.85624
Homo sapiens phospl	GO:0001666|GO:000	NM_001083	Hs.647971	8654	15.8921795
Homo sapiens plecks	GO:0005737|GO:001	NM_001134438	Hs.477114	90102	46.447823
Homo sapiens centle	GO:0007165|GO:001	NM_017738	Hs.435381	54875	4.9999995
Homo sapiens polybr	GO:0007507|GO:000	NM_018313	Hs.189920	55193	391.57034
					4.9999995
Homo sapiens zinc fi	GO:0046983|GO:000	NM_024772	Hs.471243	79830	188.0105
Homo sapiens dystor	GO:0005515|GO:003	NM_020388	Hs.604915	667	16.57079
Homo sapiens BTAF	GO:0003700|GO:001	NM_003972	Hs.500526	9044	278.32147
Homo sapiens DnaJ (	GO:0031072	NM_145174	Hs.585042	150353	6.1593328
Homo sapiens UPF2	GO:0048471|GO:000	NM_080599	Hs.370689	26019	654.0362
Homo sapiens cDNA FLJ16829 fis, clone UTERU3020583. [AK131572]				100128477	4.9999995
Homo sapiens KIAA1107 (KIAA1107), m		NM_015237	Hs.21554	23285	4.9999995
Homo sapiens ligase	GO:0000793|GO:000	NM_002312	Hs.166091	3981	21.269161
Homo sapiens DEAH	GO:0008026|GO:000	NM_198963	Hs.468226	90957	35.118587
Homo sapiens hect domain and RLD 2 pse		NR_002827	Hs.531536	440362	41.672615
Homo sapiens myotu	GO:0005515|GO:001	NM_015458	Hs.591395	66036	55.49815
Homo sapiens fibrilli	GO:0007507|GO:000	NM_000138	Hs.591133	2200	16.912897
Ig heavy chain V-I region HG3 Precursor		XM_002347483	Hs.704357		58.498646
Homo sapiens menin	GO:0060124|GO:004	NM_012215	Hs.500842	10724	3502.9575
PREDICTED: Homo sapiens hypothetical		XM_001716704	Hs.120196	100130288	5.554794
Homo sapiens taste r	GO:0007165|GO:005	NM_176886	Hs.287378	259291	10.9996605
Homo sapiens hypothetical FLJ11235 (FL		NR_027706	Hs.591264	54508	4.9999995
Homo sapiens glycop	GO:0005886|GO:000	NM_002100	Hs.654368	2994	4.9999995
Homo sapiens transm	GO:0008150|GO:000	NM_133448	Hs.507268	121256	4.9999995
Homo sapiens growt	GO:0005622|GO:003	NM_024719	Hs.170904	79774	4.9999995
Homo sapiens amylo	GO:0005515|GO:004	NM_000028	Hs.904	178	136.03062
Homo sapiens family with sequence simila		NM_001109977	Hs.633810	729830	4.9999995
Homo sapiens DnaJ (	GO:0000166|GO:000	NM_016544	Hs.434993	51277	32.207943
Homo sapiens fer (fp	GO:0007242|GO:001	NM_005246	Hs.221472	2241	64.09242
Homo sapiens Alstro	GO:0042632|GO:000	NM_015120	Hs.184720	7840	28.899374
Homo sapiens ATP-b	GO:0005794|GO:001	NM_018672	Hs.421474	23461	143.03789
Homo sapiens olfact	GO:0007608|GO:000	NM_001004481	Hs.553789	392376	4.9999995
Putative uncharacterized protein ENSP00000374949 [Source:U			Hs.74647		4.9999995
Homo sapiens Rap g	GO:0005622|GO:000	NM_016340	Hs.483329	51735	260.59097
Homo sapiens sperm	GO:0005737|GO:004	NM_001012415	Hs.120464	402381	4.9999995
Homo sapiens protei	GO:0004677|GO:000	NM_006904	Hs.491682	5591	1421.9309
Homo sapiens centro	GO:0005737|GO:000	NM_014812	Hs.533635	9859	42.929665
Homo sapiens erbb2	GO:0005515|GO:003	NM_018695	Hs.591774	55914	3331.8582
Homo sapiens BMP2	GO:0030500|GO:001	NM_198892	Hs.146551	55589	148.63701
Homo sapiens CTAC	GO:0008150|GO:001	NM_198495	Hs.720693	100128553	60.768787
Homo sapiens helica	GO:0031508|GO:000	NM_018063	Hs.655830	3070	516.67114

26.605253	2.3362613	4.5998597
324.30136	6.2907076	8.187065
178.3442	6.1393423	7.3503428
11.53024	2.3362613	3.366102
723.4884	8.002642	9.360525
14.92955	2.3362613	3.7605977
33.26659	2.3362613	4.9139576
1553.137	9.272066	10.488562
2189.6826	9.912048	11.000816
148.23643	6.0169225	7.077833
24.989523	2.3362613	4.51198
51.044434	2.3362613	5.548416
14.240221	2.3362613	3.6874835
442.13742	7.021079	8.633623
26.00357	3.4675722	4.5668716
36.921783	3.4724994	5.067539
35.511673	3.8634307	5.0117493
13.73048	2.3362613	3.6312113
146.46497	5.976952	7.0629582
47.340153	4.124634	5.43328
123.75991	5.6639075	6.8213305
29.213951	2.3362613	4.728945
1023.6501	8.76605	9.860284
19.54301	2.3362613	4.1527343
651.30994	7.697975	9.205751
183.19499	4.1852994	7.38398
763.6339	8.277033	9.437455
21.73973	2.810271	4.3074694
1828.3899	9.493963	10.733582
13.483631	2.3362613	3.6030836
19.834064	2.3362613	4.17462
98.69549	4.5448074	6.4846563
84.57038	5.2686243	6.272208
145.82538	5.504998	7.0573096
158.86069	5.9162674	7.174995
47.601074	4.2168126	5.443054
160.09215	5.9928823	7.1858635
9642.263	11.835215	13.21439
37.72311	2.6656976	5.100023
64.98628	3.608691	5.900282
13.61888	2.3362613	3.6191256
17.98636	2.3362613	4.0383906
42.452827	2.3362613	5.274457
12.749978	2.3362613	3.5178657
417.10797	7.2230844	8.549009
90.92765	2.3362613	6.3710957
79.62146	5.1458235	6.1865764
223.42303	6.1283665	7.660102
78.30041	4.9949584	6.1627913
515.89746	7.29381	8.865275
30.109316	2.3362613	4.7751074
31.105759	2.3362613	4.820256
1029.97	8.180791	9.869616
19.74043	2.3362613	4.1681037
3675.054	10.583773	11.78282
164.44434	5.550578	7.2264805
8015.413	11.76535	12.931169
478.55444	7.345627	8.749389
183.45166	6.0489197	7.385513
1361.4486	9.156631	10.293119

A_24_P94402	3.4622008	down	NM_005378	chr2:016086767-016	MYCN
A_23_P94501	2.2479735	down	NM_000700	chr9:075785002-075	ANXA1
A_23_P53856	2.1337024	down	NM_014887	chr13:033091850-03	N4BP2L2
A_23_P204324	3.0526686	down	NM_012062	chr12:032896876-03	DNM1L
A_23_P23356	2.3904049	down	NM_016052	chr1:218504518-218	RRP15
A_24_P282343	2.0546083	down	NM_003159	chrX:018638000-01{	CDKL5
A_24_P81298	2.234249	down	NM_001123355	chr9:127908953-127	PPP6C
A_33_P3258346	2.2414208	down	NM_017523	chr17:006674143-00	XAF1
A_23_P47991	2.5284566	down	NM_015335	chr12:116396747-11	MED13L
A_33_P3312044	3.5065964	down		chr5:074633011-074632952	
A_33_P3312489	3.0136546	down		chr2:098908365-098908424	
A_33_P3792641	4.6204703	down	AK090913	chr2:155292578-155	LOC100144595
A_33_P3461416	2.1302947	down	NM_001083899	chr19:055525459-05	GP6
A_23_P71598	3.1668488	down	NM_003829	chr9:013106858-013	MPDZ
A_33_P3753757	2.0831058	down	AK095652	chr9:114796264-114	LOC158402
A_24_P368575	2.6839718	down	NM_003615	chr3:027414644-027	SLC4A7
A_32_P23125	2.0326087	down	NR_001558	chr20:022541440-02	C20orf56
A_33_P3344297	4.8457645	down		chr11:133050359-133050418	
A_32_P148824	3.3507894	down	NM_017847	chr1:186390276-186	C1orf27
A_33_P3233916	4.4090288	down	NM_017508	chr11:015991854-01	SOX6
A_33_P3375127	2.3364358	down	AK092544	chr8:12436925-1243	LOC100131581
A_24_P943193	2.6094975	down	NM_006036	chr2:044545965-044	PREPL
A_33_P3322730	2.3096771	down			
A_23_P39171	5.7387296	down	NM_033106	chr19:056696864-05	GALP
A_24_P252794	2.1067849	down	NM_003591	chr10:035299334-03	CUL2
A_33_P3298406	2.6399337	down	NM_001010895	chr9:098691085-098	C9orf102
A_33_P3300757	2.8607353	down	NM_015018	chr6:083877869-083	DOPEY1
A_33_P3254191	2.4253013	down	NM_020841	chr12:076748399-07	OSBPL8
A_33_P3221119	2.3273844	down	NM_032292	chr1:155619547-155	GON4L
A_33_P3335147	2.4973119	down	NM_001018116	chr9:103349793-103	MURC
A_33_P3326872	2.0905207	down		chr13:023411111-023411052	
A_23_P204980	2.5704544	down	NM_020121	chr13:096453984-09	UGGT2
A_33_P3273969	2.8505672	down	NM_014827	chr1:203821489-203	ZC3H11A
A_33_P3298216	2.3288597	down	NM_015011	chr13:109860282-10	MYO16
A_24_P92558	2.1950421	down	NM_001039891	chrX:046359498-04(	ZNF674
A_23_P373464	2.076661	down	NM_002285		AFF3
A_33_P3367692	4.0175576	down	NM_001014975	chr1:196646719-196	CFH
A_33_P3378680	2.9549868	down		chr5:142152355-142152414	
A_23_P356021	2.1416192	down	NM_001018113	chrX:014862096-01	FANCB
A_23_P321223	4.3211044	down	NM_002674	chr12:102590400-10	PMCH
A_23_P65278	3.8689487	down	NM_015678	chr13:036246683-03	NBEA
A_23_P47904	3.8780599	down	NM_033124	chr12:049312491-04	CCDC65
A_32_P116323	2.1818365	down	NM_015092	chr16:018870483-01	SMG1
A_23_P157726	2.2747398	down	NM_017925	chr9:019372372-019	DENND4C
A_33_P3286699	2.1331655	down		chr6:026327899-026327958	
A_23_P147397	3.147977	down	NM_001080463	chr11:103349837-10	DYNC2H1
A_33_P3236642	3.4381986	down	NM_198551	chr1:222801854-222	MIA3
A_33_P3220994	2.1318111	down	XM_002342513	chr6:026869979-026	LOC100133280
A_33_P3315915	2.5717418	down		chr15:091414507-091414448	
A_23_P94911	2.145238	down	NM_181845	chr19:044352724-04	ZNF283
A_23_P77459	2.1230774	down	NM_001018159	chr16:066836909-06	NAE1
A_33_P3392829	3.2971063	down	NM_194312	chr2:239040850-239	ESPNL
A_33_P3260430	3.332221	down	NM_005988	chr1:153028658-153	SPRR2A
A_24_P100996	2.710922	down	NM_213599	chr11:022304318-02	ANO5
A_33_P3300747	2.7882125	down	NM_144650	chr8:067359516-067	ADHFE1
A_23_P105436	2.0700061	down	NM_002834	chr4:083502136-083	PTPN11
A_33_P3353125	2.1634668	down		chr5:043586668-043586609	
A_23_P395418	2.7999817	down	NM_032097	chr5:140752306-140	PCDHGB3
A_23_P158318	2.3048483	down	NM_004560	chr9:094485526-094	ROR2
A_33_P3349883	2.0218942	down		chr1:236706380-236706439	

Homo sapiens v-myc	GO:0005515|GO:000	NM_005378	Hs.25960	4613	4.9999995
Homo sapiens annexi	GO:0006629|GO:000	NM_000700	Hs.494173	301	17211.52
Homo sapiens NEDD	GO:0008150|GO:000	NM_014887	Hs.507680	10443	1546.1119
Homo sapiens dynam	GO:0005515|GO:000	NM_012062	Hs.556296	10059	301.05093
Homo sapiens riboso	GO:0005515	NM_016052	Hs.660109	51018	1612.0835
Homo sapiens cyclin-	GO:0005515|GO:004	NM_003159	Hs.659851	6792	93.37866
Homo sapiens protei	GO:0005515|GO:000	NM_001123355	Hs.715605	5537	327.05096
Homo sapiens XIAP	GO:0005739|GO:000	NM_017523	Hs.441975	54739	23.845978
Homo sapiens media	GO:0006357|GO:001	NM_015335	Hs.603766	23389	662.8684
					5.7073903
					4.9999995
Homo sapiens cDNA FLJ33594 fis, clone BRAMY2012776 [AI			Hs.683952	100144595	4.9999995
Homo sapiens glycop	GO:0005886|GO:000	NM_001083899	Hs.661752	51206	17.634466
Homo sapiens multip	GO:0005515|GO:003	NM_003829	Hs.169378	8777	104.54202
Homo sapiens cDNA FLJ38333 fis, clone FCBBF3025674 [AK			Hs.494822	158402	8.625465
Homo sapiens solute	GO:0005515|GO:000	NM_003615	Hs.250072	9497	37.247837
Homo sapiens chromosome 20 open readir		NR_001558		140828	9.484694
					4.9999995
Homo sapiens chrom	GO:0016020|GO:001	NM_017847	Hs.371210	54953	478.42572
Homo sapiens SRY (	GO:0005515|GO:003	NM_017508	Hs.368226	55553	4.9999995
Homo sapiens cDNA FLJ35225 fis, clone PROST2001116 [AK			Hs.638992	100131581	4.9999995
Homo sapiens prolyl	GO:0005737|GO:000	NM_006036	Hs.444349	9581	381.91516
					13.857219
Homo sapiens galani	GO:0008150|GO:000	NM_033106	Hs.283915	85569	4.9999995
Homo sapiens cullin	GO:0005515|GO:003	NM_003591	Hs.82919	8453	417.3697
Homo sapiens chrom	GO:0008026|GO:000	NM_001010895	Hs.432364	375748	77.87288
Homo sapiens dopey	GO:0015031	NM_015018	Hs.520246	23033	96.68474
Homo sapiens oxyste	GO:0006869|GO:000	NM_020841	Hs.430849	114882	262.83185
Homo sapiens gon-4-	GO:0006355|GO:000	NM_032292	Hs.656361	54856	597.76294
Homo sapiens muscl	GO:0030018|GO:000	NM_001018116	Hs.99004	347273	34.629787
					16.588501
Homo sapiens UDP-g	GO:0005793|GO:001	NM_020121	Hs.193226	55757	202.21104
Homo sapiens zinc fi	GO:0005515|GO:000	NM_014827	Hs.532399	9877	947.82654
Homo sapiens myosi	GO:0048471|GO:000	NM_015011	Hs.656587	23026	10.010301
Homo sapiens zinc fi	GO:0005622|GO:000	NM_001039891	Hs.636105	641339	25.575153
Homo sapiens AF4/F	GO:0007275|GO:000	NM_002285	Hs.444414	3899	5.368988
Homo sapiens compl	GO:0006957|GO:000	NM_001014975	Hs.363396	3075	4.9999995
Rho GTPase-activating protein 26 (Rho-type GTPase-activating protein 26)(Oligophrenin-1-like protein)(G					4.9999995
Homo sapiens Fanco	GO:0006281|GO:000	NM_001018113	Hs.554740	2187	182.04088
Homo sapiens pro-m	GO:0046005|GO:000	NM_002674	Hs.707990	5367	5.3738685
Homo sapiens neurol	GO:0005802|GO:000	NM_015678	Hs.491172	26960	19.232664
Homo sapiens coiled-coil domain containi		NM_033124	Hs.512805	85478	4.9999995
Homo sapiens SMG1	GO:0005515|GO:003	NM_015092	Hs.460179	23049	200.64699
Homo sapiens DENN	GO:0016020|GO:001	NM_017925	Hs.249591	55667	146.51846
					4.9999995
Homo sapiens dynein	GO:0005794|GO:000	NM_001080463	Hs.503721	79659	41.18384
Homo sapiens melan	GO:0005515|GO:003	NM_198551	Hs.118474	375056	10.97573
PREDICTED: Homo sapiens similar to Pu		XM_002342513	Hs.711155	100133280	148.30513
					17.566032
Homo sapiens zinc fi	GO:0005622|GO:000	NM_181845	Hs.652513	284349	161.54567
Homo sapiens NEDD	GO:0005515|GO:004	NM_001018159	Hs.460978	8883	2698.5493
Homo sapiens espin-like (ESPNL), mRNA		NM_194312	Hs.127724	339768	6.5421786
Homo sapiens small	GO:0005737|GO:000	NM_005988	Hs.355542	6700	6.0798535
Homo sapiens anocta	GO:0031404|GO:001	NM_213599	Hs.154329	203859	67.38273
Homo sapiens alcoho	GO:0005739|GO:004	NM_144650	Hs.720023	137872	4.9999995
Homo sapiens protei	GO:0006629|GO:000	NM_002834	Hs.506852	5781	56.21196
					360.31323
Homo sapiens protoc	GO:0005515|GO:000	NM_032097	Hs.368160	56102	8.508456
Homo sapiens recept	GO:0005515|GO:000	NM_004560	Hs.98255	4920	4.9999995
Galectin-8 (Gal-8)(Prostate carcinoma tumor antigen 1)(PCTA-1)(Po66 carbohydrate-binding protein)(Po6					4.9999995

19.200716	2.3362613	4.1279507
38463.29	14.06761	15.236235
3701.6956	10.699438	11.792797
1116.2482	8.392103	10.002174
4324.2827	10.757898	12.015153
233.02998	6.680713	7.7195764
897.7496	8.508797	9.668587
64.19654	4.7148285	5.879242
1987.8302	9.516615	10.854872
25.023243	2.7033153	4.5133867
16.734251	2.3362613	3.9277754
25.606297	2.3362613	4.544301
45.219025	4.2759237	5.3669767
402.76892	6.836321	8.499369
22.05897	3.2723653	4.3311014
119.99252	5.3502355	6.774605
23.63363	3.4048605	4.428193
26.87722	2.3362613	4.6129856
1905.7687	9.049084	10.793585
24.433722	2.3362613	4.4767222
13.112082	2.3362613	3.5605707
1205.859	8.732431	10.116203
38.71852	3.9294033	5.1370945
31.82329	2.3362613	4.8569927
1065.2668	8.852059	9.927102
250.92198	6.4182353	7.818737
336.97543	6.72692	8.243306
782.8374	8.191446	9.46961
1653.8735	9.3616905	10.5804
104.35857	5.247875	6.568251
41.78778	4.1873894	5.2512517
638.18634	7.8111906	9.173214
3104.9517	10.010275	11.521524
28.624718	3.4780693	4.697693
67.34695	4.8153024	5.949551
14.107718	2.6208835	3.6751492
22.224648	2.3362613	4.34258
16.46849	2.3362613	3.899413
479.28638	7.65286	8.751562
29.289604	2.6222801	4.7336802
89.75004	4.4001894	6.352131
21.50941	2.3362613	4.2915964
539.6692	7.799713	8.925256
406.60342	7.3256965	8.511398
12.020399	2.3362613	3.4292572
154.8815	5.48995	7.144375
45.839287	3.6056514	5.3873043
385.65613	7.3420234	8.434103
54.22104	4.27106	5.6338058
424.11777	7.4701633	8.571301
6185.437	11.474155	12.560312
26.830826	2.8902726	4.611473
25.301249	2.7909884	4.5274725
220.6131	6.2035227	7.6423063
15.523031	2.3362613	3.8156018
138.81422	5.93771	6.987345
957.4715	8.646458	9.759803
29.332115	3.2498946	4.735312
12.944739	2.3362613	3.5409331
11.427561	2.3362613	3.3519688

A_23_P345081	2.1843954	down	NM_001083956	chr7:099173733-099	ZNF655
A_33_P3412678	7.2620542	down	NM_001037498	chr6:050011349-050	DEFB112
A_33_P3236646	2.2345464	down	NM_198551	chr1:222839087-222	MIA3
A_33_P3231950	2.3448987	down		chr4:37873666-37873725	
A_33_P3398548	3.7787812	down	NM_003666	chr1:169365416-169	BLZF1
A_23_P94902	4.1193226	down	NM_198353	chr4:044176402-044	KCTD8
A_32_P186226	2.3804957	down	NM_006391	chr1:211033095-211	IPO7
A_33_P3299656	2.2230472	down	NM_000328	chrX:038147267-038	RPGR
A_33_P3321324	2.091713	down	XM_001723511	chr2:91911303-9191	LOC100133086
A_24_P191833	4.0402064	down	NM_139168	chr5:065475935-065	SFRS12
A_33_P3228014	2.8769551	down	NM_206998	chr11:062063813-06	SCGB1D4
A_33_P3323218	2.6087818	down	NM_024744	chr2:203850935-203	ALS2CR8
A_33_P3606692	2.3380489	down	NR_027358	chr12:052617537-05	LOC283404
A_23_P84118	2.0832506	down	NM_004934	chr5:019473231-019	CDH18
A_33_P3716128	4.0379555	down	NM_005496	chr3:160151581-160	SMC4
A_33_P3401902	2.7108175	down	NM_001012421	chr1:143213153-143	ANKRD20A2
A_33_P3320808	3.1051503	down	AK127759	chr19:1389360-1389419	
A_33_P3314081	3.9726016	down	XM_001128809	chr10:102899601-10	LOC728940
A_24_P56484	2.0582782	down	NM_032352	chr14:036340379-03	BRMS1L
A_33_P3379681	3.5868614	down		chr14:038508276-038508217	
A_33_P3283944	3.2202918	down	XM_001720217	chr3:105085962-105	LOC100133690
A_33_P3273474	2.5396745	down	NM_001765	chr1:158264458-158	CD1C
A_33_P3259725	3.9202039	down		chr3:150910377-150910436	
A_33_P3349395	2.2365934	down		chr22:018019144-018019203	
A_23_P161719	3.0244259	down	NM_152434	chr11:107197449-10	CWF19L2
A_33_P3383169	3.6203789	down	AF447883	chr7:36125277-36125336	
A_23_P216094	2.2774576	down	NM_004318		ASPH
A_33_P3243399	2.424674	down	AY358410	chr3:57876874-5787	SLMAP
A_33_P3348086	2.4179845	down		chr4:004075543-004075602	
A_24_P286054	3.1726712	down	NM_001105251	chr5:079774799-079	ZFYVE16
A_23_P162525	3.5900925	down	NM_014503	chr12:101777351-10	UTP20
A_23_P156402	5.7063261	down	NM_003551		NME5
A_33_P3319625	3.6374709	down	NM_001134673	chr1:061892168-061	NFIA
A_24_P29594	3.899235	down	NM_006620	chr6:135282505-135	HBS1L
A_24_P942250	4.9973248	down	NM_025134	chr16:053360496-05	CHD9
A_24_P69691	2.8462433	down	NM_145011	chr10:038239838-03	ZNF25
A_23_P344988	2.1888757	down	NM_016513	chr6:052866185-052	ICK
A_24_P126210	4.8242989	down	NM_178817	chr21:033684326-03	MRAP
A_33_P3315325	2.2239897	down	BC050396	chr8:101522864-101522805	
A_33_P3290949	2.3730158	down		chr2:086363932-086363991	
A_33_P3344454	2.1718014	down	NM_001005326	chr15:102346802-10	OR4F6
A_32_P40288	2.9061654	down	NM_052913	chr6:130763993-130	TMEM200A
A_33_P3245218	2.2527362	down	NM_001122679	chr5:167689754-167	ODZ2
A_33_P3339070	2.0856522	down	NR_024475	chr10:004692436-00	LOC100216001
A_23_P135787	2.9528875	down	NM_004487	chr3:121382909-121	GOLGB1
A_33_P3390172	2.1280201	down	NM_001145271	chr8:024259534-024	ADAMDEC1
A_23_P412515	2.1190984	down	NM_012129	chr7:090042478-090	CLDN12
A_33_P3361412	2.8657598	down	NM_018420	chr1:116569583-116	SLC22A15
A_24_P942921	2.4515182	down	NM_152376	chr1:020519089-020	UBXN10
A_33_P3420992	2.6569968	down		chrX:061999677-061999736	
A_33_P3281139	4.0681137	down	BX114036	chr13:46531321-46531262	
A_23_P343900	4.5732719	down	XR_078810	chr14:107259489-107259430	
A_33_P3295233	2.2454541	down	AK095633	chr1:205438022-205	LOC284577
A_33_P3275381	3.4009882	down	NM_001039580	chr4:156289798-156	MAP9
A_33_P3292332	3.3312393	down	NM_004521	chr10:032298059-03	KIF5B
A_33_P3252281	4.1931304	down	NM_004100	chr6:133852598-133	EYA4
A_33_P3394390	2.6494274	down	XM_001723453	chr7:137642615-137	LOC100130880
A_24_P356	4.1457048	down		chr2:69708481-69708422	
A_33_P3418611	2.6746442	down	XM_002343636	chr19:22499726-22499785	
A_23_P156562	3.5539397	down	NM_206922	chr6:043273423-043	CRIP3

Homo sapiens zinc fi	GO:0005622|GO:000	NM_001083956	Hs.599798	79027	683.6582
Homo sapiens defens	GO:0005576|GO:004	NM_001037498	Hs.571092	245915	4.9999995
Homo sapiens melan	GO:0005515|GO:003	NM_198551	Hs.118474	375056	409.8695
Putative uncharacterized protein ENSP00000382756 [Source:UniProtKB/TrEMBL;Acc:A8MVS2] [ENST					88.66115
Homo sapiens basic l	GO:0005737|GO:000	NM_003666	Hs.130746	8548	15.428111
Homo sapiens potass	GO:0005515|GO:001	NM_198353	Hs.479644	386617	5.429978
Homo sapiens import	GO:0005215|GO:000	NM_006391	Hs.523470	10527	52.49005
Homo sapiens retiniti	GO:0005515|GO:000	NM_000328	Hs.61438	6103	58.425217
		XM_001723511	Hs.631791	100133086	11.1566
Homo sapiens splicin	GO:0008380|GO:000	NM_139168	Hs.519347	140890	581.59357
Homo sapiens secret	GO:0005488|GO:000	NM_206998	Hs.473247	404552	4.9999995
Homo sapiens amyot	GO:0005515|GO:000	NM_024744	Hs.444982	79800	13.26703
Homo sapiens hypothetical LOC283404 (L		NR_027358	Hs.662443	283404	5.3714004
Homo sapiens cadher	GO:0005515|GO:000	NM_004934	Hs.317632	1016	8.59558
Homo sapiens structu	GO:0005515|GO:000	NM_005496	Hs.58992	10051	1642.2603
Homo sapiens ankyrin repeat domain 20 fa		NM_001012421	Hs.632663	441430	104.75249
NADH dehydrogenase [ubiquinone] iron-sulfur protein 7, mitochondrial Precursor (EC 1.6.5.3)(EC 1.6.99.3					4.9999995
PREDICTED: Homo sapiens hypothetical		XM_001128809	Hs.628713	728940	4.9999995
Homo sapiens breast	GO:0005634|GO:004	NM_032352	Hs.525299	84312	248.7104
					6.76794
PREDICTED: Homo sapiens hypothetical		XM_001720217	Hs.625330	100133690	16.459648
Homo sapiens CD1c	GO:0006955|GO:000	NM_001765	Hs.132448	911	4.9999995
Mediator of RNA polymerase II transcription subunit 12-like protein (Mediator complex subunit 12-like pro					4.9999995
					84.23484
Homo sapiens CWF19-like 2, cell cycle co		NM_152434	Hs.212140	143884	91.96057
Hypothetical gene supported by AF447883		Putative uncharacteri	Hs.529590		4.9999995
Homo sapiens aspart	GO:0007389|GO:000	NM_004318	Hs.591874	444	84.58145
Homo sapiens clone	GO:0005813|GO:0006936|GO:0005886|G		Hs.476432	7871	198.71129
					11.483999
Homo sapiens zinc fi	GO:0005515|GO:003	NM_001105251	Hs.482660	9765	250.20406
Homo sapiens UTP2	GO:0005515|GO:000	NM_014503	Hs.295732	27340	621.6738
Homo sapiens non-m	GO:0006241|GO:000	NM_003551	Hs.519602	8382	4.9999995
Homo sapiens nuclea	GO:0006260|GO:000	NM_001134673	Hs.191911	4774	4.9999995
Homo sapiens HBS1	GO:0007165|GO:000	NM_006620	Hs.378532	10767	12.974388
Homo sapiens chrom	GO:0005515|GO:000	NM_025134	Hs.59159	80205	44.212196
Homo sapiens zinc fi	GO:0005622|GO:000	NM_145011	Hs.499429	219749	22.11666
Homo sapiens intesti	GO:0007165|GO:000	NM_016513	Hs.417022	22858	1777.1986
Homo sapiens melan	GO:0048471|GO:000	NM_178817	Hs.584940	56246	5.2788563
Ankyrin repeat domain-containing protein 46 (Ankyrin repeat sm			Hs.530199		4.9999995
					50.66505
Homo sapiens olfact	GO:0007608|GO:000	NM_001005326	Hs.553399	390648	4.9999995
Homo sapiens transm	GO:0016020|GO:001	NM_052913	Hs.591341	114801	4.9999995
Homo sapiens odz, o	GO:0007165|GO:001	NM_001122679	Hs.631957	57451	7.7061567
Homo sapiens hypothetical LOC10021600		NR_024475		100216001	5.8551755
Homo sapiens golgin	GO:0005515|GO:000	NM_004487	Hs.213389	2804	5.3407345
Homo sapiens ADAM	GO:0007162|GO:000	NM_001145271	Hs.521459	27299	4.9999995
Homo sapiens claudi	GO:0030054|GO:001	NM_012129	Hs.258576	9069	32.991577
Homo sapiens solute	GO:0005215|GO:001	NM_018420	Hs.125482	55356	7.348941
Homo sapiens UBX domain protein 10 (U		NM_152376	Hs.432503	127733	9.83666
					1885.9951
Zinc finger CCCH domain-containing protein 13 [Source:UniPr			Hs.712794		112.81468
Putative uncharacterized protein ENSP000		XR_078810	Hs.81221		6.0737076
Homo sapiens cDNA FLJ38314 fis, clone FCBBF3022765 [AK			Hs.434167	284577	7.3743987
Homo sapiens micro	GO:0007067|GO:000	NM_001039580	Hs.61271	79884	19.58626
Homo sapiens kinesi	GO:0005515|GO:000	NM_004521	Hs.327736	3799	2951.6687
Homo sapiens eyes a	GO:0000287|GO:000	NM_004100	Hs.596680	2070	4.9999995
PREDICTED: Homo sapiens hypothetical		XM_001723453	Hs.712161	100130880	5.5491614
AP2-associated protein kinase 1 (EC 2.7.11.1)(Adaptor-associated kinase 1) [Source:UniProtKB/Swiss-Pro					11.5728
Zinc finger protein ENSP00000350085 [S		XM_002343636			781.6858
Homo sapiens cystei	GO:0005737|GO:004	NM_206922	Hs.653165	401262	5.316064

1773.3671	9.557688	10.684922
40.191666	2.3362613	5.196639
1106.8768	8.828824	9.988806
254.43591	6.60715	7.8366756
69.65004	4.0816193	5.9995403
28.23206	2.6369047	4.6793118
149.34193	5.836253	7.087515
154.87091	5.9914584	7.143997
28.484518	3.6272068	4.6918917
2749.4834	9.319412	11.333841
15.99508	2.3362613	3.860804
41.85218	3.8705013	5.2538776
15.85276	2.6219053	3.8472104
21.960762	3.265699	4.3245354
7284.042	10.783056	12.796681
346.10666	6.839709	8.278437
17.17484	2.3362613	3.9709244
21.99018	2.3362613	4.3263454
630.9271	8.116382	9.15782
30.134523	2.933302	4.776024
63.42954	4.1758766	5.863068
14.17296	2.3362613	3.6809049
21.738726	2.3362613	4.30719
228.59958	6.531845	7.693148
340.41165	6.6593447	8.256006
20.070093	2.3362613	4.192402
233.99945	6.5377164	7.7251406
591.0726	7.7832212	9.061012
33.874542	3.665241	4.939046
978.81195	8.124887	9.790585
2623.9229	9.420771	11.264792
31.631054	2.3362613	4.8488235
20.176323	2.3362613	4.199197
60.776844	3.8388953	5.8020864
268.203	5.5906463	7.9118023
75.348854	4.601182	6.110241
4379.452	10.899804	12.029994
32.112087	2.5964687	4.866788
12.498001	2.3362613	3.4894114
142.98509	5.78475	7.0314717
12.225749	2.3362613	3.4551535
16.17666	2.3362613	3.8753781
21.528828	3.1217856	4.293464
15.382682	2.7413044	3.801803
19.83974	2.6127286	4.174855
11.998141	2.3362613	3.4257731
83.86631	5.1772904	6.260741
26.07778	3.0506673	4.569585
29.615957	3.4547958	4.7484713
5530.039	10.9783535	12.38815
556.9937	6.945372	8.969732
34.921352	2.7897043	4.982931
20.52398	3.0555298	4.222537
79.91548	4.425664	6.191618
10501.75	11.606289	13.342348
23.235113	2.3362613	4.404289
18.393112	2.66426	4.0699406
57.6006	3.6763396	5.727957
2462.2383	9.753022	11.172369
23.791712	2.607942	4.4373612

A_33_P3402565	2.2877589	down	NM_004415	chr6:007586052-007	DSP
A_24_P116909	2.4226752	down	NM_006785	chr18:056416474-05	MALT1
A_33_P3322589	2.1812604	down	NM_001114636	chr2:058387309-058	FANCL
A_33_P3403887	4.0017943	down	NM_014522	chrY:004968722-004	PCDH11X
A_23_P347169	2.21515	down	NM_001001924	chr8:017502332-017	MTUS1
A_24_P339869	3.0580294	down	NM_020727	chr21:043407139-04	ZNF295
A_33_P3281363	2.6740066	down	AK124509	chr2:230695852-230695793	
A_23_P139558	2.1486761	down	NM_022771	chr12:072316935-07	TBC1D15
A_23_P428298	2.1138682	down	NM_173561	chr6:040994949-040	UNC5CL
A_33_P3280993	2.882921	down	NR_024052	chr6:030255233-030	HCG18
A_33_P3268783	2.7722726	down	NM_133463	chr7:002755002-002	AMZ1
A_33_P3383044	2.6953819	down	XM_001713998	chr17:019274663-01	LOC100130116
A_33_P3237886	2.1276052	down	AK131480	chr10:9450245-9450	LOC389936
A_33_P3420701	2.5603256	down		chr2:160261088-160261147	
A_33_P3244096	2.6256305	down		chrX:41418818-41418759	
A_24_P349151	3.2813125	down	NM_194292	chr1:100549656-100	SASS6
A_33_P3369939	2.2313766	down	NM_194283	chr5:034958867-034	DNAJC21
A_23_P213166	3.4800945	down	NM_018392	chr4:113460845-113	C4orf21
A_24_P358868	2.5316576	down	XM_001720936	chr19:023158862-02	ZNF728
A_33_P3343750	2.3962337	down	NM_152603	chr19:037211307-03	ZNF567
A_23_P88331	3.0516976	down	NM_014750	chr14:055615172-05	DLGAP5
A_33_P3332329	2.4736761	down	NM_032681	chr11:049855002-04	SPRYD5
A_23_P114740	2.1619532	down	NM_000186	chr1:196716550-196	CFH
A_23_P79247	2.3471776	down	NM_024989		PGAP1
A_23_P56659	2.8162988	down	NM_133637	chr2:074745410-074	DQX1
A_23_P141306	2.2024258	down	NM_004246	chr17:009783742-00	GLP2R
A_33_P3421973	2.147974	down	XM_001714381	chrX:106846343-106	FRMPD3
A_33_P3387951	2.4538244	down	NM_020340	chr6:138662672-138	KIAA1244
A_23_P29830	2.584479	down	NM_170662	chr3:105400640-105	CBLB
A_24_P398500	3.6930293	down	NM_022782	chr12:123646722-12	MPHOSPH9
A_33_P3210228	2.2910361	down		chr9:88959844-88959785	
A_33_P3236628	3.9927834	down		chr19:41937191-41937250	
A_33_P3234555	3.0305873	down		chr14:098047862-098047921	
A_33_P3248903	2.199103	down	NM_058238	chr22:046316713-04	WNT7B
A_23_P204564	2.0156205	down	NM_002480	chr12:080168945-08	PPP1R12A
A_33_P3399019	2.3207332	down	NM_001031665	chr19:053452784-05	ZNF816A
A_33_P3289705	4.9990314	down	NM_004487	chr3:121412749-121	GOLGB1
A_32_P489130	4.3369629	down	NM_153252	chrX:079931980-079	BRWD3
A_33_P3264424	2.0706287	down		chr8:064100250-064100309	
A_33_P3336287	2.0146543	down	NM_001142627	chr10:012191853-01	SEC61A2
A_24_P408704	2.0995114	down	NM_004946	chr5:169506070-169	DOCK2
A_33_P3269885	3.466349	down	NM_018713	chr1:220087746-220	SLC30A10
A_23_P140434	2.1934776	down	NM_018728	chr15:052484703-05	MYO5C
A_23_P417853	2.018663	down	NM_138995	chr2:171510533-171	MYO3B
A_23_P67569	3.3354361	down	NM_024888	chr19:000812797-00	LPPR3
A_32_P925529	4.9888065	down	XR_015594	chr12:069679125-06	LOC729409
A_33_P3291484	3.3453195	down	NM_005668	chr5:100143016-100	ST8SIA4
A_33_P3381821	3.2895693	down	NM_001145122	chr2:031399952-031	CAPN14
A_24_P223604	2.1515465	down	NM_015496	chr8:095500972-095	KIAA1429
A_33_P3288329	2.0476724	down	NM_001034954	chr10:097074875-09	SORBS1
A_23_P214897	2.7461263	down	NM_144497	chr6:151670006-151	AKAP12
A_33_P3361352	6.6815045	down	XM_001129872	chr20:058674055-05	LOC729296
A_23_P43580	4.2589695	down	NM_007018	chr9:123935686-123	CEP110
A_33_P3257554	4.600473	down	NM_007018	chr9:123920258-123	CEP110
A_23_P376036	2.2289182	down	NM_152596	chr15:041475122-04	EXD1
A_33_P3335371	2.1201495	down	NM_018717	chr4:140640536-140	MAML3
A_32_P34003	4.7655592	down		chr2:164459505-164	FIGN
A_24_P217365	2.4004419	down	NM_015199	chr3:015708901-015	ANKRD28
A_23_P74349	2.7466165	down	NM_145697	chr1:163325144-163	NUF2
A_23_P132763	2.8471595	down	NM_016206	chr3:086987218-086	VGLL3

Homo sapiens desmo	GO:0030057|GO:000	NM_004415	Hs.519873	1832	414.62915
Homo sapiens mucos	GO:0004842|GO:004	NM_006785	Hs.601217	10892	70.80178
Homo sapiens Fanco	GO:0005515|GO:000	NM_001114636	Hs.720331	55120	419.36893
Homo sapiens protoc	GO:0005515|GO:000	NM_014522	Hs.655673	27328	4.9999995
Homo sapiens microt	GO:0005739|GO:000	NM_001001924	Hs.7946	57509	37.05543
Homo sapiens zinc fi	GO:0005622|GO:000	NM_020727	Hs.434947	49854	155.75421
Probable E3 ubiquitin-protein ligase TRIP12 (EC 6.3.2.-)(Thyro			Hs.591633		15.897128
Homo sapiens TBC1	GO:0005739|GO:000	NM_022771	Hs.284630	64786	507.1075
Homo sapiens unc-5	GO:0005515|GO:000	NM_173561	Hs.158357	222643	18.04129
Homo sapiens HLA complex group 18 (H(		NR_024052		414777	168.34021
Homo sapiens archae	GO:0008270|GO:000	NM_133463	Hs.42221	155185	4.9999995
PREDICTED: Homo sapiens hypothetical		XM_001713998		100130116	4.9999995
Homo sapiens cDNA FLJ16652 fis, clone TESTI4036767 [AK1			Hs.532673	389936	6.1073794
					4.9999995
Peripheral plasma membrane protein CASK (hCASK)(EC 2.7.11.1)(Calcium/calmodulin-dependent serine						4.9999995
Homo sapiens spindl	GO:0007049|GO:000	NM_194292	Hs.591447	163786	86.76978
Homo sapiens DnaJ (	GO:0005622|GO:000	NM_194283	Hs.131887	134218	306.32388
Homo sapiens chrom	GO:0016020|GO:000	NM_018392	Hs.380346	55345	91.66073
PREDICTED: Homo sapiens zinc finger p		XM_001720936	Hs.616766	388523	90.463165
Homo sapiens zinc fi	GO:0005622|GO:000	NM_152603	Hs.412517	163081	191.24655
Homo sapiens discs,	GO:0005515|GO:000	NM_014750	Hs.77695	9787	2995.4546
Homo sapiens SPRY domain containing 5		NM_032681	Hs.326734	84767	8.164873
Homo sapiens compl	GO:0006957|GO:000	NM_000186	Hs.363396	3075	16.879692
Homo sapiens post-C	GO:0042578|GO:000	NM_024989	Hs.229988	80055	31.167402
Homo sapiens DEAQ	GO:0016787|GO:000	NM_133637	Hs.191705	165545	13.048961
Homo sapiens glucag	GO:0008284|GO:000	NM_004246	Hs.248202	9340	4.9999995
FERM and PDZ dom	GO:0005515|GO:000	XM_001714381	Hs.496546	84443	4.9999995
Homo sapiens KIAA	GO:0005622|GO:000	NM_020340	Hs.194408	57221	8.832354
Homo sapiens Cas-B	GO:0005515|GO:000	NM_170662	Hs.430589	868	4.9999995
Homo sapiens M-pha	GO:0005794|GO:001	NM_022782	Hs.577404	10198	152.96251
Zinc finger CCHC domain-containing protein 6 [Source:UniProtKB/Swiss-Prot;Acc:Q5VYS8] [ENST000(					7.94427
Transmembrane protein 91 [Source:UniProtKB/Swiss-Prot;Acc:Q6ZNR0] [ENST00000378196]					8.887338
					4.9999995
Homo sapiens wingle	GO:0007267|GO:000	NM_058238	Hs.512714	7477	4.9999995
Homo sapiens protei	GO:0005515|GO:000	NM_002480	Hs.49582	4659	295.2748
Homo sapiens zinc fi	GO:0005622|GO:000	NM_001031665	Hs.655592	125893	114.23181
Homo sapiens golgin	GO:0005515|GO:000	NM_004487	Hs.213389	2804	31.056849
Homo sapiens bromodomain and WD repe		NM_153252	Hs.170667	254065	6.628487
					86.83517
Homo sapiens Sec61	GO:0015450|GO:000	NM_001142627	Hs.112955	55176	7.9845176
Homo sapiens dedica	GO:0005515|GO:000	NM_004946	Hs.586174	1794	5.3695397
Homo sapiens solute	GO:0005886|GO:000	NM_018713	Hs.284450	55532	4.9999995
Homo sapiens myosi	GO:0005516|GO:000	NM_018728	Hs.487036	55930	110.225784
Homo sapiens myosi	GO:0005737|GO:000	NM_138995	Hs.671900	140469	4.9999995
Homo sapiens lipid p	GO:0016787|GO:001	NM_024888	Hs.546439	79948	4.9999995
PREDICTED: Homo sapiens similar to co		XR_015594	Hs.587721	729409	4.9999995
Homo sapiens ST8 al	GO:0003828|GO:003	NM_005668	Hs.308628	7903	10.069259
Homo sapiens calpai	GO:0005622|GO:000	NM_001145122	Hs.468059	440854	4.9999995
Homo sapiens KIAA	GO:0008380|GO:000	NM_015496	Hs.202238	25962	1704.6693
Homo sapiens sorbin	GO:0008286|GO:000	NM_001034954	Hs.595068	10580	5.6260047
Homo sapiens A kina	GO:0007165|GO:000	NM_144497	Hs.371240	9590	163.36363
PREDICTED: Homo sapiens similar to hC		XM_001129872	Hs.534781	729296	7.0792804
Homo sapiens centro	GO:0005515|GO:000	NM_007018	Hs.653263	11064	61.85789
Homo sapiens centro	GO:0005515|GO:000	NM_007018	Hs.653263	11064	44.634453
Homo sapiens exonu	GO:0005622|GO:000	NM_152596	Hs.307999	161829	4.9999995
Homo sapiens maste	GO:0016607|GO:000	NM_018717	Hs.586165	55534	151.88908
Fidgetin [Source:Un	GO:0017111|GO:0000166|GO:0005524|GO:0016363			55137	19.27771
Homo sapiens ankyri	GO:0005515|GO:000	NM_015199	Hs.335239	23243	1017.27484
Homo sapiens NUF2	GO:0005515|GO:000	NM_145697	Hs.651950	83540	1929.1832
Homo sapiens vestigi	GO:0030528|GO:000	NM_016206	Hs.720159	389136	39.284607

1146.1737	8.845097	10.039032
206.47395	6.276783	7.553384
1102.943	8.857751	9.982913
22.13064	2.3362613	4.3369083
99.06188	5.343817	6.4912214
579.1635	7.4154787	9.028081
50.872417	4.1249557	5.5439587
1307.0809	9.130483	10.233931
45.86655	4.30787	5.3877554
591.3	7.5338087	9.06134
15.442541	2.3362613	3.8073304
15.001609	2.3362613	3.766751
16.329918	2.798234	3.8874645
14.28254	2.3362613	3.6925886
14.6218195	2.3362613	3.7289252
349.15628	6.575892	8.290165
843.33563	8.417841	9.575775
389.85825	6.6527915	8.451918
280.78662	6.6358967	7.975979
562.1352	7.7245064	8.985275
9750.314	11.627611	13.237223
24.840086	3.1962094	4.502866
44.077744	4.2136917	5.326027
88.18856	5.099463	6.33039
44.375416	3.8456926	5.339493
12.38447	2.3362613	3.4753547
12.103351	2.3362613	3.4392378
26.651556	3.3068757	4.6019077
14.415101	2.3362613	3.7061348
680.7054	7.3894973	9.274302
22.455412	3.1610298	4.35703
43.62319	3.3142908	5.3116856
16.830612	2.3362613	3.9358587
12.36783	2.3362613	3.4731765
730.7529	8.362253	9.373477
322.9899	6.9659023	8.180483
187.41507	5.0952377	7.4168863
35.8207	2.9051545	5.0218396
218.15942	6.5774784	7.6275473
19.87766	3.1671557	4.177688
14.272069	2.621562	3.6916156
19.22458	2.3362613	4.1296782
293.94333	6.912423	8.045643
11.412198	2.3362613	3.3496614
18.466654	2.3362613	4.0741367
27.64816	2.3362613	4.654956
41.125854	3.486003	5.228147
18.166828	2.3362613	4.05416
4109.851	10.835351	11.940725
14.505321	2.6829846	3.7169695
546.4102	7.486244	8.943642
57.98454	2.999268	5.739441
319.91284	6.0743666	8.164871
248.50021	5.6038065	7.8055887
12.521272	2.3362613	3.492605
393.24475	7.380579	8.464745
110.83368	4.4034505	6.656096
2823.6145	10.111351	11.374651
5807.894	11.011496	12.4691515
133.97334	5.428701	6.9382243

A_23_P413180	2.2953283	down	NM_017440	chr12:068688887-06	MDM1
A_33_P3422659	2.3892983	down	NM_018972	chr8:075279274-075	GDAP1
A_23_P165239	3.2499146	down	NM_007153	chr19:022154324-02	ZNF208
A_23_P306215	3.0889859	down	NM_145175	chr2:014776066-014	FAM84A
A_33_P3407350	2.2935647	down	NM_001029865	chr11:020177820-02	DBX1
A_23_P163481	2.1635208	down	NM_001211	chr15:040512828-04	BUB1B
A_24_P178819	4.6659544	down		chr14:22356548-22356607	
A_32_P133916	5.8268049	down	NM_017637	chr9:016409646-016	BNC2
A_24_P113144	3.5556443	down	NM_024857	chr17:029221595-02	ATAD5
A_23_P352291	2.1505706	down	NM_054016	chr1:024296306-024	SFRS13A
A_23_P88489	2.6565397	down	NM_172095	chr15:043922931-04	CATSPER2
A_23_P89327	2.3228573	down	NM_020652	chr17:015620700-01	ZNF286A
A_24_P769359	2.0671083	down	NM_018979	chr12:001020048-00	WNK1
A_33_P3418766	2.183898	down	NM_018181	chr8:137877126-137	ZNF532
A_33_P3253169	5.805079	down	NM_207412	chr8:038368414-038	C8orf86
A_24_P914817	4.3457585	down	NM_013255	chr7:131180695-131	MKLN1
A_33_P3285987	2.0798882	down	NM_152636	chr3:156429344-156	METT5D1
A_23_P86660	2.2722318	down	NM_194247	chr10:044282975-04	HNRNPA3
A_33_P3395513	2.2509937	down		chr5:139230918-139230859	
A_33_P3279388	4.3184563	down		chr6:062674054-062674113	
A_33_P3347281	4.8486315	down	NM_001024075	chr2:138739135-138	HNMT
A_23_P70371	4.1286954	down	NM_015153	chr6:064423669-064	PHF3
A_33_P3274731	3.0802456	down	NM_004859	chr17:057774258-05	CLTC
A_33_P3267740	2.1327761	down	NM_178544	chr19:040521518-04	ZNF546
A_33_P3301306	2.7709429	down	NM_170606	chr7:151832089-151	MLL3
A_23_P154025	2.5071842	down	NM_003142	chr2:170668314-170	SSB
A_24_P252780	2.1534338	down	NM_198514	chr10:115664617-11	NHLRC2
A_23_P89780	2.0555844	down	NM_198129	chr18:021534735-02	LAMA3
A_24_P235429	2.7737383	down	NM_005502	chr9:107543763-107	ABCA1
A_23_P217498	22.2111949	down	NM_017711		GDPD2
A_32_P162374	2.0510476	down	NM_173529	chr18:051907485-05	C18orf54
A_33_P3696837	2.3277232	down	BC040565	chr2:174062527-174	LOC339751
A_33_P3247117	2.4482305	down	NM_004709	chrX:144909424-14	CXorf1
A_23_P162766	2.1951299	down	NM_015296	chr13:099446650-09	DOCK9
A_23_P332908	2.4960996	down	NM_144982	chr12:072050740-07	ZFC3H1
A_23_P205841	2.663794	down	NM_006901	chr15:072118519-07	MYO9A
A_23_P27180	4.2496705	down	NM_032141	chr17:028512741-02	CCDC55
A_33_P3238335	3.5360949	down	BC060765	chr1:85490292-85490233	
A_23_P388150	6.3965678	down	NM_032562	chr10:074695408-07	PLA2G12B
A_33_P3248967	2.0451349	down	NM_022474	chr14:067802437-06	MPP5
A_24_P573533	2.2811685	down	NM_001024916	chr9:000121398-000	CBWD5
A_33_P3286937	2.2555185	down	NM_001166163	chr7:094897959-094	PPP1R9A
A_33_P3418511	2.4603076	down	NR_027756	chr16:073127613-07	HTA
A_24_P360529	3.185443	down	NM_002604	chr8:066634719-066	PDE7A
A_24_P945194	3.0148516	down	NM_013374	chr3:033910819-033	PDCD6IP
A_23_P371729	2.0331914	down	NM_005266	chr1:147229843-147	GJA5
A_33_P3267186	3.2420762	down	NM_001682	chr12:089993004-08	ATP2B1
A_23_P125639	2.0490044	down	NM_003410	chrX:024228852-02	ZFX
A_23_P346006	2.9091043	down	NM_020739	chr15:055648495-05	CCPG1
A_33_P3242829	2.0257348	down		chr1:89271761-89271820	
A_33_P3374293	2.264551	down	NM_024854	chr11:106694832-10	PYROXD1
A_23_P403588	3.7863327	down	NM_017944	chr11:011980395-01	USP47
A_23_P19619	2.9473711	down	NM_002114	chr6:012164823-012	HIVEP1
A_33_P3319026	2.434781	down	NM_015534	chr1:078030632-078	ZZZ3
A_32_P790361	2.6671005	down	NM_007138	chr19:020228630-02	ZNF90
A_23_P157758	2.8815359	down	NM_001017969	chr9:005919881-005	KIAA2026
A_24_P165450	2.7704135	down	NM_024686		TTLL7
A_24_P193435	2.3788116	down	NM_003257	chr15:029992586-02	TJP1
A_33_P3279590	2.869217	down	NM_033014	chr9:095147506-095	OGN
A_33_P3296592	2.7717947	down	NM_020193	chr11:076262527-07	C11orf30

Homo sapiens Mdm1	GO:0005634	NM_017440	Hs.655702	56890	94.47177
Homo sapiens gangli	GO:0005737|GO:003	NM_018972	Hs.168950	54332	24.912209
Homo sapiens zinc fi	GO:0005622|GO:000	NM_007153	Hs.541334	7757	27.633379
Homo sapiens family with sequence simila		NM_145175	Hs.260855	151354	4.9999995
Homo sapiens develc	GO:0043565|GO:000	NM_001029865	Hs.558604	120237	16.801193
Homo sapiens buddir	GO:0005515|GO:000	NM_001211	Hs.513645	701	470.59265
Putative uncharacterized protein ENSP00000374944 [Source:UniProtKB/TrEMBL;Acc:A6NNK0] [ENST					4.9999995
Homo sapiens basonı	GO:0005622|GO:000	NM_017637	Hs.656581	54796	4.9999995
Homo sapiens ATPa:	GO:0017111|GO:000	NM_024857	Hs.528902	79915	92.51744
Homo sapiens splicir	GO:0000244|GO:000	NM_054016	Hs.3530	10772	646.7083
Homo sapiens cation	GO:0005515|GO:000	NM_172095	Hs.662284	117155	53.844772
Homo sapiens zinc fi	GO:0005622|GO:000	NM_020652	Hs.585799	57335	26.472084
Homo sapiens WNK	GO:0005515|GO:000	NM_018979	Hs.356604	65125	381.2394
Homo sapiens zinc fi	GO:0005622|GO:000	NM_018181	Hs.529023	55205	306.36783
Homo sapiens chromosome 8 open reading		NM_207412	Hs.546586	389649	4.9999995
Homo sapiens muske	GO:0005515|GO:000	NM_013255	Hs.44693	4289	70.28039
Homo sapiens methy	GO:0008168|GO:001	NM_152636	Hs.243326	196074	188.41185
Homo sapiens hetero	GO:0008380|GO:000	NM_194247	Hs.516539	220988	589.35004
					6.994535
					4.9999995
Homo sapiens histam	GO:0005737|GO:000	NM_001024075	Hs.42151	3176	6.0873556
Homo sapiens PHD f	GO:0005515|GO:000	NM_015153	Hs.348921	23469	644.1309
Homo sapiens clathri	GO:0005739|GO:000	NM_004859	Hs.491351	1213	5.2705545
Homo sapiens zinc fi	GO:0005622|GO:000	NM_178544	Hs.666807	339327	100.75092
Homo sapiens myelo	GO:0005515|GO:000	NM_170606	Hs.647120	58508	404.6744
Homo sapiens Sjogre	GO:0005515|GO:000	NM_003142	Hs.632535	6741	3796.879
Homo sapiens NHL r	GO:0045454	NM_198514	Hs.369924	374354	73.76914
Homo sapiens lamini	GO:0045995|GO:003	NM_198129	Hs.436367	3909	155.85652
Homo sapiens ATP-b	GO:0006629|GO:000	NM_005502	Hs.429294	19	26.4286
Homo sapiens glycer	GO:0006629|GO:000	NM_017711	Hs.438712	54857	5.9469333
Homo sapiens chrom	GO:0005576	NM_173529	Hs.208701	162681	151.62279
Homo sapiens hypothetical protein LOC339751, mRNA (cDNA			Hs.623925	339751	4.9999995
Homo sapiens chrom	GO:0008150|GO:000	NM_004709	Hs.106688	9142	4.9999995
Homo sapiens dedica	GO:0005085|GO:000	NM_015296	Hs.596105	23348	156.1684
Homo sapiens zinc fi	GO:0005622|GO:000	NM_144982	Hs.527874	196441	741.5509
Homo sapiens myosi	GO:0005515|GO:000	NM_006901	Hs.546268	4649	336.50443
Homo sapiens coiled	GO:0016607	NM_032141	Hs.462663	84081	522.84485
Mucolipin-3 [Source:UniProtKB/Swiss-Prot;Acc:Q8TDD5] [EI			Hs.535239		97.60492
Homo sapiens phospl	GO:0016042|GO:000	NM_032562	Hs.333175	84647	4.9999995
Homo sapiens memb	GO:0005515|GO:000	NM_022474	Hs.652312	64398	3618.2573
Homo sapiens COBV	GO:0000166|GO:000	NM_001024916	Hs.645337	220869	298.8801
Homo sapiens protei	GO:0005515|GO:000	NM_001166163	Hs.21816	55607	5.1774483
Homo sapiens hypothetical LOC283902 (F		NR_027756	Hs.382776	283902	8.567213
Homo sapiens phospl	GO:0007165|GO:001	NM_002604	Hs.527119	5150	13.92347
Homo sapiens progra	GO:0042470|GO:000	NM_013374	Hs.475896	10015	238.05957
Homo sapiens gap ju	GO:0005243|GO:000	NM_005266	Hs.447968	2702	4.9999995
Homo sapiens ATPa:	GO:0005516|GO:000	NM_001682	Hs.506276	490	388.21878
Homo sapiens zinc fi	GO:0005622|GO:003	NM_003410	Hs.336681	7543	53.972828
Homo sapiens cell cy	GO:0007049|GO:001	NM_020739	Hs.285051	9236	40.446342
Serine/threonine-protein kinase N2 (EC 2.7.11.13)(Protein kinase C-like 2)(Protein-kinase C-related kinase					8.916992
Homo sapiens pyridi	GO:0016491|GO:000	NM_024854	Hs.709545	79912	67.79953
Homo sapiens ubiqui	GO:0006511|GO:000	NM_017944	Hs.577256	55031	60.73391
Homo sapiens humar	GO:0016564|GO:000	NM_002114	Hs.567284	3096	81.01507
Homo sapiens zinc fi	GO:0008270|GO:000	NM_015534	Hs.480506	26009	748.2535
Homo sapiens zinc fi	GO:0005622|GO:000	NM_007138	Hs.55304	7643	5.9799075
Homo sapiens KIAA2026 (KIAA2026), m		NM_001017969	Hs.535060	158358	14.552169
Homo sapiens tubuli	GO:0005929|GO:000	NM_024686	Hs.445826	79739	26.43669
Homo sapiens tight ju	GO:0005515|GO:001	NM_003257	Hs.510833	7082	2183.3765
Homo sapiens osteog	GO:0005515|GO:000	NM_033014	Hs.109439	4969	9.285841
Homo sapiens chrom	GO:0006281|GO:000	NM_020193	Hs.352588	56946	74.52404

265.00122	6.6944575	7.893158
71.266975	4.776162	6.032749
109.03523	4.9303546	6.6307564
17.081602	2.3362613	3.9633946
46.50406	4.206443	5.4040346
1229.1765	9.028081	10.141462
25.854162	2.3362613	4.5584335
32.448	2.3362613	4.8789663
402.60016	6.668679	8.49879
1656.4852	9.4790535	10.583773
171.11656	5.8745537	7.284102
73.58582	4.863388	6.0792885
970.5187	8.729957	9.777571
823.30255	8.4182415	9.545147
32.292564	2.3362613	4.873577
372.9021	6.263203	8.382811
481.5647	7.702863	8.759369
1592.7039	9.341248	10.525358
19.52143	2.980971	4.151533
23.947859	2.3362613	4.446777
37.02889	2.7939534	5.071531
3096.0608	9.473309	11.518995
20.452208	2.5947719	4.2178173
262.7535	6.7875075	7.88024
1350.1543	8.810471	10.280848
10101.971	11.958298	13.284366
190.95834	6.337103	7.443742
391.11505	7.4171124	8.456661
88.26607	4.860118	6.3319497
165.33495	2.7616901	7.2349052
380.7657	7.378014	8.414375
13.06335	2.3362613	3.5551808
13.697551	2.3362613	3.6280007
418.90457	7.4195347	8.553841
2185.5757	9.67877	10.9984455
1086.5687	8.5494995	9.962982
2618.0928	9.175058	11.262409
421.2755	6.741232	8.563389
35.54254	2.3362613	5.0135593
7914.6445	11.882317	12.914513
840.65344	8.383118	9.572891
14.719041	2.5684845	3.7419436
25.855717	3.260271	4.5591097
53.12345	3.936573	5.608067
881.2725	8.048198	9.640285
11.4845495	2.3362613	3.3600073
1516.6528	8.753471	10.450389
131.8042	5.8796105	6.9145336
140.6576	5.4686337	7.0092087
22.14725	3.31936	4.3378053
184.07549	6.213203	7.392428
279.1327	6.0476255	7.9684267
292.43607	6.4794073	8.038836
2158.139	9.694506	10.978298
19.97368	2.7700272	4.1852994
50.213203	3.997642	5.52448
88.23063	4.8612957	6.331397
5662.941	11.184977	12.435218
32.84876	3.3758473	4.8965044
252.26929	6.3553486	7.826169

A_24_P943472	2.0058735	down	NM_005126	chr3:024021460-024	NR1D2
A_33_P3412349	2.9557917	down	NM_152943	chr12:133779282-13	ZNF268
A_24_P917026	2.8530584	down	NM_001042492	chr17:029704560-02	NF1
A_23_P145501	2.2855494	down	NM_004830	chr6:131908847-131	MED23
A_23_P9056	3.1288901	down	NM_014781	chr8:053536078-053	RB1CC1
A_33_P3359518	2.4358622	down	NM_001085487	chr1:059125723-059	MYSM1
A_33_P3238215	3.7718937	down	NM_014900	chr2:165541428-165	COBLL1
A_33_P3354955	4.0976677	down	NM_016649	chr20:013695615-01	ESF1
A_32_P201773	2.1398051	down	NM_015365	chrX:109437714-10(	AMMECR1
A_23_P132738	2.6645537	down	NM_017541	chr3:186256728-186	CRYGS
A_32_P66881	2.7731065	down	NM_138554	chr9:120479236-120	TLR4
A_23_P372331	2.212803	down	NM_182916	chr3:003189717-003	TRNT1
A_24_P721898	2.116825	down	NM_001002860	chr14:093704043-09	BTBD7
A_32_P128701	2.8612628	down	NM_019050	chr4:120216331-120	USP53
A_23_P99762	3.177427	down	NM_021239	chr14:073587194-07	RBM25
A_24_P336705	2.1173606	down	NM_004582	chr1:076259803-076	RABGGTB
A_33_P3363260	2.7906893	down	NM_173582	chr11:074041421-07	PGM2L1
A_33_P3242062	2.0744497	down		chr9:125451692-125451751	
A_33_P3380797	2.276123	down	NM_005247	chr11:069624795-06	FGF3
A_23_P115608	3.0612779	down	NM_020824	chr6:080773806-080	ARHGAP21
A_33_P3386453	2.8418919	down	NM_001030055	chr7:055005612-055	ARHGAP5
A_23_P409553	2.023175	down	NM_177951	chr14:060752731-06	PPM1A
A_33_P3377911	2.2887067	down		chr7:130565591-130565532	
A_23_P418083	2.115937	down	NM_181714	chr6:080195326-080	LCA5
A_33_P3294583	2.474598	down	NR_002799	chr4:100873548-100	LOC256880
A_33_P3337599	2.2309915	down	NM_006904	chr8:048686544-048	PRKDC
A_23_P82762	2.250642	down	NM_001042416	chr8:000196946-000	ZNF596
A_33_P3315719	4.2855388	down	NM_172069	chr2:043992834-043	PLEKHH2
A_33_P3339650	4.2517639	down	NM_001723	chr6:056480374-056	DST
A_33_P3331237	2.5656333	down		chr5:151192282-151	G3BP1
A_33_P3335590	2.0535043	down	NM_001103175	chr16:003077927-00	CCDC64B
A_23_P394605	2.086704	down	NM_021982	chr5:134063223-134	SEC24A
A_33_P3223964	2.2399492	down	XM_002347452	chr14:106733285-10	LOC100290415
A_33_P3279871	2.0684777	down	XM_001726991	chr19:21838600-21838659	
A_23_P201951	2.6244121	down	NM_016374	chr1:235331255-235	ARID4B
A_33_P3298577	3.3351076	down	NM_018183	chr12:123780560-12	SBNO1
A_33_P3335624	2.0277137	down		chrX:107331777-107331718	
A_23_P32938	2.3582669	down	NM_004398	chr11:108811424-10	DDX10
A_33_P3335177	3.4527295	down	NM_003014	chr7:037945829-037	SFRP4
A_23_P126037	2.6535854	down	NM_012421	chr1:040706263-040	RLF
A_33_P3351510	2.3880499	down	NM_014271	chrX:029973954-02(	IL1RAPL1
A_32_P35512	2.2003057	down	NM_003142	chr7:012511484-012	SSB
A_33_P3736691	2.3894383	down	NM_025189	chr19:023406184-02	ZNF430
A_33_P3863935	2.658951	down	NR_028492	chr10:089605232-08	CFLP1
A_24_P406714	2.4570462	down	NM_004641	chr10:022031960-02	MLLT10
A_23_P15844	3.3476398	down	NM_032043	chr17:059760967-05	BRIP1
A_33_P3416483	2.177441	down	NR_026778	chr1:245008150-245	NCRNA00201
A_24_P169386	2.0420197	down	NM_001040710		C2orf84
A_23_P90944	2.4208579	down	NM_002976	chr2:167262271-167	SCN7A
A_33_P3264955	2.635369	down		chr12:042825332-042825273	
A_33_P3328061	3.8916897	down		chr2:225363631-225363572	
A_32_P4792	3.0947876	down	AK057820	chr9:67985769-67985828	
A_32_P130265	2.3880135	down	NM_014827	chr6:066015831-066	ZC3H11A
A_23_P210300	2.6334316	down	NM_018263	chr2:025962434-025	ASXL2
A_33_P3231047	2.8053858	down	NM_001142682	chrX:111625535-111	CCDC121
A_33_P3270109	3.0695496	down	NM_138792	chr15:052252156-05	LEO1
A_23_P64280	2.9415054	down	AL832629	chr11:124659535-12	UNQ565
A_32_P102935	2.1007558	down	NM_001008779	chr2:029063246-029	SPDYA
A_32_P68504	2.7297276	down	NM_020923	chr2:207178891-207	ZDBF2
A_23_P363936	2.2913582	down	NM_014278	chr4:128754250-128	HSPA4L

Homo sapiens nuclea	GO:0043565|GO:000	NM_005126	Hs.37288	9975	181.2206
Homo sapiens zinc fi	GO:0005622|GO:000	NM_152943	Hs.124047	10795	15.30256
Homo sapiens neurof	GO:0001656|GO:000	NM_001042492	Hs.113577	4763	36.326416
Homo sapiens media	GO:0005515|GO:000	NM_004830	Hs.29679	9439	153.12817
Homo sapiens RB1-i	GO:0007507|GO:000	NM_014781	Hs.196102	9821	230.91884
Homo sapiens Myb-l	GO:0016578|GO:000	NM_001085487	Hs.477495	114803	27.644533
Homo sapiens COBL-like 1 (COBLL1), m		NM_014900	Hs.470457	22837	95.91674
Homo sapiens ESF1,	GO:0005730|GO:000	NM_016649	Hs.369284	51575	299.5708
Homo sapiens Alport	GO:0008150|GO:000	NM_015365	Hs.656243	9949	108.273994
Homo sapiens crystal	GO:0005212|GO:000	NM_017541	Hs.376209	1427	10.13144
Homo sapiens toll-lik	GO:0005515|GO:003	NM_138554	Hs.174312	7099	251.6918
Homo sapiens tRNA	GO:0005739|GO:000	NM_182916	Hs.567495	51095	161.76425
Homo sapiens BTB (	GO:0005515	NM_001002860	Hs.525549	55727	809.7411
Homo sapiens ubiqui	GO:0008150|GO:000	NM_019050	Hs.431081	54532	67.030365
Homo sapiens RNA l	GO:0005737|GO:001	NM_021239	Hs.531106	58517	323.0422
Homo sapiens Rab g	GO:0005515|GO:000	NM_004582	Hs.78948	5876	938.56036
Homo sapiens phospl	GO:0047933|GO:000	NM_173582	Hs.26612	283209	49.237514
					15.06471
Homo sapiens fibrob	GO:0005515|GO:000	NM_005247	Hs.37092	2248	215.43454
Homo sapiens Rho G	GO:0005622|GO:000	NM_020824	Hs.524195	57584	314.834
Homo sapiens Rho G	GO:0008361|GO:003	NM_001030055	Hs.592313	394	23.8418
Homo sapiens protei	GO:0008287|GO:004	NM_177951	Hs.130036	5494	64.703735
					19.795849
Homo sapiens Leber	GO:0005929|GO:000	NM_181714	Hs.21945	167691	16.000507
Homo sapiens hypothetical LOC256880 (L		NR_002799	Hs.448554	256880	4.9999995
Homo sapiens protei	GO:0004677|GO:000	NM_006904	Hs.491682	5591	1098.5792
Homo sapiens zinc fi	GO:0005622|GO:000	NM_001042416	Hs.591388	169270	9.614673
Homo sapiens plecks	GO:0005737|GO:001	NM_172069	Hs.164162	130271	6.9086666
Homo sapiens dystor	GO:0005515|GO:003	NM_001723	Hs.604915	667	29.121778
Ras GTPase-activati	GO:0005515|GO:0005886|GO:0003723|GO:0004386|GO:0007			10146	102.11222
Homo sapiens coiled-coil domain contaiin		NM_001103175	Hs.513285	146439	119.35531
Homo sapiens SEC2	GO:0005515|GO:004	NM_021982	Hs.595540	10802	1123.5281
Putative uncharacterized protein ENSP000		XM_002347452		100290415	10.994901
Putative uncharacterized protein ENSP000		XM_001726991	Hs.568075		49.90374
Homo sapiens AT ric	GO:0005622|GO:000	NM_016374	Hs.575782	51742	203.0264
Homo sapiens strawb	GO:0008150|GO:000	NM_018183	Hs.577403	55206	118.61977
26S proteasome non-ATPase regulatory subunit 10 (26S proteasome regulatory subunit p28)(Gankyrin) [So					3401.444
Homo sapiens DEAD	GO:0008026|GO:000	NM_004398	Hs.591931	1662	919.8604
Homo sapiens secret	GO:0005515|GO:000	NM_003014	Hs.658169	6424	4.9999995
Homo sapiens rearra	GO:0005622|GO:000	NM_012421	Hs.205627	6018	187.49016
Homo sapiens interle	GO:0007165|GO:001	NM_014271	Hs.658912	11141	424.31686
Homo sapiens Sjogre	GO:0005515|GO:000	NM_003142	Hs.632535	6741	3195.2627
Homo sapiens zinc fi	GO:0005622|GO:000	NM_025189	Hs.466289	80264	169.05704
Homo sapiens cofilin pseudogene 1 (CFLF		NR_028492	Hs.646123	142913	4.9999995
Homo sapiens myelo	GO:0005515|GO:000	NM_004641	Hs.30385	8028	235.61525
Homo sapiens BRCA	GO:0005515|GO:000	NM_032043	Hs.532799	83990	154.38171
Homo sapiens non-protein coding RNA 20		NR_026778	Hs.411490	284702	12.726639
Homo sapiens chromosome 2 open reading		NM_001040710	Hs.467868	653140	4.9999995
Homo sapiens sodiur	GO:0006936|GO:000	NM_002976	Hs.635368	6332	4.9999995
					963.47516
					13.96981
Novel protein Fragment [Source:UniProtKB/TrEMBL;Acc:Q4			Hs.722109		5.740408
Homo sapiens zinc fi	GO:0005515|GO:000	NM_014827	Hs.532399	9877	675.7924
Homo sapiens additi	GO:0008270|GO:000	NM_018263	Hs.119815	55252	2223.389
Homo sapiens coiled-coil domain contaiin		NM_001142682	Hs.21081	79635	4.9999995
Homo sapiens Leo1,	GO:0005515|GO:003	NM_138792	Hs.567662	123169	327.26355
Homo sapiens mRNA; cDNA DKFZp451E1418 (from clone DK			Hs.661469	100130428	18.50205
Homo sapiens speedy	GO:0008284|GO:000	NM_001008779	Hs.511956	245711	16.673979
Homo sapiens zinc fi	GO:0008270|GO:000	NM_020923	Hs.110489	57683	30.606825
Homo sapiens heat sl	GO:0006986|GO:000	NM_014278	Hs.135554	22824	419.45905

446.53058	7.6467624	8.650993
54.02855	4.0660224	5.629567
124.45047	5.3190413	6.8315506
427.34482	7.390686	8.583227
887.86066	8.006908	9.652559
81.270386	4.931095	6.2155275
441.85724	6.716997	8.632286
1478.592	8.385782	10.420585
282.66736	6.888864	7.9863434
33.149075	3.4947221	4.908616
861.94244	8.133299	9.604802
437.21286	7.471778	8.617653
2034.7662	9.80356	10.885462
231.99458	6.196252	7.712904
1243.6838	8.491356	10.159215
2309.3591	9.998751	11.081018
163.817	5.739816	7.2204375
37.623917	4.0420227	5.0947514
605.37646	7.9073715	9.09395
1165.5701	8.453224	10.067358
81.568886	4.7145047	6.2213564
156.64653	6.1429744	7.1595955
54.326786	4.441695	5.6362276
40.723827	4.134248	5.2155447
13.81476	2.3362613	3.6434555
2829.1692	10.219627	11.377312
26.488802	3.4241114	4.594448
36.781628	2.962722	5.0621986
149.92206	5.004835	7.0928965
320.12924	6.806952	8.166267
298.5158	7.028244	8.066332
2709.2144	10.249672	11.310898
30.063364	3.607883	4.771349
123.02939	5.7609196	6.809489
653.3401	7.8170757	9.20907
480.54233	7.0186467	8.75638
7356.5825	11.793727	12.813581
2523.61	9.96859	11.206317
19.140762	2.3362613	4.1239986
608.8535	7.694096	9.102039
1218.824	8.875039	10.130872
7531.175	11.708232	12.845936
494.26978	7.5390725	8.795744
14.784111	2.3362613	3.7471185
708.0791	8.034343	9.331268
626.2203	7.4027357	9.14588
33.740955	3.8119664	4.9346
11.530541	2.3362613	3.3662581
13.56596	2.3362613	3.6117797
2923.4482	10.035303	11.433308
65.016594	3.940486	5.9008827
22.227175	2.712924	4.3427644
1907.3528	9.539532	10.795343
6390.779	11.214388	12.611332
15.626077	2.3362613	3.8244605
1217.5614	8.510601	10.128628
64.86467	4.341322	5.8978767
42.25757	4.194521	5.2654295
101.16991	5.075845	6.524602
1156.198	8.858191	10.054394

A_33_P3218905	3.2646022	down	NR_002806	chr4:089651092-089	FAM13AOS
A_33_P3264846	3.0240578	down	NM_152703	chr7:092760649-092	SAMD9L
A_33_P3372413	2.7912493	down	XM_002347518	chr15:030297892-03	LOC390557
A_23_P29994	2.6429314	down	NM_203284	chr4:026432686-026	RBPJ
A_32_P27706	2.1309238	down	NM_003659	chr2:178408390-178	AGPS
A_24_P97145	3.0950485	down	NM_020738	chr2:008869593-008	KIDINS220
A_32_P219116	3.329358	down	NM_018451	chr13:025457465-02	CENPJ
A_33_P3396527	2.0000991	down	NM_006467	chr5:089810118-089	POLR3G
A_23_P215318	2.5675967	down	NM_014396	chr7:038763832-038	VPS41
A_24_P791040	3.0732857	down	NM_012164	chr9:123519894-123	FBXW2
A_23_P50108	2.3490395	down	NM_006101	chr18:002610798-00	NDC80
A_23_P163408	2.56448	down	NM_020843	chr15:076640909-07	SCAPER
A_24_P19268	2.6948893	down	NM_182515	chr19:021477040-02	ZNF714
A_24_P58122	3.1208673	down	NM_015306	chr1:055532583-055	USP24
A_23_P209689	4.3274564	down	NM_004850	chr2:011322103-011	ROCK2
A_23_P385126	2.8281756	down	NM_139160	chr11:033054824-03	DEPDC7
A_33_P3413098	2.1191181	down	NR_024618	chr3:122611203-122	LOC100129550
A_33_P3356631	2.2384395	down	NM_006298	chr6:028125177-028	ZNF192
A_32_P223777	3.140112	down		chr5:55231193-5523	IL6ST
A_23_P81463	2.3324643	down	NM_019030	chr5:054557327-054	DHX29
A_33_P3216890	2.1093048	down	NM_018440	chr8:081882726-081	PAG1
A_24_P194886	2.6335275	down	NM_015252	chr2:063091865-063	EHBP1
A_33_P3334121	2.4973344	down		chr1:148339922-148339863	
A_33_P3331856	2.934881	down	AK126467	chr7:31823215-3182	PDE1C
A_23_P335039	3.5311289	down	NM_133474	chr4:000433966-000	ZNF721
A_33_P3231953	3.1832044	down	NM_004370	chr6:075822987-075	COL12A1
A_32_P1701	2.6675383	down	NM_016937	chrX:025014903-025	POLA1
A_24_P916614	2.3406058	down	NM_005156	chr9:114981200-114	ROD1
A_33_P3253070	2.9522742	down	NM_015979	chr6:131919535-131	MED23
A_33_P3337182	2.5797694	down	XM_001716715	chr2:118595714-118	LOC389024
A_33_P3223759	3.6551763	down	NM_005121	chr17:060020094-06	MED13
A_33_P3345831	3.0063584	down	NM_001010874	chr4:065144430-065	TECRL
A_32_P159334	2.9060127	down	NM_002907	chr11:106696853-10	RECQL
A_23_P134384	3.0103704	down	NM_014660	chr7:011142499-011	PHF14
A_33_P3210583	3.4189248	down	NM_014911	chr2:069702987-069	AAK1
A_33_P3394395	2.1523048	down	NM_198549	chr1:078343754-078	FAM73A
A_23_P25215	3.4452221	down	NM_032941		RECQL
A_33_P3227666	2.010218	down	NM_018046	chr5:076360866-076	AGGF1
A_24_P390583	2.6015251	down	NM_020718	chr16:023073163-02	USP31
A_33_P3227506	4.1256788	down	NM_182641	chr17:065980372-06	BPTF
A_23_P76159	4.5480014	down	NM_003566	chr12:093166521-09	EEA1
A_33_P3240702	2.4051352	down	NM_002894	chr18:020606172-02	RBBP8
A_23_P6651	2.6558663	down	NM_015224	chr3:056654650-056	C3orf63
A_33_P3358641	3.4936997	down	NM_001137552	chr2:238672559-238	LRRFIP1
A_23_P19702	2.1138676	down	NM_015093	chr6:149732565-149	MAP3K7IP2
A_32_P524614	2.0512012	down	NM_175734	chr17:007330813-00	C17orf74
A_23_P148584	2.9129495	down	NM_144658	chrX:117819952-117	DOCK11
A_23_P157527	4.2304341	down	NM_033402	chr8:086057728-086	LRRCC1
A_24_P944299	3.059585	down	NM_003607	chr1:227178114-227	CDC42BPA
A_23_P7282	2.1654142	down	BX648349	chr4:141474758-141	ELMOD2
A_24_P717262	3.4530468	down	XM_001715121	chr19:023779543-02	LOC100131479
A_24_P180242	2.0525263	down	AB040915	chr4:27025887-27025946	
A_24_P106112	2.2837134	down	NM_000297	chr4:088998599-088	PKD2
A_23_P404108	2.8172924	down	NM_018999	chr10:086277812-08	FAM190B
A_33_P3530314	4.2326559	down	NM_023073	chr5:037107443-037	C5orf42
A_33_P3234989	2.5186716	down	NM_014055	chr12:110656447-11	IFT81
A_23_P218827	3.3015534	down	NM_199420	chr3:121150761-121	POLQ
A_24_P409824	2.5587443	down		chr9:101370186-101370247	
A_24_P371303	2.5300186	down	NM_015224	chr3:056657192-056	C3orf63
A_23_P16006	2.5980334	down	NM_198457	chr19:053269328-05	ZNF600

Homo sapiens FAM13A opposite strand (n		NR_002806	Hs.703446	285512	7.7309165
Homo sapiens sterile alpha motif domain c		NM_152703	Hs.489118	219285	11.821491
PREDICTED: Homo sapiens similar to hC		XM_002347518		390557	171.05318
Homo sapiens recom	GO:0005515|GO:000	NM_203284	Hs.479396	3516	3024.0662
Homo sapiens alkylg	GO:0005739|GO:000	NM_003659	Hs.516543	8540	378.31824
Homo sapiens kinase	GO:0007242|GO:001	NM_020738	Hs.9873	57498	342.57626
Homo sapiens centro	GO:0046785|GO:000	NM_018451	Hs.513379	55835	466.84177
Homo sapiens polym	GO:0032728|GO:004	NM_006467	Hs.282387	10622	984.0936
Homo sapiens vacuol	GO:0008017|GO:000	NM_014396	Hs.592184	27072	59.95953
Homo sapiens F-box	GO:0005515|GO:000	NM_012164	Hs.494985	26190	4.9999995
Homo sapiens NDC8	GO:0005515|GO:000	NM_006101	Hs.414407	10403	4613.882
Homo sapiens S-phas	GO:0005622|GO:000	NM_020843	Hs.458986	49855	64.44355
Homo sapiens zinc fi	GO:0005622|GO:000	NM_182515	Hs.466291	148206	3278.581
Homo sapiens ubiqui	GO:0006511|GO:000	NM_015306	Hs.477009	23358	116.38221
Homo sapiens Rho-a	GO:0005515|GO:000	NM_004850	Hs.591600	9475	197.82799
Homo sapiens DEP d	GO:0008150|GO:000	NM_139160	Hs.280990	91614	4.9999995
Homo sapiens hypothetical LOC10012955		NR_024618	Hs.61558	100129550	453.61905
Homo sapiens zinc fi	GO:0005622|GO:000	NM_006298	Hs.57679	7745	14.258582
Interleukin-6 recepto	GO:0005138|GO:0004921|GO:0008284|GO:0005886|GO:0004			3572	35.349094
Homo sapiens DEAH	GO:0008026|GO:000	NM_019030	Hs.593268	54505	826.2849
Homo sapiens phospl	GO:0005515|GO:000	NM_018440	Hs.266175	55824	28.920261
Homo sapiens EH do	GO:0005515|GO:000	NM_015252	Hs.271667	23301	480.65195
					58.984
Calcium/calmodulin-	GO:0007165|GO:0005516|GO:0016787|G		Hs.290550	5137	4.9999995
Homo sapiens zinc fi	GO:0005622|GO:000	NM_133474	Hs.428360	170960	136.38159
Homo sapiens collag	GO:0005515|GO:000	NM_004370	Hs.101302	1303	25.694544
Homo sapiens polym	GO:0005515|GO:000	NM_016937	Hs.567319	5422	891.29956
Homo sapiens ROD1	GO:0006397|GO:000	NM_005156	Hs.269988	9991	716.0447
Homo sapiens media	GO:0005515|GO:000	NM_015979	Hs.29679	9439	14.750002
PREDICTED: Homo sapiens similar to hC		XM_001716715	Hs.528527	389024	4.9999995
Homo sapiens media	GO:0046966|GO:003	NM_005121	Hs.282678	9969	376.46445
Homo sapiens trans-2	GO:0006629|GO:000	NM_001010874	Hs.227752	253017	16.0271
Homo sapiens RecQ	GO:0005515|GO:000	NM_002907	Hs.235069	5965	41.352676
Homo sapiens PHD f	GO:0005515|GO:000	NM_014660	Hs.655688	9678	55.244003
Homo sapiens AP2 a	GO:0005905|GO:000	NM_014911	Hs.468878	22848	4.9999995
Homo sapiens family	GO:0016020|GO:001	NM_198549	Hs.632419	374986	189.4768
Homo sapiens RecQ	GO:0005515|GO:000	NM_032941	Hs.235069	5965	553.1853
Homo sapiens angiog	GO:0005515|GO:000	NM_018046	Hs.634849	55109	639.82275
Homo sapiens ubiqui	GO:0006511|GO:000	NM_020718	Hs.183817	57478	53.847404
Homo sapiens bromo	GO:0005634|GO:000	NM_182641	Hs.444200	2186	20.65459
Homo sapiens early e	GO:0005516|GO:000	NM_003566	Hs.567367	8411	217.55162
Homo sapiens retinol	GO:0005515|GO:000	NM_002894	Hs.546282	5932	1640.0602
Homo sapiens chromosome 3 open reading		NM_015224	Hs.116877	23272	137.04568
Homo sapiens leucin	GO:0016564|GO:000	NM_001137552	Hs.471779	9208	78.64022
Homo sapiens mitoge	GO:0005622|GO:000	NM_015093	Hs.269775	23118	1744.1111
Homo sapiens chrom	GO:0016020|GO:001	NM_175734	Hs.380704	201243	341.96967
Homo sapiens dedica	GO:0005085|GO:005	NM_144658	Hs.368203	139818	91.5823
Homo sapiens leucin	GO:0005515|GO:000	NM_033402	Hs.193115	85444	15.63069
Homo sapiens CDC4	GO:0007242|GO:000	NM_003607	Hs.35433	8476	98.877975
ELMO domain-conta	GO:0006909|GO:0005856		Hs.450105	255520	583.5564
PREDICTED: Homo sapiens similar to hC		XM_001715121		100131479	13.092468
Stromal interaction molecule 2 Precursor [Source:UniProtKB/S			Hs.135763		17.048775
Homo sapiens polycy	GO:0008092|GO:000	NM_000297	Hs.181272	5311	206.84834
Homo sapiens family with sequence simila		NM_018999	Hs.461988	54462	170.61671
Homo sapiens chrom	GO:0016020|GO:001	NM_023073	Hs.586199	65250	216.74327
Homo sapiens intrafl	GO:0007275|GO:000	NM_014055	Hs.528382	28981	298.7292
Homo sapiens polym	GO:0006260|GO:000	NM_199420	Hs.241517	10721	144.37741
					602.05133
Homo sapiens chromosome 3 open reading		NM_015224	Hs.116877	23272	388.40207
Homo sapiens zinc fi	GO:0005622|GO:000	NM_198457	Hs.696848	162966	273.37454

31.353142	3.1257668	4.832674
43.42124	3.7066836	5.3031693
581.85156	7.554737	9.035648
8612.982	11.641566	13.043705
990.18896	8.719837	9.811316
1282.8308	8.577702	10.207664
1848.8815	9.014078	10.749322
2280.768	10.063468	11.0635395
183.72113	6.028033	7.3884516
17.0179	2.3362613	3.9560432
11272.924	12.220959	13.45303
198.04375	6.1363277	7.494994
9348.402	11.741824	13.17205
442.67993	6.993946	8.635893
1042.1049	7.7746577	9.888177
15.748469	2.3362613	3.836133
1160.6931	8.975261	10.058725
38.393387	3.9644637	5.126957
133.1103	5.277637	6.928453
2259.171	9.828808	11.050663
73.32221	4.996074	6.0728416
1517.0128	9.054389	10.4513855
175.67007	6.005533	7.325922
16.35618	2.3362613	3.8895633
585.7342	7.2264805	9.04661
99.1611	4.8226757	6.4931555
2770.774	9.929391	11.3449
1987.662	9.627827	10.854709
51.986897	4.014781	5.5766077
14.386549	2.3362613	3.7035034
1652.4606	8.709288	10.579229
57.51053	4.1374874	5.7255044
143.17615	5.4945617	7.0336027
199.0468	5.9100685	7.5000095
18.959747	2.3362613	4.109804
500.48703	7.7110276	8.81691
2239.387	9.25033	11.034927
1537.581	9.464504	10.471856
167.46887	5.874788	7.2541456
102.69702	4.5004625	6.545094
1196.0524	7.9185333	10.103766
4437.7305	10.779922	12.04604
444.74982	7.2325325	8.641715
336.3939	6.4348354	8.239591
4146.576	10.87175	11.951635
865.8634	8.574987	9.611456
325.77554	6.6516933	8.194174
79.33458	4.101462	6.1822677
370.08493	6.759678	8.373014
1503.1199	9.325003	10.439646
54.3499	3.8494697	5.6373396
42.2661	4.228632	5.2660327
583.01276	7.8484454	9.039827
585.60876	7.5515347	9.045844
1113.1968	7.9150248	9.996588
929.66376	8.382587	9.71525
579.98145	7.306467	9.029612
1822.47	9.373812	10.729248
1189.2643	8.754367	10.093515
874.19336	8.250557	9.627977

A_23_P383601	3.0851754	down	NR_029434	chr14:058732910-05	FLJ31306
A_23_P61674	2.6428873	down	NM_020666	chr5:178030053-178	CLK4
A_33_P3636080	2.3842301	down	NM_001040108	chr14:075485674-07	MLH3
A_33_P3248787	2.3226238	down	XM_001133555	chr1:149263522-149	LOC729130
A_23_P144684	2.2302045	down	NM_032290	chr5:094031167-094	ANKRD32
A_33_P3312735	3.6499525	down	NM_024989	chr2:197697837-197	PGAP1
A_33_P3234855	4.5492206	down	NM_007124	chr6:145148752-145	UTRN
A_23_P160025	2.2912216	down	NM_005531	chr1:159024619-159	IFI16
A_23_P69826	2.2629238	down	NM_001358	chr4:024531344-024	DHX15
A_24_P95029	2.2021718	down	NM_006024		TAX1BP1
A_33_P3222703	2.0893746	down	NM_001024855	chr3:044688869-044	ZNF197
A_24_P153511	3.4474908	down	NM_020841	chr12:076746619-07	OSBPL8
A_33_P3231140	2.2378448	down	NM_020337	chr4:125585620-125	ANKRD50
A_23_P68547	3.2400498	down	NM_182802	chr20:005975064-00	MCM8
A_33_P3266315	2.1314039	down	NM_016009	chr1:087213745-087	SH3GLB1
A_24_P419276	2.0770957	down	NM_021045	chr10:038120137-03	ZNF248
A_23_P312536	2.51255	down	NM_198150	chr5:094939895-094	ARSK
A_32_P179746	2.9736946	down		chr5:094050473-094050532	
A_33_P3245126	2.6245287	down		chr6:18399877-18399936	
A_24_P237757	2.9266759	down	AL136621	chr13:20593755-20593814	
A_33_P3233843	2.3903701	down	NM_175767	chr5:055265550-055	IL6ST
A_33_P3287815	2.7038554	down	NM_004728	chr10:070744186-07	DDX21
A_32_P71113	2.9356549	down	NM_173690	chr9:127705207-127	SCAI
A_23_P200772	3.2830447	down	NM_201269	chr1:091403539-091	ZNF644
A_33_P3294459	8.8132596	down	NR_024086		COPG2IT1
A_23_P356694	4.0533546	down	NM_153324	chr20:030037996-03	DEFB123
A_23_P327361	5.9313788	down	NM_015263	chr15:051741102-05	DMXL2
A_32_P61684	2.3007002	down	NM_018440	chr8:081880292-081	PAG1
A_24_P365025	2.0379025	down	NM_003971	chr17:049043155-04	SPAG9
A_23_P257503	2.5943683	down	NM_003922	chr15:063901042-06	HERC1
A_23_P70047	2.1471128	down	NM_199189	chr5:138665300-138	MATR3
A_24_P157156	3.1873585	down	NM_001080539	chr2:197590735-197	CCDC150
A_23_P97123	2.0114892	down	NR_026844	chr1:168215109-168	ANKRD36BL1
A_33_P3243394	2.1746234	down	AK124200	chr3:57883097-57883156	
A_24_P943957	2.4573756	down	NM_015040	chr2:209222843-209	PIKFYVE
A_33_P3641427	2.3881757	down	NM_016132	chr15:048434932-04	MYEF2
A_23_P381577	2.5169333	down	NM_145011	chr10:038239068-03	ZNF25
A_33_P3378051	2.5335449	down	NM_178123	chr2:179966502-179	SESTD1
A_23_P25003	2.7816897	down	NM_002956	chr12:122756161-12	CLIP1
A_23_P95736	2.5117363	down	NM_001001415	chr19:021720226-02	ZNF429
A_33_P3314902	3.9330069	down	NM_016252	chr2:032843885-032	BIRC6
A_23_P309261	4.4580939	down	NM_005751	chr7:091739783-091	AKAP9
A_32_P145051	2.2036454	down	XM_002342513	chr5:069193892-069	LOC100133280
A_33_P3258041	2.4156806	down	NM_004837	chr1:235507616-235	GGPS1
A_33_P3404671	2.325514	down	NR_024567	chr1:041154811-041	LOC100130557
A_23_P18493	2.4906869	down	NM_080685	chr4:087735935-087	PTPN13
A_33_P3248774	2.4381465	down		chr1:245027582-245027641	
A_24_P927325	2.0282989	down	NM_015531	chr11:073745605-07	C2CD3
A_23_P314191	2.4013106	down	NM_015336	chr12:077247154-07	ZDHHC17
A_24_P944131	3.2788102	down	NM_016540	chr11:094111566-09	GPR83
A_32_P316136	2.6243656	down	NM_194250	chr2:185803216-185	ZNF804A
A_33_P3276913	2.1958797	down	BG533310	chr21:38471329-38471388	
A_33_P3347241	2.5138508	down	XR_041271	chr21:15287621-15287562	
A_33_P3335840	3.9260512	down	NM_001006623	chr2:128493030-128	WDR33
A_33_P3303940	2.4192363	down	NM_001018055	chrX:154350943-15⁄	BRCC3
A_23_P52058	2.0398527	down	NM_005646	chr1:234528232-234	TARBP1
A_33_P3290296	2.1808144	down	AK125829	chr10:112663496-11	LOC100130175
A_33_P3314146	2.4334896	down	NM_001042533	chr3:097660805-097	MINA
A_23_P92614	2.5501233	down	NM_133636		HELQ
A_23_P257911	3.1131498	down	NM_001032410	chr21:030426744-03	USP16

Homo sapiens hypothetical LOC379025 (F		NR_029434	Hs.531089	379025	186.65791
Homo sapiens CDC-l	GO:0046777|GO:000	NM_020666	Hs.406557	57396	90.10117
Homo sapiens mutL l	GO:0005515|GO:000	NM_001040108	Hs.436650	27030	42.352245
PREDICTED: Homo sapiens hypothetical		XM_001133555	Hs.692489	729130	6.582093
Homo sapiens ankyri	GO:0005622	NM_032290	Hs.657315	84250	571.89594
Homo sapiens post-C	GO:0042578|GO:000	NM_024989	Hs.229988	80055	23.984217
Homo sapiens utroph	GO:0005515|GO:003	NM_007124	Hs.133135	7402	14.196861
Homo sapiens interfe	GO:0005515|GO:001	NM_005531	Hs.380250	3428	969.00195
Homo sapiens DEAI	GO:0008380|GO:000	NM_001358	Hs.696074	1665	5013.151
Homo sapiens Tax1 (	GO:0005622|GO:000	NM_006024	Hs.34576	8887	2049.1375
Homo sapiens zinc fi	GO:0005622|GO:000	NM_001024855	Hs.157035	10168	75.14882
Homo sapiens oxyste	GO:0006869|GO:000	NM_020841	Hs.430849	114882	15.40106
Homo sapiens ankyrin repeat domain 50 (A		NM_020337	Hs.480694	57182	79.01064
Homo sapiens minich	GO:0006260|GO:001	NM_182802	Hs.597484	84515	507.5398
Homo sapiens SH3-d	GO:0005739|GO:000	NM_016009	Hs.136309	51100	2286.9048
Homo sapiens zinc fi	GO:0005622|GO:000	NM_021045	Hs.528423	57209	22.101591
Homo sapiens arylsu	GO:0016787|GO:000	NM_198150	Hs.585051	153642	22.30795
					57.218014
E3 ubiquitin-protein ligase RNF144B (EC 6.3.2.-)(RING finger protein 144B)(IBR domain-containing prot					11.908108
Zinc finger MYM-type protein 2 (Zinc finger protein 198)(Fusec			Hs.644041		115.98411
Homo sapiens interle	GO:0005138|GO:000	NM_175767	Hs.532082	3572	81.64373
Homo sapiens DEAD	GO:0005515|GO:000	NM_004728	Hs.223141	9188	1623.0564
Homo sapiens suppre	GO:0005515|GO:003	NM_173690	Hs.59504	286205	55.419296
Homo sapiens zinc fi	GO:0005622|GO:000	NM_201269	Hs.173001	84146	206.11089
Homo sapiens COPG2 imprinted transcript		NR_024086	Hs.715567	53844	4.9999995
Homo sapiens defens	GO:0005576|GO:004	NM_153324	Hs.122509	245936	15.40554
Homo sapiens Dmx-l	GO:0017137|GO:003	NM_015263	Hs.511386	23312	4.9999995
Homo sapiens phospl	GO:0005515|GO:000	NM_018440	Hs.266175	55824	94.43391
Homo sapiens sperm	GO:0005515|GO:004	NM_003971	Hs.463439	9043	1805.0337
Homo sapiens hect (l	GO:0005622|GO:000	NM_003922	Hs.210385	8925	162.10191
Homo sapiens matrin	GO:0005622|GO:000	NM_199189	Hs.268939	9782	3900.1416
Homo sapiens coiled-coil domain containi		NM_001080539	Hs.132519	284992	32.329346
Homo sapiens ankyrin repeat domain 36B-		NR_026844	Hs.537067	84832	1280.418
Sarcolemmal membrane-associated protein (Sarcolemmal-associ			Hs.476432		33.71779
Homo sapiens phospl	GO:0005515|GO:000	NM_015040	Hs.720192	200576	147.1214
Homo sapiens myelir	GO:0005794|GO:000	NM_016132	Hs.6638	50804	235.41885
Homo sapiens zinc fi	GO:0005622|GO:000	NM_145011	Hs.499429	219749	33.96149
Homo sapiens SEC14	GO:0005515	NM_178123	Hs.30977	91404	4.9999995
Homo sapiens CAP-(	GO:0008017|GO:000	NM_002956	Hs.524809	6249	1022.3747
Homo sapiens zinc fi	GO:0005622|GO:000	NM_001001415	Hs.709598	353088	1601.1553
Homo sapiens baculc	GO:0005515|GO:005	NM_016252	Hs.150107	57448	180.58812
Homo sapiens A kina	GO:0007165|GO:000	NM_005751	Hs.651221	10142	148.4979
PREDICTED: Homo sapiens similar to Pu		XM_002342513	Hs.711155	100133280	51.92619
Homo sapiens gerany	GO:0005737|GO:000	NM_004837	Hs.647791	9453	4.9999995
Homo sapiens hypothetical LOC10013055		NR_024567		100130557	43.844913
Homo sapiens protei	GO:0005515|GO:000	NM_080685	Hs.436142	5783	97.46533
					8.106961
Homo sapiens C2 cal	GO:0030162|GO:002	NM_015531	Hs.694798	26005	33.28533
Homo sapiens zinc fi	GO:0005515|GO:001	NM_015336	Hs.4014	23390	791.7842
Homo sapiens G prot	GO:0007165|GO:000	NM_016540	Hs.272385	10888	4.9999995
Homo sapiens zinc fi	GO:0005622|GO:000	NM_194250	Hs.159528	91752	4.9999995
Tetratricopeptide repeat protein 3 (TPR repeat protein 3)(TPR re			Hs.659005		43.27293
chromosome 21 open reading frame 81 (C2		XR_041271	Hs.648245		4.9999995
Homo sapiens WD re	GO:0005515|GO:000	NM_001006623	Hs.620490	55339	30.301907
Homo sapiens BRCA	GO:0005515|GO:000	NM_001018055	Hs.558537	79184	203.08057
Homo sapiens TAR (	GO:0006396|GO:000	NM_005646	Hs.498115	6894	376.45822
Homo sapiens cDNA FLJ43841 fis, clone TESTI4006137. [AK			Hs.232543	100130175	198.67006
Homo sapiens MYC	GO:0005737|GO:004	NM_001042533	Hs.607776	84864	124.16676
Homo sapiens helicas	GO:0008026|GO:001	NM_133636	Hs.480101	113510	35.201874
Homo sapiens ubiqui	GO:0043130|GO:000	NM_001032410	Hs.99819	10600	882.3305

699.437	7.6875615	9.312914
291.60526	6.6312866	8.0334015
120.62888	5.5297365	6.78326
19.011366	2.8968225	4.112578
1517.9166	9.29513	10.452306
106.05471	4.7234173	6.591295
77.29561	3.9590683	6.1446877
2571.3171	10.042683	11.2388
11835.58	12.341965	13.520153
4975.222	11.093152	12.232079
189.21521	6.3673544	7.4304256
63.460426	4.0789533	5.8645
214.14377	6.442328	7.604438
1947.8085	9.131691	10.827707
5369.5977	11.248816	12.34062
54.89989	4.59929	5.6538577
67.109505	4.615052	5.9442043
204.13516	5.962082	7.5343385
37.892708	3.7159252	5.1079836
414.30533	6.988143	8.537406
237.98067	6.49063	7.747864
4872.39	10.765505	12.200523
194.07777	5.913762	7.4674444
829.7425	7.8417206	9.556755
48.674892	2.3362613	5.475937
74.593315	4.079217	6.0983334
33.06379	2.3362613	4.9046288
265.28482	6.694056	7.896129
4114.9536	10.914856	11.941941
511.43307	7.4757347	8.851118
8863.775	11.992306	13.094704
123.79948	5.1502657	6.822627
2941.9097	10.435097	11.443361
88.00739	5.207161	6.3279266
440.5987	7.3300486	8.627167
687.7195	8.033528	9.289437
103.09039	5.2196884	6.5513554
14.135102	2.3362613	3.6774187
3267.996	10.118231	11.5941925
4515.932	10.747382	12.076067
867.1092	7.6380253	9.613658
799.8307	7.344322	9.500749
136.39304	5.8210664	6.9609585
13.529109	2.3362613	3.608691
121.72222	5.5777044	6.795254
296.27255	6.7392483	8.055792
24.380785	3.1871967	4.4729815
81.001526	5.1897507	6.210021
2241.8916	9.77323	11.037052
18.11151	2.3362613	4.0494337
14.613837	2.3362613	3.72823
113.5659	5.559886	6.694685
14.02204	2.3362613	3.6661603
143.18547	5.060686	7.033765
604.9792	7.8181973	9.092749
943.1571	8.709015	9.73748
532.10486	7.782243	8.90711
370.33063	7.0905714	8.373598
108.24712	5.270836	6.621403
3188.374	9.917146	11.555521

A_23_P131383	2.0869847	down	NM_018062	chr2:058386536-058	FANCL
A_23_P406702	2.9440272	down	NM_203459	chr1:200829250-200	CAMSAP1L1
A_33_P3387771	3.1312882	down	NM_001039590	chrX:041095755-041	USP9X
A_23_P27636	2.6293465	down	NM_144566	chr19:012060977-01	ZNF700
A_24_P167496	2.7416469	down	NM_031292	chr12:044122939-04	PUS7L
A_24_P177568	2.5551785	down	NM_133473	chr19:021367846-02	ZNF431
A_24_P231057	5.5475163	down	NM_148894		BOD1L
A_33_P3306510	2.1002514	down	NM_005124	chr6:017615465-017	NUP153
A_33_P3387524	2.1937063	down	NM_001114120	chr1:068941036-068	DEPDC1
A_33_P3214849	2.2174138	down	NM_153705	chr11:108343124-10	KDELC2
A_32_P87568	2.5651159	down	NM_001008493	chr1:225675153-225	ENAH
A_33_P3234060	2.0785197	down	NM_207366	chr7:055861296-055	SEPT14
A_24_P333716	2.4856876	down	NM_022459	chr13:021353576-02	XPO4
A_33_P3272390	5.1988578	down	NM_006267	chr2:109402125-109	RANBP2
A_32_P96719	2.1746979	down	NM_024745	chr16:046615134-04	SHCBP1
A_24_P231104	2.1799576	down	NM_001003679	chr1:066100649-066	LEPR
A_23_P254573	3.3135806	down	NM_014377		DNAJC2
A_33_P3464855	4.612444	down	AL050097	chr3:196858864-196	DKFZP586B0319
A_24_P56317	3.5632211	down	NM_144778	chr13:098046226-09	MBNL2
A_33_P3309621	2.0066027	down	NR_002804	chr19:051675776-05	SIGLECP3
A_23_P144384	2.3437148	down	NM_017423	chr4:174244878-174	GALNT7
A_33_P3257558	3.2259923	down		chr9:123923724-123923783	
A_24_P706752	2.05198	down	NM_030821	chr4:110631436-110	PLA2G12A
A_33_P3233841	2.7994608	down	NM_002184	chr5:055238572-055	IL6ST
A_24_P791829	2.4815353	down	NM_020772	chr17:027583112-02	NUFIP2
A_33_P3273267	2.8962286	down	XM_002343229	chr2:132861494-132861553	
A_24_P11900	2.124377	down	NM_014981	chr3:108099821-108	MYH15
A_24_P56252	2.3212099	down	NM_002480	chr12:080167752-08	PPP1R12A
A_32_P95823	2.1466456	down	NM_014607	chr2:136541520-136	UBXN4
A_24_P278460	2.6159832	down	NM_032228	chr11:013753079-01	FAR1
A_33_P3251430	2.7836868	down	NM_003489	chr21:016337033-01	NRIP1
A_24_P943106	2.4328957	down	NM_001080415	chr3:142778660-142	SR140
A_33_P3309607	2.3763989	down		chr2:201434714-201434773	
A_33_P3291569	3.332103	down	NM_016424	chr17:048829915-04	LUC7L3
A_23_P167194	4.0775815	down	NM_001812	chr12:089896162-08	CENPC1
A_24_P114255	2.4328797	down	NM_138799	chr2:008997233-008	MBOAT2
A_32_P190049	2.8883008	down	NM_001099678	chr3:120044380-120	LRRC58
A_33_P3320994	2.1402822	down		chr1:85647224-85647165	
A_32_P74964	2.8751316	down	NM_032511	chr6:099721027-099	C6orf168
A_24_P29401	3.3881147	down	NM_181523	chr5:067596786-067	PIK3R1
A_23_P354894	2.23267	down	NM_152603	chr19:037211954-03	ZNF567
A_24_P924816	2.1455464	down	NM_052885	chr12:040149281-04	SLC2A13
A_24_P941759	2.7299446	down	NM_017769	chr14:031088739-03	G2E3
A_23_P357365	2.3994986	down	NM_005862		STAG1
A_32_P149298	3.5194512	down	NM_001129993	chr2:061350543-061	KIAA1841
A_24_P89512	4.0053047	down	NM_014739	chr6:136579829-136	BCLAF1
A_24_P226116	2.3771496	down	NM_057175	chr4:140311796-140	NARG1
A_33_P3212072	2.434702	down	NM_001146039	chr1:170514832-170	GORAB
A_23_P203665	2.189079	down	NM_018367	chr11:076732830-07	ACER3
A_33_P3341836	2.426951	down	NM_001005851	chr19:040540323-04	ZNF780B
A_33_P3257140	4.9764467	down	NM_004850	chr2:011323535-011	ROCK2
A_33_P3418294	3.4767733	down	NM_001373	chr1:225284873-225	DNAH14
A_23_P148916	2.1761153	down	NM_148909	chr1:052253537-052	OSBPL9
A_24_P350576	2.4739439	down	NM_015028	chr3:170780412-170	TNIK
A_33_P3854030	2.1384226	down	NM_018328	chr2:149267644-149	MBD5
A_24_P18146	2.5598964	down	NM_015310	chr8:018388829-018	PSD3
A_24_P8454	2.0117801	down	NM_001130528		SPAG9
A_24_P753161	3.6299357	down	NM_001204	chr2:203431522-203	BMPR2
A_33_P3312638	3.3013756	down	NM_001005851	chr19:040540162-04	ZNF780B
A_33_P3385765	2.0354362	down	NM_001012753	chr19:012091030-01	ZNF763

Homo sapiens Fanco	GO:0005515|GO:000	NM_018062	Hs.720331	55120	709.94916
Homo sapiens calmo	GO:0005874	NM_203459	Hs.23585	23271	317.52536
Homo sapiens ubiqui	GO:0005515|GO:000	NM_001039590	Hs.77578	8239	182.18703
Homo sapiens zinc fi	GO:0005622|GO:000	NM_144566	Hs.528486	90592	97.17095
Homo sapiens pseud	GO:0009982|GO:000	NM_031292	Hs.445814	83448	28.800224
Homo sapiens zinc fi	GO:0005622|GO:000	NM_133473	Hs.687547	170959	16.72401
Homo sapiens biorie	GO:0003677	NM_148894	Hs.444517	259282	303.97552
Homo sapiens nucle	GO:0005622|GO:000	NM_005124	Hs.601591	9972	2704.9036
Homo sapiens DEP d	GO:0005622|GO:000	NM_001114120	Hs.445098	55635	153.94946
Homo sapiens KDEL	GO:0005783|GO:000	NM_153705	Hs.83286	143888	3260.7715
Homo sapiens enable	GO:0017124|GO:000	NM_001008493	Hs.497893	55740	24.550379
Homo sapiens septin	GO:0031105|GO:000	NM_207366	Hs.453629	346288	11.0224695
Homo sapiens export	GO:0005515|GO:000	NM_022459	Hs.507452	64328	234.23035
Homo sapiens RAN	GO:0005515|GO:000	NM_006267	Hs.199561	5903	124.724304
Homo sapiens SHC S	GO:0005515|GO:004	NM_024745	Hs.123253	79801	730.35065
Homo sapiens leptin	GO:0005515|GO:004	NM_001003679	Hs.23581	3953	63.454975
Homo sapiens DnaJ (	GO:0006260|GO:000	NM_014377	Hs.558476	27000	649.709
Homo sapiens mRNA; cDNA DKFZp586B0319 (from clone DK			Hs.667735	26069	97.22123
Homo sapiens muscl	GO:0005737|GO:000	NM_144778	Hs.657347	10150	796.62415
Homo sapiens sialic acid binding Ig-like le		NR_002804	Hs.132045	284367	48.744667
Homo sapiens UDP-l	GO:0004653|GO:003	NM_017423	Hs.548088	51809	919.0174
Centriolin (110 kDa centrosomal protein)(Centrosomal protein 1) [Source:UniProtKB/Swiss-Prot;Acc:Q7Z					16.849503
Homo sapiens phosp	GO:0016042|GO:000	NM_030821	Hs.389452	81579	106.65761
Homo sapiens interle	GO:0005138|GO:000	NM_002184	Hs.532082	3572	463.0868
Homo sapiens nuclea	GO:0005515|GO:000	NM_020772	Hs.462598	57532	292.58456
Zinc finger protein 72 (Krueppel-related zi		XM_002343229	Hs.721896		23.03154
Homo sapiens myosi	GO:0005516|GO:003	NM_014981	Hs.225968	22989	5.1556945
Homo sapiens protei	GO:0005515|GO:000	NM_002480	Hs.49582	4659	1784.1576
Homo sapiens UBX	GO:0006986|GO:000	NM_014607	Hs.591242	23190	637.84894
Homo sapiens fatty a	GO:0005515|GO:000	NM_032228	Hs.501991	84188	204.76651
Homo sapiens nuclea	GO:0042826|GO:001	NM_003489	Hs.155017	8204	65.49199
Homo sapiens U2-as	GO:0006396|GO:000	NM_001080415	Hs.596572	23350	120.024216
Shugoshin-like 2 (Shugoshin-2)(Sgo2)(Tripin) [Source:UniProtKB/Swiss-Prot;Acc:Q562F6] [ENST00000					17.979881
Homo sapiens LUC7	GO:0008380|GO:000	NM_016424	Hs.130293	51747	2254.5803
Homo sapiens centro	GO:0005694|GO:000	NM_001812	Hs.479867	1060	119.479164
Homo sapiens memb	GO:0016020|GO:001	NM_138799	Hs.467634	129642	169.82977
Homo sapiens leucin	GO:0005515	NM_001099678	Hs.518084	116064	402.6776
Rho GTPase-activating protein SYDE2 (Synapse defective protein 1 homolog 2)(Protein syd-1 homolog 2)					141.34644
Homo sapiens chrom	GO:0006626|GO:000	NM_032511	Hs.573245	84553	5.9125524
Homo sapiens phosp	GO:0008286|GO:000	NM_181523	Hs.132225	5295	105.57379
Homo sapiens zinc fi	GO:0005622|GO:000	NM_152603	Hs.412517	163081	506.42316
Homo sapiens solute	GO:0005215|GO:000	NM_052885	Hs.558595	114134	10.308029
Homo sapiens G2/M-	GO:0000209|GO:000	NM_017769	Hs.509008	55632	31.88487
Homo sapiens stroma	GO:0005515|GO:000	NM_005862	Hs.412586	10274	159.31557
Homo sapiens KIAA1841 (KIAA1841), tr		NM_001129993	Hs.468653	84542	10.422431
Homo sapiens BCL2	GO:0016564|GO:000	NM_014739	Hs.486542	9774	2090.7131
Homo sapiens NMD	GO:0005515|GO:000	NM_057175	Hs.555985	80155	120.136894
Homo sapiens golgin	GO:0005515|GO:000	NM_001146039	Hs.183702	92344	11.129669
Homo sapiens alkali	GO:0070774|GO:000	NM_018367	Hs.23862	55331	110.98207
Homo sapiens zinc fi	GO:0005622|GO:000	NM_001005851	Hs.599728	163131	15.385919
Homo sapiens Rho-a	GO:0005515|GO:000	NM_004850	Hs.591600	9475	81.835464
Homo sapiens dynei	GO:0005929|GO:003	NM_001373	Hs.133977	127602	12.68185
Homo sapiens oxyste	GO:0006869|GO:000	NM_148909	Hs.21938	114883	1549.4934
Homo sapiens TRAF	GO:0005515|GO:000	NM_015028	Hs.34024	23043	6.506973
Homo sapiens methy	GO:0005634|GO:000	NM_018328	Hs.458312	55777	22.684252
Homo sapiens plecks	GO:0005622|GO:000	NM_015310	Hs.434255	23362	128.0772
Homo sapiens sperm	GO:0005515|GO:004	NM_001130528	Hs.463439	9043	63.074932
Homo sapiens bone	GO:0014916|GO:000	NM_001204	Hs.471119	659	93.5086
Homo sapiens zinc fi	GO:0005622|GO:000	NM_001005851	Hs.599728	163131	8.962624
Homo sapiens zinc fi	GO:0005622|GO:000	NM_001012753	Hs.646386	284390	5269.635

1768.9156	9.618254	10.679674
1132.0665	8.465364	10.023155
693.71625	7.6546907	9.301447
311.91898	6.7343907	8.129095
95.92954	4.9904585	6.4455013
51.218605	4.1992292	5.5526533
2018.9883	8.404089	10.875931
6138.4116	11.477634	12.548196
412.9535	7.3979826	8.531353
7732.915	11.734968	12.883846
75.76638	4.758422	6.117446
27.967453	3.6128626	4.668419
713.4093	8.026703	9.340348
786.0732	7.0979633	9.476158
1879.8177	9.653717	10.774532
165.60497	6.1137094	7.2380095
2540.0803	9.48646	11.214851
545.1147	6.7353716	8.940903
3320.0012	9.783971	11.617153
116.5247	5.7274103	6.7321653
2501.198	9.96606	11.194857
65.043785	4.211545	5.901288
266.25055	6.8638563	7.900873
1555.6626	9.005299	10.490448
892.0605	8.348745	9.659978
80.140816	4.660993	6.1951685
13.895827	2.5629086	3.6499484
4622.0996	10.903394	12.118271
1633.3544	9.459729	10.561813
658.2878	7.8323827	9.219736
219.87111	6.1616707	7.6386676
355.57675	7.0352345	8.317909
51.195297	4.303074	5.551851
8161.6016	11.230688	12.967121
589.4311	7.0297213	9.057435
504.2942	7.545847	8.828512
1395.8462	8.803854	10.334075
370.55173	7.276409	8.37421
21.306501	2.7539284	4.2775564
435.13144	6.8505363	8.611019
1357.8024	9.128395	10.287165
27.045015	3.5206394	4.6219845
105.19889	5.1311626	6.5800343
466.4494	7.448866	8.711599
44.652676	3.5338192	5.3491697
9060.502	11.12322	13.125132
348.08124	7.0362973	8.28553
33.147068	3.6245198	4.908265
295.30154	6.921889	8.052213
44.86658	4.076835	5.35598
497.31476	6.493354	8.80847
52.97503	3.8056045	5.6033535
3777.9966	10.702518	11.824273
20.042118	2.8832116	4.1900244
57.805183	4.636974	5.733521
399.8403	7.1311846	8.48727
151.57428	6.101054	7.1095266
415.7767	6.682268	8.542212
36.467598	3.32728	5.0503473
11112.954	12.405592	13.43093

A_23_P40866	5.4652103	down	NM_015642	chr3:114057749-114	ZBTB20
A_33_P3327165	3.7463287	down	NM_206886	chr1:093730275-093	CCDC18
A_23_P138655	2.6519999	down	NM_057157	chr10:094837013-09	CYP26A1
A_23_P316150	3.3914765	down	NM_020800	chr3:159975086-159	IFT80
A_33_P3799692	4.6378656	down	BC043009	chr10:33176365-331	LOC338620
A_24_P411749	2.0447933	down	NM_020455	chr6:142766903-142	GPR126
A_23_P148204	3.0133599	down	NM_017944		USP47
A_33_P3270429	2.1841991	down	BC070035	chr3:24004736-24004795	
A_33_P3386459	2.5079172	down	NM_001030055	chr14:032586421-03	ARHGAP5
A_33_P3373892	2.056706	down	AK056059	chr9:88601845-88601904	
A_33_P3217689	2.7926152	down	NM_152405	chr5:078622942-078	JMY
A_24_P61864	2.070118	down	NM_020198		CCDC47
A_33_P3419865	2.5926891	down	XR_041071	chr5:036864662-036	LOC646719
A_23_P201376	2.5748932	down	NM_014021	chr1:085109841-085	SSX2IP
A_33_P3314212	2.0722916	down	NM_003631	chr10:051363266-05	PARG
A_24_P402898	2.1855706	down	NM_199324	chr4:146055623-146	OTUD4
A_23_P44665	2.5728376	down	NM_145004		ADAM32
A_24_P213548	2.3149467	down	NM_006699	chr1:118039385-118	MAN1A2
A_23_P356684	2.9821979	down	NM_018685	chr7:036492587-036	ANLN
A_23_P24244	3.4765251	down	NM_017782		C10orf18
A_33_P3271445	2.6347973	down	NM_001130105	chr5:074675230-074	COL4A3BP
A_33_P3735158	3.8652687	down	AK093004	chr9:69135853-6913	LOC286272
A_23_P354387	2.4876526	down	NM_013451	chr10:095066378-09	MYOF
A_33_P3419808	2.0378544	down	XM_002345473	chr22:23011219-230	LOC100293142
A_23_P48535	2.8337687	down	NM_001030055	chr14:032624171-03	ARHGAP5
A_33_P3352268	2.1216266	down		chr10:134243928-134243869	
A_33_P3240773	2.4605632	down	BC043599	chr3:10057193-1005	CIDEC
A_23_P21409	2.6597359	down	NM_001042384	chr3:134268989-134	CEP63
A_23_P44760	2.9137732	down	NM_014159	chr3:047058639-047	SETD2
A_33_P3240348	2.6459185	down	NM_000267	chr15:022148392-02	NF1
A_23_P7056	2.1627464	down	NM_018115	chr4:076871443-076	SDAD1
A_33_P3304304	2.2224641	down		chrX:123097146-123097205	
A_23_P314222	3.6164219	down	NM_138792		LEO1
A_23_P82379	2.3527979	down	NM_000722	chr7:081579504-081	CACNA2D1
A_23_P31399	2.1936879	down	NM_000305	chr7:095034559-095	PON2
A_33_P3284453	2.0526092	down	NM_001012279	chr6:127771389-127	C6orf174
A_24_P310630	3.273221	down	NM_080632	chrX:118968535-118	UPF3B
A_33_P3287735	2.0798027	down	NM_001099412	chr8:041787702-041	MYST3
A_32_P38623	3.2137491	down	NM_017650	chr7:094925547-094	PPP1R9A
A_33_P3234657	6.7229681	down	NM_173814	chr15:055911666-05	PRTG
A_23_P406986	2.2165998	down	NM_152581	chrX:014939335-014	MOSPD2
A_24_P373286	3.1974165	down	NM_014711	chr16:019564500-01	CP110
A_33_P3221134	3.8120261	down		chr2:069826310-069826369	
A_33_P3254291	3.0398499	down	NM_001008225	chr7:135072846-135	CNOT4
A_24_P943949	3.0593151	down	NM_015350	chr1:090062798-090	LRRC8B
A_33_P3325439	2.6826029	down	NM_001001992	chr21:030403034-03	USP16
A_23_P150189	2.4474443	down	NM_005590	chr3:155830315-155	MRE11A
A_24_P179183	6.0448783	down	NM_015208	chr18:009254599-00	ANKRD12
A_33_P3483909	2.4768639	down	AK127155	chr11:36291254-36291195	
A_23_P168771	4.1316421	down	NM_020879	chr7:076924182-076	CCDC146
A_23_P23266	2.7199611	down	U79751	chr1:169356909-169	BLZF1
A_24_P941487	2.8688241	down	NM_001008401	chr19:053960813-05	ZNF761
A_24_P28295	6.7873137	down	AB019490	chr1:174959004-174	RABGAP1L
A_33_P3209135	2.0574472	down	XM_001720086	chr6:170581441-170	LOC285804
A_24_P346855	3.9671666	down	NM_002417	chr10:129895700-12	MKI67
A_33_P3862375	3.0641848	down	NM_001080481	chr6:099880709-099	USP45
A_33_P3612589	4.8287153	down	NM_000489	chrX:076761400-076	ATRX
A_33_P3351356	2.1514901	down		chr8:1851609-1851668	
A_23_P340308	3.4757051	down	NM_176888	chr12:011174380-01	TAS2R19
A_23_P59855	2.5596827	down	NM_006524	chr7:064293564-064	ZNF138

Homo sapiens zinc fi	GO:0005622|GO:000	NM_015642	Hs.655108	26137	10.656669
Homo sapiens coiled-coil domain containi		NM_206886	Hs.716682	343099	63.825638
Homo sapiens cytoch	GO:0005792|GO:000	NM_057157	Hs.150595	1592	4.9999995
Homo sapiens intrafl	GO:0005737|GO:000	NM_020800	Hs.478095	57560	195.17584
Homo sapiens hypothetical protein LOC338620, mRNA (cDNA			Hs.660499	338620	43.97386
Homo sapiens G prot	GO:0005886|GO:000	NM_020455	Hs.318894	57211	583.6679
Homo sapiens ubiqui	GO:0006511|GO:000	NM_017944	Hs.577256	55031	248.24203
Nuclear receptor subfamily 1 group D member 2 (Rev-erb-beta)(			Hs.37288		8.142584
Homo sapiens Rho G	GO:0008361|GO:003	NM_001030055	Hs.592313	394	45.12261
Protein MAK10 homolog (Embryonic growth-associated protein			Hs.436098		77.00924
Homo sapiens junctio	GO:0005515|GO:005	NM_152405	Hs.482605	133746	107.84555
Homo sapiens coiled	GO:0005515|GO:000	NM_020198	Hs.202011	57003	532.09784
PREDICTED: Homo sapiens hypothetical		XR_041071	Hs.697792	646719	24.6991
Homo sapiens synovi	GO:0005515|GO:004	NM_014021	Hs.22587	117178	74.55383
Homo sapiens poly (.	GO:0005737|GO:001	NM_003631	Hs.536158	8505	36.83147
Homo sapiens OTU (	GO:0005515|GO:000	NM_199324	Hs.270851	54726	176.05254
Homo sapiens ADAM	GO:0016020|GO:000	NM_145004	Hs.521545	203102	4.9999995
Homo sapiens mannc	GO:0005794|GO:000	NM_006699	Hs.435938	10905	16.136814
Homo sapiens anillin	GO:0000910|GO:000	NM_018685	Hs.62180	54443	3488.462
Homo sapiens chromosome 10 open readir		NM_017782	Hs.432548	54906	707.3783
Homo sapiens collag	GO:0005515|GO:003	NM_001130105	Hs.270437	10087	567.971
Homo sapiens cDNA FLJ35685 fis, clone SPLEN2019257 [AK(			Hs.648390	286272	7.781565
Homo sapiens myofe	GO:0034605|GO:000	NM_013451	Hs.602086	26509	8811.676
V2-19 protein Fragment [Source:UniProtK		XM_002345473		100293142	8.266333
Homo sapiens Rho G	GO:0008361|GO:003	NM_001030055	Hs.592313	394	126.47801
Putative uncharacterized protein ENSP00000346801 Fragment [Source:UniProtKB/TrEMBL;Acc:A6NE6					14.132941
Homo sapiens cell de	GO:0005622|GO:0005515|GO:0003674|G		Hs.635072	63924	16.91174
Homo sapiens centro	GO:0005515|GO:000	NM_001042384	Hs.443301	80254	321.6495
Homo sapiens SET d	GO:0005515|GO:000	NM_014159	Hs.517941	29072	129.8568
Homo sapiens neuro	GO:0001656|GO:000	NM_000267	Hs.113577	4763	24.123552
Homo sapiens SDA1	GO:0005515|GO:004	NM_018115	Hs.632604	55153	51.81703
Cohesin subunit SA-2 (Stromal antigen 2)(SCC3 homolog 2) [Source:UniProtKB/Swiss-Prot;Acc:Q8N3U4					4.9999995
Homo sapiens Leo1,	GO:0005515|GO:003	NM_138792	Hs.567662	123169	460.98273
Homo sapiens calciu	GO:0005515|GO:001	NM_000722	Hs.282151	781	4.9999995
Homo sapiens parao	GO:0019439|GO:001	NM_000305	Hs.530077	5445	226.66144
Homo sapiens chrom	GO:0016020|GO:001	NM_001012279	Hs.319247	387104	82.11508
Homo sapiens UPF3	GO:0005515|GO:000	NM_080632	Hs.103832	65109	392.66522
Homo sapiens MYST	GO:0070776|GO:000	NM_001099412	Hs.491577	7994	160.72974
Homo sapiens protei	GO:0005515|GO:000	NM_017650	Hs.21816	55607	35.993214
Homo sapiens protog	GO:0016020|GO:000	NM_173814	Hs.130957	283659	4.9999995
Homo sapiens motile	GO:0005886|GO:000	NM_152581	Hs.190043	158747	29.32502
Homo sapiens CP11(	GO:0005515|GO:003	NM_014711	Hs.279912	9738	129.69681
					10.41825
Homo sapiens CCR4	GO:0005515|GO:000	NM_001008225	Hs.490224	4850	18.896742
Homo sapiens leucin	GO:0005515|GO:001	NM_015350	Hs.482017	23507	4.9999995
Homo sapiens ubiqui	GO:0043130|GO:000	NM_001001992	Hs.99819	10600	270.67084
Homo sapiens MRE1	GO:0008408|GO:003	NM_005590	Hs.192649	4361	345.1861
Homo sapiens ankyri	GO:0005634	NM_015208	Hs.464585	23253	137.1058
Putative uncharacterized protein C11orf55 [Source:UniProtKB/			Hs.99443		4.9999995
Homo sapiens coiled-coil domain containi		NM_020879	Hs.113940	57639	43.609165
Golgin-45 (Basic leu	GO:0005737|GO:0005794|GO:0003700|G		Hs.130746	8548	183.18802
Homo sapiens zinc fi	GO:0005622|GO:000	NM_001008401	Hs.433293	388561	80.91381
RAB GTPase-activat	GO:0005622|GO:0032313|GO:0005097|G		Hs.585378	9910	4.9999995
PREDICTED: Homo sapiens hypothetical		XM_001720086		285804	7.295249
Homo sapiens antige	GO:0005622|GO:000	NM_002417	Hs.80976	4288	177.26944
Homo sapiens ubiqui	GO:0006511|GO:000	NM_001080481	Hs.143410	85015	84.56666
Homo sapiens alpha	GO:0005515|GO:000	NM_000489	Hs.533526	546	19.188288
Rho guanine nucleotide exchange factor 10 [Source:UniProtKB/Swiss-Prot;Acc:O15013] [ENST0000039					58.897835
Homo sapiens taste r	GO:0007165|GO:005	NM_176888	Hs.688196	259294	16.80684
Homo sapiens zinc fi	GO:0005622|GO:000	NM_006524	Hs.184080	7697	109.841194

70.434845	3.565393	6.01567
290.44308	6.1222515	8.027729
14.733019	2.3362613	3.743342
808.93805	7.7539005	9.515814
246.68001	5.582759	7.79622
1418.582	9.325226	10.357181
922.3684	8.111583	9.702956
21.89895	3.1930366	4.320141
134.461	5.6168203	6.94331
190.72325	6.4011974	7.441533
366.87772	6.8810062	8.362623
1318.0176	9.198748	10.248461
77.01253	4.7660704	6.1405196
233.26332	6.35581	7.7203226
91.99839	5.336249	6.387476
470.87085	7.596176	8.724186
14.343481	2.3362613	3.6996217
44.938576	4.1478705	5.3588495
10918.571	11.826868	13.403244
2886.4775	9.612829	11.410475
1772.6298	9.286268	10.68396
37.413837	3.1355813	5.08615
21849.387	13.125658	14.440443
20.766893	3.2135563	4.2406073
437.56558	7.11594	8.618662
36.23357	3.9543266	5.0394974
49.87027	4.216685	5.5156736
1047.591	8.484589	9.895872
460.8042	7.1517544	8.694643
76.66393	4.7313204	6.135089
133.32315	5.8176975	6.930562
12.492511	2.3362613	3.4884214
1985.0372	8.998548	10.853111
13.18595	2.3362613	3.5706387
613.7828	7.9816747	9.115033
204.45197	6.498887	7.536346
1546.895	8.77099	10.481701
408.3402	7.4612303	8.517677
139.2106	5.306123	6.9903803
37.38038	2.3362613	5.0853596
78.232216	5.0125427	6.160891
503.6266	7.1497893	8.826696
48.317642	3.5333235	5.4638815
68.69432	4.3755236	5.9795237
16.955328	2.3362613	3.94947
891.8058	8.2344265	9.65806
1034.7579	8.587481	9.878757
1001.0329	7.2335567	9.82927
13.83116	2.3362613	3.6447759
216.23903	5.5694532	7.6161685
610.0343	7.661852	9.105438
285.13763	6.47815	7.9986095
37.71038	2.3362613	5.099102
18.55489	3.0391421	4.0799975
856.99677	7.609769	9.597878
317.55917	6.5374107	8.152914
111.86559	4.3967996	6.668439
151.4554	6.0031614	7.1084976
69.92482	4.2069883	6.004294
341.89917	6.908184	8.264149

A_23_P434347	2.0360524	down	NM_147152	chr2:024469070-024	ITSN2
A_24_P487736	2.1849435	down	NM_198279	chrX:019934553-01	CXorf23
A_33_P3432961	2.8579891	down	BX648714	chr7:30588083-3058	LOC222159
A_33_P3327608	3.0043646	down	NM_001039753	chr2:055197718-055	EML6
A_33_P3370310	3.1587177	down		chr8:93933791-93933732	
A_23_P376239	2.9956893	down	NM_032632		PAPOLA
A_33_P3301097	2.1294089	down		chr4:003912744-003912685	
A_33_P3245517	4.8587052	down	NR_024380	chr14:019167900-01	LOC441666
A_23_P97810	2.0671317	down	NM_003631	chr10:051027148-05	PARG
A_23_P411881	3.0315918	down	NM_006346		PIBF1
A_33_P3280346	2.1996249	down	AK097050	chr15:32268641-322	LOC100130857
A_33_P3710442	2.1464094	down	AK021772	chr17:55357314-553	FLJ11710
A_24_P225907	2.2915361	down	NM_206831	chr3:016299693-016	DPH3
A_33_P3224212	2.2656443	down	NM_015361	chr2:136481550-136	R3HDM1
A_33_P3301940	2.3572267	down	NM_018945	chr6:136516635-136	PDE7B
A_23_P36865	3.9088982	down	NM_025114	chr12:088442894-08	CEP290
A_23_P149775	2.8659054	down	NM_018287	chr10:032095364-03	ARHGAP12
A_33_P3299854	2.1599891	down	NM_002486	chr9:100435948-100	NCBP1
A_33_P3273266	2.3372774	down	NR_024380	chr9:069847491-069	LOC441666
A_24_P161813	2.5161065	down	NR_024380	chr18:015271194-01	LOC441666
A_23_P22548	2.1673643	down	NM_000390	chrX:085117224-08	CHM
A_33_P3502037	2.0841657	down	NM_012124	chr18:002945693-00	CHORDC1
A_23_P134755	2.8031404	down	NM_014112	chr8:116421052-116	TRPS1
A_33_P3394252	2.3960594	down	NM_152519	chr2:210885586-210	C2orf67
A_23_P145874	3.2136709	down	NM_152703	chr7:092760278-092	SAMD9L
A_23_P364465	2.1206272	down	NM_022913	chr5:056558753-056	GPBP1
A_32_P167904	2.6470833	down	NM_138286	chr19:023922803-02	ZNF681
A_24_P291231	2.0921897	down	NM_016831	chr1:007904641-007	PER3
A_23_P111343	4.3348483	down	NM_014739	chr6:136582580-136	BCLAF1
A_33_P3294578	2.0894366	down	NM_002740	chr3:170023598-170	PRKCI
A_23_P7253	2.7494419	down	NM_018078	chr4:129131868-129	LARP1B
A_33_P3419139	2.0771804	down	NM_001127211	chr10:118645949-11	KIAA1598
A_33_P3380211	6.4784267	down	NM_005751	chr7:091709254-091	AKAP9
A_33_P3279519	2.2909886	down	XR_078302	chr4:063683332-063	LOC644548
A_33_P3262794	2.5450742	down	NM_017661	chr15:056969832-05	ZNF280D
A_33_P3377060	2.0108438	down	NM_003144	chr6:007287863-007	SSR1
A_33_P3338539	2.4891989	down		chr2:098167578-098167519	
A_33_P3257973	3.031908	down	NM_014877	chr17:065066671-06	HELZ
A_24_P396231	2.8564158	down	NM_002294	chrX:119560132-11	LAMP2
A_24_P880043	2.8776836	down	NM_032373	chr10:093043866-09	PCGF5
A_24_P170774	3.6500279	down	NM_015116	chr13:047317618-04	LRCH1
A_23_P311885	2.8035179	down	NM_032438	chr6:130462056-130	L3MBTL3
A_23_P28042	2.2343135	down	NM_001159293	chr19:020727423-02	ZNF737
A_33_P3410507	2.4391813	down	NM_014812	chr1:243288171-243	CEP170
A_33_P3408237	5.3755561	down		chr11:043488922-043488981	
A_32_P160883	2.1236147	down	NM_198400	chr15:056119580-05	NEDD4
A_23_P17204	2.4084334	down	NM_022662	chr2:112526856-112	ANAPC1
A_23_P364107	3.2055435	down	NM_018353	chr14:045673272-04	C14orf106
A_23_P88209	2.3055213	down	NM_016106	chr14:031204866-03	SCFD1
A_23_P156880	2.0138881	down	NM_006208	chr6:132211871-132	ENPP1
A_24_P702813	3.8315619	down	NM_004736	chr1:180859219-180	XPR1
A_33_P3242483	2.8935226	down	NM_001030055	chr14:032628828-03	ARHGAP5
A_23_P378416	3.2151655	down	NM_001001996	chrX:013792323-01	GPM6B
A_23_P419795	2.3204973	down	NM_030962	chr11:009800909-00	SBF2
A_33_P3343390	2.1696355	down	AF289562	chr22:39872483-39872542	
A_24_P743907	2.9580306	down	NM_012062	chr12:032898016-03	DNM1L
A_23_P391778	2.4875025	down	NM_033044	chr1:039951624-039	MACF1
A_33_P3302777	2.0559031	down	NM_004734	chr13:036686066-03	DCLK1
A_33_P3391076	3.5055871	down	NM_207304	chr13:097999137-09	MBNL2
A_33_P3229477	3.4696922	down	NM_173496	chr10:028339990-02	MPP7

Homo sapiens interse	GO:0005622|GO:000	NM_147152	Hs.432562	50618	38.04122
Homo sapiens chrom	GO:0005739	NM_198279	Hs.28896	256643	65.24769
Homo sapiens mRNA; cDNA DKFZp686D21117 (from clone D			Hs.561708	222159	23.23433
Homo sapiens echinc	GO:0005737|GO:000	NM_001039753	Hs.656692	400954	4.9999995
UPF0599 protein C8orf83 [Source:UniProtKB/Swiss-Prot;Acc:Q629K1] [ENST00000378861]					8.237083
Homo sapiens poly(A	GO:0006378|GO:000	NM_032632	Hs.253726	10914	739.20953
					27.637197
Homo sapiens zinc finger protein 91 pseud		NR_024380		441666	5.951559
Homo sapiens poly (	GO:0005737|GO:001	NM_003631	Hs.536158	8505	58.565117
Homo sapiens progesterone immunomodul		NM_006346	Hs.441926	10464	222.56763
Homo sapiens cDNA FLJ39731 fis, clone SMINT2015745. [AK			Hs.636752	100130857	7.536398
Homo sapiens cDNA FLJ11710 fis, clone HEMBA1005149 [AI			Hs.657294	79904	9.545275
Homo sapiens DPH3	GO:0005515|GO:000	NM_206831	Hs.388087	285381	1170.254
Homo sapiens R3H d	GO:0003676	NM_015361	Hs.412462	23518	41.75916
Homo sapiens phospl	GO:0007165|GO:001	NM_018945	Hs.594417	27115	106.282486
Homo sapiens centro	GO:0005515|GO:000	NM_025114	Hs.150444	80184	107.42388
Homo sapiens Rho G	GO:0005622|GO:000	NM_018287	Hs.499264	94134	457.02316
Homo sapiens nuclea	GO:0008380|GO:000	NM_002486	Hs.595669	4686	582.3903
Homo sapiens zinc finger protein 91 pseud		NR_024380	Hs.721896	441666	759.4195
Homo sapiens zinc finger protein 91 pseud		NR_024380	Hs.721896	441666	258.48026
Homo sapiens choroi	GO:0005515|GO:001	NM_000390	Hs.496449	1121	79.35223
Homo sapiens cystei	GO:0008150|GO:000	NM_012124	Hs.22857	26973	403.30978
Homo sapiens trichoi	GO:0043565|GO:001	NM_014112	Hs.657018	7227	20.28309
Homo sapiens chromosome 2 open reading		NM_152519	Hs.591638	151050	5.479707
Homo sapiens sterile alpha motif domain c		NM_152703	Hs.489118	219285	58.354546
Homo sapiens GC-ri	GO:0005737|GO:000	NM_022913	Hs.444279	65056	2513.403
Homo sapiens zinc fi	GO:0005622|GO:000	NM_138286	Hs.399952	148213	29.965569
Homo sapiens period	GO:0007165|GO:000	NM_016831	Hs.162200	8863	35.525772
Homo sapiens BCL2	GO:0016564|GO:000	NM_014739	Hs.486542	9774	1023.6921
Homo sapiens protei	GO:0005515|GO:000	NM_002740	Hs.478199	5584	970.0806
Homo sapiens La rib	GO:0003723	NM_018078	Hs.657067	55132	18.50034
Homo sapiens KIAA	GO:0030424|GO:000	NM_001127211	Hs.501140	57698	49.84336
Homo sapiens A kina	GO:0007165|GO:000	NM_005751	Hs.651221	10142	29.799404
PREDICTED: Homo sapiens similar to SE		XR_078302	Hs.631935	644548	59.73147
Homo sapiens zinc fi	GO:0005622|GO:000	NM_017661	Hs.511477	54816	15.626878
Homo sapiens signal	GO:0008284|GO:001	NM_003144	Hs.114033	6745	9398.095
					5.4941015
Homo sapiens helica	GO:0016787|GO:000	NM_014877	Hs.370140	9931	255.35413
Homo sapiens lysoso	GO:0031088|GO:000	NM_002294	Hs.496684	3920	176.85703
Homo sapiens polycc	GO:0005515|GO:000	NM_032373	Hs.500512	84333	618.0974
Homo sapiens leucin	GO:0005515	NM_015116	Hs.507971	23143	11.894309
Homo sapiens l(3)mb	GO:0003674|GO:000	NM_032438	Hs.658051	84456	14.303931
Homo sapiens zinc fi	GO:0005622|GO:000	NM_001159293	Hs.515696	100129842	192.72548
Homo sapiens centro	GO:0005737|GO:000	NM_014812	Hs.533635	9859	201.42813
					13.980981
Homo sapiens neural	GO:0044111|GO:004	NM_198400	Hs.1565	4734	374.89246
Homo sapiens anaph	GO:0005515|GO:000	NM_022662	Hs.436527	64682	3840.1404
Homo sapiens chrom	GO:0007067|GO:000	NM_018353	Hs.437941	55320	367.93954
Homo sapiens sec1 f	GO:0005801|GO:000	NM_016106	Hs.369168	23256	1852.1904
Homo sapiens ectonu	GO:0005515|GO:003	NM_006208	Hs.527295	5167	1585.9506
Homo sapiens xenotr	GO:0007186|GO:000	NM_004736	Hs.227656	9213	103.81
Homo sapiens Rho G	GO:0008361|GO:003	NM_001030055	Hs.592313	394	42.220695
Homo sapiens glycop	GO:0003674|GO:001	NM_001001996	Hs.495710	2824	16.8704
Homo sapiens SET b	GO:0005515|GO:000	NM_030962	Hs.577252	81846	158.99895
Homo sapiens clone pp6337 unknown mRNA. [AF289562]			Hs.276808		4.9999995
Homo sapiens dynan	GO:0005515|GO:000	NM_012062	Hs.556296	10059	313.2002
Homo sapiens microt	GO:0005515|GO:000	NM_033044	Hs.580782	23499	2299.2297
Homo sapiens doubl	GO:0005515|GO:000	NM_004734	Hs.507755	9201	5.2186856
Homo sapiens muscl	GO:0005737|GO:000	NM_207304	Hs.657347	10150	18.92752
Homo sapiens memb	GO:0005515|GO:003	NM_173496	Hs.499159	143098	19.88421

93.16253	5.3807273	6.406502
171.12985	6.157094	7.28469
79.97491	4.677433	6.1924334
16.68779	2.3362613	3.9233212
32.171696	3.2096376	4.8689766
2605.617	9.671871	11.254759
70.69499	4.93056	6.021013
36.340805	2.7633114	5.0438833
144.039	5.994462	7.0420923
829.64105	7.9562445	9.55632
20.553741	3.0874062	4.2246637
25.038681	3.4125829	4.5145082
3063.1252	10.308812	11.505127
113.097786	5.5074396	6.687361
304.9964	6.859902	8.0969925
508.61218	6.875121	8.841883
1573.3596	8.986384	10.505375
1493.2401	9.32164	10.432664
2101.3738	9.714221	10.93905
800.1898	8.169996	9.501189
208.16481	6.4485383	7.56448
1026.699	8.805801	9.865271
67.963715	4.476124	5.963168
16.55347	2.6485949	3.9092586
225.61766	5.9896574	7.6738796
5816.1274	11.385953	12.470444
96.153244	5.0446568	6.4490604
89.65558	5.285927	6.3509407
4988.0586	10.119804	12.2357855
2353.1047	10.044485	11.107599
60.676247	4.3411837	5.8003225
123.37429	5.759736	6.8143625
235.6482	5.0376472	7.7332907
163.63745	6.02337	7.2193403
47.836533	4.10108	5.4487877
19162.674	13.217363	14.225164
17.12441	2.651649	3.9673305
953.1647	8.15426	9.754486
614.9536	7.604438	9.118644
2101.1223	9.414036	10.938944
52.171387	3.7136312	5.5815387
48.107185	3.969545	5.4567833
527.39526	7.7373524	8.897184
604.55347	7.805826	9.092223
90.74499	3.9414444	6.3678584
977.8742	8.701323	9.787845
9770.445	11.971371	13.239466
1418.3224	8.676392	10.356961
4745.716	10.953726	12.158819
3589.6335	10.7349415	11.744925
483.26694	6.8263154	8.764248
145.96342	5.5254254	7.0582523
64.87842	4.213158	5.898051
449.952	7.446432	8.660866
12.214858	2.3362613	3.453714
1122.7367	8.446498	10.011135
6223.864	11.258661	12.573359
13.620449	2.579575	3.6193473
79.63707	4.3771534	6.1868095
82.873505	4.4490833	6.243891

A_23_P156443	2.6369198	down	NM_002269	chr6:117050727-117	KPNA5
A_23_P151565	2.9609846	down	NM_014990	chr14:036007738-03	RALGAPA1
A_24_P130936	2.660329	down	NM_004660	chrY:015027862-015	DDX3Y
A_33_P3242623	3.5125798	down	NM_014331	chr4:139085492-139	SLC7A11
A_32_P162250	2.0834825	down	NM_033515	chr6:129898731-129	ARHGAP18
A_24_P165656	2.2072438	down	NM_005813	chr2:037478080-037	PRKD3
A_23_P209430	2.2416353	down	NM_020919	chr2:202566045-202	ALS2
A_33_P3259403	2.7893537	down	NM_175854	chr13:028866617-02	PAN3
A_33_P3798989	2.1238708	down	AK127847	chr11:129104412-12	FLJ45950
A_33_P3882624	2.2223073	down	NM_015450	chr7:124493120-124	POT1
A_33_P3358648	3.1678631	down	NM_001137552	chr2:238673547-238	LRRFIP1
A_32_P68148	2.9257652	down	NR_027130	chr19:021567123-02	ZNF738
A_23_P212497	2.0125221	down	NM_032169	chr3:132277719-132	ACAD11
A_24_P371962	2.3070943	down	NM_001634	chr6:111216818-111	AMD1

Homo sapiens karyo[	GO:0005515|GO:000(	NM_002269	Hs.182971	3841	10.963111
Homo sapiens Ral G'	GO:0005739|GO:000(	NM_014990	Hs.113150	253959	206.74745
Homo sapiens DEAD	GO:0008026|GO:000(	NM_004660	Hs.99120	8653	68.33731
Homo sapiens solute	GO:0015327|GO:001	NM_014331	Hs.390594	23657	2057.893
Homo sapiens Rho G	GO:0005622|GO:000(	NM_033515	Hs.486458	93663	329.78506
Homo sapiens protei	GO:0005515|GO:000(	NM_005813	Hs.660757	23683	1113.0879
Homo sapiens amyot	GO:0005515|GO:003	NM_020919	Hs.471096	57679	33.270153
Homo sapiens PAN3	GO:0005737|GO:000(	NM_175854	Hs.645015	255967	4.9999995
Homo sapiens cDNA FLJ45950 fis, clone PLACE7008136. [AK127847]				399975	117.038284
Homo sapiens POT1	GO:0051974|GO:000(	NM_015450	Hs.31968	25913	39.33442
Homo sapiens leucin	GO:0016564|GO:000(	NM_001137552	Hs.471779	9208	494.59006
Homo sapiens zinc fi	GO:0005622|GO:000(	NR_027130		148203	1352.8094
Homo sapiens acyl-C	GO:0050660|GO:000(	NM_032169	Hs.441378	84129	53.581013
Homo sapiens adeno:	GO:0008295|GO:000(	NM_001634	Hs.159118	262	4731.4917

35.36755	3.6043077	5.0031614
752.8577	7.847736	9.413813
219.1653	6.223179	7.6347837
7905.5303	11.099943	12.912474
845.88544	8.523186	9.582183
2837.3535	10.237512	11.379758
89.819954	5.189046	6.3535976
15.52677	2.3362613	3.8161922
302.9166	7.000284	8.08698
105.373405	5.4303007	6.582359
1862.6194	9.097811	10.761321
4481.1133	10.514484	12.063298
128.41872	5.8676834	6.876688
11417.711	12.261339	13.467416
